# Imprudent use of MalAvi names biases the estimation of parasite diversity of avian haemosporidians

**DOI:** 10.1371/journal.ppat.1012911

**Published:** 2025-02-05

**Authors:** Juliana Tamayo-Quintero, Josué Martínez-de la Puente, Nubia E. Matta, M. Andreína Pacheco, Héctor F. Rivera-Gutierrez

**Affiliations:** 1 Grupo de Investigación de Ecología y Evolución de Vertebrados, Instituto de Biología, Universidad de Antioquia Medellín, Colombia; 2 Estación Biológica de Doñana - CSIC, Sevilla, España; 3 Ciber de Epidemiología y Salud Pública (CIBERESP), Madrid, España; 4 Departamento de Biología, Facultad de Ciencias, Universidad Nacional de Colombia, Bogotá, Colombia; 5 Biology Department/Institute of Genomics and Evolutionary Medicine (iGEM), Temple University, Philadelphia, Pennsylvania, United States of America; University of Wisconsin Medical School, UNITED STATES OF AMERICA

## Overview

Understanding patterns of biota diversity at the different geographical scales is one of the major challenges of biogeography [1] and macroecology [2], especially for parasites, one of the most diverse groups on earth [3]. The order Haemosporida includes parasites of wildlife which are among the most studied in ecology and evolution [4,5]. In birds, haemosporidians include malaria parasites of the genus *Plasmodium* and related genera *Haemoproteus* and *Leucocytozoon* [6]. These parasites have been extensively studied in bird populations, mainly in Europe and North America, where authors have identified a high genetic diversity of parasites infecting wild bird species [7]. These parasite genera are an excellent study model for understanding the ecology and evolution of parasite–host interactions [7,8] and factors affecting the specificity and generalist strategies of infections.

## A quick review of the avian haemosporidian lineages worldwide in MalAvi database

The development of molecular tools drastically changed the knowledge of the diversity of parasites in wild birds. Since Bensch and colleagues [9], advances in molecular techniques have allowed the identification of 5,131 unique lineages of avian haemosporidians in more than 2,200 bird species worldwide (accession date: 07 June 2024). Authors have extensively defined and named unique lineages using a 479 base pairs (bp) fragment of the cytochrome b gene (*cyt-b*), which is used as barcode in these bird parasites [10]. For example, these lineages include generalist parasites such as the *Plasmodium relictum* SGS1, which infect more bird species than any other *Plasmodium* lineage [11], to lineages identified in a single host species. The goal of this genetic characterization is to use a region that is sufficiently informative and easy to study, allowing unambiguous identification of parasites for direct comparisons of parasite diversity between host species and geographic regions [12]. Based on the current information, the selected genetic region has been used as a proxy for parasite species [5], since they appear to be reproductively isolated entities [10,13]. However, there is still insufficient information to determine whether each of these haplotypes correspond to a different species, which highlights the need to further evaluate the diversity of this group not only molecularly but also morphologically [5].

The MalAvi database is the most comprehensive and widely used repository for lineages of avian haemosporidians worldwide [10]. This database provides detailed information of the known parasite lineages, hosts, and geographical distribution, among other important details [10,14]. However, this database includes numerous sequences with a length lower than the standard (<479 bp), which may be a consequence of prioritizing as much data as possible to have a representation of the diversity of these groups of avian parasites around the world. Although the shorter sequences are labeled as “partial” in the database, the inclusion of these lineages in macroecological studies complicates the standardization of unique lineage names associated with particular sequences, as synonymies can occur within lineages. Synonymies, defined as lineages with different names published as partial or, occasionally, full sequences of the partial *cyt-b* gene that are genetically indifferentiable between them, may increase the error of name assignment. Recognizing this drawback is important to evaluate the databases of studies including this information. Only using the name of parasite lineages infecting birds, instead of the corresponding sequences, may affect the estimation of parasite lineage diversity circulating in an area and the host range of lineages.

## The importance of considering sequences for studies of the parasite infection patterns

The diversity of avian haemosporidians has been widely documented worldwide [15,16]. Data from MalAvi has been frequently used to explore diversity and specificity patterns, providing valuable results [16,17]. These studies typically refer to each *cyt-b* haplotype as a unique parasite lineage, following the standard naming convention for these parasites [10,18]. However, given the potential bias introduced by short sequence synonymies, we aim to evaluate the name assignment criteria in the MalAvi database and provide potential guidelines for those using this platform.

**Fig 1:**
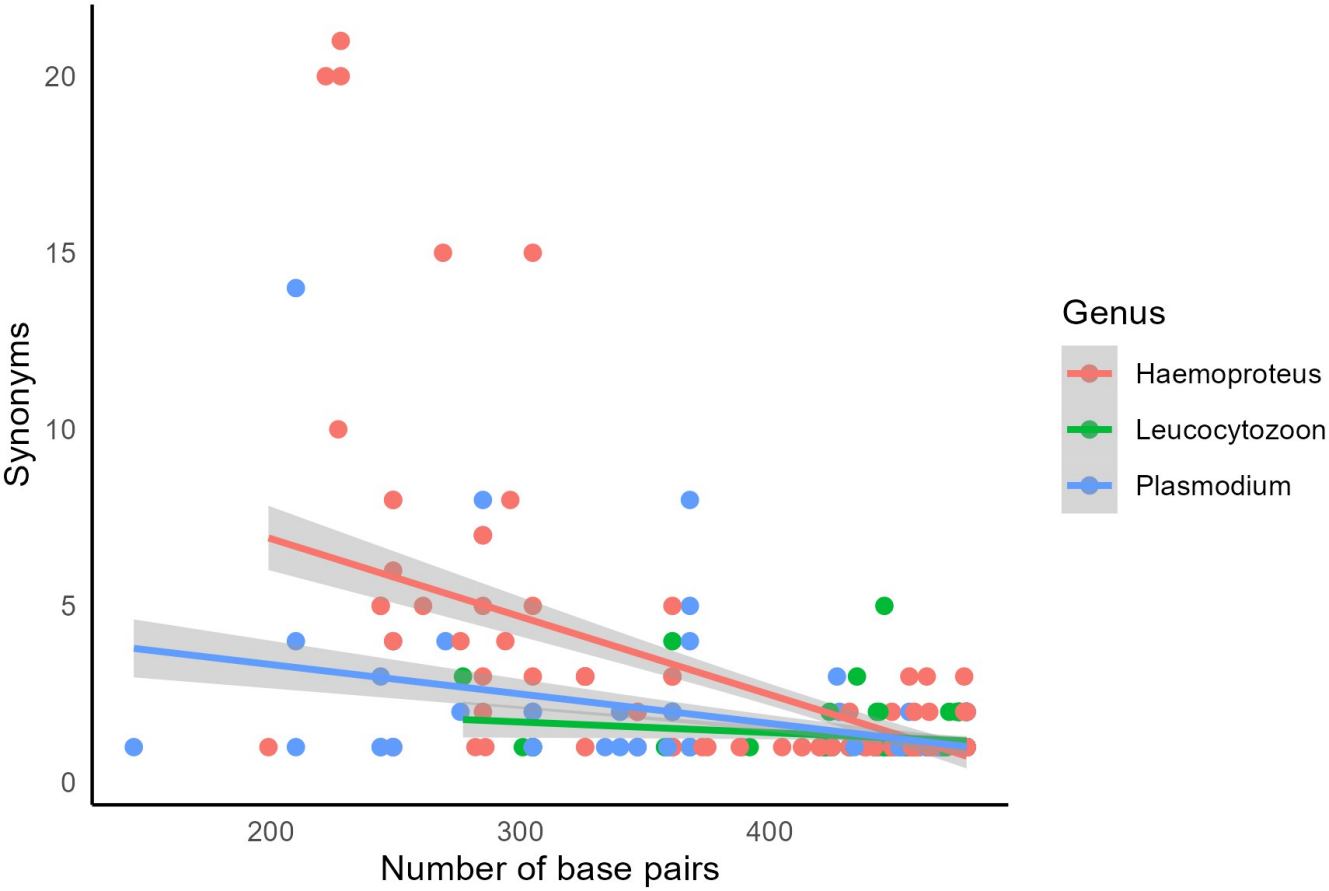
Relationship between the number of synonymies and the length (number of base pairs) of avian haemosporidian *cyt-b* sequences reported in MalAvi. Different colors correspond to the 3 genera analyzed.

We analyzed the occurrence of lineage synonymies in the complete MalAvi database (Fig A in S1 Supporting Information). These included 5,131 lineages (accession date: 07 June 2024) corresponding to: 1,559 lineages of *Plasmodium*, 2,030 lineages of *Haemoproteus*, and 1,542 lineages of *Leucocytozoon*. The data set includes partial sequences (*n* = 482) with a minimum length of 145 bp to sequences covering the whole barcoding region of at least 479 bp (*n* = 4,649). In total, 486 lineages have one to 21 synonymies, being more frequent in *Haemoproteus* (47.9%) and *Plasmodium* (30.7%) than in *Leucocytozoon* (21.4%). The occurrence of synonymies is negatively correlated with the length of sequences (Estimate = −0.06, z-value = 1,593.09, *p* < 0.001). A similar trend was found for the 3 parasite genera included in the database (all *p* < 0.05), suggesting that the relationship between the number of synonymies and the sequence length is consistent between them (Fig 1). For example, the most notable cases, which have a high number of synonymies, include sequences corresponding to the *Haemoproteus* lineages VIRFLA02 (228 bp), VIOLI15 (228 bp), VIRFLA03 (222 bp), PSADEC01 (305 bp), and MELGEO01 (269 bp), and the *Plasmodium* lineages RBQ16 (210 bp) and TABI08 (285 bp) (see further detail of the identical sequences in Table A in S1 Supporting Information). By contrast, synonymies rarely occur in the case of more extensive sequences, with only a few lineages with more than 440 pb showing from 3 to 5 synonymies (Fig 1).

**Fig 2:**
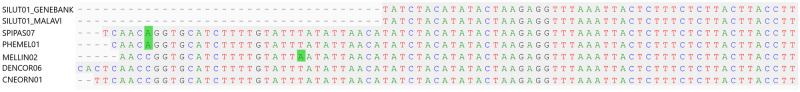
Visualization of the alignment close to N-terminal of the *cyt-b* gene synonymous sequences of the *Leucocytozoon* sp. lineage SILUT01. The 446 bp sequence reported in MalAvi and the 506 bp fragment from Genbank (EF153660) are included and compared with the 5 synonyms SPIPAS07, PHEMEL01, MELLIN02, DENCOR06, and CNEORN01. Differences between some of these lineages emerged in green when GenBank sequences were considered and compared with fragments included in MalAvi.

In some cases, these synonymies occur because the sequences included in Malavi, corresponding to the fragment of the barcode region, partially represent the complete sequence deposited in other databases such as GenBank. In this respect, differences between apparent identical sequences in MalAvi may emerge when considering the complete fragment of sequences deposited in Genbank (Fig 2). Synonymies are generated even though the GenBank sequence has sufficient information outside the barcode region.

**Fig 3:**
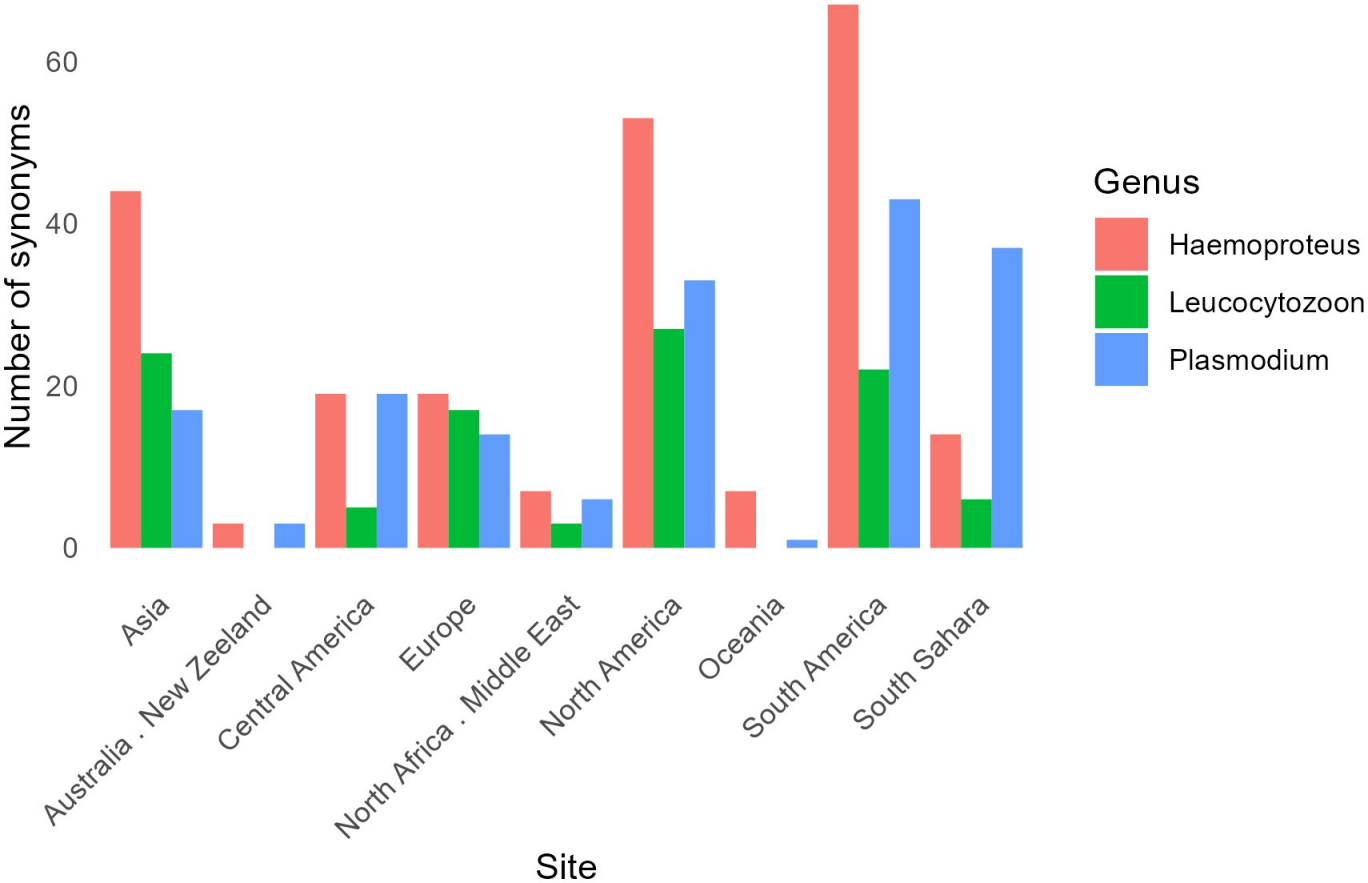
The number of parasites *cyt-b* gene lineages with synonymies reported by subcontinents. Different colors correspond to the 3 genera analyzed. Overall, 66 different lineages corresponding to *Haemoproteus* (23), *Leucocytozoon* (14), and *Plasmodium* (29) are found in more than one subcontinent. In these cases, lineages are included in each subcontinent.

Interestingly, the occurrence of synonymies in sequences included in MalAvi differs between geographical regions. The number of synonymies registered by subcontinents showed significant differences (*X*^*2*^ = 51.96, *p*-value < 0.001), being higher in South America (*n* = 132), especially for *Haemoproteus* and *Plasmodium* lineages (Fig 3). This suggests that as new lineages are being discovered in the most diverse region of earth, then special attention is required to minimize the occurrence of synonymies that may affect estimates of lineage richness and specificity in this area. A standardization of the methods used in different laboratories from different regions may significantly improve the quality of the data available in MalAvi, which may benefit researchers globally.

## Concluding remarks

The utility of the MalAvi database of avian haemosporidians is proved beyond any doubt, since it is broadly used in ecological and parasitological literature and, essentially facilitates the work of researchers on this topic. However, based on the results reported here, it is highly recommended that MalAvi users be alerted to perform a comprehensive data cleaning and lineage homologation before doing any sequence analysis. If possible, authors should avoid using partial sequences of the parasite barcoding region and use the complete MalAvi region as the minimum differentiation unit. This is especially the case in areas such as South America, where synonymies are more frequently found. Before data submission to MalAvi, researchers should ensure that at least sequences cover the 479 bp fragment. Lineages should only be named based on BLAST hits to complete 479 bp records if available and follow the standard nomenclature procedures [10]. Partial sequences, which may introduce potential synonymies, should only be used when complete records are unavailable.

To avoid biases in the analyses of richness, diversity, and specificity of haemosporidians infecting birds based on lineage or lineage names already published in MalAvi, we recommend doing a blast against sequences deposited in both MalAvi and GenBank repositories and including them in an alignment to confirm that lineage names correspond to unique parasite sequences. Providing different names to sequences corresponding to the same genetic lineage may affect different estimations including the diversity of parasites circulating in a particular area or the host ranges of the parasites. To reduce the occurrence of these potential biases, approaches similar to those used by Gil-Vargas and Sedano [14] can be used. In this study, authors identified haplotypes by clustering sequences with a similarity ≥99.3% identity. In addition, Outlaw and Ricklefs [19], in a revision of species limits in avian haemosporidians, recommend a standardized procedure to “tag” these sequences, based on percentage sequence similarity. This approach may allow the inclusion of additional sequences from other databases, such as GenBank. However, this implies not using the barcode region of the parasite, increasing the difficulty of comparing the results with those from studies already conducted that define patterns of diversity, specificity, and distribution of avian haemosporidians. In this respect, different studies have shown that a higher hidden diversity may exist among described parasite lineages when other regions are considered [20], or no variation when mitochondrial genes are used [21]. Despite that, since very limited information about the genetic diversity of parasites is available in regions outside the barcoding region considered in MalAvi, nowadays most research focus on the use of this data set.

Furthermore, it is crucial to verify the length of the original sequence from the GenBank database [22]. GenBank allows verification of the lineage name and sequence length, due to the use of different primers to amplify the partial *cyt-b* gene, that does not fall within the barcode established in the MalAvi platform. By combining both platforms for blast comparison of parasite sequences, it is possible to minimize biases in lineage identification and use, as well as the use of longer sequences for phylogenetic and specificity analyses. Additionally, for those who are publishing their new sequences, we recommend always try to submit complete sequence barcodes to the public databases. In order to obtain the complete barcode sequence, both strands of the DNA (forward and reverse) should be sequenced, and the sequence quality should be checked to avoid losing information at the ends of them. Then, confirm the identity of the lineage for this genetic region before doing any formal analysis. Submitting only forward or reverse sequences is not good practice. Researchers should ensure that their lineage has not been previously reported before assigning a new name to it, contacting the curator of MalAvi database if necessary. Following a collaborative approach, the curation of this database may be easier and benefit the scientific community to develop further analyses on bird–vector–haemosporidian parasite interactions.

## Supporting information

S1 Supporting Information **Fig A.** Workflow of our study, from data collection to analysis. Data access June 2024. **Table A.** Lineages with synonymies in the open data platform MalAvi.
(DOCX)
