# Supplementary material for: Imprudent use of MalAvi names biases the estimation of parasite diversity of avian haemosporidians
Source: PLoS Pathog. 2025 Feb 5;21(2):e1012911. doi: 10.1371/journal.ppat.1012911 (PMC11798444; doi:10.1371/journal.ppat.1012911)
Supplement: S1 Supporting Information — Fig A. Workflow of our study, from data collection to analysis. Data access June 2024. Table A. Lineages with synonymies in the open data platform MalAvi. (DOCX) [file ppat.1012911.s001.docx]

**Imprudent use of MalAvi names biases the estimation of parasite diversity of avian haemoporidians**

**Juliana Tamayo-Quintero^1*^, Josué Martínez-de la Puente^2,3*^, Nubia E. Matta^4^, M. Andreína Pacheco^5^ and Héctor F. Rivera-Gutierrez^1^**

^1^ Grupo de Investigación de Ecología y Evolución de Vertebrados, Instituto de Biología, Universidad de Antioquia Medellín, Colombia

^2^  Estación Biológica de Doñana - CSIC, Sevilla, España

^3^ Ciber de Epidemiología y Salud Pública (CIBERESP), España

^4^ Departamento de Biología, Facultad de Ciencias, Universidad Nacional de Colombia, sede Bogotá, Colombia

^5^ Biology Department/Institute of Genomics and Evolutionary Medicine (iGEM), Temple University, Philadelphia, USA

* juliana.tamayoq@udea.edu.co, jmp@ebd.csic.es


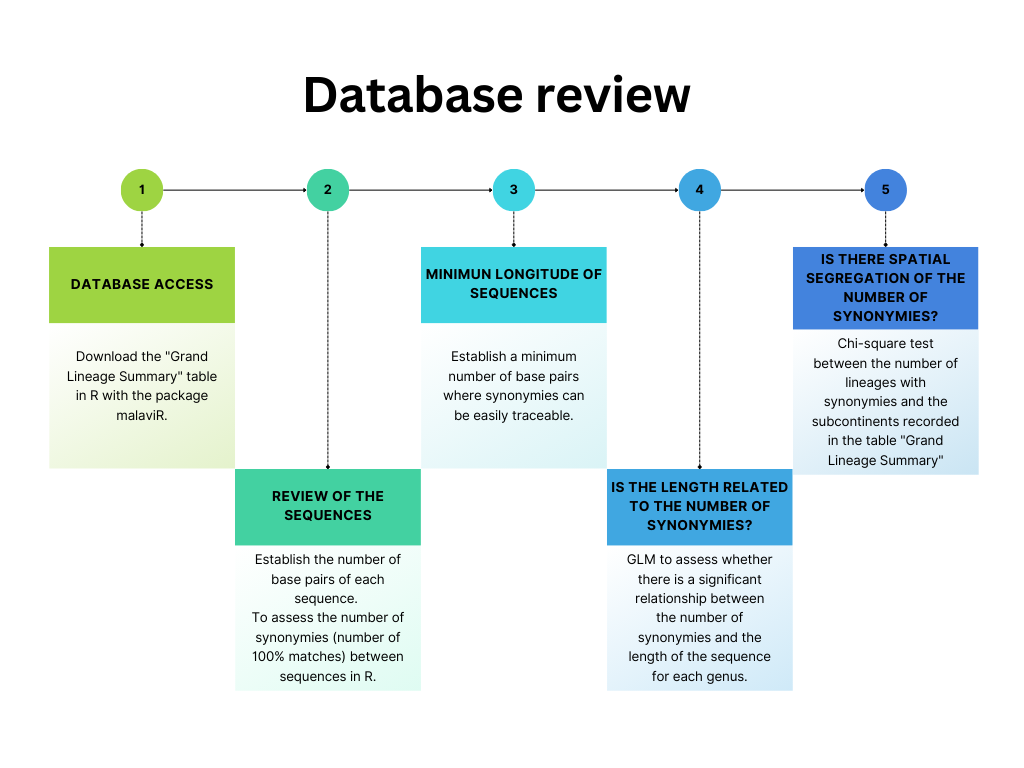


**Fig A.** Workflow of our study, from data collection to analysis. Data access June 2024.

**Table A.** Lineages with synonymies in the open data platform Malavi.

| **Genus** | **Lineage** | **Accession** | **Length** | **#Sinonyms** | **Lineage_sinonyms** | **Sequence in Malavi** | **Sequence lenght** | **Site** |
| --- | --- | --- | --- | --- | --- | --- | --- | --- |
| *Haemoproteus* | VIRFLA02 | AY817752 | Partial | 21 | TURGUL01, STANIG03, SETAUD22, SERUT05, SERUT04, SERUT02, SAXFUL01, PSIKRA01, PSAVIR01, PSADEC01, NEOBS04, NEOBS03, MIIGO01, MELGEO01, JUHYE01, HYPRO01, FIWES01, ERZAN02, ALMOR10, ALMOR07, ABSUP01 | TTTACTTATTTACATATTTTAAGAGGATTAAATTATTCATATTCATATTTACCTTTATCATGGATAACTGGACTAATAATATTTTTAATTTCTATTGTTACAGCTTTTATGGGTTATGTATTACCTTGGGGTCAAATGAGTTTCTGGGGTGCAACCGTTATTACTAATTTATTATATTTTATACCTGGACTTGTTTCATGGATTTGTGGAGGATATACTATTAGTGAT | 228 | - |
| *Haemoproteus* | VIOLI15 | AY817753 | Partial | 20 | TURGUL01, STANIG03, SERUT05, SERUT04, SERUT02, SAXFUL01, PSIKRA01, PSAVIR01, PSADEC01, NEOBS04, NEOBS03, MIIGO01, MELGEO01, JUHYE01, HYPRO01, FIWES01, ERZAN02, ALMOR10, ALMOR07, ABSUP01 | TTTACTTATTTACATATTTTAAGAGGATTAAATTATTCTTATTCATATTTACCTTTATCATGGATAACTGGATTATTAATATTCTTAATTTCTATTGTTACAGCTTTTATGGGTTATGTATTACCTTGGGGTCAAATGAGTTTCTGGGGTGCAACCGTTATTACTAATTTATTATATTTTATACCTGGACTTGTTTCATGGATTTGTGGAGGATATACTATTAGTGAT | 228 | - |
| *Haemoproteus* | VIRFLA03 | AY817754 | Partial | 20 | TURGUL01, STANIG03, SERUT05, SERUT04, SERUT02, SAXFUL01, PSIKRA01, PSAVIR01, PSADEC01, NEOBS04, NEOBS03, MIIGO01, MELGEO01, JUHYE01, HYPRO01, FIWES01, ERZAN02, ALMOR10, ALMOR07, ABSUP01 | TATTTACATATCTTAAGAGGATTAAATTATTCTTATTCATATTTACCTTTATCATGGATAACTGGATTAGTAATATTCTTAATTTCTATTGTTACAGCTTTTATGGGTTATGTATTACCTTGGGGTCAAATGAGTTTCTGGGGTGCAACCGTTATTACTAATTTATTATATTTTATACCTGGACTTGTTTCATGGATTTGTGGAGGATATACTATTAGTGAT | 222 | - |
| *Haemoproteus* | PSADEC01 | DQ241549 | Partial | 15 | ZOCAP17, ZOCAP15, VIRFLA03, VIRFLA02, VIOLI15, THRSAY01 , PIRLUD08, PIRFLA09, PIRFLA08, PHRFRU10, PHEMEL02, PADOM32, FISEM01, COEFLA01, ANISOM02 | GGTGCAACCGTTATTACTAATTTATTATATTTTATACCTGGACTTGTTTCATGGATTTGTGGAGGATATACTATTAGTGATCCAACTTTAAAAAGATTCTTTGTATTACATTTTATATTTCCTTTTATAGCTTTATGTATTGTATTTATACATATATTCTTCTTACACTTACAAGGTAGCTCTAATCCTTTAGGATATGATACAGCTTTAAAAATACCTTTCTATCCAAGTCTATTATGTCTAGATATCAAAGGATTTAATAATGTATTAGTCCTATTTCTAGCACAAAGTTTATTTGGAATTCT | 305 | South_America |
| *Haemoproteus* | MELGEO01 | AY640142 | Partial | 15 | VIRFLA03, VIRFLA02, VIOLI15, SPIPAS11, SPIARB01, SETAUD09, SETAUD02, ROFI3, QUIQUI01, PHRPLE01, PASILI01, DUNNO01, DIGHUM01, DENCOR01, CATANA01 | CCTGGACTTGTTTCATGGATTTGTGGAGGATATACTATTAGTGATCCAACTTTAAAAAGATTTTTTGTATTACATTTTATATTTCCTTTTATAGCTTTATGTATTGTATTCATACATATATTCTTCTTACACTTACAAGGTAGCTCTAATCCTTTAGGATATGATACAGCTTTAAAAATACCTTTCTATCCAAGTCTATTATGTCTAGATATCAAAGGATTTAATAATGTATTAGTCCTATTTCTAGCACAAAGTTTATTTGGAATTCT | 269 | North_America |
| *Plasmodium* | RBQ16 | EF117214 | Partial | 14 | ZOCAP09, SIAMEX02, SALMAX02, ROFI5, LEPCOR10, LBPIP1, HYPAM01, GBCAM1, COLL7, CINCHL02, CINCHL01, BSR2, ATLPIL02, ALEDIA01 | ATTCTTTGTATTACATTTTACATTTCCATTTATAGCTTTATGTATTGTATTTATACATATATTCTTTTTACATTTACAAGGTAGCACAAATCCTTTAGGGTATGATACAGCTTTAAAAATACCCTTCTATCCAAATCTTTTAAGTCTTGATATTAAAGGATTTAATAATGTATTAGTATTATTTTTAGCACAAAGTTTATTTGGAATATT | 210 | South_Sahara |
| *Haemoproteus* | SPIPAS11 | KJ910306 | Partial | 10 | TURGUL01, SERUT05, SERUT04, SERUT02, SAXFUL01, PSAVIR01, NEOBS04, NEOBS03, MELGEO01, JUHYE01, TURGUL01 | TTACTTACTTACATATATTAAGAGGATTAAACTACTCATATTCTTATTTACCTTTATCATGGATAACTGGATTAGTAATATTCTTAATCTCTATTGTTACCGCTTTTATGGGTTATGTATTACCTTGGGGTCAAATGAGTTTCTGGGGTGCCACCGTTATTACTAATTTATTATATTTTATACCTGGACTTGTTTCATGGATTTGTGGAGGATATACTATTAGTGAT | 227 | - |
| *Haemoproteus* | NECASP01 | AY714144 | Partial | 8 | CYAYNC01, CYASTE05, CYAMEL01, CYACRI01, CULCIR01, COBRA01, CIRCUM03, FANTAIL03 | TTGTGGTGGATATATAATTAGTGATCCAACTTTAAAAAGATTTTTTGTATTACATTTTATATTCCCATTTATAGCTTTATGTATTGTATTTATACATATATTCTTTTTACACTTACAAGGTAGCTCTAATCCTTTAGGATATGATACTGCTTTAAAAATACCTTTCTATCCAAGTCTATTATGTCTAGATATTAAAGGATTTAATAATGTATTAGTCTTATTTCTAGCACAAAGTTTATTTGGAATTTT | 249 | Asia, Oceania |
| *Plasmodium* | TABI07 | AY640128 | Partial | 8 | CXRES06, CERSER02, BAEBIC02, ZOCAP11, TROAED21, SEIAUR02, GEOTRI11, GEOTRI01 | TTTATTATATTTTATACCTGGTCTTGTTTCATGGATCTGTGGTGGATATCTTGTAAGCGACCCAACATTAAAAAGATTTTTTGTATTACATTTTATATTTCCATTTATAGCCTTATGTATTGTATTTATACATATATTCTTTCTACATTTACAAGGTAGCACAAATCCTTTAGGGTATGATACAGCTTTAAAAATACCCTTCTATCCAAATCTATTAAGTCTTGATATTAAAGGATTTAATAATATCTTAGTTTTATTTTTAGCACAAAGCTTATTTGGAATATT | 285 | North_America |
| *Haemoproteus* | STTRA01 | EF380177 | Partial | 8 | TYTAL4, TYTAL1, OTUSCO02, OTULEM01, NISALB02, BONUM01, AKGPH05, AKGPH02 | TTGTGGTGGATATAATATTAGTGATCCTACTTTAAAAAGATTCTTTGTATTACATTTTATATTTCCATTTGTAGCTTTATGTATTGTATTTATACATATATTCTTTTTACACTTACAAGGTAGCTCTAATCCTTTAGGATATGATACAGCTTTAAAAATACCTTTCTATCCAAGTCTATTATGTTTAGATATTAAAGGATTTAGTAATGTATTAGTATTATACTTAGCTCAAAGTTTATTTGGTATATT | 249 | Asia |
| *Plasmodium* | SEIAUR02 | EF011173 | Partial | 8 | ZOCAP12, TABI07, ICTCHR03, HYLLEU02, GEOTRI05, CINRUF01, BAEBIC02, AMMAUR01 | GCAACAGGTGCATCATTTGTATTTATTCTTACATATTTACATATTTTAAGAGGATTAAATTATTCTTATTCTTATTTACCTTTATCATGGATATCAGGATTAATAATATTTTTAATATCAATAGTTACTGCTTTTATGGGATATGTACTACCTTGGGGTCAAATGAGTTTCTGGGGTGCAACCGTCATTACTAATTTATTATATTTTATACCTGGTCTTGTTTCATGGATCTGTGGTGGATATCTTGTAAGCGACCCAACATTAAAAAGATTTTTTGTATTACATTTTATATTTCCATTTATAGCCTTATGTATTGTATTTATACATATATTCTTTCTACATTTACAAGGTAGCACAAATCCTTTAGG | 368 | North_America |
| *Haemoproteus* | JUHYE01 | AF465581 | Partial | 8 | VIRFLA03, VIRFLA02, VIOLI15, SPIPAS11, JUNPHA19, JUHYE03, ICTGUL03, GRMEL01 | GTTATTACTAATTTATTATATTTTATACCTGGACTTGTTTCATGGATTTGTGGAGGATATACTATTAGTGATCCAACTTTAAAAAGATTTTTTGTATTACATTTTATATTTCCTTTTATAGCTTTATGTATTGTATTTATACATATATTCTTCTTACACTTACAAGGTAGCTCTAATCCTTTAGGATATGATACAGCTTTAAAAATACCTTTCTATCCAAGTCTATTATGTCTAGATATCAAAGGATTTAATAATGTATTAGTCCTATTTCTAGCACAAAGTTTATTTGGTATTCT | 296 | North_America |
| *Plasmodium* | TABI08 | AY640130 | Partial | 7 | VERCEL03, SEIAUR01, MELMEL23, MELMEL04, MELMEL03, EUVIO01, BAEBIC01 | TTTATTATATTTTATACCTGGACTTGTTTCATGGATATGTGGTGGATATCTTGTAAGTGACCCAACCTTAAAAAGATTCTTTGTATTACATTTTACATTTCCATTTATAGCCTTATGTATTGTATTTATACATATATTCTTTTTACATTTACAAGGTAGCACAAATCCTTTAGGGTATGATACAGCTTTAAAAATACCCTTCTATCCAAATCTTTTAAGTCTTGATATTAAAGGATTTAATAATGTATTAGTATTATTCTTAGCACAAAGTTTATTTGGAATATT | 285 | North_America |
| *Haemoproteus* | SERUT05 | EF187473 | Partial | 7 | DENADE01, VIRFLA03, VIRFLA02, VIOLI15, SPIPAS11, SCLCAU03, LISP01 | TTTATTATATTTTATACCTGGACTTGTTTCATGGATTTGTGGAGGATATACTATTAGTGATCCAACTTTAAAAAGATTCTTTGTATTACATTTTATATTCCCTTTTATAGCTTTATGTATTGTATTTATTCATATATTCTTCTTACACTTACAAGGTAGCTCTAATCCTTTAGGATATGATACAGCTTTAAAAATACCTTTCTATCCAAGTCTATTATGTCTAGATATCAAAGGATTTAATAATGTATTAGTCCTATTTCTAGCACAAAGTTTATTTGGTATTCT | 285 | North_America |
| *Haemoproteus* | SERUT04 | EF187477 | Partial | 7 | VIRFLA03, VIRFLA02, VIOLI15, SPIPAS11, SETAUD05, SALMAX03, HABFUS01 | TTTATTATATTTTATACCTGGACTTGTTTCATGGATTTGTGGAGGATATACTATTAGTGATCCAACTTTAAAAAGATTCTTTGTATTACATTTTATATTTCCTTTTATAGCTTTATGTATTGTATTTATTCATATATTCTTCTTACACTTACAAGGTAGCTCTAATCCTTTAGGATATGATACAGCTTTAAAAATACCTTTCTATCCAAGTCTATTATGTCTAGATATCAAAGGATTTAATAATGTATTAGTCCTATTTCTAGCACAAAGTTTATTTGGTATTCT | 285 | North_America |
| *Haemoproteus* | TURGUL01 | EF380193 | Partial | 6 | VIRFLA03, VIRFLA02, VIOLI15, TURSTR02, SPIPAS11, ARGCAU1 | TTGTGGAGGATATACTATTAGTGATCCAACTTTAAAAAGATTTTTTGTATTACATTTTATATTTCCTTTTATAGCTTTATGTATTGTATTTATTCATATATTCTTTTTACACTTACAAGGTAGCTCTAATCCTTTAGGATATGATACAGCTTTAAAAATACCTTTCTATCCAAGTCTATTATGTCTAGATATCAAAGGATTTAATAATGTATTAGTTTTATTCCTAGCACAAAGTCTATTTGGAATTCT | 249 | Asia |
| *Haemoproteus* | COLPAS01 | DQ241558 | Partial | 5 | PEUCAS02, COLPAS09, COLPAS06, COLPAS05, COLPAS03 | GGTGCAACAGTTATTACTAATTTACTTTATTTTATACCTGGATTAGTCTCATGGATTTGTGGTGGATATATTGTTAGTGACCCTACCCTAAAAAGATTCTTTGTATTACATTTTATATTTCCTTTTATAGCTATATGTATAGTATTTATACATATATTCTTTCTACATTTACAAGGTAGCTCTAATCCTTTAGGATATGATACAGCTTTAAAAATACCCTTCTATCCAAGTCTATTATGCCTAGATATTAAAGGTTTTAATAACGTATTAGTATTATTCTTAGCTCAAAGCTTATTTGGAATATT | 305 | South_America |
| *Plasmodium* | TUMIG05 | EF011169 | Partial | 5 | CATUST05, CATFUS11, TUMIG22, DIGLAF01, DIGCYA02 | GCAACAGGTGCTTCATTTGTTTTCATTTTAACCTATTTACATATTTTAAGAGGATTAAATTATTCATATTCATATTTACCTTTATCATGGATTTCAGGATTATTAATATTTTTAATATCTATAGTAACAGCTTTTATGGGTTATGTATTACCTTGGGGTCAAATGAGTTTCTGGGGTGCTACTGTTATAACTAATTTATTATATTTTATACCTGGACTTGTCTCATGGATTTGTGGTGGATATCTTGTAAGTGACCCAACCTTAAAAAGATTTTTTGTATTACATTTTACATTCCCATTTATAGCTTTATGTATTGTATTTATACATATATTCTTCTTACATTTACAAGGTAGCACAAATCCTTTAGG | 368 | North_America |
| *Leucocytozoon* | SILUT01 | EF153660 | Full | 5 | SPIPAS07, PHEMEL01, MELLIN02, DENCOR06, CNEORN01 | TATCTACATATACTAAGAGGTTTAAATTACTCTTTCTCTTACTTACCTTTATCATGGATAAGTGGTTTAGTAATATTCTTAATATTTATTGTAACTGCTTTTATGGGTTATGTCTTACCATGGGGTCAAATGAGTTTCTGGGGAGCTACTGTAATTACTAACTTATTATATTTTATTCCTGGATTAATTAATTGGGTTTGTGGTGGTTTTATTATTAACGATCCAACTCTAAAAAGATTCTTTGTATTACATTTTATATTCCCATTCGTAGCTTTAGCTATTGTATTTATTCATATATTCTTCTTACATATTCAAGGTAGCACTAATCCATTAGGGTATGATACACCTTTAAAAATACCATTCTATCCAAATCTATTAACTTTAGATGTTAAAGGATTTAATTATGTATTAGTAATATTCTTATTTCAAAGTTTATTTGGTATTGC | 446 | South_America |
| *Haemoproteus* | ZONALB01 | EU254558 | Partial | 5 | ZONALB15, JUNPHA14, JUHYE13, GYMSAL02, GYMSAL01 | GCTACTGGAGCTACATTTGTATTTATTCTAACTTACTTACATATTTTAAGAGGATTAAATTATTCATATTCATATTTACCTTTATCATGGATTACTGGATTGGTAATATTTTTAATTTCTATTGTTACTGCTTTTATGGGTTATGTTTTACCTTGGGGTCAAATGAGTTTCTGGGGTGCAACCGTTATTACTAATTTATTATATTTTATACCTGGACTTGTTTCATGGATATGTGGTGGTTATACTATTAGTGATCCAACTCTAAAAAGATTTTTTGTATTACATTTTATATTTCCTTTTATAGCTTTATGCATCGTATTTATACATATATTCTTCTTACATTTACAAGGTAGCTCTAATC | 361 | North_America |
| *Haemoproteus* | NEOBS03 | DQ508394 | Partial | 5 | VIRFLA03, VIRFLA02, VIOLI15, SPIPAS11, CYAOLI03 | GAGGATATACTATTAGTGATCCAACTCTAAAAAGATTTTTTGTATTACATTTTATATTCCCTTTTATAGCTTTATGTATTGTATTTATACATATATTCTTTTTACACTTACAAGGTAGCTCTAATCCTTTAGGATATGATACAGCTTTAAAAATACCTTTCTATCCAAGTCTATTATGTCTAGATATTAAAGGATTTAATAATGTATTAGTCCTATTTCTAGCACAAAGTTTATTTGGAATTCT | 244 | South_Sahara |
| *Haemoproteus* | NEOBS04 | DQ508395 | Partial | 5 | CYAOLI05, VIRFLA03, VIRFLA02, VIOLI15, SPIPAS11 | GAGGATATACTATTAGTGATCCAACTCTAAAAAGATTCTTTGTATTACATTTTATATTTCCTTTTGTAGCTTTATGTATTGTATTTATACATATATTCTTTTTACACTTACAAGGTAGCTCTAATCCTTTAGGATATGATACAGCTTTAAAAATACCTTTCTATCCAAGTCTATTATGTCTAGATATTAAAGGATTTAATAATGTATTAGTCCTATTTCTAGCACAAAGTTTATTTGGTATTCT | 244 | South_Sahara |
| *Haemoproteus* | PSAVIR01 | DQ241540 | Partial | 5 | VIRFLA03, VIRFLA02, VIOLI15, SPIPAS11, PSABIF02 | TGTTTCATGGATTTGTGGAGGATATACTATTAGTGATCCAACTTTAAAAAGATTTTTTGTATTACATTTTATATTCCCTTTTATAGCTTTATGTATTGTATTTATACATATTTTCTTCTTACACTTACAAGGTAGCTCTAATCCTTTAGGATATGATACAGCTTTAAAAATACCTTTCTATCCAAGTCTATTATGTCTAGATATCAAAGGATTTAATAACATATTAGTCCTATTTCTAGCACAAAGTTTATTTGGAATTCT | 261 | South_America |
| *Haemoproteus* | SERUT02 | EF187479 | Partial | 5 | VIRHUT01, VIRFLA03, VIRFLA02, VIOLI15, SPIPAS11 | TTTATTATATTTTATACCTGGACTTGTTTCATGGATTTGTGGAGGATATACTATTAGTGATCCAACTTTAAAAAGATTCTTTGTATTACATTTTATATTTCCTTTTATAGCTTTATGTATTGTATTTATTCATATATTCTTCTTACACTTACAAGGTAGCTCTAATCCTTTAGGATATGATACAGCTTTAAAAATACCTTTCTATCCAAGTCTATTATGTCTAGATATCAAAGGATTTAATAATGTATTAGTCCTATTTCTAGCACAAAGTTTATTTGGAATTCT | 285 | North_America |
| *Leucocytozoon* | BUTLIN01 | EU254520 | Partial | 4 | BUTREG01, BUTJAM18, BUTJAM09, BUTJAM04 | TCTACCGGTGCCTCATTTGTATTTATTCTAACATATCTACATATACTAAAAGGATTAAATTATTCATACTCTTACTTACCATTGTCATGGATTACTGGAGTTATGATATTTCTAATATCTATAGTAACTGCCTTCTTAGGTTATGTATTACCATGGGGCCAAATGAGTTTCTGGGGTGCAACTGTAATTACTAATTTACTATATTTTATTCCTGGACTAATCTCATGGGTATGTGGTGGATATGCTGTAGGCGACCTAACCTTAAAAAGATTTTTTGTATTACACTTTATTTTCCCATTTGTAGCATTAGCTATTGTATTTATACATATATTCTTTCTACATTTACAAGGTAGCAGTAATC | 361 | North_America |
| *Plasmodium* | NEOLI01 | AF465551 | Partial | 4 | AEDTAE06, RECOB4, HEDCOL01, CYAOLI04 | ACCTGGTCTTGTTTCATGGATTTGTGGTGGATATCTTGTAAGTGATCCAACATTAAAAAGATTTTTTGTATTACATTTTATATTTCCATTTATAGCTTTATGTATTGTGTTTATACATATATTCTTTCTACATTTACAAGGTAGCACAAATCCTTTAGGATATGATACAGCTTTAAAAATACCCTTCTATCCAAATCTATTAAGTCTTGATATTAAAGGATTTAATAATATCTTAGTTTTATTTTTAGCACAAAGTTTATTTGGAATATT | 270 | South_Sahara |
| *Plasmodium* | VOLJAC01 | DQ241526 | Partial | 4 | TARUF01, NEOFAS06, GEOTRI08, BAHYP01 | TTGTGGTGGATATCTTGTAAGTGACCCAACTTTAAAAAGATTTTTCGTATTACATTTTACATTTCCATTTATAGCTTTATGTATTGTATTTATACATATATTCTTCTTACATTTACAAGGTAGCACAAATCCTTTAGGGTATGATACAGCTTTAAAAATACCCTTCTATCCAAATCTATTAAGTCTTGATATTAAAGGATTTAATAATGTATTAGTTTTATTCTTATCTCAAAGTTTATTTGGAATTTT | 249 | South_America |
| *Plasmodium* | BAEBIC01 | EF011183 | Partial | 4 | TABI08, POEATR02, BAERID01, BAEBIC04 | GCAACAGGTGCTTCATTTGTATTTATTTTAACTTATTTACATATTTTAAGAGGATTAAATTATTCATATTCATATTTACCTTTATCATGGATATCTGGACTAATTATATTTTTAATATCTATTGTAACAGCTTTTATGGGTTATGTATTACCTTGGGGTCAAATGAGTTTCTGGGGTGCTACAGTTATTACTAATTTATTATATTTTATACCTGGACTTGTTTCATGGATATGTGGTGGATATCTTGTAAGTGACCCAACCTTAAAAAGATTCTTTGTATTACATTTTACATTTCCATTTATAGCCTTATGTATTGTATTTATACATATATTCTTTTTACATTTACAAGGTAGCACAAATCCTTTAGG | 368 | North_America |
| *Haemoproteus* | VIGRI01 | AF465586 | Partial | 4 | VIRBEL02, VIGIL07, VIGIL06, LEIPER02 | TATTACTAATTTATTATATTTTATACCTGGACTTGTTTCATGGATTTGTGGAGGATATACTATAAGTGATCCAACTTTAAAAAGATTCTTTGTATTACATTTTATATTCCCTTTTATAGCTTTATGTATTGTATTTATACATATATTCTTCTTACATTTACAAGGTAGCTCTAATCCTTTAGGATATGATACAGCTTTAAAAATACCTTTCTATCCAAGTCTATTATGTCTAGATATTAAAGGATTTAATAATGTATTAGTCCTATTTCTAGCACAAAGTTTATTTGGAATTCT | 294 | North_America |
| *Plasmodium* | ZOSBRU01 | KT376934 | Partial | 4 | TSUB01, GALLUS47, FALTIN05, BUBBUB03 | ATTTTTTGTATTACATTTTATATTCCCATTTATAGCCTTATGTATTGTATTTATACATATTTTCTTTTTACATTTACAAGGTAGCACAAATCCTTTAGGGTATGATACAGCTTTAAAAATACCCTTCTATCCAAATCTATTAAGTCTTGATATTAAAGGATTTAATAATGTATTAGTTTTATTTTTATCACAAAGCTTATTTGGAATATT | 210 | South_Sahara |
| *Haemoproteus* | CATUST02 | DQ490060 | Partial | 4 | MONCAC01, CHAFA01, CATUST22, ADEMEL01 | TTTTATACCTGGACTTGTTTCATGGATTTGTGGTGGATATATTATTAGTGATCCAACTTTAAAAAGATTTTTTGTATTACATTTTATATTTCCATTTATAGCTTTATGTATTGTATTTATACATATATTCTTTTTACACTTACAAGGTAGCTCTAATCCTTTAGGATATGATACTGCTTTAAAAATACCTTTCTATCCAAGTCTATTATGTCTAGATATTAAAGGATTTAATAATGTATTAGTCTTATTTCTAGCACAAAGTTTATTTGGAATATT | 276 | North_America |
| *Haemoproteus* | SAXFUL01 | EF380195 | Partial | 4 | VIRFLA03, VIRFLA02, VIOLI15, SPIPAS11 | TTGTGGAGGATATACTATTAGTGATCCAACTCTAAAAAGATTTTTTGTATTACATTTTATATTTCCTTTTATAGCTTTATGTATTGTATTTATACATATATTCTTTCTACATTTACAAGGTAGCTCTAATCCTTTAGGATATGATACAGCTTTAAAAATACCTTTCTATCCAAGTCTATTATGTCTAGATATTAAAGGATTTAATAATGTATTAGTCCTATTTCTAGCACAAAGTTTATTTGGAATTCT | 249 | Asia |
| *Haemoproteus* | TABI05 | AY640151 | Partial | 3 | TABI02, SETAUD07, DENCOR03 | TTTATTATATTTTATACCTGGACTTGTTTCATGGATTTGTGGTGGATATACTATAAGTGATCCAACTCTAAAAAGATTTTTTGTATTACATTTTATATTTCCTTTTATAGCTTTATGCATCGTATTTATACATATATTCTTCTTACATTTACAAGGTAGCTCTAATCCTTTAGGATATGATACAGCTTTAAAAATACCTTTCTATCCAAGTCTATTATGTTTAGATATTAAAGGATTTAATAATGTATTAGTTATATTTTTAGCACAAAGTTTATTTGGTATTCT | 285 | North_America |
| *Plasmodium* | CXTHE01 | JF411408 | Full | 3 | DONANA03, CXPIP23, CXPIP21 | ATCTTAACTTACTTACACATTTTAAGAGGATTAAATTATTCATATTCATACTTACCTTTATCATGGATATCAGGATTAATGATATTTTTAATATCAATAGTTACAGCTTTTATGGGTTATGTATTACCTTGGGGTCAAATGAGTTTCTGGGGTGCAACTGTTATCACTAATTTATTATATTTTATCCCTGGACTTGTTTCATGGATTTGTGGTGGATATCTTGTAAGTGACCCAACTTTAAAAAGATTCTTTGTATTACATTTTACATTTCCATTTATAGCTTTATGTATTGTATTTATACATATATTCTTTCTACATTTACAAGGTAGCACTAATCCTTTAGGGTATGATACAGCTTTAAAAATACCCTTCTATCCAAATCTATTAAGTCTCGACATAAAAGGATTTAATAATGTATTAGTCTTAT | 427 | North_Africa_._Middle_East |
| *Haemoproteus* | DENCOR01 | EU254550 | Partial | 3 | PASILI01, MELGEO01, DENPEN02 | GCTACCGGTGCTACATTTGTTTTTATTTTAACTTACTTACATATATTAAGAGGATTAAACTACTCATATTCTTACTTACCTTTATCATGGATAACTGGATTAGTAATATTCTTAATCTCTATTGTTACCGCTTTTATGGGTTATGTATTACCTTGGGGTCAAATGAGTTTCTGGGGTGCAACCGTTATTACTAATTTATTATATTTTATACCTGGACTTGTTTCATGGATTTGTGGAGGATATACTATTAGTGATCCAACTTTAAAAAGATTTTTTGTATTACATTTTATATTTCCTTTTATAGCTTTATGTATTGTATTCATACATATATTCTTCTTACACTTACAAGGTAGCTCTAATC | 361 | North_America |
| *Leucocytozoon* | DENCOR06 | KF314798 | Full | 3 | CNEORN01, SILUT01, SETAUD25 | TCAACCGGTGCATCTTTTGTATTTATATTAACATATCTACATATACTAAGAGGTTTAAATTACTCTTTCTCTTACTTACCTTTATCATGGATAAGTGGTTTAGTAATATTCTTAATATTTATTGTAACTGCTTTTATGGGTTATGTCTTACCATGGGGTCAAATGAGTTTCTGGGGAGCTACTGTAATTACTAACTTATTATATTTTATTCCTGGATTAATTAATTGGGTTTGTGGTGGTTTTATTATTAACGATCCAACTCTAAAAAGATTCTTTGTATTACATTTTATATTCCCATTCGTAGCTTTAGCTATTGTATTTATTCATATATTCTTCTTACATATTCAAGGTAGCACTAATCCATTAGGGTATGATACACCTTTAAAAATACCATTCTATCCAAATCTATTAACTTTAGATGTTAAAGGATTTAAT | 435 | North_America |
| *Plasmodium* | NEOBS02 | DQ508392 | Partial | 3 | CYAOLI13, CYAOLI09, ELAALB03 | GAGGATATCTTGTAAGTGACCCAACTTTAAAAAGATTCTTTGTATTACATTTTATATTCCCATTTATAGCTTTATGTATTGTATTTATACATATATTCTTTTTACATCTACAAGGTAGCACAAATCCTTTAGGGTATGATACAGCTTTAAAAATACCCTTCTATCCAAATCTTTTAAGTCTTGATATAAAAGGATTTAATAATATATTAGTATTATTCTTATCACAAAGTTTATTTGGAATATT | 244 | South_Sahara |
| *Haemoproteus* | ADEMEL01 | KJ661259 | Full | 3 | CHAFA01, CATUST22, CATUST02 | CATTTGTCTTTATTTTAACTTATTTACATATATTAAGAGGATTAAATTATTCATATTCATATTTACCTTTATCATGGATATCTGGATTAATAATATTCTTAATTTCTATTGTTACTGCTTTTATGGGTTATGTATTACCTTGGGGTCAAATGAGTTTCTGGGGTGCAACCGTTATAACTAATTTATTATATTTTATACCTGGACTTGTTTCATGGATTTGTGGTGGATATATTATTAGTGATCCAACTTTAAAAAGATTTTTTGTATTACATTTTATATTTCCATTTATAGCTTTATGTATTGTATTTATACATATATTCTTTTTACACTTACAAGGTAGCTCTAATCCTTTAGGATATGATACTGCTTTAAAAATACCTTTCTATCCAAGTCTATTATGTCTAGATATTAAAGGATTTAATAATGTATTAGTCTTATTTCTAGCACAAAGTTTAT | 456 | South_America |
| *Haemoproteus* | CHAFA01 | EU254557 | Partial | 3 | ADEMEL01, CATUST22, CATUST02 | GCTACTGGTGCTACATTTGTCTTTATTTTAACTTATTTACATATATTAAGAGGATTAAATTATTCATATTCATATTTACCTTTATCATGGATATCTGGATTAATAATATTCTTAATTTCTATTGTTACTGCTTTTATGGGTTATGTATTACCTTGGGGTCAAATGAGTTTCTGGGGTGCAACCGTTATAACTAATTTATTATATTTTATACCTGGACTTGTTTCATGGATTTGTGGTGGATATATTATTAGTGATCCAACTTTAAAAAGATTTTTTGTATTACATTTTATATTTCCATTTATAGCTTTATGTATTGTATTTATACATATATTCTTTTTACACTTACAAGGTAGCTCTAATC | 361 | North_America |
| *Leucocytozoon* | HYPAM01 | AB183556 | Partial | 3 | RBQ16, RBQ15, HYPAM03 | TCAACAGGTGCATCTTTTGTATTTATCTTAACATATCTACATATCTTAAGAGGATTAAATTATTCTTTCTCTTACTTACCTTTATCATGGTATAGCGGTTTAATAATATTCTTAATCTTTATTGTAACTGCTTTTATGGGTTATGTTTTACCATGGGGACAAATGAGTTTCTGGGGAGCAACTGTAATTACTAACTTATTATATTTCATTCCTGGATTAATTAATTGGGTCTGTGGTGGATTTATTATTAATGACCCAACATTAAAAAGATTCTTTG | 277 | Asia |
| *Haemoproteus* | MIMPOL03 | MW081124 | Full | 3 | TOXCUR01, MIMPOL06, MIMPOL05 | TTTGTATTTATTTTAACTTATTTACATATATTAAGAGGATTAAATTATTCATATTCATATTTACCTTTATCATGGATATCTGGATTAATAATATTCTTAATTTCTATAGTTACTGCTTTTATGGGTTATGTATTACCTTGGGGTCAAATGAGTTTCTGGGGTGCAACCGTTATTACTAATTTATTATATTTTATACCTGGACTTGTTTCATGGATTTGTGGTGGATATATTATTAGTGATCCAACTTTAAAAAGATTCTTTGTATTACATTTTATATTCCCATTTATAGCTTTATGTATTGTATTTATACATATATTCTTTTTACACTTACAAGGTAGCTCTAATCCTTTAGGATATGATACTGCTTTAAAAATACCTTTCTATCCAAGTCTATTATGTCTAGATATTAAAGGATTTAATAATGTATTAGTCTTATTTCTAGCACAAAGTTTATTTGGAATAT | 463 | North_America |
| *Haemoproteus* | ABSUP01 | KJ145121 | Partial | 3 | VIRFLA03, VIRFLA02, VIOLI15 | TGGGGTCAAATGAGTTTCTGGGGTGCAACCGTTATTACTAATTTATTATATTTTATACCTGGACTTGTTTCATGGATTTGTGGAGGATATACTATTAGTGATCCAACTTTAAAAAGATTTTTTGTATTACATTTTATATTCCCTTTTATAGCTTTATGTATTGTATTTATACATATATTCTTCTTACACTTACAAGGTAGCTCTAATCCTTTAGGATATGATACAGCTTTAAAAATACCTTTCTATCCAAGTCTATTATGTCTAGATATTAAAGGATTTAATAATGTATTAGTCCTATTTCTAGCACAAAGTCTATTTGGAATTTT | 326 | Asia |
| *Haemoproteus* | ALMOR07 | KJ145114 | Partial | 3 | VIRFLA03, VIRFLA02, VIOLI15 | TGGGGTCAAATGAGTTTCTGGGGTGCAACCGTTATTACTAATTTATTATATTTTATACCTGGACTTGTTTCATGGATTTGTGGAGGATATACTATTAGTGATCCAACTTTAAAAAGATTTTTTGTATTACATTTTATATTCCCTTTTATAGCCCTATGTATTGTATTTATACATATATTTTTCTTACACTTACAAGGTAGCTCTAATCCTTTAGGATATGATACAGCTTTAAAAATACCTTTCTATCCAAGTCTATTATGTCTAGATATAAAAGGATTTAATAATGTATTAGTCCTATTTCTAGCACAAAGTTTATTTGGAATTTT | 326 | Asia |
| *Haemoproteus* | ALMOR10 | KJ145124 | Partial | 3 | VIRFLA03, VIRFLA02, VIOLI15 | TGGGGTCAAATGAGTTTCTGGGGTGCAACCGTTATTACTAATTTATTATATTTTATACCTGGACTTGTTTCATGGATTTGTGGAGGATATACTATTAGTGATCCAACTTTAAAAAGATTTTTTGTATTACATTTTATATTCCCTTTTATAGCTTTATGTATTGTATTTATACATATATTCTTTTTACACTTACAAGGTAGCTCTAATCCTTTAGGATATGATACAGCTTTAAAAATACCTTTCTATCCAAGTCTATTATGTCTAGATATTAAAGGATTTAATAATGTATTAGTCTTATTTCTAGCACAAAGTTTATTTGGAATATT | 326 | Asia |
| *Haemoproteus* | CATUST22 | KJ584600 | Full | 3 | ADEMEL01, CHAFA01, CATUST02 | CTACTGGTGCTACATTTGTCTTTATTTTAACTTATTTACATATATTAAGAGGATTAAATTATTCATATTCATATTTACCTTTATCATGGATATCTGGATTAATAATATTCTTAATTTCTATTGTTACTGCTTTTATGGGTTATGTATTACCTTGGGGTCAAATGAGTTTCTGGGGTGCAACCGTTATAACTAATTTATTATATTTTATACCTGGACTTGTTTCATGGATTTGTGGTGGATATATTATTAGTGATCCAACTTTAAAAAGATTTTTTGTATTACATTTTATATTTCCATTTATAGCTTTATGTATTGTATTTATACATATATTCTTTTTACACTTACAAGGTAGCTCTAATCCTTTAGGATATGATACTGCTTTAAAAATACCTTTCTATCCAAGTCTATTATGTCTAGATATTAAAGGATTTAATAATGTATTAGTCTTATTTCTAGCACAAAGTTTATTTGGAATATT | 478 | North_America |
| *Haemoproteus* | ERZAN02 | KJ145105 | Partial | 3 | VIRFLA03, VIRFLA02, VIOLI15 | TGGGGTCAAATGAGTTTCTGGGGTGCAACCGTTATTACTAATTTATTATATTTTATACCTGGACTTGTTTCATGGATTTGTGGAGGATATACTATTAGTGATCCAACTTTAAAAAGATTTTTTGTATTACATTTCATATTTCCTTTTATAGCTCTATGTATTGTATTTATACATATATTCTTCTTACATTTACAAGGTAGCTCTAATCCTTTAGGATATGATACAGCTTTAAAAATACCTTTCTATCCAAGTCTATTATGTTTAGATATTAAAGGATTTAATAATGTATTAGTCCTATTTCTAGCACAAAGTTTATTTGGAATTTT | 326 | Asia |
| *Haemoproteus* | FIWES01 | KJ145112 | Partial | 3 | VIRFLA03, VIRFLA02, VIOLI15 | TGGGGTCAAATGAGTTTCTGGGGTGCAACCGTTATTACTAATTTATTATATTTTATACCTGGACTTGTTTCATGGATTTGTGGAGGATATACTATTAGTGATCCAACTTTAAAAAGATTTTTTGTATTACATTTTATATTTCCTTTTATAGCTTTATGTATTGTATTTATTCATATATTCTTTTTACATTTACAAGGTAGCTCTAATCCTTTAGGATATGATACAGCTTTAAAAATACCTTTCTATCCAAGTCTATTATGTCTAGATATTAAAGGATTTAATAATGTATTAGTCCTATTTTTAGCACAAAGTTTATTTGGTATACT | 326 | Asia |
| *Haemoproteus* | HYPRO01 | KJ145125 | Partial | 3 | VIRFLA03, VIRFLA02, VIOLI15 | TGGGGTCAAATGAGTTTCTGGGGTGCAACCGTTATTACTAATTTATTATATTTTATACCTGGACTTGTTTCATGGATTTGTGGAGGATATACTATTAGTGATCCAACTTTAAAAAGATTTTTTGTATTACATTTTATATTCCCTTTTATAGCTTTATGTATTGTATTTATACATATATTCTTTTTACACTTACAAGGTAGCTCTAATCCTTTAGGATATGATACAGCTTTAAAAATACCTTTCTATCCAAGTCTATTATGTCTAGATATTAAAGGATTTAATAATGTATTAGTCCTATTTCTAGCACAAAGTTTATTTGGAATTTT | 326 | Asia |
| *Haemoproteus* | MIIGO01 | KJ145118 | Partial | 3 | VIRFLA03, VIRFLA02, VIOLI15 | TGGGGTCAAATGAGTTTCTGGGGTGCAACCGTTATTACTAATTTATTATATTTTATACCTGGACTTGTTTCATGGATTTGTGGAGGATATACTATTAGTGATCCAACTTTAAAAAGATTTTTTGTATTACATTTTATATTTCCTTTTATAGCTTTATGTATTGTATTTATTCATATATTCTTTTTACATTTACAAGGTAGCTCTAATCCTTTAGGATATGATACAGCTTTAAAAATACCTTTCTATCCAAGTCTATTATGTCTAGATATTAAAGGATTTAATAATGTATTAGTCCTATTTCTAGCACAAAGTTTATTTGGAATACT | 326 | Asia |
| *Haemoproteus* | PSIKRA01 | EF380207 | Partial | 3 | VIRFLA03, VIRFLA02, VIOLI15 | GGTGCAACCGTTATTACTAATTTATTATATTTTATACCTGGACTTGTTTCATGGATTTGTGGAGGATATACTATTAGTGATCCAACTTTAAAAAGATTCTTTGTATTACATTTTATATTCCCATTTATAGCTTTATGTATTGTATTTATACATATATTTTTCTTACACTTACAAGGTAGCTCTAATCCTTTAGGATATGATACAGCTTTAAAAATACCTTTCTATCCAAGTCTATTATGTCTAGATATTAAAGGATTTAATAATATATTAGTCCTATTTCTAGCACAAAGTTTATTTGGAATATT | 305 | Asia |
| *Haemoproteus* | STANIG03 | KJ145122 | Partial | 3 | VIRFLA03, VIRFLA02, VIOLI15 | TGGGGTCAAATGAGTTTCTGGGGTGCAACCGTTATTACTAATTTATTATATTTTATACCTGGACTTGTTTCATGGATTTGTGGAGGATATACTATTAGTGATCCAACTTTAAAAAGATTTTTTGTATTACATTTTATATTCCCTTTTATAGCTTTATGTATTGTATTTATTCATATATTCTTTTTACACTTACAAGGTAGCTCTAATCCTTTAGGATATGATACAGCTTTAAAAATACCTTTCTATCCAAGTCTATTATGTCTAGATATTAAAGGATTTAATAATGTATTAGTCCTATTTCTAGCACAAAGTTTATTTGGAATTTT | 326 | Asia |
| *Haemoproteus* | DENPET01 | AY640129 | Partial | 2 | SIAMEX01, POEATR07 | TTTATTATATTTTATACCTGGACTTGTTTCATGGATTTGTGGTGGATATACTATAAGTGATCCAACTCTAAAAAGATTTTTTGTATTACATTTTATATTTCCTTTTATAGCTTTATGCATCGTATTTATACATATATTCTTCTTACATTTACAAGGTAGCTCTAATCCTTTAGGATATGATACAGCTTTAAAAATACCTTTCTATCCAAGTCTATTATGTTTAGATATTAAAGGATTTAATAATGTATTAGTTATATTTTTAGCACAAAGTTTATTTGGTATTTT | 285 | North_America |
| *Leucocytozoon* | ACCBRE01 | EU254519 | Partial | 2 | ACCBRE03, ACCBRE02 | TCTACAGGTGCATCATTTGTATTTATTCTAACATACTTACATATATTAAAAGGATTGAATTATTCATATTCATATTTACCATTATCATGGATTACTGGAATTGTAATATTTCTAATATCTATAGTAACCGCTTTCTTAGGTTATGTTTTACCATGGGGTCAAATGAGTTTCTGGGGTGCTACCGTAATTACTAATTTATTATATTTTATTCCTGGACTAATTTCATGGGTCTGTGGTGGATATGCTGTAGGCGATCTTACTCTAAAAAGATTCTTTGTATTACACTTTATTTTTCCATTTGTAGCTTTAGCTATTGTATTTATTCATATATTCTTTCTACATTTACAAGGTAGCAGTAATC | 361 | North_Africa_._Middle_East |
| *Haemoproteus* | APSPI01 | EF153652 | Full | 2 | ZONCAP01, ZOCAP08 | ACTTACTTACATATATTAAGAGGATTAAATTATTCATATTCTTATTTACCTTTATCATGGATAACTGGACTAATAATATTCTTAATTTCTATTGTTACCGCTTTTATGGGTTATGTATTACCTTGGGGACAAATGAGTTTCTGGGGTGCAACCGTTATTACTAATTTATTATATTTTATACCTGGACTTGTTTCATGGATTTGTGGAGGATATACTATTAGTGATCCAACTTTAAAAAGATTTTTTGTACTACATTTTATATTTCCTTTTATAGCTTTATGTATTGTATTTATACATATATTCTTCTTACACTTACAAGGTAGCTCTAATCCTTTAGGATATGATACAGCTTTAAAAATACCTTTCTATCCAAGTCTATTATGTCTAGATATCAAAGGATTTAATAATGTATTAGTCCTATTTCTAGCACAAAGTTTATTTGGAATTCT | 449 | South_America |
| *Leucocytozoon* | CATUST11 | JN792150 | Full | 2 | PHYBOR02, CATMIN05 | TATCTACATATCTTAAGAGGTTTAAATTATTCATTCTCTTATTTACCTTTATCATGGTATACAGGTTTAATAATATTCTTAATATTCATTGTAACTGCTTTTATGGGTTACGTATTACCATGGGGACAAATGAGTTTCTGGGGAGCAACTGTTATTACTAATTTATTATATTTTATTCCTGGATTAATCAATTGGGTATGTGGTGGATTTATTATTAATGATCCAACCCTAAAAAGATTCTTCGTATTACATTTCATATTCCCATTTGTAGCTTTAGCTATTGTATTTATTCATATATTCTTCTTACATATTCATGGTAGCACTAATCCTTTAGGGTATGATACACCTCTAAAAATACCATTCTATCCAAATCTATTAACTTTAGATATTAAAGGATTTAACTATGTATTAGTTATATTCTTATTCCAAAGTTTATTTGGAAT | 443 | North_America, Central_America, South_America |
| *Leucocytozoon* | COLBF09 | KR052941 | Full | 2 | COLBF24, COLBF01 | CAACTGGTGCATCTTTTGTCTTTATATTAACATATCTACATATATTAAGAGGACTAAACTATTCTTACTCATACCTACCTCTATCATGGATCACAGGTTTAATTATATTCTTAATATCCATAATGACAGCCTTTATGGGTTATGTATTACCATGGGGACAAATGAGTTATTGGGGAGCAACTGTAATTACCAATCTATTATACTTTATTCCTGGATTAATCTCATGGGTCTGTGGAGGATTCGTGGTTAATGATCCAACTCTAAAAAGATTCTTTGTACTTCATTTTATTTTCCCATTTGTAGCATTAATTATAGTATTTATTCACATATTCTACTTACATCTACAAGGTAGCACTAATCCTTTAGGATATGATACAGCTCTAAAAATACCCTTCTATCCAAATCTTTTATGTCTAGATATTAAAGGATTTGCAAATATACTAGTATTATTCCTAGCACAAAGTTTATTTGG | 472 | North_America |
| *Plasmodium* | LARINC02 | EU254547 | Partial | 2 | WW3, GEOTRI06 | GCAACAGGTGCTTCATTTGTTTTCATTTTAACCTATTTACATATTTTAAGAGGATTAAATTACTCATATTCATATTTACCTTTATCATGGATTTCAGGATTATTAATATTTTTAATATCCATAGTTACTGCTTTTATGGGTTATGTATTACCTTGGGGTCAAATGAGTTTCTGGGGTGCTACAGTTATAACTAACTTATTATATTTTATACCTGGACTTGTCTCATGGATTTGTGGTGGATATCTTGTAAGTGACCCAACCTTAAAAAGATTTTTTGTATTACATTTTACATTCCCATTTATAGCTTTATGTATTGTATTCATACATATATTCTTCTTACATTTACAAGGTAGCACAAATC | 361 | North_America |
| *Plasmodium* | PARVEN02 | KT757573 | Full | 2 | TARCYA01, POEMON03 | TCTACAGGTGCATCTTTTGTATTTATCTTAACATATCTACACATCTTAAGAGGACTAAACTATTCATTCTCTTACTTACCTTTATCATGGTATAGTGGTTTAGTTATATTCTTAATCTTTATTGTAACTGCTTTTATGGGTTACGTTTTACCATGGGGACAAATGAGTTTCTGGGGAGCAACTGTAATTACTAATTTATTATATTTTATTCCTGGATTAATTAATTGGGTCTGTGGTGGATTCATTATTAATGACCCAACATTAAAAAGATTCTTCGTCTTACATTTTATATTTCCATTTGTAGCCTTAGCTATTGTATTTATTCATATATTCTTCTTACATATTCATGGTAGCACTAATCCTTTAGGGTATGATACACCTTTAAAAATACCATTCTATCCAAATCTATTAACTTTAGATATTAAAGG | 428 | Asia |
| *Leucocytozoon* | PERATE08 |  | Full | 2 | PERATE09, PERATE07 | TCTACATATTTTAAGAGGATTAAATTATTCTTTCTCTTACTTACCTTTATCATGGTATAGTGGTTTAATTATATTCTTAATCTTTATTGTAACTGCTTTTATGGGTTACGTTCTACCATGGGGACAAATGAGTTTCTGGGGAGCAACTGTAATTACTAACTTATTATATTTCATTCCTGGATTAATTAATTGGGTCTGTGGTGGATTTATTATTAATGACCCAACACTAAAAAGATTCTTTGTATTACATTTTATATTCCCATTTGTAGCCTTAGCTATTGTATTTATTCATATATTCTTCTTACATATTCATGGTAGCACTAATCCTTTAGGGTATGATACACCTTTAAAAATACCATTCTATCCAAATCTATTAACTTTAGATGTTAAAGCATTTAATTATGTATTAGTTATATTTTTATTTCAAAGTTTATTTGGAATTGC | 444 | Asia |
| *Plasmodium* | SALCOE01 | DQ241532 | Partial | 2 | THRPAL01, RHYSIM01 | GGAGCAACTGTAATTACCAATTTATTATATTTTATTCCAGGACTTGTATCATGGATTTGTGGTGGATATCTTGTTAGTGACCCAACACTAAAAAGATTTTTTGTATTACATTTTACATTTCCATTTATAGCTTTATGTATTGTATTTATACATATATTCTTTTTACATTTACAAGGTAGCACTAATCCTTTAGGGTATGATACAGCTTTAAAAATACCCTTCTATCCAAATCTATTAAGTCTCGATATTAAAGGATTTAATAATATATTAGTACTATTTTTAGCACAAAGTTTATTTGGAATCTT | 305 | South_America |
| *Leucocytozoon* | TURMER11 | MZ571112 | Full | 2 | TURMER13, TURMER12 | CAACAGGTGCATCTTTTGTATTTATATTAACATATTTACACATATTAAGAGGATTAAATTATTCATTCTCTTATTTACCACTATCATGGTTAAGTGGTTTAATATTATTTATGATATTTATTGTAACTGCTTTTATGGGTTATGTATTACCATGGGGACAAATGAGTTTCTGGGGAGCAACTGTTATTACTAATTTATTATATTTTATTCCTGGATTAATTAATTGGGTTTGTGGTGGATTTATTATTAACGATCCAACATTAAAAAGATTCTTTGTATTACATTTTATATTCCCATTTATAGCATTAGTTATTGTATTTGTACATATATTTTTCTTACATCTTCAAGGTAGCTCAAATCCTTTAGGGTATAATACCCCATTAAAAATACCATTCTATCCAAATCTATTAACTCTAGATGTTAAAGGATTTAATTATGTATTAGTATTATTCCTATTTCAAAGTTTATTTGGAATC | 476 | Europe |
| *Leucocytozoon* | TURMER12 | MZ571117 | Full | 2 | TURMER13, TURMER11 | CAACAGGTGCATCTTTTGTATTTATATTAACATATTTACACATATTAAGAGGATTAAATTATTCATTCTCTTATTTACCACTATCATGGTTAAGTGGTTTAATATTATTTATGATATTTATTGTAACTGCTTTTATGGGTTATGTATTACCATGGGGACAAATGAGTTTCTGGGGAGCAACTGTTATTACTAATTTATTATATTTTATTCCTGGATTAATTAATTGGGTTTGTGGTGGATTTATTATTAACGATCCAACATTAAAAAGATTCTTTGTATTACATTTTATATTCCCATTTATAGCATTAGTTATTGTATTTGTACATATATTTTTCTTACATCTTCAAGGTAGCTCAAATCCTTTAGGGTATAATACCCCATTAAAAATACCATTCTATCCAAATCTATTAACTCTAGATGTTAAAGGATTTAATTATGTATTAGTATTATTCCTATTTCAAAGTTTATTTGGAATC | 476 | Europe |
| *Leucocytozoon* | TURMER13 | MZ571118 | Full | 2 | TURMER12, TURMER11 | CAACAGGTGCATCTTTTGTATTTATATTAACATATTTACACATATTAAGAGGATTAAATTATTCATTCTCTTATTTACCACTATCATGGTTAAGTGGTTTAATATTATTTATGATATTTATTGTAACTGCTTTTATGGGTTATGTATTACCATGGGGACAAATGAGTTTCTGGGGAGCAACTGTTATTACTAATTTATTATATTTTATTCCTGGATTAATTAATTGGGTTTGTGGTGGATTTATTATTAACGATCCAACATTAAAAAGATTCTTTGTATTACATTTTATATTCCCATTTATAGCATTAGTTATTGTATTTGTACATATATTTTTCTTACATCTTCAAGGTAGCTCAAATCCTTTAGGGTATAATACCCCATTAAAAATACCATTCTATCCAAATCTATTAACTCTAGATGTTAAAGGATTTAATTATGTATTAGTATTATTCCTATTTCAAAGTTTATTTGGAATC | 476 | Europe |
| *Haemoproteus* | ZEGAL04 | FJ462673 | Partial | 2 | COLTAL01, ZEGAL05 | GCCACAGGTGCATCATTTGTATTTATTTTAACATACCTACATATTTTAAGAGGATTAAATTACTCATATTCATATTTACCATTATCATGGATTACCGGATTAATAATATTTATAATCTCTATAATGACTGCTTTCTTGGGTTATGTTCTACCTTGGGGTCAAATGAGTTTCTGGGGTGCAACTGTTATTACTAATTTACTATATTTTATTCCGGGATTAGTATCATGGATTTGTGGTGGTTATATAGTTAGTGATCCTACACTAAAAAGATTCTTTGTATTACATTTTATATTTCCATTTATAGCTATATGTATAGTATTTATACATATATTCTTTTTACATTTACA | 347 | South_America |
| *Leucocytozoon* | ZOCAP05 | EF153657 | Partial | 2 | CARBAR02, CARBAR01 | TAAACTATTCATTCTCTTACTTACCTTTATCATGGTATAGTGGTTTAGTTATATTCTTAATCTTTATTGTAACTGCTTTTATGGGTTACGTTTTACCATGGGGACAAATGAGTTTCTGGGGAGCAACTGTAATTACTAATTTATTATATTTTATTCCTGGATTAATTAATTGGGTCTGTGGTGGATTCATAATTAATGACCCAACATTAAAAAGATTCTTCGTATTACACTTTATATTCCCATTTATAGCCTTAGCTATTGTATTTATTCATATATTCTTCTTACATATTCATGGTAGCACTAATCCTTTAGGGTATGATACACCTTTAAAAATACCATTCTATCCAAATCTATTAACTTTAGATATTAAAGGATTTAACTATGTATTAGTTATATTTTTATTTCAAAGTTTATTTGGAATTGC | 424 | South_America |
| *Plasmodium* | CINCHL02 | EU810663 | Partial | 2 | RBQ16, CINCHL01 | GGTGCTACCGTAATAACTAATTTACTATATTTTATACCTGGACTTGTTTCATGGATATGTGGTGGATATCTTGTAAGTGACCCAACTTTAAAAAGATTCTTTGTATTACATTTTACATTTCCATTTATAGCTTTATGTATTGTATTTATACATATATTCTTTTTACATTTACAAGGTAGCACAAATCCTTTAGGGTATGATACAGCTTTAAAAATACCCTTCTATCCAAATCTTTTAAGTCTTGATATTAAAGGATTTAATAATGTATTAGTATTATTTTTAGCACAAAGTTTATTTGGAATATT | 305 | South_Sahara |
| *Plasmodium* | CINPRE02 | KJ446984 | Partial | 2 | DELFRA04, CYAOLI10 | TTTATTTTAACTTATTTACATATTTTAAGAGGATTAAATTACTCATACTCCTACTTACCTTTATCATGGATGTCAGGGTTAATAATATTTCTAATATCTATAGTAACAGCTTTTATGGGTTATGTATTACCATGGGGTCAAATGAGTTTCTGGGGTGCAACTGTAATTACTAATTTATTATATTTTATACCTGGACTTGTTTCATGGATATGCGGAGGATATCTTGTAAGTGACCCAACTTTAAAAAGATTCTTTGTATTACATTTTATATTCCCATTTGTAGCCTTATGTATTGTATTTATACATATATTCTTCTTACATTTACAAGGTAGCACAAATC | 340 | South_Sahara |
| *Plasmodium* | CYAOLI10 | FJ404717 | Partial | 2 | DELFRA04, CINPRE02 | TTTTATACCTGGACTTGTTTCATGGATATGCGGAGGATATCTTGTAAGTGACCCAACTTTAAAAAGATTCTTTGTATTACATTTTATATTCCCATTTGTAGCCTTATGTATTGTATTTATACATATATTCTTCTTACATTTACAAGGTAGCACAAATCCTTTAGGGTATGATACAGCTTTAAAAATACCCTTCTATCCAAATCTTTTAAGTCTTGATATAAAAGGATTTAACAATATATTAGTATTATTTTTATCACAAAGTTTATTTGGAATATT | 276 | South_Sahara |
| *Plasmodium* | DIGCYA02 | KJ661266 | Full | 2 | TUMIG05, DIGLAF01 | CATTTGTTTTCATTTTAACCTATTTACATATTTTAAGAGGATTAAATTATTCATATTCATATTTACCTTTATCATGGATTTCAGGATTATTAATATTTTTAATATCTATAGTAACAGCTTTTATGGGTTATGTATTACCTTGGGGTCAAATGAGTTTCTGGGGTGCTACTGTTATAACTAATTTATTATATTTTATACCTGGACTTGTCTCATGGATTTGTGGTGGATATCTTGTAAGTGACCCAACCTTAAAAAGATTTTTTGTATTACATTTTACATTCCCATTTATAGCTTTATGTATTGTATTTATACATATATTCTTCTTACATTTACAAGGTAGCACAAATCCTTTAGGGTATGATACAGCTTTAAAAATACCCTTCTATCCAAATCTATTAAGTCTTGATATTAAAGGATTTAATAATGTATTAGTTTTATTCCTATCTCAAAGTTTAT | 456 | South_America |
| *Leucocytozoon* | PERATE07 |  | Full | 2 | PERATE09, PERATE08 | TCAACAGGTGCATCTTTTGTATTTATATTAACATATCTACATATTTTAAGAGGATTAAATTATTCTTTCTCTTACTTACCTTTATCATGGTATAGTGGTTTAATTATATTCTTAATCTTTATTGTAACTGCTTTTATGGGTTACGTTCTACCATGGGGACAAATGAGTTTCTGGGGAGCAACTGTAATTACTAACTTATTATATTTCATTCCTGGATTAATTAATTGGGTCTGTGGTGGATTTATTATTAATGACCCAACACTAAAAAGATTCTTTGTATTACATTTTATATTCCCATTTGTAGCCTTAGCTATTGTATTTATTCATATATTCTTCTTACATATTCATGGTAGCACTAATCCTTTAGGGTATGATACACCTTTAAAAATACCATTCTATCCAAATCTATTAACTTTAGATGTTAAAGCATTTAATTATGTATTAGTTATATTTTTATTTCAAAGTTTATTTGGAATTGC | 479 | Asia |
| *Leucocytozoon* | PERATE09 |  | Full | 2 | PERATE08, PERATE07 | TCAACAGGTGCATCTTTTGTATTTATATTAACATATCTACATATTTTAAGAGGATTAAATTATTCTTTCTCTTACTTACCTTTATCATGGTATAGTGGTTTAATTATATTCTTAATCTTTATTGTAACTGCTTTTATGGGTTACGTTCTACCATGGGGACAAATGAGTTTCTGGGGAGCAACTGTAATTACTAACTTATTATATTTCATTCCTGGATTAATTAATTGGGTCTGTGGTGGATTTATTATTAATGACCCAACACTAAAAAGATTCTTTGTATTACATTTTATATTCCCATTTGTAGCCTTAGCTATTGTATTTATTCATATATTCTTCTTACATATTCATGGTAGCACTAATCCTTTAGGGTATGATACACCTTTAAAAATACCATTCTATCCAAATCTATTAACTTTAGATGTTAAAGCATTTAATTATGTATTAGTTATATTTTTATTTCAAAGTTTATTTGGAATTGC | 479 | Asia |
| *Leucocytozoon* | PHEMEL01 | KJ584586 | Full | 2 | SPIPAS07, SILUT01 | CAACAGGTGCATCTTTTGTATTTATATTAACATATCTACATATACTAAGAGGTTTAAATTACTCTTTCTCTTACTTACCTTTATCATGGATAAGTGGTTTAGTAATATTCTTAATATTTATTGTAACTGCTTTTATGGGTTATGTCTTACCATGGGGTCAAATGAGTTTCTGGGGAGCTACTGTAATTACTAACTTATTATATTTTATTCCTGGATTAATTAATTGGGTTTGTGGTGGTTTTATTATTAACGATCCAACTCTAAAAAGATTCTTTGTATTACATTTTATATTCCCATTCGTAGCTTTAGCTATTGTATTTATTCATATATTCTTCTTACATATTCAAGGTAGCACTAATCCATTAGGGTATGATACACCTTTAAAAATACCATTCTATCCAAATCTATTAACTTTAGATGTTAAAGGATTTAATTATGTATTAGTAATATTCTTATTTCAAAGTTTATTTGGTAT | 475 | North_America |
| *Leucocytozoon* | POEMON03 |  | Full | 2 | TARCYA01, PARVEN02 | ACAGGTGCATCTTTTGTATTTATCTTAACATATCTACACATCTTAAGAGGACTAAACTATTCATTCTCTTACTTACCTTTATCATGGTATAGTGGTTTAGTTATATTCTTAATCTTTATTGTAACTGCTTTTATGGGTTACGTTTTACCATGGGGACAAATGAGTTTCTGGGGAGCAACTGTAATTACTAATTTATTATATTTTATTCCTGGATTAATTAATTGGGTCTGTGGTGGATTCATTATTAATGACCCAACATTAAAAAGATTCTTCGTCTTACATTTTATATTTCCATTTGTAGCCTTAGCTATTGTATTTATTCATATATTCTTCTTACATATTCATGGTAGCACTAATCCTTTAGGGTATGATACACCTTTAAAAATACCATTCTATCCAAATCTATTAACTTTAGATATTAAAGGATTTAACTATGTATTAGTTATATTTTTATTTCAAAGTTTATTTGGAATTGC | 476 | Asia |
| *Leucocytozoon* | SPIPAS07 | MF817795 | Full | 2 | SILUT01, PHEMEL01 | TCAACAGGTGCATCTTTTGTATTTATATTAACATATCTACATATACTAAGAGGTTTAAATTACTCTTTCTCTTACTTACCTTTATCATGGATAAGTGGTTTAGTAATATTCTTAATATTTATTGTAACTGCTTTTATGGGTTATGTCTTACCATGGGGTCAAATGAGTTTCTGGGGAGCTACTGTAATTACTAACTTATTATATTTTATTCCTGGATTAATTAATTGGGTTTGTGGTGGTTTTATTATTAACGATCCAACTCTAAAAAGATTCTTTGTATTACATTTTATATTCCCATTCGTAGCTTTAGCTATTGTATTTATTCATATATTCTTCTTACATATTCAAGGTAGCACTAATCCATTAGGGTATGATACACCTTTAAAAATACCATTCTATCCAAATCTATTAACTTTAGATGTTAAAGGATTTAATTATGTATTAGTAATATTCTTATTTCAAAGTTTATTTGGTATTGC | 479 | North_America, Central_America |
| *Leucocytozoon* | TARCYA01 |  | Full | 2 | PARVEN02, POEMON03 | TCTACAGGTGCATCTTTTGTATTTATCTTAACATATCTACACATCTTAAGAGGACTAAACTATTCATTCTCTTACTTACCTTTATCATGGTATAGTGGTTTAGTTATATTCTTAATCTTTATTGTAACTGCTTTTATGGGTTACGTTTTACCATGGGGACAAATGAGTTTCTGGGGAGCAACTGTAATTACTAATTTATTATATTTTATTCCTGGATTAATTAATTGGGTCTGTGGTGGATTCATTATTAATGACCCAACATTAAAAAGATTCTTCGTCTTACATTTTATATTTCCATTTGTAGCCTTAGCTATTGTATTTATTCATATATTCTTCTTACATATTCATGGTAGCACTAATCCTTTAGGGTATGATACACCTTTAAAAATACCATTCTATCCAAATCTATTAACTTTAGATATTAAAGGATTTAACTATGTATTAGTTATATTTTTATTTCAAAGTTTATTTGGAATTGC | 479 | Asia |
| *Haemoproteus* | TYTAL1 | JN863575 | Full | 2 | TYTAL4, STTRA01 | AAGAGGATTAAATTATTCATATTCATATTTACCTTTATCATGGATAACTGGACTAATGATTTTCTTAATTTCTATTGTTACTGCTTTTATGGGTTATGTATTACCTTGGGGTCAAATGAGTTTCTGGGGTGCAACCGTTATTACTAACTTATTATATTTTATACCTGGACTTGTTTCATGGATTTGTGGTGGATATAATATTAGTGATCCTACTTTAAAAAGATTCTTTGTATTACATTTTATATTTCCATTTGTAGCTTTATGTATTGTATTTATACATATATTCTTTTTACACTTACAAGGTAGCTCTAATCCTTTAGGATATGATACAGCTTTAAAAATACCTTTCTATCCAAGTCTATTATGTTTAGATATTAAAGGATTTAGTAATGTATTAGTATTATACTTAGCTCAAAGTTTATTTGGTATATT | 432 | Europe |
| *Haemoproteus* | ZOMON01 | JX418175 | Full | 2 | ZOSPAL07, ZOSPAL02 | TTTGTCTTTATTTTAACTTATTTACATATATTAAGAGGATTAAATTATTCATATTCATATTTACCATTATCATGGATAACAGGATTAGTAATATTTTTAATTTCTATTGTTACTGCTTTTATGGGTTATGTACTACCTTGGGGTCAAATGAGTTTCTGGGGTGCAACCGTTATTACTAATTTATTATATTTTATACCTGGATTAGTTTCATGGATTTGTGGTGGATATATTATTAGTGATCCAACTTTAAAAAGATTTTTTGTATTACATTTTATATTCCCATTTATAGCTTTATGTATTGTATTTATACATATATTCTTTTTACACTTACAAGGTAGCTCTAATCCTTTAGGATATGATACTGCTTTAAAAATACCTTTCTATCCAAGTCTATTATGTCTAGATATTAAAGGATTTAATAATGTATTAGTCTTATTTCTAGCACAAAGTTTATTCGGAATTTT | 464 | Asia |
| *Haemoproteus* | ZOSPAL02 | MF565821 | Full | 2 | ZOSPAL07, ZOMON01 | ACTGGTGCTACATTTGTCTTTATTTTAACTTATTTACATATATTAAGAGGATTAAATTATTCATATTCATATTTACCATTATCATGGATAACAGGATTAGTAATATTTTTAATTTCTATTGTTACTGCTTTTATGGGTTATGTACTACCTTGGGGTCAAATGAGTTTCTGGGGTGCAACCGTTATTACTAATTTATTATATTTTATACCTGGATTAGTTTCATGGATTTGTGGTGGATATATTATTAGTGATCCAACTTTAAAAAGATTTTTTGTATTACATTTTATATTCCCATTTATAGCTTTATGTATTGTATTTATACATATATTCTTTTTACACTTACAAGGTAGCTCTAATCCTTTAGGATATGATACTGCTTTAAAAATACCTTTCTATCCAAGTCTATTATGTCTAGATATTAAAGGATTTAATAATGTATTAGTCTTATTTCTAGCACA | 458 | Asia |
| *Plasmodium* | BAEBIC02 | AF465555 | Full | 2 | TABI07, SEIAUR02 | GCAACAGGTGCATCATTTGTATTTATTCTTACATATTTACATATTTTAAGAGGATTAAATTATTCTTATTCTTATTTACCTTTATCATGGATATCAGGATTAATAATATTTTTAATATCAATAGTTACTGCTTTTATGGGATATGTACTACCTTGGGGTCAAATGAGTTTCTGGGGTGCAACCGTCATTACTAATTTATTATATTTTATACCTGGTCTTGTTTCATGGATCTGTGGTGGATATCTTGTAAGCGACCCAACATTAAAAAGATTTTTTGTATTACATTTTATATTTCCATTTATAGCCTTATGTATTGTATTTATACATATATTCTTTCTACATTTACAAGGTAGCACAAATCCTTTAGGGTATGATACAGCTTTAAAAATACCCTTCTATCCAAATCTATTAAGTCTTGATATTAAAGGATTTAATAATATCTTAGTTTTATTTTTAGCACAAAGCTTATTTGGAATATT | 479 | North_America, Central_America, South_America |
| *Plasmodium* | CINCHL01 | DQ659577 | Full | 2 | RBQ16, CINCHL02 | GCAACTGGTGCTTCATTTGTATTTATTTTAACTTATTTACATATTTTAAGAGGATTAAATTACTCATATTCATATTTACCTTTATCATGGATATCTGGATTAATAATATTTTTAATATCTATAGTAACAGCTTTTATGGGTTATGTATTACCTTGGGGTCAAATGAGTTTCTGGGGTGCTACCGTAATAACTAATTTACTATATTTTATACCTGGACTTGTTTCATGGATATGTGGTGGATATCTTGTAAGTGACCCAACTTTAAAAAGATTCTTTGTATTACATTTTACATTTCCATTTATAGCTTTATGTATTGTATTTATACATATATTCTTTTTACATTTACAAGGTAGCACAAATCCTTTAGGGTATGATACAGCTTTAAAAATACCCTTCTATCCAAATCTTTTAAGTCTTGATATTAAAGGATTTAATAATGTATTAGTATTATTTTTAGCACAAAGTTTATTTGGAATATT | 479 | Europe, South_Sahara |
| *Leucocytozoon* | CNEORN01 | DQ355976 | Full | 2 | SILUT01, DENCOR06 | TCAACCGGTGCATCTTTTGTATTTATATTAACATATCTACATATACTAAGAGGTTTAAATTACTCTTTCTCTTACTTACCTTTATCATGGATAAGTGGTTTAGTAATATTCTTAATATTTATTGTAACTGCTTTTATGGGTTATGTCTTACCATGGGGTCAAATGAGTTTCTGGGGAGCTACTGTAATTACTAACTTATTATATTTTATTCCTGGATTAATTAATTGGGTTTGTGGTGGTTTTATTATTAACGATCCAACTCTAAAAAGATTCTTTGTATTACATTTTATATTCCCATTCGTAGCTTTAGCTATTGTATTTATTCATATATTCTTCTTACATATTCAAGGTAGCACTAATCCATTAGGGTATGATACACCTTTAAAAATACCATTCTATCCAAATCTATTAACTTTAGATGTTAAAGGATTTAATTATGTATTAGTAATATTCTTATTTCAAAGTTTATTTGGTATTGC | 479 | North_America, Central_America, South_America |
| *Plasmodium* | DELFRA04 | MN202213 | Full | 2 | CYAOLI10, CINPRE02 | GCAACAGGTGCTTCATTTGTATTTATTTTAACTTATTTACATATTTTAAGAGGATTAAATTACTCATACTCCTACTTACCTTTATCATGGATGTCAGGGTTAATAATATTTCTAATATCTATAGTAACAGCTTTTATGGGTTATGTATTACCATGGGGTCAAATGAGTTTCTGGGGTGCAACTGTAATTACTAATTTATTATATTTTATACCTGGACTTGTTTCATGGATATGCGGAGGATATCTTGTAAGTGACCCAACTTTAAAAAGATTCTTTGTATTACATTTTATATTCCCATTTGTAGCCTTATGTATTGTATTTATACATATATTCTTCTTACATTTACAAGGTAGCACAAATCCTTTAGGGTATGATACAGCTTTAAAAATACCCTTCTATCCAAATCTTTTAAGTCTTGATATAAAAGGATTTAACAATATATTAGTATTATTTTTATCACAAAGTTTATTTGGAATATT | 479 | - |
| *Plasmodium* | DIGLAF01 | KF537276 | Full | 2 | TUMIG05, DIGCYA02 | GCAACAGGTGCTTCATTTGTTTTCATTTTAACCTATTTACATATTTTAAGAGGATTAAATTATTCATATTCATATTTACCTTTATCATGGATTTCAGGATTATTAATATTTTTAATATCTATAGTAACAGCTTTTATGGGTTATGTATTACCTTGGGGTCAAATGAGTTTCTGGGGTGCTACTGTTATAACTAATTTATTATATTTTATACCTGGACTTGTCTCATGGATTTGTGGTGGATATCTTGTAAGTGACCCAACCTTAAAAAGATTTTTTGTATTACATTTTACATTCCCATTTATAGCTTTATGTATTGTATTTATACATATATTCTTCTTACATTTACAAGGTAGCACAAATCCTTTAGGGTATGATACAGCTTTAAAAATACCCTTCTATCCAAATCTATTAAGTCTTGATATTAAAGGATTTAATAATGTATTAGTTTTATTCCTATCTCAAAGTTTATTTGGAATTTT | 479 | North_America, South_America |
| *Haemoproteus* | GEMON01 | FJ462651 | Partial | 2 | COLINC01, MICRO01 | GCCACAGGTGCATCATTTGTATTTATTTTAACATACCTACACATTTTAAGAGGATTAAATTACTCATATTCATATTTACCATTATCATGGATTACCGGATTAATAATATTTATTATCTCTATTATGACTGCTTTCTTAGGTTATGTTCTACCTTGGGGTCAAATGAGTTTCTGGGGTGCAACTGTTATTACTAATTTATTATATTTTATTCCAGGATTAGTCTCATGGATTTGTGGTGGATATATTGTTAGTGATCCTACACTAAAAAGATTCTTTGTATTACATTTTATATTTCCATTTATAGCTATATGTATAGTATTTATTCATATATTCTTTTTACATTTACA | 347 | Central_America, South_America |
| *Haemoproteus* | PASILI01 | KJ584597 | Full | 2 | DENCOR01, MELGEO01 | CTACCGGTGCTACATTTGTTTTTATTTTAACTTACTTACATATATTAAGAGGATTAAACTACTCATATTCTTACTTACCTTTATCATGGATAACTGGATTAGTAATATTCTTAATCTCTATTGTTACCGCTTTTATGGGTTATGTATTACCTTGGGGTCAAATGAGTTTCTGGGGTGCAACCGTTATTACTAATTTATTATATTTTATACCTGGACTTGTTTCATGGATTTGTGGAGGATATACTATTAGTGATCCAACTTTAAAAAGATTTTTTGTATTACATTTTATATTTCCTTTTATAGCTTTATGTATTGTATTCATACATATATTCTTCTTACACTTACAAGGTAGCTCTAATCCTTTAGGATATGATACAGCTTTAAAAATACCTTTCTATCCAAGTCTATTATGTCTAGATATCAAAGGATTTAATAATGTATTAGTCCTATTTCTAGCACAAAGTTTATTTGGAATTCT | 478 | North_America |
| *Plasmodium* | SIAMEX02 | EU254538 | Partial | 2 | RBQ16, TROAED24 | GCAACTGGTGCTTCATTTGTATTTATTTTAACTTATTTACATATTTTAAGAGGACTAAATTATTCATATTCATATTTACCTTTATCATGGATATCTGGATTAATAATATTTTTAATATCTATAGTAACAGCTTTTATGGGTTATGTATTACCTTGGGGTCAAATGAGTTTCTGGGGTGCTACTGTAATTACTAATTTATTATATTTTATACCTGGACTTGTTTCATGGATATGTGGTGGATATCTTGTAAGCGACCCAACTTTAAAAAGATTCTTTGTATTACATTTTACATTTCCATTTATAGCTTTATGTATTGTATTTATACATATATTCTTTTTACATTTACAAGGTAGCACAAATC | 361 | North_America |
| *Haemoproteus* | TYTAL4 | MK390810 | Full | 2 | STTRA01, TYTAL1 | GCTACTGGTGCTACATTTGTTTTTATATTAACATATTTACATATCTTAAGAGGATTAAATTATTCATATTCATATTTACCTTTATCATGGATAACTGGACTAATGATTTTCTTAATTTCTATTGTTACTGCTTTTATGGGTTATGTATTACCTTGGGGTCAAATGAGTTTCTGGGGTGCAACCGTTATTACTAACTTATTATATTTTATACCTGGACTTGTTTCATGGATTTGTGGTGGATATAATATTAGTGATCCTACTTTAAAAAGATTCTTTGTATTACATTTTATATTTCCATTTGTAGCTTTATGTATTGTATTTATACATATATTCTTTTTACACTTACAAGGTAGCTCTAATCCTTTAGGATATGATACAGCTTTAAAAATACCTTTCTATCCAAGTCTATTATGTTTAGATATTAAAGGATTTAGTAATGTATTAGTATTATACTTAGCTCAAAGTTTATTTGGTATATT | 479 | Asia |
| *Haemoproteus* | ZOSPAL07 | MK493400 | Full | 2 | ZOMON01, ZOSPAL02 | GCTACTGGTGCTACATTTGTCTTTATTTTAACTTATTTACATATATTAAGAGGATTAAATTATTCATATTCATATTTACCATTATCATGGATAACAGGATTAGTAATATTTTTAATTTCTATTGTTACTGCTTTTATGGGTTATGTACTACCTTGGGGTCAAATGAGTTTCTGGGGTGCAACCGTTATTACTAATTTATTATATTTTATACCTGGATTAGTTTCATGGATTTGTGGTGGATATATTATTAGTGATCCAACTTTAAAAAGATTTTTTGTATTACATTTTATATTCCCATTTATAGCTTTATGTATTGTATTTATACATATATTCTTTTTACACTTACAAGGTAGCTCTAATCCTTTAGGATATGATACTGCTTTAAAAATACCTTTCTATCCAAGTCTATTATGTCTAGATATTAAAGGATTTAATAATGTATTAGTCTTATTTCTAGCACAAAGTTTATTCGGAATTTT | 479 | Asia |
| *Haemoproteus* | ANSOM01 | KM211350 | Full | 1 | ANIFLA01 | CTAACTTACTTACATATTTTAAGAGGGTTAAACTATTCATATTCTTATTTACCTTTATCATGGATAACTGGATTAGTTATATTCTTAATTTCAATTGTTACCGCTTTTATGGGTTATGTATTACCTTGGGGTCAAATGAGTTTCTGGGGTGCAACCGTTATTACTAATTTATTATATTTTATTCCTGGACTTGTTTCATGGATTTGTGGAGGATATACTATTAGTGATCCAACTCTAAAAAGATTTTTTGTATTACATTTTATATTTCCTTTTATAGCTTTATGTATTGTATTTATACATATATTCTTTTTACACTTACAAGGTAGCTCTAATCCTTTAGGATATGATACAGCTTTAAAAATACCTTTCTATCCAAGTCTATTATGTCTAGATATCAAAGGATTTAATAATGTATTAGTCCTATTTCTAGCACAAAGTTTATTTGGAATTTT | 452 | South_America |
| *Leucocytozoon* | COLBF01 | KR052933 | Full | 1 | COLBF09 | CAACTGGTGCATCTTTTGTCTTTATATTAACATATCTACATATATTAAGAGGACTAAACTATTCTTACTCATACCTACCTCTATCATGGATCACAGGTTTAATTATATTCTTAATATCCATAATGACAGCCTTTATGGGTTATGTATTACCATGGGGACAAATGAGTTATTGGGGAGCAACTGTAATTACCAATCTATTATACTTTATTCCTGGATTAATCTCATGGGTCTGTGGAGGATTCGTGGTTAATGATCCAACTCTAAAAAGATTCTTTGTACTTCATTTTATTTTCCCATTTGTAGCATTAATTATAGTATTTATTCACATATTCTACTTACATCTACAAGGTAGCACTAATCCTTTAGGATATGATACAGCTCTAAAAATACCCTTCTATCCAAATCTTTTATGTCTAGATATTAAAGGATTTGCAAATATACTAGTATTATTCCTAGCACAAAGTTTATTTGGAATAT | 477 | North_America |
| *Leucocytozoon* | COLBF24 | KR052956 | Full | 1 | COLBF09 | CAACTGGTGCATCTTTTGTCTTTATATTAACATATCTACATATATTAAGAGGACTAAACTATTCTTACTCATACCTACCTCTATCATGGATCACAGGTTTAATTATATTCTTAATATCCATAATGACAGCCTTTATGGGTTATGTATTACCATGGGGACAAATGAGTTATTGGGGAGCAACTGTAATTACCAATCTATTATACTTTATTCCTGGATTAATCTCATGGGTCTGTGGAGGATTCGTGGTTAATGATCCAACTCTAAAAAGATTCTTTGTACTTCATTTTATTTTCCCATTTGTAGCATTAATTATAGTATTTATTCACATATTCTACTTACATCTACAAGGTAGCACTAATCCTTTAGGATATGATACAGCTCTAAAAATACCCTTCTATCCAAATCTTTTATGTCTAGATATTAAAGGATTTGCAAATATACTAGTATTATTCCTAGCACAAAGTTTATTTGGAATT | 476 | North_America |
| *Plasmodium* | CUCROC01 | MF442562 | Full | 1 | SYBOR21 | GCAACAGGAGCTTCATTTGTATTTATTTTAACTTATCTACATATTTTAAGAGGATTAAATTATTCTTATTCATATCTACCTTTATCATGGATTTCAGGATTAATTATATTTTTAATATCTATAGTTACTGCTTTTATGGGATATGTATTACCTTGGGGTCAAATGAGTTTTTGGGGAGCAACCGTAATTACTAACTTATTATATTTTATTCCAGGACTTGTTTCATGGATCTGTGGTGGATATTTAGTTAGTGACCCAACATTAAAAAGATTTTTCGTATTACATTTTACATTTCCATTTATAGCTTTATGTATTGTATTTATACATATATTCTTTTTACATCTACAAGGTAGCACAAATCCTTTAGGGTATGATACAGCTTTAAAAATACCCTTCTATCCAAATCTATTAAGTCTCGATATTAAAGGATTTAATAATGTATTAGTATTATTTTTAGCACAAAGTTTATTTGGAATTTT | 479 | South_Sahara |
| *Plasmodium* | PORUF03 | KJ145067 | Partial | 1 | MONFAI01 | TATTTACATATTTTAAGAGGATTAAATTATTCATATTCATATTTACCTTTATCATGGATTTCAGGATTATTAATATTTTTAATATCTATAGTAACAGCTTTTATGGGTTATGTATTACCTTGGGGTCAAATGAGTTTCTGGGGTGCTACTGTTATAACTAATTTATTATATTTTATACCTGGACTTGTCTCATGGATTTGTGGTGGATATCTTGTAAGTGACCCAACCTTAAAAAGATTCTTTGTATTACATTTTACATTCCCATTTATAGCTTTATGTATTGTATTTATACATATATTCTTCTTACATTTACAAGGTAGCACAAATCCTTTAGGGTATGATACAGCTTTAAAAATACCCTTCTATCCAAATCTATTAAGTCTTGATATTAAAGGATTTAATAATGTATTAGTTTTATTCTTATCACAAAGTTTATTTGGAATTCT | 446 | Asia |
| *Leucocytozoon* | SEINOV02 | JQ764623 | Full | 1 | ACAFLA03 | TTTGTATTTATATTAACATATTTACATATATTAAGAGGATTAAATTATTCATTTACTTACTTACCTTTATCATGGATAAGTGGTTTAATAATATTCTTAATATTTATTGTAACTGCTTTTATGGGTTATGTCTTACCATGGGGTCAAATGAGTTTTTGGGGAGCTACTGTTATAACTAATTTATTATATTTTATTCCTGGATTAATTAATTGGGTTTGCGGTGGATTTATTATTAACGACCCAACTCTAAAAAGATTCTTCGTATTACATTTTATATTCCCATTTGTAGCATTAGCTATAGTATTTATACATATATTCTTCTTACATATTCAAGGTAGCACTAATCCTTTAGGGTATGATACACCTTTAAAAATACCATTCTATCCAAATCTATTAACTTTAGATGTTAAAGGATTTAACTATGTATTAGTATTATTCCTATTTCAAAGTTTATTTGGAATTGC | 464 | North_America, South_America |
| *Plasmodium* | SYBOR21 | EF032871 | Full | 1 | CUCROC01 | CTTCATTTGTATTTATTTTAACTTATCTACATATTTTAAGAGGATTAAATTATTCTTATTCATATCTACCTTTATCATGGATTTCAGGATTAATTATATTTTTAATATCTATAGTTACTGCTTTTATGGGATATGTATTACCTTGGGGTCAAATGAGTTTTTGGGGAGCAACCGTAATTACTAACTTATTATATTTTATTCCAGGACTTGTTTCATGGATCTGTGGTGGATATTTAGTTAGTGACCCAACATTAAAAAGATTTTTCGTATTACATTTTACATTTCCATTTATAGCTTTATGTATTGTATTTATACATATATTCTTTTTACATCTACAAGGTAGCACAAATCCTTTAGGGTATGATACAGCTTTAAAAATACCCTTCTATCCAAATCTATTAAGTCTCGATATTAAAGGATTTAATAATGTATTAGTATTATTTTTAGCACAAAGTTTATTTGGAATTTT | 469 | Europe, South_Sahara |
| *Haemoproteus* | TURTYM01 | JX275888 | Partial | 1 | TURTYM02 | TTTGTATTTATTTTAACATACTTACATATTCTAAGAGGATTAAATTATTCTTATTCTTATTTACCATTATCATGGATTACCGGATTAATAATATTTTTAATCTCTATTGTAACTGCTTTTATGGGTTACGTTTTACCTTGGGGTCAAATGAGTTTCTGGGGTGCAACTGTTATAACTAATTTACTATATTTTATTCCTGGATTAGTATCATGGATTTGTGGTGGATATATAGTTAGTGACCCAACCCTAAAAAGATTCTTTGTATTACATTTTATATTTCCATTTATAGCAATATGTATAGTATTTATACATATATTCTTTCTACATTTACAAGGTAGCTCTAATCCTTTAGGATATGATACAGCTTTAAAAATACCCTTCTATCCAAG | 389 | South_Sahara |
| *Haemoproteus* | ACAED01 | EF380189 | Partial | 1 | ACDUM3 | GGTGCAACCGTTATTACTAATTTATTATATTTTATTCCTGGACTTGTATCATGGATTTGTGGTGGATATACTATTAGTGATCCAACCTTAAAAAGATTCTTCGTATTACATTTTATATTTCCATTTATAGCCTTATGTATTGTTTTTATACATATATTCTTCTTACACTTACAAGGTAGCTCTAATCCTTTAGGATATGATACAGCTTTAAAAATACCTTTCTATCCAAGTCTATTATGTTTAGATGTTAAAGGATTTAATAATGTATTAGTTATATTCTTAGCACAAAGTTTATTTGGAATTCT | 305 | Asia |
| *Leucocytozoon* | ACAFLA02 | MG726138 | Full | 1 | CARFLA04 | TCAACAGGTGCATCATTTGTATTTATATTAACATATCTACATATATTAAGAGGATTAAATTATTCTTTTACTTACTTACCTTTATCATGGATAAGTGGTTTAGTAATATTCTTAATATTTATTGTAACTGCTTTTATGGGTTATGTCTTACCATGGGGTCAAATGAGTTTTTGGGGAGCTACTGTCATTACTAATTTATTATATTTTATTCCTGGACTAATTAATTGGGTTTGTGGTGGATTTATTATTAACGACCCAACTCTAAAAAGATTCTTCGTATTACATTTTATATTCCCATTTGTAGCATTAGCTATTGTATTTATACATATATTCTTCTTACATATTCAAGGTAGCACTAATCCTTTAGGGTATGATACACCTTTAAAAATACCATTCTATCCAAATCTATTAACTTTAGATGTTAAAGGATTTAACTATGTATTAGTATTATTCCTATTTCAAAGTTTATTTGGAATTGC | 479 | North_America |
| *Leucocytozoon* | ACAFLA03 | MG726132 | Full | 1 | SEINOV02 | TCAACAGGTGCATCATTTGTATTTATATTAACATATTTACATATATTAAGAGGATTAAATTATTCATTTACTTACTTACCTTTATCATGGATAAGTGGTTTAATAATATTCTTAATATTTATTGTAACTGCTTTTATGGGTTATGTCTTACCATGGGGTCAAATGAGTTTTTGGGGAGCTACTGTTATAACTAATTTATTATATTTTATTCCTGGATTAATTAATTGGGTTTGCGGTGGATTTATTATTAACGACCCAACTCTAAAAAGATTCTTCGTATTACATTTTATATTCCCATTTGTAGCATTAGCTATAGTATTTATACATATATTCTTCTTACATATTCAAGGTAGCACTAATCCTTTAGGGTATGATACACCTTTAAAAATACCATTCTATCCAAATCTATTAACTTTAGATGTTAAAGGATTTAACTATGTATTAGTATTATTCCTATTTCAAAGTTTATTTGGAATTGC | 479 | North_America |
| *Leucocytozoon* | AEGCAU03 | KT757542 | Full | 1 | FIPAR02 | GCCACAGGTGCTACATTTGTATTTATTTTAACTTACTTACATATTTTAAGAGGATTAAATTATTCATACTCTTACTTACCTTTATCATGGATAACTGGATTATTAATATTCTTAATTTCTATTGTTACTGCTTTTATGGGTTATGTATTACCTTGGGGTCAAATGAGTTTCTGGGGTGCAACTGTTATCACTAATTTATTATATTTTATACCTGGACTAGTTTCATGGATTTGTGGAGGATATATTATAAGTGATCCAACTTTAAAAAGATTTTTTGTATTACATTTTATATTCCCTTTTATAGCCCTATGTATTGTGTTTATACATATATTTTTCTTACATTTACAAGGTAGCTCTAATCCTTTAGGATATGATACAGCTTTAAAAATACCTTTCTATCCAAGTCTATTATGTCTAGATATCAAAGGATTT | 432 | Asia |
| *Leucocytozoon* | AFR238 | KM056552 | Full | 1 | PASDIF03 | CAACAGGTGCATCTTTTGTATTTATATTAACCTATCTACATATATTAAGAGGATTAAACTATTCTTTCTCTTACTTACCTTTATCATGGTATAGTGGTTTAGTTATATTCTTAATCTTTATTGTAACTGCTTTTATGGGTTACGTCTTACCATGGGGACAAATGAGTTTCTGGGGAGCAACTGTAATTACTAATTTATTATATTTTATTCCTGGATTAATTAATTGGGTCTGTGGTGGATTTATTATTAATGACCCAACATTAAAAAGATTCTTTGTATTACACTTTATATTCCCATTTGTAGCATTAGCTATTGTATTTATTCATATATTCTTTTTACATATTCATGGTAGCACTAATCCTTTAGGGTATGATACACCTTTAAAAATACCATTCTATCCAAATCTATTAACCTTAGATATTAAAGGATTTAACTATGTATTAGTTATATTTTTATTT | 458 | South_Sahara |
| *Leucocytozoon* | AFR241 | KM056554 | Full | 1 | AFR176 | CAACAGGTGCATCATTCGTATTTATATTAACATACTTACATATATTAAGAGGATTAAATTATTCTTTCACTTACTTACCTTTATCATGGATAAGTGGTTTAATAATATTCTTAATATTTATTGTAACTGCTTTTATGGGTTACGTCTTACCATGGGGTCAAATGAGTTTTTGGGGAGCTACTGTTATTACTAATTTATTATATTTTATTCCTGGACTAATTAATTGGGTTTGTGGTGGATTTATTATTAATGACCCAACTTTAAAAAGATTCTTCGTATTACATTTTATATTCCCATTTGTAGCATTAGCTATTGTATTTATACATATATTCTTCTTACATATTCAAGGTAGCACTAATCCTTTAGGGTATGATACACCTTTAAAAATACCATTCTATCCAAATCTATTAACTTTAGATGTTAAAGGATTTAACTATGTATTAGTATTATTCCTATTT | 458 | South_Sahara |
| *Haemoproteus* | ALMOR06 | KJ145089 | Partial | 1 | ALCPOI02 | TGGGGTCAAATGAGTTTCTGGGGTGCAACCGTTATAACTAATTTATTATATTTTATACCTGGACTAGTCTCATGGATTTGTGGTGGATATATTATTAGTGATCCAACTTTAAAAAGATTTTTCGTATTACATTTTATATTCCCTTTTATTGCTTTATGTATTGTATTTATACATATATTCTTTTTACACTTACAAGGTAGCTCTAATCCTTTAGGATATGATACTGCTTTAAAAATACCTTTCTATCCAAGTCTATTATGTCTAGATATTAAAGGATTTAATAATGTATTAGTCTTATTTCTAGCACAAAGTTTATTTGGAATATT | 326 | Asia |
| *Leucocytozoon* | ANACU02 | KC409120 | Partial | 1 | ANACRE02 | TTACTTACCTTTATCATGGAGTAGTGGTTTAATTATATTCTTAATATTTATTGTTACTGCTTTCATGGGATATGTTTTACCATGGGGTCAAATGAGTTTCTGGGGAGCAACTGTAATTACTAATTTATTATATTTTATTCCTGGATTAATTAATTGGGTTTGTGGTGGATTTATAATTAATGATCCAACATTAAAAAGATTCTTTGTATTACATTTTATATTCCCATTCGTAGCTTTAGCTATTGTATTTATACATATATTCTTCTTACATATTCAAGGTAGCACTAATCCTCTAGGGTATGATACACCTTTAAAAATACCATTCTATCCAAGTCTATTAACTTTAGACATTAAAGGA | 358 | North_America |
| *Haemoproteus* | ANIFLA01 | MH457294 | Full | 1 | ANSOM01 | TACCGGTGCTACATTTGTTTTTATTCTAACTTACTTACATATTTTAAGAGGGTTAAACTATTCATATTCTTATTTACCTTTATCATGGATAACTGGATTAGTTATATTCTTAATTTCAATTGTTACCGCTTTTATGGGTTATGTATTACCTTGGGGTCAAATGAGTTTCTGGGGTGCAACCGTTATTACTAATTTATTATATTTTATTCCTGGACTTGTTTCATGGATTTGTGGAGGATATACTATTAGTGATCCAACTCTAAAAAGATTTTTTGTATTACATTTTATATTTCCTTTTATAGCTTTATGTATTGTATTTATACATATATTCTTTTTACACTTACAAGGTAGCTCTAATCCTTTAGGATATGATACAGCTTTAAAAATACCTTTCTATCCAAGTCTATTATGTCTAGATATCAAAGGATTTAATAATGTATTAGTCCTATTTCTAGCACAAAGTTTATTTGGAATTTT | 477 | South_America |
| *Haemoproteus* | ANLAT02 | EU770153 | Full | 1 | PHICT03 | CTACATTTGTCTTTATTTTAACTTATTTACATATATTAAGAGGATTAAATTATTCATATTCATACTTACCTTTATCATGGATAACTGGATTACTAATATTCTTAATTTCTATTGTTACTGCTTTTATGGGTTATGTATTACCTTGGGGTCAAATGAGTTTCTGGGGTGCAACCGTTATAACTAATTTATTATATTTTATACCTGGACTTGTATCATGGATTTGTGGTGGATATATTATTAGTGATCCAACTTTAAAAAGATTTTTTATATTACATTTTATTTTCCCATTTATAGCTTTATGTATTGTATTTATACATATATTCTTTTTACATTTACAAGGTAGCTCTAATCCTTTAGGATATGATACTGCTTTAAAAATACCTTTCTATCCAAGTCTATTATGTCTAGATATTAAAGGATTTAATAATGTATTAGTCTTATTTCTAGCACAAAGTTTATTTGGAATTT | 468 | South_Sahara |
| *Leucocytozoon* | APSPI04 | EF153655 | Partial | 1 | ELAALB05 | TAAACTATTCATTCTCTTACTTACCTTTATCATGGTATAGTGGTTTAGTTATATTCTTAATCTTTATTGTAACTGCTTTTATGGGTTACGTTTTACCATGGGGACAAATGAGTTTCTGGGGAGCAACTGTAATTACTAATTTATTATATTTTATTCCTGGATTAATTAATTGGGTCTGTGGTGGATTCATTATTAATGACCCAACACTAAAAAGATTCTTCGTATTACACTTTATATTCCCATTTGTAGCCTTAGCTATTGTATTTATTCATATATTCTTCTTACATATTCATGGTAGCACTAATCCTTTAGGGTATGATACACCTTTAAAAATACCATTCTATCCAAATCTATTAACTTTAGATATTAAAGGATTTAACTATGTATTAGTTATATTTTTATTTCAAAGTTTATTTGGAATTGC | 424 | South_America |
| *Leucocytozoon* | APSPI05 | EF153656 | Full | 1 | DIUDIU11 | ATATCTACATATTTTAAGAGGATTAAACTATTCATTCTCTTACTTACCTTTATCATGGTATAGTGGTTTAATTATATTCTTAATCTTTATTGTAACTGCTTTTATGGGTTACGTTTTACCATGGGGACAAATGAGTTTCTGGGGAGCAACTGTAATTACTAATTTATTATATTTTATTCCTGGATTAATTAATTGGGTCTGTGGTGGATTCATAATTAATGACCCAACATTAAAAAGATTCTTCGTATTACACTTTATATTCCCATTTATAGCCTTAGCTATTGTATTTATTCATATATTCTTCTTACATATTCATGGTAGCACTAATCCTTTAGGGTATGATACACCTTTAAAAATACCATTCTATCCAAATCTATTAACTTTAGATATTAAAGGATTTAACTATGTATTAGTTATATTTTTATTTCAAAGTTTATTTGGAATTGC | 447 | South_America |
| *Haemoproteus* | ARBRU01 | KF537292 | Full | 1 | ATLBRU01 | GCTACCGGTGCTACATTTGTTTTTATTCTAACTTACTTACATATATTAAGAGGATTAAATTATTCATATTCTTATTTACCTTTATCATGGATAACTGGATTATTTATATTCTTAATTTCAATTGTTACCGCTTTTATGGGTTATGTATTACCTTGGGGTCAAATGAGTTTCTGGGGTGCAACCGTTATAACTAATTTATTATATTTTATTCCTGGACTTGTTTCATGGATTTGTGGAGGATATACTATTAGTGATCCAACTTTAAAAAGATTTTTTGTATTACATTTTATATTTCCTTTTATAGCTTTATGTATTGTATTTATACATATATTCTTCTTACACTTACAAGGTAGCTCTAATCCTTTAGGATATGATACAGCTTTAAAAATACCTTTCTATCCAAGTCTATTATGTCTAGATATCAAAGGATTTAATAATGTATTAGT | 446 | South_America |
| *Haemoproteus* | ASOT07 | JN863577 | Full | 1 | STRURA03 | AAGAGGATTAAATTATTCATATTCATATTTACCTTTATCATGGATAACTGGATTAACTATTTTCTTAATTTCTATTGTAACTGCTTTTATGGGTTATGTATTACCTTGGGGTCAAATGAGTTTCTGGGGTGCAACCGTTATTACTAACTTATTATATTTTATTCCTGGACTTGTTTCATGGATTTGTGGTGGATATAATATTAGTGATCCTACTTTAAAAAGATTCTTTATATTACATTTTATATTTCCATTTATAGCTTTATGTATTGTATTTATACATATATTCTTTTTACATTTACAAGGTAGTTCTAATCCTTTAGGATATGATACAGCTTTAAAAATACCTTTCTATCCAAGTCTATTATGTTTAGATATTAAAGGATTTAGTAATATATTAGTATTATATTTAGCTCAAAGTTTATTTGGTATATT | 432 | Europe |
| *Plasmodium* | BELL02 | JN415759 | Full | 1 | CYNOV1 | ATTTTAACTTATTTACATATTTTAAGAGGATTAAATTATTCATATTCATATTTACCTTTATCATGGATATCTGGATTAATAATATTCTTAATATCTATAGTAACAGCTTTTATGGGTTACGTATTACCTTGGGGTCAAATGAGTTTCTGGGGTGCTACCGTAATAACTAATTTATTATACTTTATACCTGGACTTGTTTCATGGATATGTGGTGGATATCTTGTAAGTGACCCAACTTTAAAAAGATTCTTTGTATTACATTTTACATTTCCTTTTATAGCTTTATGTATTGTATTTATACATATATTCTTTTTACATTTACAAGGTAGCACAAATCCTTTAGGGTATGATACAGCTTTAAAAATACCCTTCTATCCAAATCTTTTAAGTCTTGATATTAAAGGATTTAATAATGTATTAGTTTTATTTTTAGCACAAAGTTTATTTGGAATATT | 455 | Australia_._New_Zeeland |
| *Leucocytozoon* | BUTBUT05 | MT281506 | Full | 1 | GALLUS34 | TCAACAGGTGCATCTTTTGTTTTTATATTAACATATTTACATATATTAAGAGGATTAAATTATTCTTTCTCTTATTTACCTTTATCATGGATTAGTGGTTTAGTAATATTCTTAATATTTATTGTAACTGCTTTTATGGGTTATGTCTTACCATGGGGACAAATGAGTTTCTGGGGAGCTACTGTTATAACTAATTTATTATATTTTATTCCTGGATTAATTAATTGGGTTTGTGGTGGATTTATTATTAATGACCCAACATTAAAAAGATTCTTTGTATTACACTTTATATTCCCATTTATAGCATTAGCAATTGTATTTATTCATATATTCTTTTTACATATTCAAGGTAGCACTAATCCTTTAGGGTATGATACACCTTTAAAAATACCATTCTATCCAAATCTATTAACTTTAGATATTAAAGGATTTAATTATGTACTAGTAATATTCTTAT | 457 | Asia |
| *Leucocytozoon* | CARFLA04 | KF314788 | Full | 1 | ACAFLA02 | TCAACAGGTGCATCATTTGTATTTATATTAACATATCTACATATATTAAGAGGATTAAATTATTCTTTTACTTACTTACCTTTATCATGGATAAGTGGTTTAGTAATATTCTTAATATTTATTGTAACTGCTTTTATGGGTTATGTCTTACCATGGGGTCAAATGAGTTTTTGGGGAGCTACTGTCATTACTAATTTATTATATTTTATTCCTGGACTAATTAATTGGGTTTGTGGTGGATTTATTATTAACGACCCAACTCTAAAAAGATTCTTCGTATTACATTTTATATTCCCATTTGTAGCATTAGCTATTGTATTTATACATATATTCTTCTTACATATTCAAGGTAGCACTAATCCTTTAGGGTATGATACACCTTTAAAAATACCATTCTATCCAAATCTATTAACTTTAGATGTTAAAGGATTTAACTATGTATTAGTATTATTCCTATTTCAAAGTTTATTTGGAAT | 476 | North_America |
| *Leucocytozoon* | CATBIC07 |  | Full | 1 | CATFUS17 | TATCTACATATCTTAAGAGGTTTAAATTATTCATTCTCTTATTTACCTTTATCATGGTATACAGGTTTAATAATATTCTTAATATTCATTGTAACTGCTTTTATGGGTTACGTATTACCATGGGGACAAATGAGTTTCTGGGGAGCAACTGTTATTACTAATTTATTATATTTTATTCCTGGATTAATCAATTGGGTATGTGGTGGATTTATTATTAATGATCCAACCCTAAAAAGATTCTTCGTATTACATTTTATATTCCCATTTGTAGCTTTAGCTATTGTATTTATTCATATATTCTTCTTACATATTCATGGTAGCACTAATCCTTTAGGGTATGATACACCTCTAAAAATACCATTCTATCCAAATCTATTAACTTTAGATATTAAAGGATTTAACTATGTATTAGTTATATTCTTATTCCAAAGTTTATTTGGAAT | 443 | - |
| *Leucocytozoon* | CATMIN07 |  | Partial | 1 | HYLMUS02 | TATCTTCATATCTTAAGAGGATTAAACTATTCTTTCTCTTACTTACCTTTATCATGGTATAGTGGTTTAATAATATTCTTAATTTTTATTGTAACTGCTTTCATGGGTTACGTCTTACCATGGGGACAAATGAGTTTCTGGGGAGCAACTGTAATTACTAATTTATTATATTTTATTCCTGGATTAATTAATTGGGTATGTGGTGGATTTATTATTAATGACCCAACACTAAAAAGATTCTTCGTATTACACTTCATATTCCCATTTATAGCATTAGCTATTGTATTTATTCATATATTCTTCTTACATATTCATGGTAGCACTAATCCTTTAGGGTATGATACACCTTTAAAAATACCATTCTATCCAAATCTATTAACTTTAGATATTAAAGGATTCAACTATGTATTAGTTATATTCTTATTTCAAAGTTTATTTGGAAT | 443 | - |
| *Leucocytozoon* | CATMIN08 |  | Partial | 1 | CATFUS18 | TATCTTCATATCTTAAGAGGATTAAACTATTCTTTCTCTTACTTACCTTTATCATGGTATAGTGGTTTAATAATATTCTTAATTTTTATTGTAACTGCTTTCATGGGTTACGTCTTACCATGGGGACAAATGAGTTTCTGGGGAGCAACTGTAATTACTAATTTATTATATTTTATTCCTGGATTAATTAATTGGGTATGTGGTGGATTTATTATTAATGACCCAACACTAAAAAGATTTTTCGTATTACACTTCATATTCCCATTTATAGCATTAGCTATTGTATTTATTCATATATTCTTCTTACATATTCATGGTAGCACTAATCCTTTAGGGTATGATACACCTTTAAAAATACCATTCTATCCAAATCTATTAACTTTAGATATTAAAGGATTCAACTATGTATTAGTTATATTCTTATTTCAAAGTTTATTTGGAAT | 443 | - |
| *Plasmodium* | CATUST01 | EF011190 | Partial | 1 | CATUST06 | GCAACAGGTGCATCATTTGTCTTTATTCTTACATATTTACATATATTAAGAGGACTAAATTACTCTTATTCTTATTTACCTCTATCATGGATATCAGGATTAATAATATTTTTAATATCAATAGTAACAGCTTTTATGGGATATGTATTACCTTGGGGACAAATGAGTTTCTGGGGTGCAACAGTTATTACAAACTTATTATATTTTATACCTGGTCTTGTTTCATGGATATGTGGTGGATATCTTGTAAGTGACCCAACATTAAAAAGATTTTTTGTTTTACATTTTATATTTCCATTTATAGCTTTATGTATTGTATTTATACATATATTCTTTTTACATTTACAAGGTAGCACAAATCCTTTAGG | 368 | North_America |
| *Plasmodium* | CET01 | HQ262949 | Full | 1 | COLL13 | GCTTCATTTGTATTTATTTTAACTTATTTACATATTTTAAGAGGATTAAATTATTCATATTCATATTTACCTTTATCATGGATATCTGGATTAATAATATTTTTAATATCTATAGTAACAGCTTTTATGGGTTACGTATTACCTTGGGGTCAAATGAGTTTCTGGGGTGCTACCGTAATAACTAATTTATTATATTTTATACCTGGACTAGTTTCATGGATATGTGGTGGATATCTTGTAAGTGACCCAACCTTAAAAAGATTCTTTGTACTACATTTTACATTTCCTTTTATAGCTTTATGTATTGTATTCATACATATATTCTTTTTACATTTACAAGGTAGCACAAATCCTTTAGGGTATGATACAGCTTTAAAAATACCCTTCTATCCAAATCTTTTAAGTCTTGATATTAAAGGATTTAATAATGTATTAGTACTATTTTTAGCACAAAGTTTATTTGGAATACT | 470 | Europe |
| *Haemoproteus* | CHLOP01 | JQ764618 | Full | 1 | ZOCAP14 | TTTGTTTTTATTCTAACTTACTTACATATCTTAAGAGGATTAAATTATTCATATTCTTATTTACCTTTATCATGGATAACTGGATTAGTTATATTCTTAATTTCAATTGTTACCGCTTTTATGGGTTATGTATTACCTTGGGGTCAAATGAGTTTCTGGGGTGCAACCGTTATAACTAATTTATTATATTTTATTCCTGGACTTGTTTCATGGATTTGTGGAGGATATACTATAAGTGATCCAACTTTAAAAAGATTTTTTGTATTACATTTTATATTTCCTTTTATAGCTTTATGTATTGTATTTATACATATATTCTTCTTACACTTACAAGGTAGCTCTAATCCTTTAGGATATGATACAGCTTTAAAAATACCTTTCTATCCAAGTCTATTATGTCTAGATATCAAAGGATTTAATAATGTATTAGTCCTATTTCTAGCACAAAGTTTATTTGGAATTCT | 464 | South_America |
| *Haemoproteus* | CINSOV01 | MF442570 | Full | 1 | NENOT04 | GCTACTGGTGCTACATTTGTATTTATTTTAACTTACTTACATATATTAAGAGGATTAAACTATTCATACTCTTATTTACCTTTATCATGGATAACAGGATTACTAATATTCTTAATTTCTATTGTTACCGCTTTTATGGGTTATGTATTACCTTGGGGTCAAATGAGTTTCTGGGGTGCAACCGTTATTACTAATTTATTATATTTTATACCTGGACTTGTATCATGGATTTGTGGAGGATATACTATTAGTGATCCAACTCTAAAAAGATTTTTTGTATTACATTTTATATTTCCTTTTATAGCTTTATGTATTGTATTTATACATATATTCTTTTTACACTTACAAGGTAGCTCTAATCCTTTAGGATATGATACAGCTTTAAAAATACCTTTCTATCCAAGTCTATTATGTCTAGATATTAAAGGATTTAATAATGTATTAGTCCTATTTCTAGCACAAAGTTTATTTGGAATTCT | 479 | South_Sahara |
| *Haemoproteus* | COCOR01 | DQ451411 | Partial | 1 | CXPIP27 | GCAACTGGTGCTACATTTGTCTTTATCTTAACTTATCTACATATACTAAGAGGATTAAATTATTCATATTCATATTTACCTTTATCATGGACAACTGGAATATTAATTTTCTTAATTTCTATTGTCACTGCTTTTATGGGTTATGTATTACCTTGGGGTCAAATGAGTTTCTGGGGTGCAACCGTTATAACTAATTTATTATATTTTATACCTGGACTTGTTTCATGGATTTGTGGTGGATATATAATTAGTGATCCAACTTTAAAAAGATTTTTTGTATTACATTTTATATTTCCATTTATAGCTTTATGTATTGTATTTATACATATATTCTTTTTACACTTACAAGGTAGCTCTAATC | 361 | North_Africa_._Middle_East |
| *Leucocytozoon* | COCOR03 | JX867112 | Full | 1 | CORMAC03 | TCAACAGGTGCATCTTTTGTATTTATATTAACATATCTACATATCTTAAGAGGATTAAATTATTCTTTCTCTTACTTACCTTTATCATGGTATAGTGGTTTAATCATATTCTTAATCTTTATTGTAACTGCTTTTATGGGTTACGTCTTACCATGGGGACAAATGAGTTTCTGGGGAGCAACTGTAATTACTAACTTATTATATTTCATTCCTGGATTAATTAATTGGGTCTGTGGTGGATTTATTATTAATGACCCAACACTAAAAAGATTCTTCGTATTACATTTTATATTCCCATTTATAGCTTTAGCTATTGTATTTATTCATATATTCTTCTTACATATTCATGGTAGCACTAATCCTTTAGGGTATGATACACCTTTAAAAATACCATTCTATCCAAATCTATTAACCTTAGATGTTAAAGGATTTAACTATGTACTAGTTATATTTTTATTTCAAAGTTTATTTGGAATT | 477 | Europe |
| *Leucocytozoon* | COLPAS08 |  | Full | 1 | TURMIG11 | TCAACAGGTGCATCTTTTGTATTTATATTAACATATTTACACATATTAAGAGGATTAAATTATTCTTTCTCTTACTTACCTTTATCATGGATAAGTGGTTTAATTATATTTTTAATATTTATTGTAACTGCTTTTATGGGTTATGTCTTGCCATGGGGTCAAATGAGTTTCTGGGGAGCTACTGTAATTACTAATCTATTATATTTTATCCCTGGACTAATTAATTGGGTTTGTGGAGGATTTATTATTAATGACCCAACTCTAAAAAGATTCTTTGTATTACATTTTATATTCCCATTTATAGCATTAGCAATCGTATTTATACATATATTCTTCTTACATATTCAAGGTAGCACTAATCCTTTAGGGTATGATACACCTTTAAAAATACCATTCTATCCAAATCTATTAACTTTAGATGTTAAAGGATTTAATTATGTAATAGTATTATTCTTATTTCAAAGTTTATTTGGAATTGC | 479 | - |
| *Haemoproteus* | COLPIC01 | KU258521 | Full | 1 | COPIC01 | ATATTCTAAGAGGTTTGAATTACTCTTATTCATATTTACCATTATCATGGATTACCGGATTAATAATATTTTTAATCTCTATTGTAACTGCTTTTATGGGTTACGTATTACCTTGGGGTCAAATGAGTTTCTGGGGTGCAACTGTTATTACTAATTTACTTTATTTTATTCCTGGATTAGTCTCATGGATTTGTGGTGGATATATTGTTAGTGACCCAACCCTAAAAAGATTTTTTGTATTACATTTTATATTTCCATTTATAGCTATATGTATAGTATTTATACATATATTCTTTCTACATTTACAAGGTAGCTCTAATCCTTTAGGATATGATACAGCTTTAAAAATACCCTTCTATCCAAGTCTATTATGCCTAGATATTAAAGGTTTTAATAACGTATTAGTTTTATTCTTAGCTCAAAGTTTATTTGGAATATT | 439 | South_America |
| *Haemoproteus* | COLSQU01 | FJ462662 | Partial | 1 | COSQU04 | GCAACTGGTGCATCTTTTGTATTTATTTTAACATACTTACATATTCTAAGAGGATTGAATTACTCCTATTCATATTTACCATTATCATGGATTACTGGATTAATAATATTTCTAATCTCTATTGTAACTGCTTTTATGGGTTACGTATTACCTTGGGGTCAAATGAGTTTCTGGGGTGCAACAGTTATTACTAATTTACTTTATTTTATTCCTGGATTAGTCTCATGGATTTGTGGTGGATATATTGTTAGTGACCCTACCCTAAAAAGATTCTTTGTATTACATTTTATATTTCCTTTTATAGCTATATGTATAGTATTTATACATATATTCTTTCTACATTTACA | 347 | South_America |
| *Leucocytozoon* | CORMAC01 | AB183553 | Partial | 1 | COCOR16 | TCAACAGGTGCATCTTTTGTATTTATATTAACATACCTACATATTTTAAGAGGATTAAATTACTCTTTCTCTTACTTACCTTTATCATGGTATAGTGGTTTAATTATATTCTTAATCTTTATTGTAACTGCTTTTATGGGTTACGTTTTACCATGGGGACAAATGAGTTTCTGGGGAGCAACTGTAATTACTAATTTATTATATTTCATACCTGGACTAATTAATTGGGTCTGTGGTGGATTTATTATTAATGACCCAACACTAAAAAGATTCTTTGTATTACATTTTATATTCCCATTTGTAGC | 305 | Asia |
| *Leucocytozoon* | CORMAC03 | AB741500 | Full | 1 | COCOR03 | TCAACAGGTGCATCTTTTGTATTTATATTAACATATCTACATATCTTAAGAGGATTAAATTATTCTTTCTCTTACTTACCTTTATCATGGTATAGTGGTTTAATCATATTCTTAATCTTTATTGTAACTGCTTTTATGGGTTACGTCTTACCATGGGGACAAATGAGTTTCTGGGGAGCAACTGTAATTACTAACTTATTATATTTCATTCCTGGATTAATTAATTGGGTCTGTGGTGGATTTATTATTAATGACCCAACACTAAAAAGATTCTTCGTATTACATTTTATATTCCCATTTATAGCTTTAGCTATTGTATTTATTCATATATTCTTCTTACATATTCATGGTAGCACTAATCCTTTAGGGTATGATACACCTTTAAAAATACCATTCTATCCAAATCTATTAACCTTAGATGTTAAAGGATTTAACTATGTACTAGTTATATTTTTATTTCAAAGTTTATTTGGAATTGC | 479 | Europe, Asia |
| *Plasmodium* | CXPIP21 | JF411402 | Full | 1 | CXTHE01 | GCAAAAGGAGCTTCATTTGTATTTATCTTAACTTACTTACACATTTTAAGAGGATTAAATTATTCATATTCATACTTACCTTTATCATGGATATCAGGATTAATGATATTTTTAATATCAATAGTTACAGCTTTTATGGGTTATGTATTACCTTGGGGTCAAATGAGTTTCTGGGGTGCAACTGTTATCACTAATTTATTATATTTTATCCCTGGACTTGTTTCATGGATTTGTGGTGGATATCTTGTAAGTGACCCAACTTTAAAAAGATTCTTTGTATTACATTTTACATTTCCATTTATAGCTTTATGTATTGTATTTATACATATATTCTTTCTACATTTACAAGGTAGCACTAATCCTTTAGGGTATGATACAGCTTTAAAAATACCCTTCTATCCAAATCTATTAAGTCTCGACATAAAAGGATTTAATAATGTATTAGTCTTATTTTTAGCACAAAGCTTATTTGGAATCTT | 479 | North_Africa_._Middle_East |
| *Haemoproteus* | CYACAE08 | MZ571103 | Full | 1 | CYACAE09 | CTACTGGTGCTACATTTGTCTTTATTTTAACTTATTTACATATATTAAGAGGATTAAATTATTCATATTCATATTTACCTTTATCATGGATATCTGGATTAATAATATTCTTAATCTCTATTGTTACTGCTTTTATGGGTTATGTATTACCTTGGGGTCAAATGAGTTTCTGGGGTGCAACCGTTATTACTAATTTATTATATTTTATACCTGGACTTGTTTCATGGATTTGTGGAGGATATACTATTAGTGATCCAACTTTAAAAAGATTTTTTGTATTGCATTTTATATTCCCTTTTATAGCCCTATGTATTGTGTTTATACATATATTTTTCTTACACTTACAAGGTAGCTCTAATCCTTTAGGATATGATACAGCTTTAAAAATACCTTTCTATCCAAGTCTATTATGTCTAGATATTAAAGGATTTAATAATGTATTAGTCTTATTTCTAGCACAAAGTTTATTTGGAATATT | 478 | Europe |
| *Haemoproteus* | CYACAE09 | MZ571102 | Full | 1 | CYACAE08 | CTACTGGTGCTACATTTGTCTTTATTTTAACTTATTTACATATATTAAGAGGATTAAATTATTCATATTCATATTTACCTTTATCATGGATATCTGGATTAATAATATTCTTAATCTCTATTGTTACTGCTTTTATGGGTTATGTATTACCTTGGGGTCAAATGAGTTTCTGGGGTGCAACCGTTATTACTAATTTATTATATTTTATACCTGGACTTGTTTCATGGATTTGTGGAGGATATACTATTAGTGATCCAACTTTAAAAAGATTTTTTGTATTGCATTTTATATTCCCTTTTATAGCCCTATGTATTGTGTTTATACATATATTTTTCTTACACTTACAAGGTAGCTCTAATCCTTTAGGATATGATACAGCTTTAAAAATACCTTTCTATCCAAGTCTATTATGTCTAGATATTAAAGGATTTAATAATGTATTAGTCTTATTTCTAGCACAAAGTTTATTTGGAATATT | 478 | Europe |
| *Plasmodium* | DENPEN01 | EF011174 | Partial | 1 | KEWA01 | GCAACAGGTGCATCATTTGTATTTATTCTTACTTATCTACATATTTTAAGAGGATTAAATTATTCTTATTCTTATTTACCTTTATCATGGATATCAGGATTAATAATATTCTTAATATCAATAGTAACTGCTTTTATGGGATATGTATTACCTTGGGGTCAAATGAGTTTCTGGGGTGCAACTGTCATTACTAATTTATTATATTTTATACCTGGTCTTGTTTCATGGATTTGTGGTGGATATCTTGTAAGCGACCCAACATTAAAAAGATTTTTTGTATTACATTTTATATTTCCATTTATAGCCTTATGTATTGTATTTATACATATATTCTTTCTACATTTACAAGGTAGCACAAATCCTTTAGG | 368 | North_America |
| *Haemoproteus* | DICBAL02 | JX418183 | Full | 1 | DICBAL06 | TTTGTTTTTATTTTAACTTACTTACATATTCTAAGAGGATTAAATTATTCATATTCATATTTACCTTTATCATGGATAACTGGATTAATAATATTCCTAATTTCAATTGTTACAGCTTTTATGGGTTATGTATTACCTTGGGGTCAAATGAGTTTCTGGGGTGCAACCGTTATTACTAATTTATTATACTTTATACCTGGACTTGTTTCATGGATTTGTGGAGGATATACAATTAGTGATCCAACTTTAAAAAGATTTTTTGTATTACATTTTATATTCCCATTTATAGCCCTATGTATTGTATTTATACATATTTTCTTCTTACATTTACAAGGTAGCTCTAATCCTTTAGGATATGATACAGCTTTAAAAATACCTTTCTATCCAAGTCTATTATGTCTAGATATTAAAGGATTTAATAATGTATTAGTCCTATTTCTAGCACAAAGTTTATTTGGTATTTT | 464 | Asia |
| *Haemoproteus* | DICBAL06 | JX418195 | Full | 1 | DICBAL02 | TTTGTTTTTATTTTAACTTACTTACATATTCTAAGAGGATTAAATTATTCATATTCATATTTACCTTTATCATGGATAACTGGATTAATAATATTCCTAATTTCAATTGTTACAGCTTTTATGGGTTATGTATTACCTTGGGGTCAAATGAGTTTCTGGGGTGCAACCGTTATTACTAATTTATTATACTTTATACCTGGACTTGTTTCATGGATTTGTGGAGGATATACAATTAGTGATCCAACTTTAAAAAGATTTTTTGTATTACATTTTATATTCCCATTTATAGCCCTATGTATTGTATTTATACATATTTTCTTCTTACATTTACAAGGTAGCTCTAATCCTTTAGGATATGATACAGCTTTAAAAATACCTTTCTATCCAAGTCTATTATGTCTAGATATTAAAGGATTTAATAATGTATTAGTCCTATTTCTAGCACAAAGTTTATTTGG | 458 | Asia |
| *Haemoproteus* | DICLEU01 | EF380170 | Partial | 1 | DICADS01 | GGTGCAACCGTTATAACTAATTTATTATATTTTATTCCTGGACTTGTTTCATGGATTTGTGGTGGATATATAATTAGTGATCCAACTTTAAAAAGATTTTTTGTATTACATTTTATATTTCCATTTATAGCTTTATGTATTGTATTTATACATATATTCTTTTTACACTTACAAGGTAGCTCTAATCCTTTAGGATATGATACTGCTTTAAAAATACCTTTCTATCCAAGTCTATTATGTCTAGATATAAAAGGATTTAATAATGTATTAGTCTTATTTCTAGCACAAAGTTTATTTGGAATTTT | 305 | Asia |
| *Haemoproteus* | DIGCYA01 | KJ661265 | Full | 1 | DIGCAE01 | CATTTGTTTTTATTCTAACTTACTTACATATCTTAAGAGGATTAAATTATTCATATTCTTATTTACCTTTATCATGGATAACTGGATTAGTTATATTCTTAATTTCAATTGTTACCGCTTTTATGGGTTATGTATTACCTTGGGGTCAAATGAGTTTCTGGGGTGCAACCGTTATAACTAATTTATTATATTTTATTCCTGGACTTGTTTCATGGATTTGTGGAGGATATACTATTAGTGATCCAACTTTAAAAAGATTTTTTGTATTACATTTTATATTTCCTTTTATAGCTTTATGTATTGTATTTATACATATATTCTTCTTACACTTACAAGGTAGCTCTAATCCTTTAGGATATGATACAGCTTTAAAAATACCTTTCTATCCAAGTCTATTATGTCTAGATATCAAAGGATTTAATAATGTATTAGTCCTATTTCTAGCACAAAGTTTAT | 456 | South_America |
| *Haemoproteus* | DIGCYA03 | KJ661274 | Full | 1 | HEMATR01 | CATTTGTTTTTATTCTAACTTACTTACATATCTTAAGAGGATTAAATTATTCATATTCTTATTTACCTTTATCATGGATAACTGGGTTAGTTATATTCTTAATATCAATTGTTACCGCTTTTATGGGTTATGTATTACCTTGGGGTCAAATGAGTTTCTGGGGTGCAACCGTTATAACTAATTTATTATATTTTATTCCTGGACTTGTTTCATGGATTTGTGGAGGATATACTATTAGTGATCCAACTTTAAAAAGATTTTTTGTATTACATTTTATATTTCCTTTTATAGCTTTATGTATTGTATTTATACATATATTCTTCTTACACTTACAAGGTAGCTCTAATCCTTTAGGATATGATACAGCTTTAAAAATACCTTTCTATCCAAGTCTATTATGTCTAGATATCAAAGGATTTAATAATGTATTAGTCCTATTTCTAGCACAAAGTTTAT | 456 | South_America |
| *Leucocytozoon* | DIGCYA05 | KJ661320 | Full | 1 | HEMATR03 | CTGTTGTCTTTATATTAACATATTTACATATTCTAAGAGGTTTAAATTACTCTTTCTCTTACTTACCTTTATCATGGACAAGTGGTTTAATAATATTCTTAATATTTATTGTTACTGCGTTTATGGGTTATGTCTTACCATGGGGTCAAATGAGTTTCTGGGGAGCTACTGTAATTACAAATTTATTATATTTTATTCCTGGATTAATAAATTGGGTTTGTGGTGGTTTTATAATTAACGACCCAACTCTAAAAAGATTCTTTGTATTACATTTTATATTCCCATTCGTAGCACTAGCAATGGTATTTATTCATATATTCTTCTTACATATTCAAGGTAGCACTAATCCTTTAGGGTATGATACACCTTTAAAAATACCATTCTATCCAAATTTATTAACTCTAGATGTTAAAGGATTTAATTATGTATTAGTAATCTTCTTATTTCAAAGTTTAT | 456 | South_America |
| *Plasmodium* | DOLFRI01 | DQ241508 | Partial | 1 | LEPCOR05 | GCAACAGGTGCTTCATTTGTATTTATTTTAACTTATTTACATATTTTAAGAGGATTAAATTATTCATATTCATATTTACCTTTATCATGGATATCTGGACTAATTATATTTTTAATATCTATTGTAACAGCTTTTATGGGTTATGTATTACCTTGGGGTCAAATGAGTTTCTGGGGTGCTACAGTTATAACTAATTTATTATATTTTATACCTGGACTTGTTTCATGGATATGTGGTGGATATCTTGTAAGTGACCCAACCTTAAAAAGATTCTTTGTATTACATTTTACATTTCCATTTATAGCCTTATGTATTGTATTTATACATATATTCTTTCTACATTTACAAGGTAGCACAAAT | 360 | South_America |
| *Plasmodium* | DONANA03 | JX458328 | Full | 1 | CXTHE01 | CAACGGGAGCTTCATTTGTATTTATCTTAACTTACTTACACATTTTAAGAGGATTAAATTATTCATATTCATACTTACCTTTATCATGGATATCAGGATTAATGATATTTTTAATATCAATAGTTACAGCTTTTATGGGTTATGTATTACCTTGGGGTCAAATGAGTTTCTGGGGTGCAACTGTTATCACTAATTTATTATATTTTATCCCTGGACTTGTTTCATGGATTTGTGGTGGATATCTTGTAAGTGACCCAACTTTAAAAAGATTCTTTGTATTACATTTTACATTTCCATTTATAGCTTTATGTATTGTATTTATACATATATTCTTTCTACATTTACAAGGTAGCACTAATCCTTTAGGGTATGATACAGCTTTAAAAATACCCTTCTATCCAAATCTATTAAGTCTCGACATAAAAGGATTTAATAATGTATTAGTCTTATTTTTAGCACAAAGCTTATTTGGAATCTT | 478 | Europe |
| *Haemoproteus* | DUMCAR10 | MW081137 | Partial | 1 | DUMCAR08 | GCTACATTTGTATTTATTCTAACTTATTTACATATCTTAAGAGGATTAAATTACTCATATTCATATTTACCACTATCATGGATAACAGGATTGGTAATATTCTTAATTTCTATTGTTACTGCTTTTATGGGTTATGTATTACCTTGGGGTCAAATGAGTTTCTGGGGTGCAACCGTTATTACTAATTTATTATATTTTATACCTGGACTTGTTTCATGGATTTGTGGAGGATATACTATAAGTGATCCAACTTTAAAAAGATTTTTTGTATTACACTTTATATTTCCTTTTATAGCCTTATGTATTGTATTTATACATATATTTTTCTTACACTTACAAGGTAGCTCTAATCCTTTAGGATATGATACAGCTTTAAAAATACCTTTCTATCCAAGTCTATTATGTCTAGATAT | 413 | North_America |
| *Haemoproteus* | ELALB01 | EF153647 | Full | 1 | MYISWA01 | TTTGTTTTTATTTTAACATATTTACATATTTTAAGAGGTTTAAATTATTCATATTCATATTTACCTTTATCATGGATATCTGGATTAATTATATTTTTAATTTCTATAGTTACTGCATTTATGGGTTATGTATTACCTTGGGGTCAAATGAGTTTCTGGGGTGCAACCGTTATAACTAATTTATTATATTTTATACCTGGACTTGTTTCATGGATTTGCGGTGGATATACAATTAGTGATCCAACTTTAAAAAGATTCTTTGTATTACATTTTATATTTCCATTTATAGCTTTATGTATTGTATTTATACATATATTTTTCTTACATTTACAAGGTAGCTCTAATCCTTTAGGATATGATACAGCTTTAAAAATACCTTTCTATCCAAGTCTATTATGTCTAGATATTAAAGGATTTAATAATGTATTAGTCCTATTTCTAGCACAAAGTTTATTTGGAATTTT | 464 | South_America |
| *Plasmodium* | ELALB02 | EF153641 | Full | 1 | CURCUR01 | TTGTATTTATTCTTACATATTTACATATTTTAAGAGGATTAAATTATTCTTATTCTTATTTACCTTTATCATGGATATCAGGATTAATAATATTTTTAATATCAATAGTTACTGCTTTTATGGGATATGTACTACCTTGGGGTCAAATGAGTTTCTGGGGTGCAACCGTCATTACTAATTTACTATATTTTATACCTGGTCTTGTTTCATGGATCTGTGGTGGATATCTTGTAAGCGACCCAACATTAAAAAGATTTTTTGTATTACATTTTATATTTCCATTTATAGCCTTATGTATTGTATTTATACATATATTCTTTCTACATTTACAAGGTAGCACTAATCCTTTAGGGTATGATACAGCTTTAAAAATACCCTTCTATCCAAATCTATTAAGTCTTGATATTAAAGGATTTAATAATATCTTAGTTTTATTCTTAGCACAAAGTTTATTTGGAATATT | 463 | South_America |
| *Haemoproteus* | EMGOD01 | KT757553 | Full | 1 | DENVID02 | GCAACAGGTGCTTCATTTGTATTTATTTTAACTTACTTACATATTTTAAGAGGATTAAATTATTCATATTCATATTTACCATTATCATGGATTTCAGGATTAATAATATTCTTAATATCTATAGTAACTGCTTTTATGGGTTATGTATTACCTTGGGGTCAAATGAGTTTCTGGGGTGCTACAGTTATTACTAATTTATTATATTTTATTCCAGGACTTGTATCATGGATTTGTGGTGGATATCTTGTTAGTGACCCAACATTAAAAAGATTCTTTGTATTACACTTTACATTCCCATTTATAGCTTTATGTATTGTATTTATACATATATTCTTTTTACATTTACAAGGTAGCACTAATCCTTTAGGGTATGATACAGCTTTAAAAATACCCTTCTATCCAAATCTATTAAGCCTTGATATTAAAGGATTT | 432 | Asia |
| *Plasmodium* | EMHOR1 | DQ451406 | Partial | 1 | AEDVEX01 | GCAACAGGTGCTTCATTTGTATTTATTTTAACTTATCTACATATTTTAAGAGGATTAAATTATTCATATTCATACTTACCTTTATCATGGATATCTGGATTATTAATATTTTTAATATCTATAGTAACAGCTTTTATGGGTTATGTATTACCTTGGGGTCAAATGAGTTTCTGGGGTGCTACAGTAATTACTAATTTATTATATTTTATACCTGGACTTGTTTCATGGATATGTGGTGGATATCTTGTAAGTGACCCAACATTAAAAAGATTTTTTGTATTACACTTTACATTTCCATTTATAGCTTTATGTATTGTATTTATACATATATTCTTTTTACATTTACAAGGTAGCACAAATC | 361 | North_Africa_._Middle_East |
| *Plasmodium* | EUVIO01 | JX021469 | Full | 1 | TABI08 | CAACAGGTGCTTCATTTGTATTTATTTTAACTTATTTACATATTTTAAGAGGATTAAATTATTCATATTCATATTTACCTTTATCATGGATATCTGGACTACTTATATTTTTAATATCTATTGTAACAGCTTTTATGGGTTATGTATTACCTTGGGGTCAAATGAGTTTCTGGGGTGCTACAGTTATAACTAATTTATTATATTTTATACCTGGACTTGTTTCATGGATATGTGGTGGATATCTTGTAAGTGACCCAACCTTAAAAAGATTCTTTGTATTACATTTTACATTTCCATTTATAGCCTTATGTATTGTATTTATACATATATTCTTTTTACATTTACAAGGTAGCACAAATCCTTTAGGGTATGATACAGCTTTAAAAATACCCTTCTATCCAAATCTTTTAAGTCTTGATATTAAAGGATTTAATAATGTATTAGTATTATTCTTAGCACAAAGTTTATTTGGAATATT | 478 | South_America |
| *Haemoproteus* | FASPA01 | EU254556 | Partial | 1 | BNOW03 | GCTACTGGCGCTACATTTGTATTCATTTTAACTTATCTACATATTCTAAGAGGATTAAATTATTCATATGTATATTTACCTTTATCATGGATAACTGGATTAATTATATTCTTAATCTCTATAGTTACTGCTTTTATGGGTTATGTTCTACCTTGGGGTCAAATGAGTTTCTGGGGTGCAACAGTTATTACTAATTTATTATACTTCATACCTGGACTAGTATCATGGATTTGTGGTGGATATACTATTAGTGACCCTACTTTAAAAAGATTCTTTGTATTACACTTTATATTCCCATTTATAGCCTTATGTATCGTATTTATACATATATTCTTCTTACATCTACAAGGTAGCTCTAATC | 361 | North_America |
| *Plasmodium* | FOUOMI04 | MF442548 | Full | 1 | NESOU01 | GCAACTGGTGCTTCATTTGTATTTATTTTAACTTATTTACATATTTTAAGAGGATTAAATTATTCATATTCATACTTACCTTTATCATGGATATCTGGATTAATAATATTCTTAATATCTATAGTTACAGCTTTTATGGGTTATGTATTACCTTGGGGTCAAATGAGTTTCTGGGGTGCCACTGTAATTACTAATCTACTATATTTTATACCTGGACTTGTTTCATGGATTTGTGGTGGATATCTTGTAAGTGACCCAACATTAAAAAGATTCTTTGTATTACATTTTACATTTCCATTTATAGCTTTATGTATTGTATTCATACATATATTCTTTTTACATTTACAAGGTAGCACTAATCCTTTAGGGTATGATACAGCTTTAAAAATACCCTTCTATCCAAATCTTTTAAGTCTTGATATTAAAGGATTTAATAATATATTAGTATTATTTTTAGCACAAAGTTTATTTGGAATCTT | 479 | South_Sahara |
| *Leucocytozoon* | GALLUS34 | MW043770 | Full | 1 | BUTBUT05 | TCAACAGGTGCATCTTTTGTTTTTATATTAACATATTTACATATATTAAGAGGATTAAATTATTCTTTCTCTTATTTACCTTTATCATGGATTAGTGGTTTAGTAATATTCTTAATATTTATTGTAACTGCTTTTATGGGTTATGTCTTACCATGGGGACAAATGAGTTTCTGGGGAGCTACTGTTATAACTAATTTATTATATTTTATTCCTGGATTAATTAATTGGGTTTGTGGTGGATTTATTATTAATGACCCAACATTAAAAAGATTCTTTGTATTACACTTTATATTCCCATTTATAGCATTAGCAATTGTATTTATTCATATATTCTTTTTACATATTCAAGGTAGCACTAATCCTTTAGGGTATGATACACCTTTAAAAATACCATTCTATCCAAATCTATTAACTTTAGATATTAAAGGATTTAATTATGTACTAGTAATATTCTTATTTCAAAGTTTATTTGGAATTGC | 479 | Asia |
| *Haemoproteus* | GW5 | KJ396637 | Full | 1 | PHSIB2 | CTTATTTACATATATTAAGAGGACTAAATTATTCATATTCATATTTACCTTTATCATGGATATCTGGATTATTAATATTCTTAATTTCTATTGTTACTGCTTTTATGGGTTATGTATTACCTTGGGGTCAAATGAGTTTCTGGGGTGCAACCGTTATAACTAATTTATTATATTTTATACCTGGACTTGTTTCATGGATTTGTGGTGGATATATTATTAGTGATCCAACTTTAAAAAGATTTTTTGTATTACATTTTATATTCCCATTTATAGCTTTATGTATTGTATTTATACATATATTCTTTTTACACTTACAAGGTAGCTCTAATCCTTTAGGATATGATACTGCTTTAAAAATACCTTTCTATCCAAGTCTATTATGTCTAGATATTAAAGGATTTAATAATGTATTAGTCTTATTTCTAGCACAAAGTTTATTTGGAATATT | 448 | Asia |
| *Plasmodium* | HALVOC01 | EF011195 | Partial | 1 | CXPOI01 | GCAACAGGTGCATCATTTGTATTTATTCTTACCTATTTACATATTTTAAGAGGATTAAATTATTCTTATTCATATCTACCTTTATCTTGGATATCAGGATTAATAATATTTTTAATATCAATAGTAACTGCTTTTATGGGATATGTTTTACCTTGGGGACAAATGAGTTTTTGGGGTGCAACTGTTATAACTAACTTATTATATTTTATACCTGGTCTTGTTTCATGGATTTGTGGTGGATATCTTGTAAGCGACCCAACCTTAAAAAGATTTTTTGTATTACATTTTATATTTCCATTTATAGCTTTATGTATTGTATTTATACATATATTCTTTTTACATTTACAAGGTAGCACAAATCCTTTAGG | 368 | South_Sahara |
| *Haemoproteus* | JUNPHA04 |  | Full | 1 | TURASS05 | GCTACCGGTGCTACATTTGTTTTTATTCTAACTTACTTACATATTTTAAGAGGACTAAACTATTCATATTCTTATTTACCTTTATCATGGATTACTGGATTAATAATATTCTTAATTTCAATTGTTACCGCTTTTATGGGTTATGTATTACCTTGGGGTCAAATGAGTTTCTGGGGTGCAACCGTTATTACTAATTTATTATATTTTATACCTGGACTTGTTTCATGGATTTGTGGAGGATATACTATTAGTGATCCAACTTTAAAAAGATTCTTTGTATTACATTTTATATTCCCTTTTATAGCTTTATGTATTGTATTTATACATATATTCTTCTTACACTTACAAGGTAGCTCTAATCCTTTAGGATATGATACAGCTTTAAAAATACCTTTCTATCCAAGTCTATTATGTCTAGATATCAAAGGATTTAATAATGTATTAGTCCTATTTCTAGCACAAAGTTTATTTGGAATTCT | 479 | - |
| *Haemoproteus* | LARSCO01 | GQ404558 | Partial | 1 | BLUTI09 | ATTTATTATATTTTATACCTGGACTTGTTTCATGGATTTGTGGTGGATATACAATAAGTGATCCAACCTTAAAAAGATTTTTTGTATTACATTTTATATTTCCTTTTATAGCCTTATGTATTGTGTTTATTCATATATTCTTCTTACATTTACAAGGTAGCTCTAATCCTTTAGGATATGATACAGCTTTAAAAATACCTTTCTATCCAAGTCTATTATGTTTAGATATTAAAGGATTTAATAATGTATTAGTCATATTTTTAGCACAAAGTTTATTTGGTATTCT | 286 | South_America |
| *Haemoproteus* | LEPRUF01 | DQ241543 | Partial | 1 | TACCRI01 | GGTGCAACCGTTATAACTAATTTATTATATTTTATACCTGGACTTGTTTCATGGATTTGTGGAGGATATACTATTAGTGATCCAACTTTAAAAAGATTTTTTGTATTACATTTTATATTCCCTTTTATAGCTTTATGTATTGTATTTATACATATATTCTTCTTACACTTACAAGGTAGCTCTAATCCTTTAGGATATGATACAGCTTTAAAAATACCTTTCTATCCAAGTCTATTATGTCTAGATATCAAAGGATTTAATAATGTATTAGTCCTATTTCTAGCACAAAGTTTATTTGGAATTCT | 305 | South_America |
| *Plasmodium* | LEVER02 | FJ462684 | Partial | 1 | NYCNYC01 | GCAACAGGTGCTTCATTTGTTTTTATTTTAACTTACCTACATATTTTAAGAGGACTAAATTATTCATACTCATACTTACCATTATCATGGATATCTGGATTAATAATATTCTTAATATCTATAGTTACAGCTTTTATGGGTTATGTATTACCTTGGGGTCAAATGAGTTTCTGGGGTGCTACTGTAATAACTAATTTACTTTATTTTATTCCTGGACTTGTCTCATGGATTTGTGGTGGATATCTTGTAAGTGACCCAACCTTAAAAAGATTCTTTGTATTACATTTTACATTTCCATTTATAGCTTTATGTATTGTATTTATACATATCTTCTTTTTACATTTACA | 347 | South_America |
| *Haemoproteus* | LWT3 | MF565830 | Partial | 1 | SYCUR03 | GGTGCAACTGTTATTACTAATTTATTATATTTTATACCTGGATTAGTTTCATGGATTTGTGGGGGATATACTATAAGTGATCCAACTTTAAAAAGATTTTTTGTATTACATTTTATATTTCCATTTATAGCTTTATGTATTGTCTTTATTCATATATTCTTCTTACATCTACAAGGTAGCTCTAACCCTTTAGGATATGATACAGCTTTAAAAATACCTTTCTATCCAAGTCTATTATGTCTAGATATTAAAGGATTTAATAATGTATTAGTTATATTTTTAGCACAAAGTTTATTTGGAATTCT | 305 | Asia |
| *Haemoproteus* | MACFLA01 | AY714159 | Partial | 1 | PTIVIC02 | TTGTGGTGGATATATTATTAGTGATCCAACTTTAAAAAGATTTTTTGTATTACATTTTATATTTCCATTTATAGCTTTATGTATTGTGTTTATACATATATTCTTTTTACACTTACAAGGTAGCACTAATCCTTTAGGATATGATACAGCTTTAAAAATACCTTTCTATCCAAGTCTATTATGTCTAGATATAAAAGGATTTAATAATGTATTAGTCTTATTTCTAGCACAAAGTTTATTTGGAATATT | 249 | Australia_._New_Zeeland |
| *Plasmodium* | NECASP02 | AY714194 | Partial | 1 | MALCOR02 | TTGTGGTGGATATCTTGTAAGCGATCCAACTTTAAAAAGATTTTTTGTATTACATTTCACATTTCCTTTTATAGCTTTATGTATTGTATTTATACATATTTTCTTTCTACATTTACAAGGTAGCACAAATCCTTTAGGGTATGATACTGCTTTAAAAATACCCTTCTATCCAAATCTATTAAGTCTTGATATTAAAGGATTTAATAATATTTTAGTATTATTTTTAGCTCAAAGTTTATTTGGAATTTT | 249 | Oceania |
| *Haemoproteus* | NENOT04 | KX506755 | Full | 1 | CINSOV01 | TTTGTATTTATTTTAACTTACTTACATATATTAAGAGGATTAAACTATTCATACTCTTATTTACCTTTATCATGGATAACAGGATTACTAATATTCTTAATTTCTATTGTTACCGCTTTTATGGGTTATGTATTACCTTGGGGTCAAATGAGTTTCTGGGGTGCAACCGTTATTACTAATTTATTATATTTTATACCTGGACTTGTATCATGGATTTGTGGAGGATATACTATTAGTGATCCAACTCTAAAAAGATTTTTTGTATTACATTTTATATTTCCTTTTATAGCTTTATGTATTGTATTTATACATATATTCTTTTTACACTTACAAGGTAGCTCTAATCCTTTAGGATATGATACAGCTTTAAAAATACCTTTCTATCCAAGTCTATTATGTCTAGATATTAAAGGATTTAATAATGTATTAGTCCTATTTCTAGCACAAAGTTTATTTGGAATTCT | 464 | South_Sahara |
| *Plasmodium* | NESOU01 | KX506752 | Full | 1 | FOUOMI04 | TTGTATTTATTTTAACTTATTTACATATTTTAAGAGGATTAAATTATTCATATTCATACTTACCTTTATCATGGATATCTGGATTAATAATATTCTTAATATCTATAGTTACAGCTTTTATGGGTTATGTATTACCTTGGGGTCAAATGAGTTTCTGGGGTGCCACTGTAATTACTAATCTACTATATTTTATACCTGGACTTGTTTCATGGATTTGTGGTGGATATCTTGTAAGTGACCCAACATTAAAAAGATTCTTTGTATTACATTTTACATTTCCATTTATAGCTTTATGTATTGTATTCATACATATATTCTTTTTACATTTACAAGGTAGCACTAATCCTTTAGGGTATGATACAGCTTTAAAAATACCCTTCTATCCAAATCTTTTAAGTCTTGATATTAAAGGATTTAATAATATATTAGTATTATTTTTAGCACAAAGTTTATTTGGAATCTT | 463 | South_Sahara |
| *Plasmodium* | NEWAM01 | KX506750 | Full | 1 | NEWAM06 | TTTGTATTTATATTAACTTACTTACATATTTTAAGAGGATTAAATTATTCATACTCATACTTACCTTTATCATGGATTTCTGGATTAATGATATTTTTAATATCTATAGTTACAGCTTTTATGGGTTATGTATTACCTTGGGGTCAAATGAGTTTTTGGGGAGCAACAGTAATTACTAATTTATTATATTTTATACCTGGTCTTGTTTCATGGATTTGTGGTGGATATCTTGTAAGTGACCCAACTTTAAAAAGATTTTTTGTATTACATTTTACTTTTCCATTTATTGCTTTATGTATTGTATTCATACATATATTTTTCTTACATTTACAAGGTAGCACAAATCCTTTAGGGTATGATACAGCTTTAAAAATACCCTTCTATCCAAATCTTTTAAGTCTTGATATTAAAGGATTTAATAATGTATTAGTATTATTTCTAGCTCAAAGTTTATTTGGAATATT | 464 | South_Sahara |
| *Plasmodium* | NEWAM06 | MF442546 | Full | 1 | NEWAM01 | GCAACAGGTGCTTCATTTGTATTTATATTAACTTACTTACATATTTTAAGAGGATTAAATTATTCATACTCATACTTACCTTTATCATGGATTTCTGGATTAATGATATTTTTAATATCTATAGTTACAGCTTTTATGGGTTATGTATTACCTTGGGGTCAAATGAGTTTTTGGGGAGCAACAGTAATTACTAATTTATTATATTTTATACCTGGTCTTGTTTCATGGATTTGTGGTGGATATCTTGTAAGTGACCCAACTTTAAAAAGATTTTTTGTATTACATTTTACTTTTCCATTTATTGCTTTATGTATTGTATTCATACATATATTTTTCTTACATTTACAAGGTAGCACAAATCCTTTAGGGTATGATACAGCTTTAAAAATACCCTTCTATCCAAATCTTTTAAGTCTTGATATTAAAGGATTTAATAATGTATTAGTATTATTTCTAGCTCAAAGTTTATTTGGAATATT | 479 | South_Sahara |
| *Haemoproteus* | NEWBR01 | MF442588 | Full | 1 | NEWBR04 | GCTACAGGTGCTACATTTGTATTTATTTTAACATACTTACATATTTTAAGAGGATTAAATTATTCATATTCATATTTACCTTTATCATGGATAACAGGATTAATGATATTTTTAATTTCAATTGTTACCGCTTTTATGGGTTATGTATTACCTTGGGGTCAAATGAGTTTCTGGGGTGCAACCGTTATTACTAATTTATTATACTTTATACCTGGATTAGTTTCATGGATTTGTGGAGGATATACTATTAGTGATCCAACTTTAAAAAGATTCTTTGTATTACATTTTATATTCCCTTTTATAGCTTTATGTATCGTATTTATACATATATTTTTCTTACACTTACAAGGTAGCTCTAATCCTTTAGGATATGATACAGCTTTAAAAATACCTTTCTATCCAAGTCTATTATGTCTAGATATTAAAGGATTTAATAATGTATTAGTCCTATTTTTAGCACAAAGTTTATTTGGAATTCT | 479 | South_Sahara |
| *Haemoproteus* | NEWBR04 | MF442598 | Full | 1 | NEWBR01 | GCTACAGGTGCTACATTTGTATTTATTTTAACATACTTACATATTTTAAGAGGATTAAATTATTCATATTCATATTTACCTTTATCATGGATAACAGGATTAATGATATTTTTAATTTCAATTGTTACCGCTTTTATGGGTTATGTATTACCTTGGGGTCAAATGAGTTTCTGGGGTGCAACCGTTATTACTAATTTATTATACTTTATACCTGGATTAGTTTCATGGATTTGTGGAGGATATACTATTAGTGATCCAACTTTAAAAAGATTCTTTGTATTACATTTTATATTCCCTTTTATAGCTTTATGTATCGTATTTATACATATATTTTTCTTACACTTACAAGGTAGCTCTAATCCTTTAGGATATGATACAGCTTTAAAAATACCTTTCTATCCAAGTCTATTATGTCTAGATATTAAAGGATTTAATAATGTATTAGTCCTATTTTTAGCACAAAGTTTATTTGGAATTCT | 479 | South_Sahara |
| *Haemoproteus* | NINOX06 | LC230131 | Full | 1 | BUBBUB02 | ACATATTTACATATTTTAAGAGGATTAAATTATTCATATTCATATTTACCTTTATCATGGATAACTGGATTAATTATTTTTTTAATTTCTATTGTTACTGCTTTTATGGGTTATGTATTACCTTGGGGTCAAATGAGTTTCTGGGGTGCAACCGTTATTACTAACTTATTATATTTTATACCTGGACTTGTTTCATGGATTTGTGGTGGATATAATATTAGTGATCCTACTTTAAAAAGATTCTTTGTATTACATTTTATATTTCCATTTGTAGCTTTATGTATTGTATTTATACATATATTCTTTTTACACTTACAAGGTAGCTCTAATCCTTTAGGATATGATACAGCTTTAAAAATACCTTTCTATCCAAGTCTATTATGTTTAGATATTAAAGGATTTAGTAATATATTAGTATTATATTTAGCTCAAAGTTTATTTGGTATATT | 449 | Asia |
| *Leucocytozoon* | OTSCO02 | AB183555 | Partial | 1 | OTUSCO03 | TCAACAGGAGCATCTTTTGTATTTATATTAACATATCTACATATTCTAAGAGGATTAAATTATTCATTCTCCTACTTACCTTTATCATGGATAAGTGGTTTAGTTATCTTTTGTTTATTTATTGTAACTGCTTTTATGGGTTATGTCTTACCATGGGGACAAATGAGTTTCTGGGGAGCTACTGTTATTACTAATTTATTATACTTTATTCCTGGATTAATCAATTGGGTTTGCGGTGGATTTATTATTAATGATCCAACACTAAAAAGATTCTTCGTATTACATTTTATATTCCCATTTATAGC | 305 | Asia |
| *Plasmodium* | PADOM07 |  | Full | 1 | ALERUF02 | TTACTTACATATTTTAAGAGGATTAAATTATTCATATTCATATTTACCTTTATCATGGATATCTGGATTAATAATATTTTTAATATCTATAGTAACAGCTTTTATGGGTTACGTATTACCTTGGGGTCAAATGAGTTTCTGGGGTGCTACCGTAATAACTAATTTATTATATTTTATACCTGGACTAGTTTCATGGATATGTGGTGGATATCTTGTAAGTGACCCAACCTTAAAAAGATTCTTTGTACTACATTTTACATTTCCTTTTATAGCTTTATGTATTGTATTTATACATATATTCTTTCTACATTTACAAGGTAGCACAAATCCTTTAGGGTATGATACAGCTTTAAAAATACCCTTCTATCCAAATCTTTTAAGTCTTGATATTAAAGGATTTAATAATGTATTAGTACTATTTTTAGCACAAAGTTTATTTGGAATACT | 447 | - |
| *Leucocytozoon* | PARUS37 | JX855052 | Full | 1 | PICVIR01 | TAAATTATTCTTTTACTTACTTACCTTTATCATGGATAAGTGGTTTAGTAATATTCTTAATATTTATTGTAACTGCTTTTATGGGTTATGTCTTACCATGGGGTCAAATGAGTTTTTGGGGAGCTACTGTCATTACTAATTTATTATATTTTATTCCTGGACTAATTAATTGGGTTTGTGGTGGATTTATTATTAACGATCCAACTCTAAAAAGATTCTTCGTATTACATTTTATATTCCCATTTGTAGCACTAGCTATTGTATTTATACATATATTCTTCTTACATATTCAAGGTAGCACTAATCCTTTAGGGTATGATACACCTTTAAAAATACCATTCTATCCAAATCTATTAACTCTAGATGTTAAAGGATTAAACTATGTATTAGTA | 392 | Europe |
| *Haemoproteus* | PARUS64 | KT757568 | Full | 1 | EMGOD06 | TCAACAGGTGCATCTTTTGTATTTATATTAACATATCTACATATCTTAAGAGGATTAAATTATTCTTTCTCTTACTTACCTTTATCATGGTATAGTGGTTTAATTATATTCTTAATACTTATTGTAACTGCTTTTATGGGTTACGTTTTACCATGGGGACAAATGAGTTTCTGGGGAGCAACTGTAATTACTAACTTATTATATTTTATTCCTGGATTAATTAATTGGGTCTGTGGTGGATTTATTATTAATGACCCTACACTAAAAAGATTCTTCGTATTACATTTTATATTCCCATTTGTAGCCTTAGCTATTGTATTTATTCATATATTCTTCTTACATATTCATGGTAGCAATAATCCTTTAGGGTATGATACACCTTTAAAAATACCATTCTATCCAAATCTATTAACTTTAGATGTTAAAGGATTT | 432 | Asia |
| *Leucocytozoon* | PARUS90 | MN782321 | Full | 1 | PARUS93 | TTATATTAACATACTTACATATATTAAGAGGATTAAATTATTCTTTTACTTACTTACCATTATCATGGATAAGTGGTTTAGTAATATTCTTAATATTTATTGTAACTGCTTTTATGGGTTACGTATTACCATGGGGTCAAATGAGTTTTTGGGGAGCTACTGTTATAACAAATTTATTATACTTTATTCCTGGATTAATTAATTGGGTTTGTGGTGGATTCATTATTAATGACCCAACTTTAAAAAGATTCTTTGTATTACATTTTATATTCCCATTTGTAGCATTAGCTATTGTATTTATACATATATTTTTCTTACATATTCAAGGTAGCACTAATCCTTTAGGGTATGATACACCTTTAAAAATACCATTCTATCCAAATCTATTAACTTTAGATGTTAAAGGATTTAACTACGTATTAGTATTATTCCTATTTCAAAGTTTATTTGGAATTGC | 457 | Europe |
| *Haemoproteus* | PHICT03 | EU810757 | Full | 1 | ANLAT02 | GCTACTGGTGCTACATTTGTCTTTATTTTAACTTATTTACATATATTAAGAGGATTAAATTATTCATATTCATACTTACCTTTATCATGGATAACTGGATTACTAATATTCTTAATTTCTATTGTTACTGCTTTTATGGGTTATGTATTACCTTGGGGTCAAATGAGTTTCTGGGGTGCAACCGTTATAACTAATTTATTATATTTTATACCTGGACTTGTATCATGGATTTGTGGTGGATATATTATTAGTGATCCAACTTTAAAAAGATTTTTTATATTACATTTTATTTTCCCATTTATAGCTTTATGTATTGTATTTATACATATATTCTTTTTACATTTACAAGGTAGCTCTAATCCTTTAGGATATGATACTGCTTTAAAAATACCTTTCTATCCAAGTCTATTATGTCTAGATATTAAAGGATTTAATAATGTATTAGTCTTATTTCTAGCACAAAGTTTATTTGGAATTTT | 479 | South_Sahara |
| *Haemoproteus* | PIPUB01 | EU254552 | Partial | 1 | MELSTR01 | GCTACTGGTGCTACATTTGTTTTTATATTAACATATTTACATATTTTAAGAGGATTAAATTATTCATATTCATATTTACCTTTATCATGGATAACTGGATTAATGATTTACTTAATTTCTATTGTTACTGCTTTTATGGGTTATGTATTACCTTGGGGTCAAATGAGTTTCTGGGGTGCAACTGTTATTACTAACTTATTATATTTTATACCTGGACTTGTTTCATGGATTTGTGGTGGATATAATATTAGTGATCCTACATTAAAGAGATTCTTTGTATTACATTTTATATTTCCATTTATAGCTTTATGTATTGTATTTATACATATATTCTTCTTACACTTACAAGGTAGCTCTAATC | 361 | North_America |
| *Haemoproteus* | PTIPER02 | MG387220 | Full | 1 | PTIPER04 | GCTACTGGTGCTACATTTGTTTTTATTTTAACTTATTTACATATTTTAAGAGGATTAAATTATTCATATTCATATTTACCTTTATCATGGATAACTGGATTAATAATATTTTTAATTTCTATAGTTACTGCTTTTATGGGTTATGTATTACCTTGGGGTCAAATGAGTTTCTGGGGTGCAACCGTTATTACTAATTTATTATATTTTATACCTGGACTTGTTTCATGGATTTGTGGAGGATATACAATAAGTGATCCAACTTTAAAAAGATTTTTTGTATTACATTTTATATTCCCTTTTATAGCTTTATGTATTGTATTTATACATATATTCTTCTTACATTTACAAGGTAGCTCTAATCCTTTAGGATATGATACAGCTTTAAAAATACCTTTCTATCCAAGTCTATTATGTCTAGATATTAAAGGATTTAATAATGTATTA | 444 | Oceania |
| *Plasmodium* | PV5 | DQ508379 | Partial | 1 | STEGRA01 | GTGGATATCTCGTAAGTGACCCAACATTAAAAAGATTCTTTGTATTACACTTCATATTTCCATTCATAGCTTTATGTATTGTATTTATACATATATTCTTTCTACATTTACAAGGTAGCACAAATCCTTTAGGGTATGATACAGCTTTAAAAATACCCTTCTATCCAAATCTATTAAGTCTTGATATTAAAGGATTTAATAATATCCTAGTTTTATTTTTAGCACAAAGTTTATTTGGAATATT | 244 | South_Sahara |
| *Plasmodium* | PV8 | DQ508383 | Partial | 1 | STIERY01 | GTGGATATCTTGTAAGCGACCCAACATTAAAAAGATTCTTTGTATTACATTTTATATTTCCGTTTATAGCTTTATGTATTGTGTTTATACATATATTCTTTTTACATTTACAAGGTAGCACAAATCCTTTAGGGTATGATACAGCTTTAAAAATACCCTTCTATCCAAATCTATTAAGTCTTGATATTAAAGGATTTAATAATATTCTAGTTTTATTTTTAGCACAAAGTTTATTTGGAATATT | 244 | South_Sahara |
| *Plasmodium* | PYCCAF01 | EF380142 | Partial | 1 | IOLIND04 | GGTGCAACCGTTATTACTAATTTATTATATTTCATACCTGGTCTTGTTTCATGGATTTGCGGTGGATATCTTGTAAGCGATCCAACACTAAAAAGATTCTTCGTATTACATTTTATATTTCCATTTATAGCTTTGTGTATTGTGTTCATACATATATTCTTTCTACATTTACAAGGTAGCACAAATCCTTTAGGGTATGATACAGCTTTAAAAATACCCTTCTATCCAAATCTTTTAAGTCTCGATATCAAAGGATTTAATAATATCCTAGTTTTATTTTTAGCACAAAGTTTATTTGGAATATT | 305 | Asia |
| *Leucocytozoon* | RS4 | DQ847231 | Full | 1 | RECOB3 | CATCTTTTGTATTTATATTAACATATCTACATATATTAAGAGGATTAAACTATTCTTTCTCTTACTTACCTTTATCATGGTATAGTGGTTTAATTATATTCTTAATCTTTATTGTAACTGCTTTTATGGGTTACGTCTTACCATGGGGACAAATGAGTTTCTGGGGAGCAACTGTAATTACTAATTTATTATATTTTATTCCTGGATTAATTAATTGGGTCTGTGGTGGATTTATTATTAATGACCCAACATTAAAAAGATTCTTTGTATTACACTTTATATTCCCATTTGTAGCATTAGCTATTGTATTTATTCATATATTCTTTTTACATATTCATGGTAGCACTAATCCTTTAGGGTATGATACACCTTTAAAAATACCATTCTATCCAAATCTATTAACCTTAGATATTAAAGGATTTAACTATGTATTAGTTATATTTTTATTTCAAAGTTTATTTGGAATTGC | 469 | Europe, South_Sahara |
| *Haemoproteus* | RW3 | DQ368364 | Full | 1 | MW3 | GCTACATTTGTTTTTATTTTAACTTACTTACATATTTTAAGAGGATTAAATTACTCATATTCATATTTACCTTTATCATGGATATCTGGATTAATAATATTCTTAATCTCTATTGTTACTGCTTTTATGGGTTATGTTTTACCTTGGGGTCAAATGAGTTTCTGGGGTGCAACCGTTATTACTAATTTATTATATTTTATTCCTGGACTTGTATCATGGATTTGTGGTGGATATATTATTAGTGATCCAACACTAAAAAGATTCTTTGTATTACATTTTATATTTCCATTTATAGCTTTATGTATTGTTTTTATACATATATTCTTCTTACATTTACAAGGTAGCTCTAATCCTTTAGGATATGATACAGCTTTAAAAATACCTTTCTATCCAAGTCTATTATGTTTAGATATTAAAGGATTTAATAATGTATTAGTTATATTTTTAGCTCAAAGTTTATTTGGTATTCT | 470 | Europe |
| *Plasmodium* | SATOR01 | KX506753 | Full | 1 | SATOR02 | TTGTATTTATTCTTACTTATTTACACATTTTAAGAGGATTAAATTACTCTTACTCTTATTTACCTTTATCATGGATATCAGGATTATTAATATTTTTAATATCAATAGTAACTGCCTTTATGGGATATGTATTACCTTGGGGTCAAATGAGTTTCTGGGGTGCTACTGTTATTACCAATTTATTATACTTTATACCTGGTCTTGTTTCATGGATCTGTGGTGGATATCTTGTAAGTGACCCAACATTAAAAAGATTCTTTGTTTTACATTTTATATTTCCATTTATAGCTTTATGTATCGTATTTATACATATATTCTTTTTACATTTACAAGGTAGCACAAATCCTTTAGGGTATGATACAGCTTTAAAAATACCCTTCTATCCAAATCTATTAAGTCTTGATATTAAAGGATTTAATAATATATTTGTTTTATTTTTAGCACAAAGTCTATTTGGAATATT | 463 | South_Sahara |
| *Plasmodium* | SEINOV01 | EF011187 | Partial | 1 | GEOTRI09 | GCAACAGGCGCTTCATTTGTATTTATTTTAACTTACTTACATATTTTAAGAGGATTAAATTACTCATATTCATACTTACCTTTATCATGGATATCTGGTTTAATAATATTTTTAATATCAATAGTAACAGCTTTTATGGGTTATGTATTACCTTGGGGTCAAATGAGTTTCTGGGGTGCTACTGTAATTACTAATTTATTATATTTTATACCTGGACTTGTTTCATGGATTTGTGGTGGATATCTTGTTAGTGACCCAACATTAAAAAGATTCTTCGTATTACATTTTACATTTCCATTTATAGCTTTATGTATTGTATTTATACATATATTCTTCTTACATTTACAAGGTAGCACAAATCCTTTAGG | 368 | North_America |
| *Leucocytozoon* | SEPSEP01 | EF153659 | Full | 1 | APSPI12 | TATCTACATATATTAAGAGGATTAAACTATTCTTACACTTACTTACCATTATCATGGATAAGTGGTTTAGTTATATTTTTAATATTTATTGTAACTGCTTTTATGGGTTATGTCTTACCATGGGGTCAAATGAGTTTTTGGGGAGCTACTGTAATAACTAATTTATTATATTTTATTCCTGGATTAATCAATTGGGTTTGTGGTGGATTTATTATTAATGATCCAACACTAAAAAGATTCTTTATATTACATTTTATATTCCCATTCGTAGCACTTGCAATTGTATTTATTCATATATTCTTCTTACATATTCAAGGTAGCACTAATCCTTTAGGGTATGATACACCTTTAAAAATACCATTCTATCCAAATCTATTAACTTTAGATGTTAAAGGATTAAATTATGTATTAGTATTATTCTTATTTCAAAGTTTATTTGGAATTGC | 446 | South_America |
| *Leucocytozoon* | SETAUD27 | MF752692 | Full | 1 | SETCOR06 | CAACAGGTGCATCATTTGTCTTTATATTAACATACTTACATATATTAAGAGGATTAAATTATTCATTTACTTACTTACCTTTATCATGGATAAGTGGTTTAATAATATTCTTAATATTTATTGTAACTGCTTTTATGGGTTATGTCTTACCATGGGGTCAAATGAGTTTTTGGGGAGCTACTGTTATAACTAATTTATTATATTTTATTCCTGGATTAATTAATTGGGTTTGCGGTGGATTTATTATTAACGACCCAACTCTAAAAAGATTCTTCGTATTACATTTTATATTCCCATTTGTAGCATTAGCTATCGTATTTATACATATATTCTTCTTACATATTCAAGGTAGCACTAATCCTTTAGGGTATGATACACCTTTAAAAATACCATTCTATCCAAATCTATTAACTTTAGATGTTAAAGGATTTAACTATGTATTAGTATTAT | 450 | North_America |
| *Haemoproteus* | SIAMEX01 | AF465562 | Full | 1 | DENPET01 | GCTACTGGAGCTACATTTGTATTTATTCTTACTTACTTACATATTTTAAGAGGATTAAATTATTCATATTCATATTTACCTTTATCATGGATTACTGGATTGGTAATATTTTTAATTTCTATTGTTACTGCTTTTATGGGTTATGTTTTACCTTGGGGTCAAATGAGTTTCTGGGGTGCAACCGTTATTACTAATTTATTATATTTTATACCTGGACTTGTTTCATGGATTTGTGGTGGATATACTATAAGTGATCCAACTCTAAAAAGATTTTTTGTATTACATTTTATATTTCCTTTTATAGCTTTATGCATCGTATTTATACATATATTCTTCTTACATTTACAAGGTAGCTCTAATCCTTTAGGATATGATACAGCTTTAAAAATACCTTTCTATCCAAGTCTATTATGTTTAGATATTAAAGGATTTAATAATGTATTAGTTATATTTTTAGCACAAAGTTTATTTGGTATTTT | 479 | North_America |
| *Plasmodium* | SIAMEX02 | EU254538 | Partial | 1 | RBQ16 | GCAACTGGTGCTTCATTTGTATTTATTTTAACTTATTTACATATTTTAAGAGGACTAAATTATTCATATTCATATTTACCTTTATCATGGATATCTGGATTAATAATATTTTTAATATCTATAGTAACAGCTTTTATGGGTTATGTATTACCTTGGGGTCAAATGAGTTTCTGGGGTGCTACTGTAATTACTAATTTATTATATTTTATACCTGGACTTGTTTCATGGATATGTGGTGGATATCTTGTAAGCGACCCAACTTTAAAAAGATTCTTTGTATTACATTTTACATTTCCATTTATAGCTTTATGTATTGTATTTATACATATATTCTTTTTACATTTACAAGGTAGCACAAATC | 361 | North_America |
| *Haemoproteus* | SITTAKRU1 | JN863584 | Full | 1 | MYIFLA01 | AAGAGGACTAAACTATTCATACTCTTACTTACCTTTATCATGGATAACTGGATTAATAATATTCTTAATTTCTATTGTTACTGCTTTTATGGGTTATGTATTACCTTGGGGTCAAATGAGTTTCTGGGGTGCAACCGTTATTACTAATTTACTATATTTTATACCTGGACTTGTTTCATGGATTTGTGGAGGATATACTATTAGTGATCCAACTTTAAAAAGATTTTTTGTATTACATTTTATATTCCCTTTTATAGCCCTATGTATTGTATTTATACATATATTTTTCTTACACTTACAAGGTAGCTCTAATCCTTTAGGATATGATACAGCTTTAAAAATACCTTTCTATCCAAGTCTATTATGTCTAGATATTAAAGGATTTAATAATGTATTAGTCCTATTTCTAGCACAAAGTTTATTTGGAATACT | 432 | North_Africa_._Middle_East |
| *Plasmodium* | SPDEM01 |  | Full | 1 | SPDEM02 | TATTTATTCTTACTTATTTACATATTTTAAGAGGATTAAACTATTCTTATTCATATCTACCTTTATCATGGATATCAGGACTAATTATATTCCTAATATCAATAGTAACTGCTTTTATGGGATATGTATTACCTTGGGGTCAAATGAGTTTTTGGGGTGCAACTGTTATTACTAACTTATTATATTTTATACCTGGTCTCGTTTCATGGATTTGTGGTGGATATCTTGTAAGCGACCCAACATTAAAAAGATTCTTTGTATTACATTTTATATTTCCATTTATAGCCCTATGTATTGTATTTATACATATATTCTTTTTACATTTACAAGGTAGCACAAATCCTTTAGGGTATGATACAGCTTTAAAAATACCCTTCTATCCAAATCTATTAAGTCTTGATATTAAAGGATTTAATAATATATTAGTTTTATTTTTAGCACAAAGTTTATTTGGAATATT | 460 | - |
| *Plasmodium* | SPDEM02 | KY653786 | Full | 1 | SPDEM01 | GCAACAGGTGCATCATTCGTATTTATTCTTACTTATTTACATATTTTAAGAGGATTAAACTATTCTTATTCATATCTACCTTTATCATGGATATCAGGACTAATTATATTCCTAATATCAATAGTAACTGCTTTTATGGGATATGTATTACCTTGGGGTCAAATGAGTTTTTGGGGTGCAACTGTTATTACTAACTTATTATATTTTATACCTGGTCTCGTTTCATGGATTTGTGGTGGATATCTTGTAAGCGACCCAACATTAAAAAGATTCTTTGTATTACATTTTATATTTCCATTTATAGCCCTATGTATTGTATTTATACATATATTCTTTTTACATTTACAAGGTAGCACAAATCCTTTAGGGTATGATACAGCTTTAAAAATACCCTTCTATCCAAATCTATTAAGTCTTGATATTAAAGGATTTAATAATATATTAGTTTTATTTTTAGCACAAAGTTTATTTGGAATATT | 479 | South_Sahara |
| *Plasmodium* | SPMEN01 | GQ395640 | Partial | 1 | SPMEN03 | GCAACAGGAGCTTCATTTGTATTTATTTTAACTTATCTACATATTTTAAGAGGATTAAACTATTCATACTCATATTTACCTTTATCATGGATATCAGGATTAATAATATTCTTAATATCAATAGTTACAGCTTTTATGGGTTATGTATTACCTTGGGGTCAAATGAGTTTCTGGGGTGCAACTGTTATAACTAATTTATTATATTTTATTCCTGGACTTGTCTCATGGATTTGTGGTGGATATCTTGTAAGTGACCCAACTTTAAAAAGATTTTTTGTATTACATTTTACATTTCCATTTATAGCTTTATGTATTGTATTTATACATATATTCTTTTTACATTTACA | 347 | South_America |
| *Haemoproteus* | STRZON01 | DQ241552 | Partial | 1 | PACPOL01 | TTGTGGTGGATATATTATTAGTGATCCAACTTTAAAAAGATTTTTTGTATTACATTTTATATTTCCATTTATAGCTTTATGTATTGTATTTATACATATATTCTTTTTACATTTACAAGGTAGCTCTAATCCTTTAGGATATGATACTGCTTTAAAAATACCTTTCTATCCAAGTCTATTATGTCTAGATATTAAAGGATTTAATAATGTATTAGTCTTATTTCTAGCACAAAGTTTATTTGGTATTTT | 249 | South_America |
| *Haemoproteus* | STSEN1 | DQ451425 | Partial | 1 | SPISEN01 | GCTACTGGTGCTACATTTGTTTTTATATTAACATATTTACATATCTTAAGAGGATTAAATTATTCATATTCATACTTACCTTTATCATGGATAACTGGACTATTAATCTTCTTAATTTCTATTGTTACTGCTTTTATGGGTTATGTATTACCTTGGGGTCAAATGAGTTTCTGGGGTGCAACCGTTATTACTAACTTATTATATTTCATACCTGGACTTGTTTCATGGATTTGTGGTGGATATAATATTAGTGATCCTACTTTAAAAAGATTTTTTGTATTACATTTTATATTCCCATTTATAGCTTTATGTATTGTATTTATACATATATTCTTCTTACACTTACAAGGTAGCACTAATC | 361 | North_Africa_._Middle_East |
| *Leucocytozoon* | SYAT42 |  | Full | 1 | SYCON05 | AAGAGGATTAAATTATTCTTTCTCTTACTTACCTTTATCATGGTATAGTGGTTTAATTATATTCTTAATCTTTATTGTAACTGCTTTTATGGGTTACGTCTTACCATGGGGACAAATGAGTTTCTGGGGAGCAACTGTAATTACTAATTTATTATATTTTATTCCTGGATTAATTAATTGGGTCTGTGGTGGATTTATTATTAATGACCCAACATTAAAAAGATTCTTTGTATTACATTTTATATTCCCATTTGTAGCATTAGCTATTGTATTTATTCATATATTCTTTTTACATATTCATGGTAGCACTAATCCTTTAGGGTATGATACACCTTTAAAAATACCATTCTATCCAAATCTATTAACCTTAGATATTAAAGGATTTAACTATGTATTAGTTATATTTTTATTTCAAAGTTTATTTGGAATTGC | 432 | Europe |
| *Leucocytozoon* | SYBOR25 | Not Sub | Full | 1 | SYCON06 | CATCTTTTGTATTTATATTAACATATCTACATATATTAAGAGGATTAAACTATTCTTTCTCTTACTTACCTTTATCATGGTATAGTGGTTTAATTATATTCTTAATCTTTATTGTAACTGCTTTTATGGGTTACGTCTTACCATGGGGACAAATGAGTTTCTGGGGAGCAACTGTAATTACTAATTTATTATATTTTATTCCTGGATTAATTAATTGGGTCTGTGGTGGATTTATTATTAATGACCCAACATTAAAAAGATTCTTTGTATTACACTTTATATTCCCATTCGTAGCATTAGCTATTGTATTTATTCATATATTCTTTTTACATATTCATGGTAGCACTAATCCTTTAGGGTATGATACACCTTTAAAAATACCATTCTATCCAAATCTATTAACCTTAGATATTAAAGGATTTAACTATGTATTAGTTATATTTTTATTTCAAAGTTTATTTGGAATTGC | 469 | Europe |
| *Haemoproteus* | TABI02 | AF465563 | Full | 1 | TABI05 | GCTACTGGAGCTACATTTGTATTTATTCTTACTTACTTACATATTTTAAGAGGATTAAATTATTCATATTCATATTTACCTTTATCATGGATTACTGGATTGGTAATATTTTTAATTTCTATTGTTACTGCTTTTATGGGTTATGTTTTACCTTGGGGTCAAATGAGTTTCTGGGGTGCAACCGTTATTACTAATTTATTATATTTTATACCTGGACTTGTTTCATGGATTTGTGGTGGATATACTATAAGTGATCCAACTCTAAAAAGATTTTTTGTATTACATTTTATATTTCCTTTTATAGCTTTATGCATCGTATTTATACATATATTCTTCTTACATTTACAAGGTAGCTCTAATCCTTTAGGATATGATACAGCTTTAAAAATACCTTTCTATCCAAGTCTATTATGTTTAGATATTAAAGGATTTAATAATGTATTAGTTATATTTTTAGCACAAAGTTTATTTGGTATTCT | 479 | North_America, South_America |
| *Haemoproteus* | THAFAN01 | KJ661308 | Full | 1 | EUPXAN02 | CATTTGTATTTATTCTAACTTACTTACATATCTTAAGAGGATTAAATTATTCATATTCATATTTACCTATATCATGGATAACAGGATTGGTAATATTCTTAATTTCTATTGTTACTGCTTTTATGGGTTATGTATTACCATGGGGTCAAATGAGTTTCTGGGGTGCAACCGTTATTACTAATTTATTGTACTTTATACCTGGTCTTGTATCATGGATTTGTGGTGGATATACTATAAGCGATCCAACTTTAAAAAGATTTTTTGTACTACATTTTATATTTCCTTTTATAGCCTTATGTATTGTATTTATACATATATTCTTCTTACATTTACAAGGTAGCTCTAATCCTTTAGGATATGATACAGCTTTAAAAATACCTTTCTATCCAAGTCTATTATGTCTAGATATTAAAGGATTTAATAATGTATTAGTTATATTTTTAGCACAAAGTTTAT | 456 | South_America |
| *Leucocytozoon* | TROAED08 | KF767427 | Full | 1 | TURSER01 | CAACAGGTGCATCTTTTGTATTTATATTAACATATTTACATATCTTAAGAGGATTAAATTATTCTTTCTCTTATTTACCTTTATCATGGTATAGTGGTTTAATTATATTTTTAATCTTTATTGTAACTGCTTTTATGGGTTACGTCTTACCATGGGGACAAATGAGTTTCTGGGGAGCTACTGTAATTACTAATTTATTATACTTTATTCCTGGACTAATCAATTGGGTATGTGGTGGATTTATTATTAATGACCCAACATTAAAAAGATTCTTTGTATTACACTTCATATTCCCATTTGTTGCATTAGCTATTGTATTTATTCATATATTCTTCCTACATATTCATGGTAGCACAAATCCTTTAGGGTATGATACACCTTTAAAAATACCATTCTATCCAAATCTATTAACTTTAGATATTAAAGGATTTAACTATGTATTAGTTAT | 448 | South_America |
| *Haemoproteus* | TROAED17 | KF767416 | Full | 1 | ZOCAP19 | CTACTGGAGCCACGTTTGTATTCATTCTAACTTACTTACATATCTTAAGAGGATTAAATTATTCATATTCATATTTACCTTTATCATGGATAACAGGATTGGTAATATTCTTAATTTCTATTGTTACTGCTTTTATGGGTTATGTATTACCATGGGGTCAAATGAGTTTCTGGGGTGCAACCGTTATTACTAATTTATTATACTTTATACCTGGTCTTGTATCATGGATTTGTGGTGGTTATACTATAAGCGATCCAACTTTAAAAAGATTTTTTGTATTACATTTTATATTTCCTTTTGTAGCCTTATGTATTGTATTTATACATATATTCTTTTTACATTTACAAGGTAGCTCTAATCCTTTAGGATATGATACAGCTTTAAAAATACCTTTCTATCCAAGTCTATTATGTCTAGATATTAAAGGTTTTAATAATGTATTAGTTATATTTTTA | 455 | South_America |
| *Leucocytozoon* | TRPIP3 |  | Partial | 1 | ANTTRI02 | AATTATTCTTTTACATACTTACCTTTATCATGGATAAGTGGTTTAGTAATATTCTTAATATTTATTGTAACTGCTTTTATGGGTTATGTTTTACCATGGGGTCAAATGAGTTTTTGGGGAGCTACTGTTATTACTAATTTATTATATTTTATTCCTGGATTAATTAATTGGGTTTGTGGTGGATTTATTATTAATGACCCAACTCTAAAAAGATTCTTCGTATTACATTTTATATTCCCATTTGTAGCACTAGCTATTGTATTTATACATATATTCTTCTTACATATTCAAGGTAGCACTAATCCTTTAGGGTATGATACACCTTTAAAAATACCATTCTATCCAAATCTATTAACTTTAGATATTAAAGGATTTAACTATGTATTAGTATTATTCCTATTTCAAAGTTTATTTGGAATTGC | 422 | - |
| *Plasmodium* | TUBOU01 | KJ145052 | Partial | 1 | TURMER06 | TATTTACATATTTTAAGAGGATTAAATTATTCATATTCATACTTACCTTTATCATGGATATCAGGATTAATAATATTTCTAATATCAATAGTAACAGCTTTTATGGGATATGTATTACCTTGGGGTCAAATGAGTTTTTGGGGTGCAACTGTTATAACTAATTTATTATATTTTATACCTGGTCTTGTTTCATGGATCTGTGGTGGATATCTTGTAAGTGACCCAACATTAAAAAGATTCTTTGTTTTACATTTTATATTTCCATTTATAGCTTTATGTATTGTATTTATACATATATTCTTTTTACATTTACAAGGTAGCACAAATCCTTTAGGGTATGATACAGCTTTAAAAATACCCTTCTATCCAAATCTATTAAGTCTTGATATTAAAGGATTTAATAATATCTTAGTCTTATTTTTAGCACAAAGCTTATTTGGAATATT | 446 | Asia |
| *Leucocytozoon* | TUFAL04 | JX984671 | Partial | 1 | TURFAL05 | GAGCTACAGTAATTACTAATCTATTATATTTTATTCCTGGACTAATTAATTGGGTTTGTGGAGGATTTATTATTAATGACCCAACTCTAAAAAGATTCTTTGTATTACATTTTATATTCCCATTTGTAGCATTAGCAATCGTATTTATACATATATTCTTCTTACATATTCAAGGTAGCACTAATCCTTTAGGGTATGATACACCTTTAAAAATACCATTCTATCCAAATCTATTAACTTTAGATGTTAAAGGATTTAATTATGTAATAGTATTATTCTTATTCCAAAGTTTATTTGGAAT | 301 | South_America |
| *Plasmodium* | TURAF01 | EU810698 | Partial | 1 | MILANS06 | GGTGCTACTGTAATAACCAATTTACTTTATTTTATTCCTGGACTTGTCTCATGGATTTGTGGTGGATATCTTGTAAGTGACCCAACCTTAAAAAGATTCTTTGTATTACATTTTACATTTCCTTTTATAGCTTTATGTATTGTATTTATACATATCTTTTTCTTACATTTACAAGGTAGCACTAATCCTTTAGGGTATGATACAGCTTTAAAAATACCCTTCTATCCAAATCTTTTAAGTCTTGATATTAAAGGATTTAATAATGTATTAGTATTATTCTTAGCTCAAAGTTTATTTGGAATATT | 305 | South_Sahara |
| *Plasmodium* | TURALB06 | MT724409 | Full | 1 | TURAMA06 | TACTGGTGCTACATTTGTATTTATTTTAACTTATTTACATATATTAAGAGGATTAAATTATTCATATTCATATTTACCTTTATCATGGATATCTGGATTAATAATATTCTTAATTTCTATAGTTACTGCTTTTATGGGTTATGTATTACCTTGGGGTCAAATGAGTTTCTGGGGTGCAACCGTTATTACTAATTTATTATATTTTATACCTGGACTTGTTTCATGGATTTGTGGTGGATATATTATTAGTGATCCTACTTTAAAAAGATTCTTTGTATTACATTTTATATTCCCATTTATAGCTTTATGTATTGTATTTATACATATATTCTTTTTACACTTACAAGGTAGCTCTAATCCTTTAGGATATGATACTGCTTTAAAAATACCTTTCTATCCAAGTCTATTATGTCTAGATATTAAAGGATTTAATAATGTATTAGTCTTACTTCTAGCACAAAGTTTATTTGGAATATT | 477 | South_America |
| *Haemoproteus* | TURAMA06 | MT724525 | Full | 1 | TURALB06 | TACTGGTGCTACATTTGTATTTATTTTAACTTATTTACATATATTAAGAGGATTAAATTATTCATATTCATATTTACCTTTATCATGGATATCTGGATTAATAATATTCTTAATTTCTATAGTTACTGCTTTTATGGGTTATGTATTACCTTGGGGTCAAATGAGTTTCTGGGGTGCAACCGTTATTACTAATTTATTATATTTTATACCTGGACTTGTTTCATGGATTTGTGGTGGATATATTATTAGTGATCCTACTTTAAAAAGATTCTTTGTATTACATTTTATATTCCCATTTATAGCTTTATGTATTGTATTTATACATATATTCTTTTTACACTTACAAGGTAGCTCTAATCCTTTAGGATATGATACTGCTTTAAAAATACCTTTCTATCCAAGTCTATTATGTCTAGATATTAAAGGATTTAATAATGTATTAGTCTTACTTCTAGCACAAAGTTTATTTGGAATATT | 477 | South_America |
| *Haemoproteus* | TURASS05 |  | Full | 1 | JUNPHA04 | GCTACCGGTGCTACATTTGTTTTTATTCTAACTTACTTACATATTTTAAGAGGACTAAACTATTCATATTCTTATTTACCTTTATCATGGATTACTGGATTAATAATATTCTTAATTTCAATTGTTACCGCTTTTATGGGTTATGTATTACCTTGGGGTCAAATGAGTTTCTGGGGTGCAACCGTTATTACTAATTTATTATATTTTATACCTGGACTTGTTTCATGGATTTGTGGAGGATATACTATTAGTGATCCAACTTTAAAAAGATTCTTTGTATTACATTTTATATTCCCTTTTATAGCTTTATGTATTGTATTTATACATATATTCTTCTTACACTTACAAGGTAGCTCTAATCCTTTAGGATATGATACAGCTTTAAAAATACCTTTCTATCCAAGTCTATTATGTCTAGATATCAAAGGATTTAATAATGTATTAGTCCTATTTCTAGCACAAAGTTTATTTGGAATTCT | 479 | Central_America |
| *Leucocytozoon* | TURMIG11 |  | Full | 1 | COLPAS08 | CAACAGGTGCATCTTTTGTATTTATATTAACATATTTACACATATTAAGAGGATTAAATTATTCTTTCTCTTACTTACCTTTATCATGGATAAGTGGTTTAATTATATTTTTAATATTTATTGTAACTGCTTTTATGGGTTATGTCTTGCCATGGGGTCAAATGAGTTTCTGGGGAGCTACTGTAATTACTAATCTATTATATTTTATCCCTGGACTAATTAATTGGGTTTGTGGAGGATTTATTATTAATGACCCAACTCTAAAAAGATTCTTTGTATTACATTTTATATTCCCATTTATAGCATTAGCAATCGTATTTATACATATATTCTTCTTACATATTCAAGGTAGCACTAATCCTTTAGGGTATGATACACCTTTAAAAATACCATTCTATCCAAATCTATTAACTTTAGATGTTAAAGGATTTAATTATGTAATAGTATTATTCTTATTTCAAAGTTTATTTGGAATTG | 477 | - |
| *Plasmodium* | TURUF01 | DQ241524 | Partial | 1 | TURUF03 | TTGTGGTGGATATCTTGTTAGTGATCCAACATTAAAAAGATTTTTTGTTTTACATTTTATATTTCCATTTATAGCTTTATGTATTGTATTTATACATATATTCTTTTTACATTTACAAGGTAGCACAAATCCTTTAGGGTATGATACAGCTTTAAAAATACCCTTCTATCCAAATCTATTAAGTCTTGATATTAAAGGATTTAATAATATTCTAGTTTTATTTTTAGCACAAAGTTTATTTGGAATATT | 249 | South_America |
| *Leucocytozoon* | TUSW01 | JQ314220 | Full | 1 | ANSFAB01 | TTAACTTACTTACATATCTTAAGAGGATTAAATTATTCTTTCTGTTACTTACCTTTATCATGGAGTAGTGGTTTAATTATATTTTTAATATTTATTGTTACTGCTTTCATGGGATATGTTTTACCATGGGGTCAAATGAGTTTCTGGGGAGCAACAGTAATTACTAATTTATTATATTTTATTCCTGGATTAATTAATTGGGTTTGTGGTGGATTTATAATTAATGATCCAACATTAAAAAGATTTTTCGTATTACATTTTATATTCCCATTCGTAGCTTTAGCTATTGTATTTATACATATATTCTTCTTACATATACAAGGTAGCACTAATCCTCTAGGGTATGATACACCTTTAAAAATACCATTCTATCCAAGTCTATTAACTTTAGACATTAAAGGATTTAATTATGTATTTGTATTA | 423 | North_America |
| *Leucocytozoon* | TUSW02 | JQ314221 | Full | 1 | CLAHYE01 | TTAACCTACTTACATATCTTAAGAGGATTAAATTATTCTTTCTGTTACTTACCTTTATCATGGAGTAGTGGTTTAATTATATTTTTAATATTTATTGTTACTGCTTTCATGGGATATGTTTTACCATGGGGTCAAATGAGTTTCTGGGGAGCAACAGTAATTACTAATTTATTATATTTTATTCCTGGATTAATAAATTGGGTTTGTGGTGGATTTATAATTAATGATCCAACATTAAAAAGATTTTTCGTATTACATTTTATATTCCCATTCGTAGCTTTAGCTATTGTATTTATACATATATTCTTCTTACATATACAAGGTAGCACTAATCCTCTAGGGTATGATACACCTTTAAAAATACCATTCTATCCAAGTCTATTAACTTTAGACATTAAAGGATTTAATTATGTATTTGTATTA | 423 | North_America |
| *Haemoproteus* | TUSW06 | JQ314225 | Full | 1 | ANACRE01 | TTAACATATTTACATATCTTAAGAGGATTAAATTATTCATATTCATATTTACCTTTATCATGGATAACAGGACTAATGATTTTCTTAATTTCTATTGTTACTGCTTTTATGGGTTATGTATTACCTTGGGGTCAAATGAGTTTCTGGGGTGCAACCGTTATTACTAACTTATTATATTTTATACCTGGACTTGTTTCATGGATTTGTGGTGGATATAATATTAGTGATCCTACTTTAAAAAGATTCTTTGTATTACATTTTATATTCCCATTTATAGCTTTATGTATTGTATTTATACATATATTCTTTTTACATTTACAAGGTAGCTCTAATCCTTTAGGATATGATACAGCTTTAAAAATACCTTTCTATCCAAGTCTATTATGTTTAGATATTAAAGGATTTAGTAATGTATTTGTATTA | 423 | North_America |
| *Haemoproteus* | ZEGAL06 | GU296218 | Partial | 1 | ZEGAL07 | GCAACAGGTGCATCATTTGTATTTATTTTAACATACCTACACATTTTAAGAGGATTAAATTACTCATATTCATATTTACCATTATCATGGATTACCGGATTAATCATATTTATAATCTCTATTATGACTGCTTTCTTAGGTTATGTTCTACCTTGGGGTCAAATGAGTTTCTGGGGTGCAACTGTTATTACTAATTTATTATATTTTATTCCAGGATTAGTCTCATGGATTTGTGGTGGATATATTGTTAGTGATCCTACACTAAAAAGATTCTTTGTATTACATTTTATATTTCCATTTATAGCTATATGTATTGTATTTATTCATATATTCTTTTTACATCTACAAGGTAGCTCTAATCCTTTAGGATATGATACAGCATTAAAAA | 388 | South_America |
| *Plasmodium* | ZOCAP03 | EF153643 | Full | 1 | APSPI14 | ACTTATTTACATATTTTAAGAGGATTAAATTATTCATATTCATATTTACCTTTATCATGGATATCTGGACTAATTATCTTTTTAATATCTATTGTAACAGCTTTTATGGGTTATGTATTACCTTGGGGTCAAATGAGTTTCTGGGGTGCTACAGTTATAACTAATTTATTATATTTTATACCTGGACTTGTTTCATGGATATGTGGTGGATATCTTGTAAGTGACCCAACCTTAAAAAGATTCTTTGTATTACATTTTACATTTCCATTTATAGCCTTATGTATTGTATTTATACATATATTCTTTTTACATTTACAAGGTAGCACAAATCCTTTAGGGTATGATACAGCTTTAAAAATACCCTTCTATCCAAATCTTTTAAGTCTTGATATTAAAGGATTTAATAATGTATTAGTATTATTTTTAGCACAAAGTTTATTTGGAATACT | 449 | South_America, Asia |
| *Leucocytozoon* | ZOCAP06 | EF153665 | Partial | 1 | TROAED02 | AACTATTCATTCTCTTACTTACCTTTATCATGGTATAGTGGTTTAGTTATATTCTTAATCTTTATTGTAACTGCTTTTATGGGTTACGTTTTACCATGGGGACAAATGAGTTTCTGGGGAGCAACTGTAATTACTAATTTATTATATTTTATTCCTGGATTAATTAATTGGGTCTGTGGTGGATTCATAATTAATGACCCAACATTAAAAAGATTCTTCGTATTACACTTTATATTCCCATTTGTAGCCTTAGCTATTGTATTTATTCATATATTCTTCTTACATATTCATGGTAGCACTAATCCTTTAGGGTATGATACACCTTTAAAAATACCATTCTATCCAAATCTATTAACTTTAGATATTAAAGGATTTAACTATGTATTAGTTATATTTTTATTTCAAAGTTTATTTGGAATTGC | 422 | South_America, Asia |
| *Haemoproteus* | ZOCAP19 | MH444684 | Full | 1 | TROAED17 | GCTACTGGAGCCACGTTTGTATTCATTCTAACTTACTTACATATCTTAAGAGGATTAAATTATTCATATTCATATTTACCTTTATCATGGATAACAGGATTGGTAATATTCTTAATTTCTATTGTTACTGCTTTTATGGGTTATGTATTACCATGGGGTCAAATGAGTTTCTGGGGTGCAACCGTTATTACTAATTTATTATACTTTATACCTGGTCTTGTATCATGGATTTGTGGTGGTTATACTATAAGCGATCCAACTTTAAAAAGATTTTTTGTATTACATTTTATATTTCCTTTTGTAGCCTTATGTATTGTATTTATACATATATTCTTTTTACATTTACAAGGTAGCTCTAATCCTTTAGGATATGATACAGCTTTAAAAATACCTTTCTATCCAAGTCTATTATGTCTAGATATTAAAGGTTTTAATAATGTATTAGTTATATTTTTAGCACAAAGTTTGTTTGGAATATT | 479 | South_America |
| *Haemoproteus* | ZOSFLA01 | FJ664159 | Partial | 1 | ZOSLAT04 | GGTGCAACCGTTATTACTAATTTATTATATTTTATACCTGGATTAGTTTCATGGATTTGTGGTGGATATATTATTAGTGATCCAACTTTAAAAAGATTTTTTGTATTACATTTTATATTTCCATTTATAGCTTTATGTATTGTATTTATACATATATTCTTTTTACACTTACAAGGTAGCTCTAATCCTTTAGGATATGATACTGCTTTAAAAATACCTTTCTATCCAAGTCTATTATGTCTAGATATTAAAGGATTTAATAATGTATTAGTCTTATTTCTAGCACAAAGTTTATTCGGAATTTT | 305 | Oceania |
| *Haemoproteus* | ZOSLAT11 | KX604235 | Full | 1 | ZOSXAN02 | GCTACTGGTGCTACATTTGTCTTTATTTTAACTTATTTACATATATTAAGAGGATTAAATTATTCATATTCATATTTACCATTATCATGGATAACAGGATTAATAATATTTTTAATTTCTATTGTTACTGCTTTTATGGGTTATGTACTACCTTGGGGTCAAATGAGTTTCTGGGGTGCAACCGTTATCACTAATTTATTATATTTTATACCTGGATTAGTTTCATGGATTTGTGGTGGATATATTATTAGTGATCCAACTTTAAAAAGATTTTTTGTATTACATTTTATATTTCCATTTATAGCTTTATGTATTGTATTTATACATATATTCTTTTTACACTTACAAGGTAGCTCTAATCCTTTAGGATATGATACTGCTTTAAAAATACCTTTCTATCCAAGTCTATTATGTCTAGATATTAAAGGATTTAATAATGTATTAGTC | 447 | Oceania |
| *Haemoproteus* | ZOSLUG02 | KT595668 | Full | 1 | ZOSLUG01 | TACATATTTTAAGAGGATTAAATTATTCATATTCATATTTACCTGCATCATGGATAACTGGATTAATTATATTCTTAATTTCTATTGTTACTGCTTTTATGGGTTATGTTTTACCTTGGGGTCAAATGAGTTTCTGGGGTGCAACCGTTATTACTAATTTATTATACTTTATACCTGGACTTGTTTCATGGATTTGTGGTGGATATACTATAAGTGATCCAACCTTAAAAAGATTTTTTGTATTACATTTCATATTTCCATTTATAGCCTTATGTATTGTCTTTATTCATATATTTTTCCTACACTTACAAGGTAGCTCTAATCCTTTAGGATATGATACAGCTTTAAAAATACCTTTCTATCCAAGTCTATTATGTCTAGATATTAAAGGATTTAATAATGTATTAGTTATATTTTTAGCACAAAGTTTATTTGGAATTCT | 442 | South_Sahara |
| *Haemoproteus* | ZOSPAL01 | EF380172 | Partial | 1 | ZOSPAL06 | GGTGCAACCGTTATTACTAATTTATTATATTTTATTCCTGGATTAGTTTCATGGATTTGTGGTGGATATATTATTAGTGATCCAACTTTAAAAAGATTTTTTGTATTACATTTTATATTCCCATTTATAGCTTTATGTATTGTATTTATACATATATTCTTTTTACACTTACAAGGTAGCTCTAATCCTTTAGGATATGATACTGCTTTAAAAATACCTTTCTATCCAAGTCTATTATGTCTAGATATTAAAGGATTTAATAATGTATTAGTCTTATTTCTAGCACAAAGTTTATTCGGAATTTT | 305 | Asia |
| *Leucocytozoon* | ACCBRE02 | DQ177235 | Full | 1 | ACCBRE01 | TCTACAGGTGCATCATTTGTATTTATTCTAACATACTTACATATATTAAAAGGATTGAATTATTCATATTCATATTTACCATTATCATGGATTACTGGAATTGTAATATTTCTAATATCTATAGTAACCGCTTTCTTAGGTTATGTTTTACCATGGGGTCAAATGAGTTTCTGGGGTGCTACCGTAATTACTAATTTATTATATTTTATTCCTGGACTAATTTCATGGGTCTGTGGTGGATATGCTGTAGGCGATCTTACTCTAAAAAGATTCTTTGTATTACACTTTATTTTTCCATTTGTAGCTTTAGCTATTGTATTTATTCATATATTCTTTCTACATTTACAAGGTAGCAGTAATCCTTTAGGATATGATACACCATTAAAAATACCCTTCTATCCAAATCTATTATGCTTAGATATAAAAGGATTGATGAGTGTATTAATATTATTTATAGCTCAAAGCTTCTTTGGTATTCT | 479 | Asia |
| *Leucocytozoon* | ACCBRE03 | DQ177236 | Full | 1 | ACCBRE01 | TCTACAGGTGCATCATTTGTATTTATTCTAACATACTTACATATATTAAAAGGATTGAATTATTCATATTCATATTTACCATTATCATGGATTACTGGAATTGTAATATTTCTAATATCTATAGTAACCGCTTTCTTAGGTTATGTTTTACCATGGGGTCAAATGAGTTTCTGGGGTGCTACCGTAATTACTAATTTATTATATTTTATTCCTGGACTAATTTCATGGGTCTGTGGTGGATATGCTGTAGGCGATCTTACTCTAAAAAGATTCTTTGTATTACACTTTATTTTTCCATTTGTAGCTTTAGCTATTGTATTTATTCATATATTCTTTCTACATTTACAAGGTAGCAGTAATCCTTTAGGATATGATACACCATTAAAAATACCCTTCTATCCAAATCTATTATGCTTAGATATAAAAGGATTGATGAGTGTATTAATCTTATTTATAGCTCAAAGCTTCTTTGGTATTCT | 479 | Asia |
| *Haemoproteus* | ACDUM3 | KY695227 | Full | 1 | ACAED01 | GCTACTGGAGCTACATTTGTTTTTATTTTAACTTACTTACATATTTTAAGAGGATTAAATTACTCATATTCATACTTACCTTTATCATGGATATCTGGATTATTAATATTCTTAATTTCTATTGTTACTGCTTTTATGGGTTATGTTTTACCTTGGGGTCAAATGAGTTTCTGGGGTGCAACCGTTATTACTAATTTATTATATTTTATTCCTGGACTTGTATCATGGATTTGTGGTGGATATACTATTAGTGATCCAACCTTAAAAAGATTCTTCGTATTACATTTTATATTTCCATTTATAGCCTTATGTATTGTTTTTATACATATATTCTTCTTACACTTACAAGGTAGCTCTAATCCTTTAGGATATGATACAGCTTTAAAAATACCTTTCTATCCAAGTCTATTATGTTTAGATGTTAAAGGATTTAATAATGTATTAGTTATATTCTTAGCACAAAGTTTATTTGGAATTCT | 479 | Asia |
| *Haemoproteus* | AECA01 |  | Full | 1 | FIZAN01 | GCTACAGGTGCTACATTTGTATTTATTTTAACTTACCTACATATTTTAAGAGGATTAAATTATTCATACTCTTACTTACCTTTATCATGGATAACTGGATTAATCATATTCTTAATTTCTATTGTTACTGCTTTCATGGGTTATGTATTACCTTGGGGTCAAATGAGTTTCTGGGGTGCAACCGTTATTACTAATTTACTATATTTTATACCTGGACTTGTTTCATGGATTTGTGGAGGATATACTATTAGTGATCCAACTTTAAAAAGATTTTTTGTATTACATTTTATATTTCCATTTATAGCCCTATGTATTGTATTTATACATATATTCTTCTTACACTTACAAGGTAGCTCTAATCCTTTAGGATATGATACAGCTTTAAAAATACCTTTCTATCCAAGTCTATTATGTCTAGATATTAAAGGATTTAATAATGTATTAGTTTTATTTTTAGCACAAAGTTTATTTGGAATACT | 479 | Asia |
| *Plasmodium* | AEDTAE06 | HQ853677 | Full | 1 | NEOLI01 | TCATTTGTATTTATTCTTACTTATTTACATATTTTAAGAGGATTAAACTATTCTTATTCATATTTACCTTTATCATGGATGTCAGGATTAATAATATTTTTAATATCAATAGTAACTGCTTTTATGGGGTATGTATTACCTTGGGGTCAAATGAGTTTCTGGGGTGCAACTGTTATTACCAACTTACTCTACTTTATACCTGGTCTTGTTTCATGGATTTGTGGTGGATATCTTGTAAGTGATCCAACATTAAAAAGATTTTTTGTATTACATTTTATATTTCCATTTATAGCTTTATGTATTGTGTTTATACATATATTCTTTCTACATTTACAAGGTAGCACAAATCCTTTAGGATATGATACAGCTTTAAAAATACCCTTCTATCCAAATCTATTAAGTCTTGATATTAAAGGATTTAATAATATCTTAGTTTTATTTTTAGCACAAAGTTTATTTGGAATATT | 467 | Central_America |
| *Plasmodium* | AEDVEX01 | HQ677623 | Full | 1 | EMHOR1 | GCAACAGGTGCTTCATTTGTATTTATTTTAACTTATCTACATATTTTAAGAGGATTAAATTATTCATATTCATACTTACCTTTATCATGGATATCTGGATTATTAATATTTTTAATATCTATAGTAACAGCTTTTATGGGTTATGTATTACCTTGGGGTCAAATGAGTTTCTGGGGTGCTACAGTAATTACTAATTTATTATATTTTATACCTGGACTTGTTTCATGGATATGTGGTGGATATCTTGTAAGTGACCCAACATTAAAAAGATTTTTTGTATTACACTTTACATTTCCATTTATAGCTTTATGTATTGTATTTATACATATATTCTTTTTACATTTACAAGGTAGCACAAATCCTTTAGGGTATGATACAGCTTTAAAAATACCCTTCTATCCAAATCTTTTAAGTCTTGATATTAAAGGATTCAATAATGTATTAGTATTATTTTTAGCACAAAGTTTATTTGGAATATT | 479 | North_Africa_._Middle_East |
| *Leucocytozoon* | AFR176 | KM056494 | Full | 1 | AFR241 | CAACAGGTGCATCATTCGTATTTATATTAACATACTTACATATATTAAGAGGATTAAATTATTCTTTCACTTACTTACCTTTATCATGGATAAGTGGTTTAATAATATTCTTAATATTTATTGTAACTGCTTTTATGGGTTACGTCTTACCATGGGGTCAAATGAGTTTTTGGGGAGCTACTGTTATTACTAATTTATTATATTTTATTCCTGGACTAATTAATTGGGTTTGTGGTGGATTTATTATTAATGACCCAACTTTAAAAAGATTCTTCGTATTACATTTTATATTCCCATTTGTAGCATTAGCTATTGTATTTATACATATATTCTTCTTACATATTCAAGGTAGCACTAATCCTTTAGGGTATGATACACCTTTAAAAATACCATTCTATCCAAATCTATTAACTTTAGATGTTAAAGGATTTAACTATGTATTAGTATTATTCCTATTTCAAAGTTTATTTGGAATT | 476 | South_Sahara |
| *Plasmodium* | AFTRU07 | EU810631 | Partial | 1 | CXRES01 | GGTGCAACTGTTATTACTAATTTATTATATTTTATACCTGGTCTTGTTTCATGGATTTGTGGTGGATATCTTGTTAGTGATCCAACATTAAAAAGATTTTTTGTTTTACATTTTATATTTCCATTTATAGCTTTATGTATTGTATTCATACATATATTCTTTCTACATCTACAAGGTAGCACAAATCCTTTAGGGTATGATACAGCTTTAAAAATACCCTTCTATCCAAATCTTTTAAGTCTTGATATTAAAGGATTTAATAATATTCTAGTTTTATTTTTAGCACAAAGTTTATTTGGAATATT | 305 | South_Sahara |
| *Haemoproteus* | AKGPH02 | KU257615 | Partial | 1 | STTRA01 | TATTAACATACTTACATATCTTAAGAGGATTAAATTATTCGTATTCATACTTACCTTTATCATGGATAACTGGATTAATGATTTTCTTAATTTCTATTGTTACTGCTTTTATGGGTTATGTATTACCTTGGGGTCAAATGAGTTTCTGGGGTGCAACCGTTATCACTAATTTATTATATTTTATACCTGGACTTGTTTCATGGATTTGTGGTGGATATAATATTAGTGATCCTACTTTAAAAAGATTCTTTGTATTACATTTTATATTTCCATTTGTAGCTTTATGTATTGTATTTATACATATATTCTTTTTACACTTACAAGGTAGCTCTAATCCTTTAGGATATGATACAGCTTTAAAAATACCTTTCTATCCAAGTCTATTATGTTTAGATATTAAAGGATTTAGTAATGTATTAGTATTA | 425 | - |
| *Haemoproteus* | AKGPH05 | KU257618 | Partial | 1 | STTRA01 | TATTAACATACTTACATATCTTAAGAGGATTAAATTATTCATATTCATACTTACCTTTATCATGGATAACTGGATTAATTATTTTCTTAATTTCTATTGTTACTGCTTTTATGGGTTATGTATTACCTTGGGGTCAAATGAGTTTCTGGGGTGCAACCGTTATCACTAACTTATTATATTTTATACCTGGACTTGTTTCATGGATTTGTGGTGGATATAATATTAGTGATCCTACTTTAAAAAGATTCTTTGTATTACATTTTATATTTCCATTTGTAGCTTTATGTATTGTATTTATACATATATTCTTTTTACACTTACAAGGTAGCTCTAATCCTTTAGGATATGATACAGCTTTAAAAATACCTTTCTATCCAAGTCTATTATGTTTAGATATTAAAGGATTTAGTAATGTATTAGTATTA | 425 | - |
| *Leucocytozoon* | AKGPL03 | KU257622 | Partial | 1 | CALMIN01 | TATTAACATATTTACACATATTAAGAGGATTAAATTATTCTTTCTGTTACTTACCTTTATCATGGATAAGTGGATTAGTAATATTTTTAATATTTATTGTAACTGCTTTTATGGGTTATGTATTACCATGGGGTCAAATGAGTTTTTGGGGAGCTACTGTAATTACTAATTTATTATATTTTATTCCTGGACTAATTAATTGGGTTTGTGGTGGATTTATTATTAATGACCCAACACTTAAAAGATTCTTTGTATTACATTTCATATTCCCATTTATAGCACTAGCTATTGTATTTATACATATATTCTTTCTACATATTCAAGGTAGCACTAATCCTTTAGGGTATGATACACCTTTAAAAATACCATTCTATCCAAATCTATTAACTTTAGATGTAAAAGGATTTAATTATGTATTAGTAATA | 425 | - |
| *Leucocytozoon* | AKGPL06 | KU257625 | Partial | 1 | LAMUT01 | TATTAACATATCTACATATATTAAGAGGACTAAACTATTCTTACTCATATTTACCTCTATCATGGATATCAGGTTTAATTATATTCTTTATATCCATAATGACAGCCTTTATGGGTTATGTTTTACCATGGGGACAAATGAGTTATTGGGGAGCAACTGTAATTACCAATTTATTATATTTTATTCCTGGATTAATTTCATGGGTTTGTGGAGGATTCGTTGTTAATGATCCAACTATAAAAAGATTCTTTGTACTTCACTTTATTTTCCCATTTATAGCATTAATTATAGTATTTATTCACATATTTTACTTACATCTACAAGGTAGCACTAATCCTTTAGGATATGATACAGCTCTAAAAATACCCTTCTATCCAAATCTTTTATGCCTAGATATTAAAGGATTTGCAAATGTACTAGTATTA | 425 | - |
| *Haemoproteus* | ALCPOI02 | MK493368 | Full | 1 | ALMOR06 | GCTACTGGTGCTACATTTGTCTTTATTTTAACTTATTTACATATATTAAGAGGATTAAATTATTCATATTCATATTTACCTTTATCATGGATATCTGGATTATTAATATTCTTAATTTCTATTGTTACTGCTTTTATGGGTTATGTATTACCTTGGGGTCAAATGAGTTTCTGGGGTGCAACCGTTATAACTAATTTATTATATTTTATACCTGGACTAGTCTCATGGATTTGTGGTGGATATATTATTAGTGATCCAACTTTAAAAAGATTTTTCGTATTACATTTTATATTCCCTTTTATTGCTTTATGTATTGTATTTATACATATATTCTTTTTACACTTACAAGGTAGCTCTAATCCTTTAGGATATGATACTGCTTTAAAAATACCTTTCTATCCAAGTCTATTATGTCTAGATATTAAAGGATTTAATAATGTATTAGTCTTATTTCTAGCACAAAGTTTATTTGGAATATT | 479 | Asia |
| *Plasmodium* | ALEDIA01 | DQ659552 | Full | 1 | RBQ16 | GCAACTGGTGCTTCATTTGTATTTATTTTAACTTATTTACATATTTTAAGAGGATTAAACTATTCATATTCATATTTACCTTTATCATGGATGTCTGGATTAATTATATTTTTAATATCTATTGTAACAGCTTTTATGGGTTATGTATTACCTTGGGGTCAAATGAGTTTCTGGGGTGCTACAGTTATTACTAATTTATTATATTTTATACCTGGACTTGTTTCATGGATATGTGGTGGATATCTTGTAAGTGACCCAACCTTAAAAAGATTCTTTGTATTACATTTTACATTTCCATTTATAGCTTTATGTATTGTATTTATACATATATTCTTTTTACATTTACAAGGTAGCACAAATCCTTTAGGGTATGATACAGCTTTAAAAATACCCTTCTATCCAAATCTTTTAAGTCTTGATATTAAAGGATTTAATAATGTATTAGTATTATTTTTAGCACAAAGTTTATTTGGAATATT | 479 | South_Sahara, South_America, Central_America |
| *Plasmodium* | ALERUF02 | EU395838 | Full | 1 | PADOM07 | CAACAGGTGCTTCATTTGTATTTATTTTAACTTACTTACATATTTTAAGAGGATTAAATTATTCATATTCATATTTACCTTTATCATGGATATCTGGATTAATAATATTTTTAATATCTATAGTAACAGCTTTTATGGGTTACGTATTACCTTGGGGTCAAATGAGTTTCTGGGGTGCTACCGTAATAACTAATTTATTATATTTTATACCTGGACTAGTTTCATGGATATGTGGTGGATATCTTGTAAGTGACCCAACCTTAAAAAGATTCTTTGTACTACATTTTACATTTCCTTTTATAGCTTTATGTATTGTATTTATACATATATTCTTTCTACATTTACAAGGTAGCACAAATCCTTTAGGGTATGATACAGCTTTAAAAATACCCTTCTATCCAAATCTTTTAAGTCTTGATATTAAAGGATTTAATAATGTATTAGTACTATTTTTAGCACAAAGTTTATTTGGAATAC | 477 | Europe |
| *Plasmodium* | AMMAUR01 | KF482349 | Full | 1 | SEIAUR02 | GCAACAGGTGCATCATTTGTATTTATTCTTACATATTTACATATTTTAAGAGGATTAAATTATTCTTATTCTTATTTACCTTTATCATGGATATCAGGATTAATAATATTTTTAATATCAATAGTTACTGCTTTTATGGGATATGTACTACCTTGGGGTCAAATGAGTTTCTGGGGTGCAACCGTCATTACTAATTTATTATATTTTATACCTGGTCTTGTTTCATGGATCTGTGGTGGATATCTTGTAAGCGACCCAACATTAAAAAGATTTTTTGTATTACATTTTATATTTCCATTTATAGCCTTATGTATTGTATTTATACATATATTCTTTCTACATTTACAAGGTAGCACAAATCCTTTAGGGTATGATACAGCTTTAAAAATACCCTTCTATCCAAATCTATTAAGTCTTGATATTAAAGGATTTAATAATATCTTAGTTTTATTTTTAGCACAAAGTTTATTTGGAATATT | 479 | South_America |
| *Haemoproteus* | ANACRE01 | GQ141557 | Full | 1 | TUSW06 | GCTACTGGTGCTACATTTGTTTTTATATTAACATATTTACATATCTTAAGAGGATTAAATTATTCATATTCATATTTACCTTTATCATGGATAACAGGACTAATGATTTTCTTAATTTCTATTGTTACTGCTTTTATGGGTTATGTATTACCTTGGGGTCAAATGAGTTTCTGGGGTGCAACCGTTATTACTAACTTATTATATTTTATACCTGGACTTGTTTCATGGATTTGTGGTGGATATAATATTAGTGATCCTACTTTAAAAAGATTCTTTGTATTACATTTTATATTCCCATTTATAGCTTTATGTATTGTATTTATACATATATTCTTTTTACATTTACAAGGTAGCTCTAATCCTTTAGGATATGATACAGCTTTAAAAATACCTTTCTATCCAAGTCTATTATGTTTAGATATTAAAGGATTTAGTAATGTATTTGTATTATACTTAGCTCAAAGCTTATTTGGTATATT | 479 | North_America |
| *Leucocytozoon* | ANACRE02 | AB743872 | Full | 1 | ANACU02 | TCAACTGGTGCATCTTTTGTATTTATTTTAACCTACTTACATATCTTAAGAGGATTAAATTATTCTTTCTGTTACTTACCTTTATCATGGAGTAGTGGTTTAATTATATTCTTAATATTTATTGTTACTGCTTTCATGGGATATGTTTTACCATGGGGTCAAATGAGTTTCTGGGGAGCAACTGTAATTACTAATTTATTATATTTTATTCCTGGATTAATTAATTGGGTTTGTGGTGGATTTATAATTAATGATCCAACATTAAAAAGATTCTTTGTATTACATTTTATATTCCCATTCGTAGCTTTAGCTATTGTATTTATACATATATTCTTCTTACATATTCAAGGTAGCACTAATCCTCTAGGGTATGATACACCTTTAAAAATACCATTCTATCCAAGTCTATTAACTTTAGACATTAAAGGATTTAATTATGTATTTGTATTATTCCTATTTCAAAGTTTATTTGGAATTGC | 479 | Asia |
| *Haemoproteus* | ANSOM01 | KM211350 | Full | 1 | ANIFLA01 | CTAACTTACTTACATATTTTAAGAGGGTTAAACTATTCATATTCTTATTTACCTTTATCATGGATAACTGGATTAGTTATATTCTTAATTTCAATTGTTACCGCTTTTATGGGTTATGTATTACCTTGGGGTCAAATGAGTTTCTGGGGTGCAACCGTTATTACTAATTTATTATATTTTATTCCTGGACTTGTTTCATGGATTTGTGGAGGATATACTATTAGTGATCCAACTCTAAAAAGATTTTTTGTATTACATTTTATATTTCCTTTTATAGCTTTATGTATTGTATTTATACATATATTCTTTTTACACTTACAAGGTAGCTCTAATCCTTTAGGATATGATACAGCTTTAAAAATACCTTTCTATCCAAGTCTATTATGTCTAGATATCAAAGGATTTAATAATGTATTAGTCCTATTTCTAGCACAAAGTTTATTTGGAATTTT | 452 | South_America |
| *Leucocytozoon* | ANSFAB01 | AB741512 | Full | 1 | TUSW01 | TCAACTGGTGCATCTTTTGTATTTATTTTAACTTACTTACATATCTTAAGAGGATTAAATTATTCTTTCTGTTACTTACCTTTATCATGGAGTAGTGGTTTAATTATATTTTTAATATTTATTGTTACTGCTTTCATGGGATATGTTTTACCATGGGGTCAAATGAGTTTCTGGGGAGCAACAGTAATTACTAATTTATTATATTTTATTCCTGGATTAATTAATTGGGTTTGTGGTGGATTTATAATTAATGATCCAACATTAAAAAGATTTTTCGTATTACATTTTATATTCCCATTCGTAGCTTTAGCTATTGTATTTATACATATATTCTTCTTACATATACAAGGTAGCACTAATCCTCTAGGGTATGATACACCTTTAAAAATACCATTCTATCCAAGTCTATTAACTTTAGACATTAAAGGATTTAATTATGTATTTGTATTATTCCTATTTCAAAGTTTATTTGGAATTGC | 479 | Asia |
| *Leucocytozoon* | ANTTRI02 |  | Full | 1 | TRPIP3 | TACTAACATACTTACATATATTAAGAGGATTAAATTATTCTTTTACATACTTACCTTTATCATGGATAAGTGGTTTAGTAATATTCTTAATATTTATTGTAACTGCTTTTATGGGTTATGTTTTACCATGGGGTCAAATGAGTTTTTGGGGAGCTACTGTTATTACTAATTTATTATATTTTATTCCTGGATTAATTAATTGGGTTTGTGGTGGATTTATTATTAATGACCCAACTCTAAAAAGATTCTTCGTATTACATTTTATATTCCCATTTGTAGCACTAGCTATTGTATTTATACATATATTCTTCTTACATATTCAAGGTAGCACTAATCCTTTAGGGTATGATACACCTTTAAAAATACCATTCTATCCAAATCTATTAACTTTAGATATTAAAGGATTTAACTATGTATTAGTATTATTCCTATTTCAAAGTTTATTTGGAATTGC | 454 | Europe |
| *Leucocytozoon* | APSPI12 | MN083265 | Full | 1 | SEPSEP01 | CAACAGGTGCATCTTTTGTATTTATATTAACATATCTACATATATTAAGAGGATTAAACTATTCTTACACTTACTTACCATTATCATGGATAAGTGGTTTAGTTATATTTTTAATATTTATTGTAACTGCTTTTATGGGTTATGTCTTACCATGGGGTCAAATGAGTTTTTGGGGAGCTACTGTAATAACTAATTTATTATATTTTATTCCTGGATTAATCAATTGGGTTTGTGGTGGATTTATTATTAATGATCCAACACTAAAAAGATTCTTTATATTACATTTTATATTCCCATTCGTAGCACTTGCAATTGTATTTATTCATATATTCTTCTTACATATTCAAGGTAGCACTAATCCTTTAGGGTATGATACACCTTTAAAAATACCATTCTATCCAAATCTATTAACTTTAGATGTTAAAGGATTAAATTATGTATTAGTATTATTCTTATTTCAAAGTTTATTTGGAATTGC | 478 | South_America |
| *Plasmodium* | APSPI14 | MN083267 | Full | 1 | ZOCAP03 | CAACAGGTGCTTCATTTGTATTTATTTTAACTTATTTACATATTTTAAGAGGATTAAATTATTCATATTCATATTTACCTTTATCATGGATATCTGGACTAATTATCTTTTTAATATCTATTGTAACAGCTTTTATGGGTTATGTATTACCTTGGGGTCAAATGAGTTTCTGGGGTGCTACAGTTATAACTAATTTATTATATTTTATACCTGGACTTGTTTCATGGATATGTGGTGGATATCTTGTAAGTGACCCAACCTTAAAAAGATTCTTTGTATTACATTTTACATTTCCATTTATAGCCTTATGTATTGTATTTATACATATATTCTTTTTACATTTACAAGGTAGCACAAATCCTTTAGGGTATGATACAGCTTTAAAAATACCCTTCTATCCAAATCTTTTAAGTCTTGATATTAAAGGATTTAATAATGTATTAGTATTATTTTTAGCACAAAGTTTATTTGGAATACT | 478 | South_America |
| *Haemoproteus* | ARGCAU1 |  | Full | 1 | TURGUL01 | GCTACCGGTGCTACATTTGTATTTATATTAACCTACTTACATATTTTAAGAGGATTAAATTATTCATATTCATATTTACCTTTATCATGGATAACCGGATTAATCATATTCTTAATTTCCATTGTTACCGCTTTTATGGGTTATGTATTACCTTGGGGTCAAATGAGTTTCTGGGGTGCAACCGTTATTACTAATTTATTATATTTTATACCTGGACTTGTTTCATGGATTTGTGGAGGATATACTATTAGTGATCCAACTTTAAAAAGATTTTTTGTATTACATTTTATATTTCCTTTTATAGCTTTATGTATTGTATTTATTCATATATTCTTTTTACACTTACAAGGTAGCTCTAATCCTTTAGGATATGATACAGCTTTAAAAATACCTTTCTATCCAAGTCTATTATGTCTAGATATCAAAGGATTTAATAATGTATTAGTTTTATTCCTAGCACAAAGTCTATTTGGAATTCT | 479 | - |
| *Haemoproteus* | ASCLA01 | MT919270 | Partial | 1 | THAMEL01 | TATTCATACTTACCTTTATCATGGATTACTGGATTAATGATTTTCTTAATTTCTATTGTCACTGCTTTTATGGGTTATGTATTACCTTGGGGTCAAATGAGTTTCTGGGGTGCAACCGTTATTACTAACTTATTATATTTCATACCTGGACTTGTTTCATGGATTTGTGGTGGATATAATATTAGTGATCCTACTTTAAAAAGATTCTTTGTATTACATTTTATTTTTCCATTTATAGCTTTATGTATTGTATTTATACATATATTCTTTTTACACTTACAAGGTAGCTCTAATCCTTTAGGATATGATACAGCTTTAAAAATACCTTTCTATCCAAGTCTATTATGTTTAGATATTAAAGGATTTAGTAATGTATTAGTATTATACTTAGCTCAAAGTTTATTTGGTATACT | 413 | South_America |
| *Haemoproteus* | ASOT03 | EU627836 | Partial | 1 | CULPIC02 | ATTATATTTCATACCTGGACTTGTTTCATGGATTTGTGGTGGATATAATATTAGTGATCCTACTTTAAAAAGATTCTTTGTATTACATTTTATATTTCCATTTATAGCTTTATGTATTGTATTTATACATATATTCTTTTTACACTTACAAGGTAGCTCTAATCCTTTAGGATATGATACAGCTTTAAAAATACCTTTCTATCCAAGTCTATTATGTTTAGATATTAAAGGATTTAGTAATGTATTAGTATTATACTTAGCTCAAAGTTTATTTGGTATACT | 282 | Europe, North_America |
| *Haemoproteus* | ATLBRU01 | MH457416 | Full | 1 | ARBRU01 | TACCGGTGCTACATTTGTTTTTATTCTAACTTACTTACATATATTAAGAGGATTAAATTATTCATATTCTTATTTACCTTTATCATGGATAACTGGATTATTTATATTCTTAATTTCAATTGTTACCGCTTTTATGGGTTATGTATTACCTTGGGGTCAAATGAGTTTCTGGGGTGCAACCGTTATAACTAATTTATTATATTTTATTCCTGGACTTGTTTCATGGATTTGTGGAGGATATACTATTAGTGATCCAACTTTAAAAAGATTTTTTGTATTACATTTTATATTTCCTTTTATAGCTTTATGTATTGTATTTATACATATATTCTTCTTACACTTACAAGGTAGCTCTAATCCTTTAGGATATGATACAGCTTTAAAAATACCTTTCTATCCAAGTCTATTATGTCTAGATATCAAAGGATTTAATAATGTATTAGTCCTATTTCTAGCACAAAGTTTATTTGGAATTCT | 477 | South_America |
| *Haemoproteus* | ATLPIL02 | MH091775 | Full | 1 | RBQ16 | AACTGGTGCTTCATTTGTATTTATTTTAATTTATTTACATATTTTAAGAGGATTAAACTATTCATATTCATATTTACCTTTATCATGGATGTCTGGATTAATTATATTTTTAATATCTATTGTAACAGCTTTTATGGGTTATGTATTACCTTGGGGTCAAATGAGTTTCTGGGGTGCTACAGTTATTACTAATTTATTATATTTTATACCTGGACTTGTTTCATGGATATGTGGTGGATATCTTGTAAGTGACCCAACCTTAAAAAGATTCTTTGTATTACATTTTACATTTCCATTTATAGCTTTATGTATTGTATTTATACATATATTCTTTTTACATTTACAAGGTAGCACAAATCCTTTAGGGTATGATACAGCTTTAAAAATACCCTTCTATCCAAATCTTTTAAGTCTTGATATTAAAGGATTTAATAATGTATTAGTATTATTTTTAGCACAAAGTTTATTTGGAATATT | 477 | Central_America |
| *Plasmodium* | BAEBIC04 | MF817785 | Full | 1 | BAEBIC01 | GCAACAGGTGCTTCATTTGTATTTATTTTAACTTATTTACATATTTTAAGAGGATTAAATTATTCATATTCATATTTACCTTTATCATGGATATCTGGACTAATTATATTTTTAATATCTATTGTAACAGCTTTTATGGGTTATGTATTACCTTGGGGTCAAATGAGTTTCTGGGGTGCTACAGTTATTACTAATTTATTATATTTTATACCTGGACTTGTTTCATGGATATGTGGTGGATATCTTGTAAGTGACCCAACCTTAAAAAGATTCTTTGTATTACATTTTACATTTCCATTTATAGCCTTATGTATTGTATTTATACATATATTCTTTTTACATTTACAAGGTAGCACAAATCCTTTAGGGTATGATACAGCTTTAAAAATACCCTTCTATCCAAATCTTTTAAGTCTTGATATTAAAGGATTTAATAATGTATTAGTATTATTTTTAGCACAAAGTTTATTTGGAATACT | 479 | North_America |
| *Plasmodium* | BAHYP01 | JX029875 | Full | 1 | VOLJAC01 | CAACAGGTGCTTCATTTGTTTTCATTCTAACCTATTTACATATTTTAAGAGGATTAAATTATTCATACTCATATTTACCTTTATCATGGATTTCAGGATTATTAATATTTCTAATATCTATAGTTACTGCTTTTATGGGTTATGTATTACCTTGGGGTCAAATGAGTTTCTGGGGTGCTACAGTTATAACTAATTTATTATATTTTATACCTGGACTTGTCTCATGGATTTGTGGTGGATATCTTGTAAGTGACCCAACTTTAAAAAGATTTTTCGTATTACATTTTACATTTCCATTTATAGCTTTATGTATTGTATTTATACATATATTCTTCTTACATTTACAAGGTAGCACAAATCCTTTAGGGTATGATACAGCTTTAAAAATACCCTTCTATCCAAATCTATTAAGTCTTGATATTAAAGGATTTAATAATGTATTAGTTTTATTCTTATCTCAAAGTTTATTTGGAATTTT | 478 | South_America |
| *Plasmodium* | BAERID01 | MK216265 | Full | 1 | BAEBIC01 | CAACAGGTGCTTCATTTGTATTTATTTTAACTTATTTACATATTTTAAGAGGATTAAATTATTCATATTCATATTTACCTTTATCATGGATATCTGGACTAATTATATTTTTAATATCTATTGTAACAGCTTTTATGGGTTATGTATTACCTTGGGGTCAAATGAGTTTCTGGGGTGCTACAGTTATTACTAATTTATTATATTTTATACCTGGACTTGTTTCATGGATATGTGGTGGATATCTTGTAAGTGACCCAACCTTAAAAAGATTCTTTGTATTACATTTTACATTTCCATTTATAGCCTTATGTATTGTATTTATACATATATTCTTTTTACATTTACAAGGTAGCACAAATCCTTTAGGGTATGATACAGCTTTAAAAATACCCTTCTATCCAAATCTTTTAAGTCTTGATATTAAAGGATTTAATAATGTATTAATATTATTTTTAGCACAAAGTTTATTTGGAATACT | 478 | North_America |
| *Haemoproteus* | BLUTI09 | HQ123333 | Full | 1 | LARSCO01 | CTACTGGTGCTACATTTGTATTTATTTTAACATATTTACATATTCTAAGAGGATTAAATTACTCATATTCATATTTACCTTTATCATGGATAACTGGATTAGTAATATTCTTAATTTCAATTGTTACTGCTTTTATGGGTTATGTTTTACCTTGGGGTCAAATGAGTTTCTGGGGTGCAACCGTTATTACTAATTTATTATATTTTATACCTGGACTTGTTTCATGGATTTGTGGTGGATATACAATAAGTGATCCAACCTTAAAAAGATTTTTTGTATTACATTTTATATTTCCTTTTATAGCCTTATGTATTGTGTTTATTCATATATTCTTCTTACATTTACAAGGTAGCTCTAATCCTTTAGGATATGATACAGCTTTAAAAATACCTTTCTATCCAAGTCTATTATGTTTAGATATTAAAGGATTTAATAATGTATTAGTCATATTTTTAGCACAAAGTTTATTTGGTATTCT | 478 | Europe |
| *Haemoproteus* | BNOW03 | EU627830 | Full | 1 | FASPA01 | GCTACTGGCGCTACATTTGTATTCATTTTAACTTATCTACATATTCTAAGAGGATTAAATTATTCATATGTATATTTACCTTTATCATGGATAACTGGATTAATTATATTCTTAATCTCTATAGTTACTGCTTTTATGGGTTATGTTCTACCTTGGGGTCAAATGAGTTTCTGGGGTGCAACAGTTATTACTAATTTATTATACTTCATACCTGGACTAGTATCATGGATTTGTGGTGGATATACTATTAGTGACCCTACTTTAAAAAGATTCTTTGTATTACACTTTATATTCCCATTTATAGCCTTATGTATCGTATTTATACATATATTCTTCTTACATCTACAAGGTAGCTCTAATCCTTTAGGATATGATACAGCTTTAAAAATACCTTTCTATCCAAGTCTATTATGTTTAGATGTTAAAGGATTTAATAATGTATTAGTCTTATTCTTAGCACAAAGTTTATTTGGTATTCT | 479 | North_America, Central_America |
| *Haemoproteus* | BONUM01 | EU254555 | Partial | 1 | STTRA01 | GCTACTGGTGCTACATTTGTTTTTATATTAACATACTTACATATCTTAAGAGGATTAAATTATTCATATTCATACTTACCTTTATCATGGATAACTGGATTAATGATTTTCTTAATTTCTATTGTTACTGCTTTTATGGGTTATGTATTACCTTGGGGTCAAATGAGTTTCTGGGGTGCAACCGTTATCACTAACTTATTATATTTTATACCTGGACTTGTTTCATGGATTTGTGGTGGATATAATATTAGTGATCCTACTTTAAAAAGATTCTTTGTATTACATTTTATATTTCCATTTGTAGCTTTATGTATTGTATTTATACATATATTCTTTTTACACTTACAAGGTAGCTCTAATC | 361 | North_America |
| *Plasmodium* | BSR2 | AF495549 | Full | 1 | RBQ16 | CAACTGGTGCTTCATTTGTATTTATTTTAACTTATTTACATATTTTAAGAGGATTAAATTATTCATATTCATATTTACCTTTATCATGGATATCTGGACTAGTCATATTTTTAATATCTATTGTAACAGCTTTTATGGGTTATGTATTACCTTGGGGTCAAATGAGTTTCTGGGGTGCTACTGTTATAACTAATTTATTATATTTTATACCTGGACTTGTTTCATGGATATGTGGTGGATATCTTGTAAGTGACCCAACCTTAAAAAGATTCTTTGTATTACATTTTACATTTCCATTTATAGCTTTATGTATTGTATTTATACATATATTCTTTTTACATTTACAAGGTAGCACAAATCCTTTAGGGTATGATACAGCTTTAAAAATACCCTTCTATCCAAATCTTTTAAGTCTTGATATTAAAGGATTTAATAATGTATTAGTATTATTTTTAGCACAAAGTTTATTTGGAATATT | 478 | South_Sahara |
| *Haemoproteus* | BUBBUB02 | MT281469 | Full | 1 | NINOX06 | GCTACTGGTGCTACATTTGTTTTTATATTAACATATTTACATATTTTAAGAGGATTAAATTATTCATATTCATATTTACCTTTATCATGGATAACTGGATTAATTATTTTTTTAATTTCTATTGTTACTGCTTTTATGGGTTATGTATTACCTTGGGGTCAAATGAGTTTCTGGGGTGCAACCGTTATTACTAACTTATTATATTTTATACCTGGACTTGTTTCATGGATTTGTGGTGGATATAATATTAGTGATCCTACTTTAAAAAGATTCTTTGTATTACATTTTATATTTCCATTTGTAGCTTTATGTATTGTATTTATACATATATTCTTTTTACACTTACAAGGTAGCTCTAATCCTTTAGGATATGATACAGCTTTAAAAATACCTTTCTATCCAAGTCTATTATGTTTAGATATTAAAGGATTTAGTAATATATTAGTATTATATTTAGCTCAAAGTTTATTTGGTATATT | 479 | Asia |
| *Plasmodium* | BUBBUB03 | MT281525 | Full | 1 | ZOSBRU01 | GCAACAGGTGCATCATTTGTATTTATTCTTACTTATTTACATATTTTGAGAGGATTAAATTACTCATACTCATATTTACCTTTATCATGGATATCTGGATTAATAATATTTTTAATATCAATAGTAACAGCTTTTATGGGATATGTATTACCTTGGGGTCAAATGAGCTTTTGGGGTGCAACTGTTATAACAAATTTACTATACTTTATTCCTGGTCTTGTTTCATGGATTTGTGGTGGATATCTTGTAAGTGACCCAACATTAAAAAGATTTTTTGTATTACATTTTATATTCCCATTTATAGCCTTATGTATTGTATTTATACATATTTTCTTTTTACATTTACAAGGTAGCACAAATCCTTTAGGGTATGATACAGCTTTAAAAATACCCTTCTATCCAAATCTATTAAGTCTTGATATTAAAGGATTTAATAATGTATTAGTTTTATTTTTATCACAAAGCTTATTTGGAATATT | 479 | Asia |
| *Leucocytozoon* | BUTJAM04 | DQ177269 | Full | 1 | BUTLIN01 | TCTACCGGTGCCTCATTTGTATTTATTCTAACATATCTACATATACTAAAAGGATTAAATTATTCATACTCTTACTTACCATTGTCATGGATTACTGGAGTTATGATATTTCTAATATCTATAGTAACTGCCTTCTTAGGTTATGTATTACCATGGGGCCAAATGAGTTTCTGGGGTGCAACTGTAATTACTAATTTACTATATTTTATTCCTGGACTAATCTCATGGGTATGTGGTGGATATGCTGTAGGCGACCTAACCTTAAAAAGATTTTTTGTATTACACTTTATTTTCCCATTTGTAGCATTAGCTATTGTATTTATACATATATTCTTTCTACATTTACAAGGTAGCAGTAATCCTTTAGGATATGATACTCCTTTAAAGATACCCTTCTATCCAAATCTATTATGTCTAGATATTAAAGGATTAACAAATATATTAATCTTATTCTTAGCTCAAAGCTTCTTTGGTATTTT | 479 | North_America |
| *Leucocytozoon* | BUTJAM09 | DQ177263 | Full | 1 | BUTLIN01 | TCTACCGGTGCCTCATTTGTATTTATTCTAACATATCTACATATACTAAAAGGATTAAATTATTCATACTCTTACTTACCATTGTCATGGATTACTGGAGTTATGATATTTCTAATATCTATAGTAACTGCCTTCTTAGGTTATGTATTACCATGGGGCCAAATGAGTTTCTGGGGTGCAACTGTAATTACTAATTTACTATATTTTATTCCTGGACTAATCTCATGGGTATGTGGTGGATATGCTGTAGGCGACCTAACCTTAAAAAGATTTTTTGTATTACACTTTATTTTCCCATTTGTAGCATTAGCTATTGTATTTATACATATATTCTTTCTACATTTACAAGGTAGCAGTAATCCTTTAGGATATGATACTCCTTTAAAGATACCCTTCTATCCAAATCTATTATGTCTAGATATTAAAGGATTAACAAATATATTAATCTTATTCTTAGCTCAAAGCTTCTTTGGAATTTT | 479 | North_America |
| *Leucocytozoon* | BUTJAM18 | HM142922 | Full | 1 | BUTLIN01 | TCTACCGGTGCCTCATTTGTATTTATTCTAACATATCTACATATACTAAAAGGATTAAATTATTCATACTCTTACTTACCATTGTCATGGATTACTGGAGTTATGATATTTCTAATATCTATAGTAACTGCCTTCTTAGGTTATGTATTACCATGGGGCCAAATGAGTTTCTGGGGTGCAACTGTAATTACTAATTTACTATATTTTATTCCTGGACTAATCTCATGGGTATGTGGTGGATATGCTGTAGGCGACCTAACCTTAAAAAGATTTTTTGTATTACACTTTATTTTCCCATTTGTAGCATTAGCTATTGTATTTATACATATATTCTTTCTACATTTACAAGGTAGCAGTAATCCTTTAGGATATGATACTCCTTTAAAGATACCCTTCTATCCAAATCTATTATGTCTAGATATTAAAGGATTGACAAATATATTAATCTTATTCTTAGCTCAAAGCTTCTTTGGTATTTT | 479 | - |
| *Leucocytozoon* | BUTREG01 | DQ177264 | Full | 1 | BUTLIN01 | TCTACCGGTGCCTCATTTGTATTTATTCTAACATATCTACATATACTAAAAGGATTAAATTATTCATACTCTTACTTACCATTGTCATGGATTACTGGAGTTATGATATTTCTAATATCTATAGTAACTGCCTTCTTAGGTTATGTATTACCATGGGGCCAAATGAGTTTCTGGGGTGCAACTGTAATTACTAATTTACTATATTTTATTCCTGGACTAATCTCATGGGTATGTGGTGGATATGCTGTAGGCGACCTAACCTTAAAAAGATTTTTTGTATTACACTTTATTTTCCCATTTGTAGCATTAGCTATTGTATTTATACATATATTCTTTCTACATTTACAAGGTAGCAGTAATCCTTTAGGATATGATACTCCTTTAAAGATACCCTTCTATCCAAATCTATTATGTCTAGATATTAAAGGATTAACAAATATATTAATCTTATTCTTAGCTCAAAGCTTCTTTGGCATTTT | 479 | North_America |
| *Leucocytozoon* | CALMIN01 | MG726162 | Full | 1 | AKGPL03 | TGCATCTTTTGTATTTATATTAACATATTTACACATATTAAGAGGATTAAATTATTCTTTCTGTTACTTACCTTTATCATGGATAAGTGGATTAGTAATATTTTTAATATTTATTGTAACTGCTTTTATGGGTTATGTATTACCATGGGGTCAAATGAGTTTTTGGGGAGCTACTGTAATTACTAATTTATTATATTTTATTCCTGGACTAATTAATTGGGTTTGTGGTGGATTTATTATTAATGACCCAACACTTAAAAGATTCTTTGTATTACATTTCATATTCCCATTTATAGCACTAGCTATTGTATTTATACATATATTCTTTCTACATATTCAAGGTAGCACTAATCCTTTAGGGTATGATACACCTTTAAAAATACCATTCTATCCAAATCTATTAACTTTAGATGTAAAAGGATTTAATTATGTATTAGTAATATTCTTATTTCAAAGTTTATTTGGAATTGC | 471 | North_America |
| *Leucocytozoon* | CARBAR01 | MK947661 | Full | 1 | ZOCAP05 | AACTGGTGCATCTTTTGTATTTATCTTAACATATCTACATATTCTAAGAGGATTAAACTATTCATTCTCTTACTTACCTTTATCATGGTATAGTGGTTTAGTTATATTCTTAATCTTTATTGTAACTGCTTTTATGGGTTACGTTTTACCATGGGGACAAATGAGTTTCTGGGGAGCAACTGTAATTACTAATTTATTATATTTTATTCCTGGATTAATTAATTGGGTCTGTGGTGGATTCATAATTAATGACCCAACATTAAAAAGATTCTTCGTATTACACTTTATATTCCCATTTATAGCCTTAGCTATTGTATTTATTCATATATTCTTCTTACATATTCATGGTAGCACTAATCCTTTAGGGTATGATACACCTTTAAAAATACCATTCTATCCAAATCTATTAACTTTAGATATTAAAGGATTTAACTATGTATTAGTTATATTTTTATTTCAAAGTTTATTTGGAATTGC | 477 | South_America |
| *Leucocytozoon* | CARBAR02 | MK947662 | Full | 1 | ZOCAP05 | AACTGGTGCATCTTTTGTATTTATCTTAACATATCTACATATTTTAAGAGGATTAAACTATTCATTCTCTTACTTACCTTTATCATGGTATAGTGGTTTAGTTATATTCTTAATCTTTATTGTAACTGCTTTTATGGGTTACGTTTTACCATGGGGACAAATGAGTTTCTGGGGAGCAACTGTAATTACTAATTTATTATATTTTATTCCTGGATTAATTAATTGGGTCTGTGGTGGATTCATAATTAATGACCCAACATTAAAAAGATTCTTCGTATTACACTTTATATTCCCATTTATAGCCTTAGCTATTGTATTTATTCATATATTCTTCTTACATATTCATGGTAGCACTAATCCTTTAGGGTATGATACACCTTTAAAAATACCATTCTATCCAAATCTATTAACTTTAGATATTAAAGGATTTAACTATGTATTAGTTATATTTTTATTTCAAAGTTTATTTGGAATTGC | 477 | South_America |
| *Haemoproteus* | CATANA01 | MN459001 | Full | 1 | MELGEO01 | CTACCGGTGCTACATTTGTTTTTATTCTAACTTACTTACATATATTAAGAGGGTTAAACTATTCATATTCATATTTACCTTTATCATGGATAACTGGATTATTTATATTCTTAATTTCAATTGTTACCGCTTTTATGGGTTATGTATTACCTTGGGGTCAAATGAGTTTCTGGGGTGCAACCGTTATTACTAATTTATTATATTTTATTCCTGGACTTGTTTCATGGATTTGTGGAGGATATACTATTAGTGATCCAACTTTAAAAAGATTTTTTGTATTACATTTTATATTTCCTTTTATAGCTTTATGTATTGTATTCATACATATATTCTTCTTACACTTACAAGGTAGCTCTAATCCTTTAGGATATGATACAGCTTTAAAAATACCTTTCTATCCAAGTCTATTATGTCTAGATATCAAAGGATTTAATAATGTATTAGTCCTATTTCTAGCACAAAGTTTATTTGGAATTCT | 478 | South_America |
| *Plasmodium* | CATFUS11 |  | Full | 1 | TUMIG05 | GCAACAGGTGCTTCATTTGTTTTCATTTTAACCTATTTACATATTTTAAGAGGATTAAATTATTCATATTCATATTTACCTTTATCATGGATTTCAGGATTATTAATATTTTTAATATCTATAGTAACAGCTTTTATGGGTTATGTATTACCTTGGGGTCAAATGAGTTTCTGGGGTGCTACTGTTATAACTAATTTATTATATTTTATACCTGGACTTGTCTCATGGATTTGTGGTGGATATCTTGTAAGTGACCCAACCTTAAAAAGATTTTTTGTATTACATTTTACATTCCCATTTATAGCTTTATGTATTGTATTTATACATATATTCTTCTTACATTTACAAGGTAGCACAAATCCTTTAGGGTATGATACAGCTTTAAAAATACCCTTCTATCCAAATCTATTAAGTCTTGATATTAAAGGATTTAATAATGTATTAGTTTTATTCTTATCTCAAAGTTTATTTGTAATTTT | 479 | - |
| *Leucocytozoon* | CATFUS17 | MK947817 | Full | 1 | CATBIC07 | TACAGGTGCATCTTTTGTTTTTATATTAACATATCTACATATCTTAAGAGGTTTAAATTATTCATTCTCTTATTTACCTTTATCATGGTATACAGGTTTAATAATATTCTTAATATTCATTGTAACTGCTTTTATGGGTTACGTATTACCATGGGGACAAATGAGTTTCTGGGGAGCAACTGTTATTACTAATTTATTATATTTTATTCCTGGATTAATCAATTGGGTATGTGGTGGATTTATTATTAATGATCCAACCCTAAAAAGATTCTTCGTATTACATTTTATATTCCCATTTGTAGCTTTAGCTATTGTATTTATTCATATATTCTTCTTACATATTCATGGTAGCACTAATCCTTTAGGGTATGATACACCTCTAAAAATACCATTCTATCCAAATCTATTAACTTTAGATATTAAAGGATTTAACTATGTATTAGTTATATTCTTATTCCAAAGTTTATTTGGAATTGC | 477 | - |
| *Leucocytozoon* | CATFUS18 | MK947818 | Full | 1 | CATMIN08 | CACAGGTGCATCTTTTGTATTTATATTAACATATCTTCATATCTTAAGAGGATTAAACTATTCTTTCTCTTACTTACCTTTATCATGGTATAGTGGTTTAATAATATTCTTAATTTTTATTGTAACTGCTTTCATGGGTTACGTCTTACCATGGGGACAAATGAGTTTCTGGGGAGCAACTGTAATTACTAATTTATTATATTTTATTCCTGGATTAATTAATTGGGTATGTGGTGGATTTATTATTAATGACCCAACACTAAAAAGATTTTTCGTATTACACTTCATATTCCCATTTATAGCATTAGCTATTGTATTTATTCATATATTCTTCTTACATATTCATGGTAGCACTAATCCTTTAGGGTATGATACACCTTTAAAAATACCATTCTATCCAAATCTATTAACTTTAGATATTAAAGGATTCAACTATGTATTAGTTATATTCTTATTTCAAAGTTTATTTGGAATTGC | 477 | - |
| *Leucocytozoon* | CATMIN05 | MG726119 | Full | 1 | CATUST11 | TCTACAGGTGCATCTTTTGTTTTTATATTAACATATCTACATATCTTAAGAGGTTTAAATTATTCATTCTCTTATTTACCTTTATCATGGTATACAGGTTTAATAATATTCTTAATATTCATTGTAACTGCTTTTATGGGTTACGTATTACCATGGGGACAAATGAGTTTCTGGGGAGCAACTGTTATTACTAATTTATTATATTTTATTCCTGGATTAATCAATTGGGTATGTGGTGGATTTATTATTAATGATCCAACCCTAAAAAGATTCTTCGTATTACATTTCATATTCCCATTTGTAGCTTTAGCTATTGTATTTATTCATATATTCTTCTTACATATTCATGGTAGCACTAATCCTTTAGGGTATGATACACCTCTAAAAATACCATTCTATCCAAATCTATTAACTTTAGATATTAAAGGATTTAACTATGTATTAGTTATATTCTTATTCCAAAGTTTATTTGGAATTGC | 479 | North_America |
| *Plasmodium* | CATUST05 | JQ026526 | Full | 1 | TUMIG05 | GCAACAGGTGCTTCATTTGTTTTCATTTTAACCTATTTACATATTTTAAGAGGATTAAATTATTCATATTCATATTTACCTTTATCATGGATTTCAGGATTATTAATATTTTTAATATCTATAGTAACAGCTTTTATGGGTTATGTATTACCTTGGGGTCAAATGAGTTTCTGGGGTGCTACTGTTATAACTAATTTATTATATTTTATACCTGGACTTGTCTCATGGATTTGTGGTGGATATCTTGTAAGTGACCCAACCTTAAAAAGATTTTTTGTATTACATTTTACATTCCCATTTATAGCTTTATGTATTGTATTTATACATATATTCTTCTTACATTTACAAGGTAGCACAAATCCTTTAGGGTATGATACAGCTTTAAAAATACCCTTCTATCCAAATCTATTAAGTCTTGATATTAAAGGATTTAATAATGTATTAGTTTTATTCTTATCTCAAAGTTTATTTGGAATTTT | 479 | South_Sahara, North_America, Central_America, South_America |
| *Plasmodium* | CATUST06 | JQ026525 | Full | 1 | CATUST01 | GCAACAGGTGCATCATTTGTCTTTATTCTTACATATTTACATATATTAAGAGGACTAAATTACTCTTATTCTTATTTACCTCTATCATGGATATCAGGATTAATAATATTTTTAATATCAATAGTAACAGCTTTTATGGGATATGTATTACCTTGGGGACAAATGAGTTTCTGGGGTGCAACAGTTATTACAAACTTATTATATTTTATACCTGGTCTTGTTTCATGGATATGTGGTGGATATCTTGTAAGTGACCCAACATTAAAAAGATTTTTTGTTTTACATTTTATATTTCCATTTATAGCTTTATGTATTGTATTTATACATATATTCTTTTTACATTTACAAGGTAGCACAAATCCTTTAGGGTATGATACAGCTTTAAAAATACCCTTCTATCCAAATCTATTAAGTCTTGATATTAAAGGATTTAATAATATCTTAGTATTATTTTTATCACAAAGTTTATTTGGAATATT | 479 | North_America, Central_America, South_America |
| *Plasmodium* | CERSER02 | JQ988579 | Full | 1 | TABI07 | CAACAGGTGCATCATTTGTATTTATTCTTACATATTTACATATTTTAAGAGGATTAGATTATTCTTATTCTTATTTACCTTTATCATGGATATCAGGATTAATAATATTTTTAATATCAATAGTTACTGCTTTTATGGGATATGTACTACCTTGGGGTCAAATGAGTTTCTGGGGTGCAACCGTCATTACTAATTTATTATATTTTATACCTGGTCTTGTTTCATGGATCTGTGGTGGATATCTTGTAAGCGACCCAACATTAAAAAGATTTTTTGTATTACATTTTATATTTCCATTTATAGCCTTATGTATTGTATTTATACATATATTCTTTCTACATTTACAAGGTAGCACAAATCCTTTAGGGTATGATACAGCTTTAAAAATACCCTTCTATCCAAATCTATTAAGTCTTGATATTAAAGGATTTAATAATATCTTAGTTTTATTTTTAGCACAAAGCTTATTTGGAATATT | 478 | South_America |
| *Haemoproteus* | CHRRID01 | ON950078 | Full | 1 | LARCAC01 | GCTACTGGTGCTACATTTGTCTTTATATTAACATATTTACATATCTTAAGAGGATTAAATTATTCATATTCATACTTACCTTTATCATGGATAACAGGATTAATTATTTTCTTAATTTCTATTGTTACTGCTTTTATGGGTTATGTGTTACCTTGGGGTCAAATGAGTTTCTGGGGTGCAACTGTTATTACTAACTTATTATATTTTATACCTGGACTTGTTTCATGGATTTGTGGTGGATATAATATTAGTGATCCTACTTTAAAAAGATTCTTTGTATTACATTTTATATTCCCATTTGTTGCTTTATGTATTGTATTTATACATATATTCTTTTTACACTTACAAGGTAGCTCTAATCCTTTAGGATATGATACAGCTTTAAAAATACCTTTCTATCCAAGTCTATTATGTTTAGATATTAAAGGATTTAGTAATGTATTAGTTTTATACTTAGCTCAAAGTTTATTTGGTATACT | 479 | Europe |
| *Plasmodium* | CINRUF01 | AF465553 | Full | 1 | SEIAUR02 | GCAACAGGTGCATCATTTGTATTTATTCTTACATATTTACATATTTTAAGAGGATTAAATTATTCTTATTCTTATTTACCTTTATCATGGATATCAGGATTAATAATATTTTTAATATCAATAGTTACTGCTTTTATGGGATATGTACTACCTTGGGGTCAAATGAGTTTCTGGGGTGCAACCGTCATTACTAATTTATTATATTTTATACCTGGTCTTGTTTCATGGATCTGTGGTGGATATCTTGTAAGCGACCCAACATTAAAAAGATTTTTTGTATTACATTTTATATTTCCATTTATAGCCTTATGTATTGTATTTATACATATATTCTTTCTACATTTACAAGGTAGCACAAATCCTTTAGGGTATGATACAGCTTTAAAAATACCCTTCTATCCAAATCTATTAAGTCTTGATATCAAAGGATTTAATAATATCTTAGTTTTATTCTTAGCACAAAGTTTATTTGGAATATT | 479 | Central_America |
| *Haemoproteus* | CIRCUM03 | KC994898 | Full | 1 | NECASP01 | CAACTGGTGCTACATTTGTCTTTATCTTAACTTATTTACATATACTAAGAGGATTAAATTATTCATATTCATATTTACCTTTATCATGGACAACTGGAATATTGATTTTCTTAATTTCTATTGTCACTGCTTTTATGGGTTATGTATTACCTTGGGGTCAAATGAGTTTCTGGGGTGCAACTGTTATAACTAATTTATTATATTTTATACCTGGACTTGTTTCTTGGATTTGTGGTGGATATATAATTAGTGATCCAACTTTAAAAAGATTTTTTGTATTACATTTTATATTCCCATTTATAGCTTTATGTATTGTATTTATACATATATTCTTTTTACACTTACAAGGTAGCTCTAATCCTTTAGGATATGATACTGCTTTAAAAATACCTTTCTATCCAAGTCTATTATGTCTAGATATTAAAGGATTTAATAATGTATTAGTCTTATTTCTAGCACAAAGTTTATTTGGAATTTT | 478 | Europe |
| *Leucocytozoon* | CLAHYE01 |  | Full | 1 | TUSW02 | TCAACTGGTGCATCTTTTGTATTTATTTTAACCTACTTACATATCTTAAGAGGATTAAATTATTCTTTCTGTTACTTACCTTTATCATGGAGTAGTGGTTTAATTATATTTTTAATATTTATTGTTACTGCTTTCATGGGATATGTTTTACCATGGGGTCAAATGAGTTTCTGGGGAGCAACAGTAATTACTAATTTATTATATTTTATTCCTGGATTAATAAATTGGGTTTGTGGTGGATTTATAATTAATGATCCAACATTAAAAAGATTTTTCGTATTACATTTTATATTCCCATTCGTAGCTTTAGCTATTGTATTTATACATATATTCTTCTTACATATACAAGGTAGCACTAATCCTCTAGGGTATGATACACCTTTAAAAATACCATTCTATCCAAGTCTATTAACTTTAGACATTAAAGGATTTAATTATGTATTTGTATTATTCCTATTTCAAAGTTTATTTGGAATTGC | 479 | - |
| *Haemoproteus* | COBRA01 | AF465573 | Full | 1 | NECASP01 | GCAACTGGTGCTACATTTGTCTTTATCTTAACTTATTTACATATACTAAGAGGATTAAATTATTCATATTCATATTTACCTTTATCATGGACAACTGGAATAGTTATTTTCTTAATTTCTATTGTCACTGCTTTTATGGGTTATGTATTACCTTGGGGTCAAATGAGTTTCTGGGGTGCAACCGTTATAACTAATTTATTATATTTTATACCTGGACTTGTTTCATGGATTTGTGGTGGATATATAATTAGTGATCCAACTTTAAAAAGATTTTTTGTATTACATTTTATATTCCCATTTATAGCTTTATGTATTGTATTTATACATATATTCTTTTTACACTTACAAGGTAGCTCTAATCCTTTAGGATATGATACTGCTTTAAAAATACCTTTCTATCCAAGTCTATTATGTCTAGATATTAAAGGATTTAATAATGTATTAGTCTTATTTCTAGCACAAAGTTTATTTGGAATTTT | 479 | North_America |
| *Leucocytozoon* | COCOR16 | LC230140 | Full | 1 | CORMAC01 | TCAACAGGTGCATCTTTTGTATTTATATTAACATACCTACATATTTTAAGAGGATTAAATTACTCTTTCTCTTACTTACCTTTATCATGGTATAGTGGTTTAATTATATTCTTAATCTTTATTGTAACTGCTTTTATGGGTTACGTTTTACCATGGGGACAAATGAGTTTCTGGGGAGCAACTGTAATTACTAATTTATTATATTTCATACCTGGACTAATTAATTGGGTCTGTGGTGGATTTATTATTAATGACCCAACACTAAAAAGATTCTTTGTATTACATTTTATATTCCCATTTGTAGCTTTAGCTATTGTATTTATTCATATATTCTTCTTACATATTCATGGTAGCACTAATCCTTTAGGGTATGATACACCTTTAAAAATACCATTCTATCCAAATCTATTAACCTTAGATGTTAAAGGATTTAACTATGTATTAGTTATATTTTTATTTCAAAGTTTATTTGGAATTGC | 479 | Asia |
| *Haemoproteus* | COEFLA01 | MN459460 | Full | 1 | PSADEC01 | CTACCGGTGCTACATTTGTTTTTATTTTAACTTACTTACATATCTTAAGAGGATTAAACTATTCATATTCATATTTACCTTTATCATGGATAACTGGATTAGTAATATTCTTAATTTCAATTGTTACCGCTTTTATGGGTTATGTATTACCTTGGGGTCAAATGAGTTTCTGGGGTGCAACCGTTATTACTAATTTATTATATTTTATACCTGGACTTGTTTCATGGATTTGTGGAGGATATACTATTAGTGATCCAACTTTAAAAAGATTCTTTGTATTACATTTTATATTTCCTTTTATAGCTTTATGTATTGTATTTATACATATATTCTTCTTACACTTACAAGGTAGCTCTAATCCTTTAGGATATGATACAGCTTTAAAAATACCTTTCTATCCAAGTCTATTATGTCTAGATATCAAAGGATTTAATAATGTATTAGTCCTATTTCTAGCACAAAGTTTATTTGGAATTCT | 478 | South_America |
| *Haemoproteus* | COLINC01 | FJ462680 | Partial | 1 | GEMON01 | GCCACAGGTGCATCATTTGTATTTATTTTAACATACCTACACATTTTAAGAGGATTAAATTACTCATATTCATATTTACCATTATCATGGATTACCGGATTAATAATATTTATTATCTCTATTATGACTGCTTTCTTAGGTTATGTTCTACCTTGGGGTCAAATGAGTTTCTGGGGTGCAACTGTTATTACTAATTTATTATATTTTATTCCAGGATTAGTCTCATGGATTTGTGGTGGATATATTGTTAGTGATCCTACACTAAAAAGATTCTTTGTATTACATTTTATATTTCCATTTATAGCTATATGTATAGTATTTATTCATATATTCTTTTTACATTTACAAGGTAGCTCTAATCCTTTTAGGATATGA | 375 | Central_America |
| *Plasmodium* | COLL7 | DQ368376 | Full | 1 | RBQ16 | GCAACTGGTGCTTCATTTGTATTTATTTTAACTTATTTACATATTTTAAGAGGATTAAATTATTCATATTCATATTTACCTTTATCATGGATATCTGGATTAATTATATTTTTAATATCTATAGTAACAGCTTTTATGGGTTATGTATTACCTTGGGGTCAAATGAGTTTCTGGGGTGCTACCGTAATTACTAATTTATTATATTTTATACCTGGACTAGTTTCATGGATATGTGGTGGATATCTTGTAAGTGACCCAACTTTAAAAAGATTCTTTGTATTACATTTTACATTTCCATTTATAGCTTTATGTATTGTATTTATACATATATTCTTTTTACATTTACAAGGTAGCACAAATCCTTTAGGGTATGATACAGCTTTAAAAATACCCTTCTATCCAAATCTTTTAAGTCTTGATATTAAAGGATTTAATAATGTATTAGTATTATTTTTAGCACAAAGTTTATTTGGAATATT | 479 | Europe, South_Sahara |
| *Plasmodium* | COLL13 |  | Full | 1 | CET01 | GCAACTGGTGCTTCATTTGTATTTATTTTAaCTTATTTACATATTTTAAGAGGATTAAATTATTCaTATTCATATTTACCTTTATCATGGATATCTGGATTAATAATATTTTTAATATCTATAGTAACAGCTTTTATGGGTTACGTATTACCTTGGGGTCAAATGAGTTTCTGGGGTGCTACCGTAATAACTAATTTATTATATTTTATACCTGGACTAGTTTCATGGATATGtGGtGGATATCTTGTAAGTGACCCAACCTTAAAAAGATTCTTTGTACTACATTTTACATTTCCTTTTATAGCTTTATGTATTGTATTCATACATATATTCTTTTTACATTTACAAGGtAGCACAAATCCTTTAGGGTATGATACAGCTTTAAAAATACCCTTCTATCCAAATCTTTTAAGTCTTGATATTAAAGGATTTAATAATGTATTAGTACTATTTTTAGCACAAAGTTTATTTGGAATACT | 479 | Europe |
| *Haemoproteus* | COLPAS03 | FJ462657 | Full | 1 | COLPAS01 | GCAACAGGTGCATCTTTTGTATTTATTTTAACATACTTACATATTCTAAGAGGATTGAATTATTCCTATTCATATTTACCATTATCATGGATTACCGGTTTATTAATATTTCTAATCTCTATTGTAACTGCTTTTATGGGTTACGTATTACCTTGGGGTCAAATGAGTTTCTGGGGTGCAACAGTTATTACTAATTTACTTTATTTTATACCTGGATTAGTCTCATGGATTTGTGGTGGATATATTGTTAGTGACCCTACCCTAAAAAGATTCTTTGTATTACATTTTATATTTCCTTTTATAGCTATATGTATAGTATTTATACATATATTCTTTCTACATTTACAAGGTAGCTCTAATCCTTTAGGATATGATACAGCTTTAAAAATACCCTTCTATCCAAGTCTATTATGCCTAGATATTAAAGGTTTTAATAACGTATTAGTATTATTCTTAGCTCAAAGCTTATTTGGAATATT | 479 | North_America, Central_America, South_America |
| *Haemoproteus* | COLPAS05 | JN788939 | Full | 1 | COLPAS01 | ATACTTACATATTCTAAGAGGATTGAATTATTCCTATTCATATTTACCATTATCATGCATTACCAGATTAAAAATATTGCTAATCTCTATTGTGACTGCTTTTATGGGTTACGTATTACCTTGGGGTCAAATGAGTTTCTGGGGTGCAACAGTTATTACTAATTTACTTTATTTTATACCTGGATTAGTCTCATGGATTTGTGGTGGATATATTGTTAGTGACCCTACCCTAAAAAGATTCTTTGTATTACATTTTATATTTCCTTTTATAGCTATATGTATAGTATTTATACATATATTCTTTCTACATTTACAAGGTAGCTCTAATCCTTTAGGATATGATACAGCTTTAAAAATACCCTTCTATCCAAGTCTATTATGCCTAGATATTAAAGGTTTTAATAACGTATTAGTATTATTCTTAGCTCAAAGCTTATTTGGAATATT | 447 | Central_America |
| *Haemoproteus* | COLPAS06 | KU562226 | Full | 1 | COLPAS01 | AACAGGTGCATCTTTTGTATTTATTTTAAAATACTTACATATTCTAAGAGGATTGAATTATTCCTATTCATATTTACCATTATCATGGATTACCGGTTTATTAATATTTCTAATCTCTATTGTAACTGCTTTTATGGGTTACGTATTACCTTGGGGTCAAATGAGTTTCTGGGGTGCAACAGTTATTACTAATTTACTTTATTTTATACCTGGATTAGTCTCATGGATTTGTGGTGGATATATTGTTAGTGACCCTACCCTAAAAAGATTCTTTGTATTACATTTTATATTTCCTTTTATAGCTATATGTATAGTATTTATACATATATTCTTTCTACATTTACAAGGTAGCTCTAATCCTTTAGGATATGATACAGCTTTAAAAATACCCTTCTATCCAAGTCTATTATGCCTAGATATTAAAGGTTTTAATAACGTATTAGTATTATTCTTAGCTCAAAGCTTATTTGGAATATT | 477 | South_America |
| *Leucocytozoon* | COLPAS09 | MZ604564 | Full | 1 | COLPAS01 | CAACAGGTGCATCTTTTGTATTTATTTTAACATACTTACATATTCTAAGAGGATTGAATTATTCCTATTCATATTTACCATTATCATGGATTACCGGATTATTAATATTTCTAATCTCTATTGTAACTGCTTTTATGGGTTACGTATTACCTTGGGGTCAAATGAGTTTCTGGGGTGCAACAGTTATTACTAATTTACTTTATTTTATACCTGGATTAGTCTCATGGATTTGTGGTGGATATATTGTTAGTGACCCTACCCTAAAAAGATTCTTTGTATTACATTTTATATTTCCTTTTATAGCTATATGTATAGTATTTATACATATATTCTTTCTACATTTACAAGGTAGCTCTAATCCTTTAGGATATGATACAGCTTTAAAAATACCCTTCTATCCAAGTCTATTATGCCTAGATATTAAAGGTTTTAATAACGTATTAGTATTATTCTTAGCTCAAAGCTTATTTGGAATATT | 478 | - |
| *Haemoproteus* | COPIC01 | KP686106 | Full | 1 | COLPIC01 | GCAACAGGTGCATCTTTTGTATTTATTTTAACATACTTACATATTCTAAGAGGTTTGAATTACTCTTATTCATATTTACCATTATCATGGATTACCGGATTAATAATATTTTTAATCTCTATTGTAACTGCTTTTATGGGTTACGTATTACCTTGGGGTCAAATGAGTTTCTGGGGTGCAACTGTTATTACTAATTTACTTTATTTTATTCCTGGATTAGTCTCATGGATTTGTGGTGGATATATTGTTAGTGACCCAACCCTAAAAAGATTTTTTGTATTACATTTTATATTTCCATTTATAGCTATATGTATAGTATTTATACATATATTCTTTCTACATTTACAAGGTAGCTCTAATCCTTTAGGATATGATACAGCTTTAAAAATACCCTTCTATCCAAGTCTATTATGCCTAGATATTAAAGGTTTTAATAACGTATTAGTTTTATTCTTAGCTCAAAGTTTATTTGGAATATT | 479 | Central_America, South_America |
| *Haemoproteus* | COLTAL01 | GU296214 | Full | 1 | ZEGAL04 | GCCACAGGTGCATCATTTGTATTTATTTTAACATACCTACATATTTTAAGAGGATTAAATTACTCATATTCATATTTACCATTATCATGGATTACCGGATTAATAATATTTATAATCTCTATAATGACTGCTTTCTTGGGTTATGTTCTACCTTGGGGTCAAATGAGTTTCTGGGGTGCAACTGTTATTACTAATTTACTATATTTTATTCCGGGATTAGTATCATGGATTTGTGGTGGTTATATAGTTAGTGATCCTACACTAAAAAGATTCTTTGTATTACATTTTATATTTCCATTTATAGCTATATGTATAGTATTTATACATATATTCTTTTTACATTTACAAGGTAGCTCTAATCCTCTAGGATATGATACAGCATTAAAAATACCATTTTACCCAAATCTACTATGTTTAGATATAAAAGGATTTGATAACGTATTAGTATTATTCTTAGCTCAAAGCTTATTTGGAATATT | 479 | Central_America, South_America |
| *Leucocytozoon* | CORCAU02 | MG765396 | Partial | 1 | JUHYE21 | TATTAACATACTTACATATATTAAGAGGATTAAATTATTCATTTACTTACTTACCTTTATCATGGATAAGTGGTTTAATAATATTCTTAATATTTATTGTAACTGCTTTTATGGGTTATGTCTTACCATGGGGTCAAATGAGTTTTTGGGGAGCTACTGTTATAACTAATTTATTATATTTTATTCCTGGATTAATTAATTGGGTTTGCGGTGGATTTATTATTAATGACCCAACTCTAAAAAGATTCTTCGTATTACATTTTATATTCCCATTTGTAGCATTAGCTATCGTATTTATACATATATTCTTCTTACATATTCAAGGTAGCACTAATCCTTTAGGGTATGATACACCTTTAAAAATACCATTCTATCCAAATCTATTAACTTTAGATGTTAAAGGATTTAACTATGTATTAGTATTA | 425 | North_America |
| *Plasmodium* | CORCAU05 | MG765401 | Partial | 1 | DENCOR09 | TTCTAACTTATTTACATATTTTAAGAGGATTAAATTATTCATATTCATATTTACCTTTATCATGGATATCTGGATTATTTATATTCTTAATATCTATAGTTACAGCTTTTATGGGTTATGTATTACCTTGGGGTCAAATGAGTTTCTGGGGTGCAACTGTAATTACTAATCTATTATATTTTATACCTGGACTTGTTTCATGGATTTGTGGTGGATATCTTGTAAGTGACCCAACATTAAAAAGATTCTTTGTATTACATTTTACATTTCCATTTATAGCTTTATGTATTGTATTTATACATATATTCTTTCTACATTTACAAGGTAGCACTAATCCTTTAGGGTATGATACAGCTTTAAAAATACCCTTCTATCCAAATCTTTTAAGTCTCGATATTAAAGGATTTAATAATGTATTAGTATTA | 425 | North_America |
| *Haemoproteus* | COSQU04 | KP686099 | Full | 1 | COLSQU01 | GCAACTGGTGCATCTTTTGTATTTATTTTAACATACTTACATATTCTAAGAGGATTGAATTACTCCTATTCATATTTACCATTATCATGGATTACTGGATTAATAATATTTCTAATCTCTATTGTAACTGCTTTTATGGGTTACGTATTACCTTGGGGTCAAATGAGTTTCTGGGGTGCAACAGTTATTACTAATTTACTTTATTTTATTCCTGGATTAGTCTCATGGATTTGTGGTGGATATATTGTTAGTGACCCTACCCTAAAAAGATTCTTTGTATTACATTTTATATTTCCTTTTATAGCTATATGTATAGTATTTATACATATATTCTTTCTACATTTACAAGGTAGCTCTAATCCTTTAGGATATGATACAGCTTTAAAAATACCCTTCTATCCAAGTCTATTATGCCTAGATATTAAAGGTTTTAATAACGTATTAGTTCTATTCTTAGCTCAAAGCTTATTTGGAATATT | 479 | South_America |
| *Haemoproteus* | CULCIR01 | KC815468 | Full | 1 | NECASP01 | CAACTGGTGCTACATTTGTCTTTATCTTAACTTATTTACATATATTAAGAGGATTAAATTATTCATATTCATATTTACCTTTATCATGGACAACTGGAATATTGATTTTCTTAATTTCTATTGTCACTGCTTTTATGGGTTATGTATTACCTTGGGGTCAAATGAGTTTCTGGGGTGCAACTGTTATAACTAATTTATTATATTTTATACCTGGACTTGTTTCTTGGATTTGTGGTGGATATATAATTAGTGATCCAACTTTAAAAAGATTTTTTGTATTACATTTTATATTCCCATTTATAGCTTTATGTATTGTATTTATACATATATTCTTTTTACACTTACAAGGTAGCTCTAATCCTTTAGGATATGATACTGCTTTAAAAATACCTTTCTATCCAAGTCTATTATGTCTAGATATTAAAGGATTTAATAATGTATTAGTCTTATTTCTAGCACaAAGttTAtTTGgaATttt | 478 | Europe |
| *Haemoproteus* | CULPIC02 | PP003132 | Full | 1 | ASOT03 | GCTACTGGTGCTACATTTGTTTTTATATTAACATATTTACATATCTTAAGAGGATTAAATTATTCATATTCATACTTACCTTTATCATGGATTACTGGATTAATCATTTTCTTAATTTCTATTGTCACTGCCTTTATGGGTTATGTATTACCTTGGGGTCAAATGAGTTTCTGGGGTGCAACCGTTATTACTAACTTATTATATTTCATACCTGGACTTGTTTCATGGATTTGTGGTGGATATAATATTAGTGATCCTACTTTAAAAAGATTCTTTGTATTACATTTTATATTTCCATTTATAGCTTTATGTATTGTATTTATACATATATTCTTTTTACACTTACAAGGTAGCTCTAATCCTTTAGGATATGATACAGCTTTAAAAATACCTTTCTATCCAAGTCTATTATGTTTAGATATTAAAGGATTTAGTAATGTATTAGTATTATACTTAGCTCAAAGTTTATTTGGTATACT | 479 | Europe |
| *Plasmodium* | CURCUR01 | MK695443 | Full | 1 | ELALB02 | AACAGGTGCATCATTTGTATTTATTCTTACATATTTACATATTTTAAGAGGATTAAATTATTCTTATTCTTATTTACCTTTATCATGGATATCAGGATTAATAATATTTTTAATATCAATAGTTACTGCTTTTATGGGATATGTACTACCTTGGGGTCAAATGAGTTTCTGGGGTGCAACCGTCATTACTAATTTACTATATTTTATACCTGGTCTTGTTTCATGGATCTGTGGTGGATATCTTGTAAGCGACCCAACATTAAAAAGATTTTTTGTATTACATTTTATATTTCCATTTATAGCCTTATGTATTGTATTTATACATATATTCTTTCTACATTTACAAGGTAGCACTAATCCTTTAGGGTATGATACAGCTTTAAAAATACCCTTCTATCCAAATCTATTAAGTCTTGATATTAAAGGATTTAATAATATCTTAGTTTTATTCTTAGCACAAAGTTTATTTGGAATATT | 477 | South_America |
| *Plasmodium* | CXPIP01 | GQ471939 | Partial | 1 | RBQ18 | CAACAGGTGCATCATTTGTATTTATTCTTACTTATTTACATATTTTAAGAGGATTAAATTATTCTTACTCATATTTACCATTATCATGGATATCAGGATTAATAATATTTTTAATATCAATAGTTACAGCTTTTATGGGATATGTATTACCTTGGGGTCAAATGAGTTTCTGGGGTGCAACTGTTATTACTAATTTATTATATTTTATACCTGGTCTTGTTTCATGGATTTGTGGTGGATATCTTGTTAGTGATCCAACATTAAAAAGATTTTTTGTTTTACACTTTATATTTCCATTTATAGCTTTATGTATTGTATTCATACATATATTCTT | 334 | - |
| *Plasmodium* | CXPIP23 | JF411405 | Full | 1 | CXTHE01 | GCAACTGGAGCTTCATTTGTATTTATCTTAACTTACTTACACATTTTAAGAGGATTAAATTATTCATATTCATACTTACCTTTATCATGGATATCAGGATTAATGATATTTTTAATATCAATAGTTACAGCTTTTATGGGTTATGTATTACCTTGGGGTCAAATGAGTTTCTGGGGTGCAACTGTTATCACTAATTTATTATATTTTATCCCTGGACTTGTTTCATGGATTTGTGGTGGATATCTTGTAAGTGACCCAACTTTAAAAAGATTCTTTGTATTACATTTTACATTTCCATTTATAGCTTTATGTATTGTATTTATACATATATTCTTTCTACATTTACAAGGTAGCACTAATCCTTTAGGGTATGATACAGCTTTAAAAATACCCTTCTATCCAAATCTATTAAGTCTCGACATAAAAGGATTTAATAATGTATTAGTCTTATTTTTAGCACAAAGCTTATTTGGAATCTT | 479 | Europe, North_Africa_._Middle_East |
| *Haemoproteus* | CXPIP27 | KJ579154 | Full | 1 | COCOR01 | GCAACTGGTGCTACATTTGTCTTTATCTTAACTTATCTACATATACTAAGAGGATTAAATTATTCATATTCATATTTACCTTTATCATGGACAACTGGAATATTAATTTTCTTAATTTCTATTGTCACTGCTTTTATGGGTTATGTATTACCTTGGGGTCAAATGAGTTTCTGGGGTGCAACCGTTATAACTAATTTATTATATTTTATACCTGGACTTGTTTCATGGATTTGTGGTGGATATATAATTAGTGATCCAACTTTAAAAAGATTTTTTGTATTACATTTTATATTTCCATTTATAGCTTTATGTATTGTATTTATACATATATTCTTTTTACACTTACAAGGTAGCTCTAATCCTTTAGGATATGATACTGCTTTAAAAATACCTTTCTATCCAAGTCTATTATGTCTAGATATTAAAGGATTTAATAATGTATTAGTCTTATTTCTAGCACAAAGTTTATTTGGAATTTT | 479 | Europe |
| *Plasmodium* | CXPOI01 | HM179151 | Full | 1 | HALVOC01 | GGTGCATCATTTGTATTTATTCTTACCTATTTACATATTTTAAGAGGATTAAATTATTCTTATTCATATCTACCTTTATCTTGGATATCAGGATTAATAATATTTTTAATATCAATAGTAACTGCTTTTATGGGATATGTTTTACCTTGGGGACAAATGAGTTTTTGGGGTGCAACTGTTATAACTAACTTATTATATTTTATACCTGGTCTTGTTTCATGGATTTGTGGTGGATATCTTGTAAGCGACCCAACCTTAAAAAGATTTTTTGTATTACATTTTATATTTCCATTTATAGCTTTATGTATTGTATTTATACATATATTCTTTTTACATTTACAAGGTAGCACAAATCCTTTAGGGTATGATACAGCTTTAAAAATACCCTTCTATCCAAATCTATTAAGTCTTGATATTAAAGGATTTAATAATATTTTAGTTTTATTTTGAGCACAAAGTTTATT | 464 | South_Sahara |
| *Plasmodium* | CXRES01 | GQ471946 | Partial | 1 | AFTRU07 | CAACAGGTGCATCATTTGTATTTATTCTTACTTATTTACATATTTTAAGAGGATTAAATTATTCTTATTCATATTTACCATTATCATGGATATCAGGATTAATAATATTTTTAATATCAATAGTTACAGCTTTTATGGGATATGTATTACCTTGGGGTCAAATGAGTTTCTGGGGTGCAACTGTTATTACTAATTTATTATATTTTATACCTGGTCTTGTTTCATGGATTTGTGGTGGATATCTTGTTAGTGATCCAACATTAAAAAGATTTTTTGTTTTACATTTTATATTTCCATTTATAGCTTTATGTATTGTATTCATACATATATTCTTTCTACATCTACAAGGTAGCACAAAT | 359 | - |
| *Plasmodium* | CXRES06 | KY639439 | Full | 1 | TABI07 | GCAACAGGTGCATCATTTGTATTTATTCTTACATATTTACATATTTTAAGAGGATTAAATTATTCTTATTCTTATTTACCTTTATCATGGATATCAGGATTAATAATATTTTTAATATCAATAGTTACTGCTTTTATGGGATATGTACTACCTTGGGGTCAAATGAGTTTCTGGGGTGCAACTGTCATTACTAATTTATTATATTTTATACCTGGTCTTGTTTCATGGATCTGTGGTGGATATCTTGTAAGCGACCCAACATTAAAAAGATTTTTTGTATTACATTTTATATTTCCATTTATAGCCTTATGTATTGTATTTATACATATATTCTTTCTACATTTACAAGGTAGCACAAATCCTTTAGGGTATGATACAGCTTTAAAAATACCCTTCTATCCAAATCTATTAAGTCTTGATATTAAAGGATTTAATAATATCTTAGTTTTATTTTTAGCACAAAGCTTATTTGGAATATT | 479 | Central_America |
| *Haemoproteus* | CYACRI01 | MF817763 | Full | 1 | NECASP01 | GCAACTGGTGCTACATTTGTCTTTATCTTAACTTATTTACATATACTAAGAGGATTAAATTATTCATATTCATATTTACCTTTATCATGGACAACTGGAATATTGATTTTCTTAATTTCTATTGTCACTGCTTTTATGGGTTATGTATTACCTTGGGGTCAAATGAGTTTCTGGGGTGCAACCGTTATAACTAATTTATTATATTTTATACCTGGACTTGTTTCATGGATTTGTGGTGGATATATAATTAGTGATCCAACTTTAAAAAGATTTTTTGTATTACATTTTATATTCCCATTTATAGCTTTATGTATTGTATTTATACATATATTCTTTTTACACTTACAAGGTAGCTCTAATCCTTTAGGATATGATACTGCTTTAAAAATACCTTTCTATCCAAGTCTATTATGTCTAGATATTAAAGGATTTAATAATGTATTAGTCTTATTTCTAGCACAAAGTTTATTTGGAATTTT | 479 | North_America |
| *Haemoproteus* | CYAMEL01 | OR063112 | Full | 1 | NECASP01 | AACTGGTGCTACATTTGTCTTTATCTTAACTTATTTACATATACTAAGAGGATTAAATTATTCATATTCATATTTACCTTTATCATGGATAACTGGAATATTTATTTTCTTAATTTCTATTGTCACTGCTTTTATGGGTTATGTATTACCTTGGGGTCAAATGAGTTTCTGGGGTGCAACCGTTATAACTAATTTATTATATTTTATACCTGGACTTGTTTCATGGATTTGTGGTGGATATATAATTAGTGATCCAACTTTAAAAAGATTTTTTGTATTACATTTTATATTCCCATTTATAGCTTTATGTATTGTATTTATACATATATTCTTTTTACACTTACAAGGTAGCTCTAATCCTTTAGGATATGATACTGCTTTAAAAATACCTTTCTATCCAAGTCTATTATGTCTAGATATTAAAGGATTTAATAATGTATTAGTCTTATTTCTAGCACAAAGTTTATTTGGAATTTT | 477 | Central_America |
| *Haemoproteus* | CYAOLI03 | EU810741 | Full | 1 | NEOBS03 | GCAACCGGTGCTACATTTGTTTTTATTTTAACTTACTTACATATATTAAGAGGATTAAACTATTCATATTCTTATTTACCTTTATCATGGATAACAGGATTAGTAATATTCTTAATTTCTATTGTTACCGCTTTTATGGGTTATGTATTACCTTGGGGTCAAATGAGTTTCTGGGGTGCAACCGTTATTACTAATTTATTATATTTTATACCTGGACTTGTTTCATGGATTTGTGGAGGATATACTATTAGTGATCCAACTCTAAAAAGATTTTTTGTATTACATTTTATATTCCCTTTTATAGCTTTATGTATTGTATTTATACATATATTCTTTTTACACTTACAAGGTAGCTCTAATCCTTTAGGATATGATACAGCTTTAAAAATACCTTTCTATCCAAGTCTATTATGTCTAGATATTAAAGGATTTAATAATGTATTAGTCCTATTTCTAGCACAAAGTTTATTTGGAATTCT | 479 | South_Sahara |
| *Plasmodium* | CYAOLI04 | EU810652 | Full | 1 | NEOLI01 | GCAACAGGTGCATCATTTGTATTTATTCTTACTTATTTACATATTTTAAGAGGATTAAACTATTCTTATTCATATTTACCTTTATCATGGATGTCAGGATTAATAATATTTTTAATATCAATAGTAACTGCTTTTATGGGATATGTATTACCTTGGGGTCAAATGAGTTTCTGGGGTGCAACTGTTATTACCAACTTACTCTACTTTATACCTGGTCTTGTTTCATGGATTTGTGGTGGATATCTTGTAAGTGATCCAACATTAAAAAGATTTTTTGTATTACATTTTATATTTCCATTTATAGCTTTATGTATTGTGTTTATACATATATTCTTTCTACATTTACAAGGTAGCACAAATCCTTTAGGATATGATACAGCTTTAAAAATACCCTTCTATCCAAATCTATTAAGTCTTGATATTAAAGGATTTAATAATATCTTAGTTTTATTTTTAGCACAAAGTTTATTTGGAATATT | 479 | South_Sahara, Central_America |
| *Haemoproteus* | CYAOLI05 | FJ404696 | Full | 1 | NEOBS04 | GCTACCGGTGCTACATTTGTTTTTGTTTTAACTTACTTACATATATTAAGAGGACTAAATTATTCATACTCTTATTTACCTCAATCATGGATAACTGGATTAATAATATTCTTAATTTCTATTGTTACAGCTTTTATGGGTTATGTATTACCTTGGGGTCAAATGAGTTTCTGGGGTGCAACCGTTATTACTAATTTATTATATTTTATACCTGGACTTGTTTCATGGATTTGTGGAGGATATACTATTAGTGATCCAACTCTAAAAAGATTCTTTGTATTACATTTTATATTTCCTTTTGTAGCTTTATGTATTGTATTTATACATATATTCTTTTTACACTTACAAGGTAGCTCTAATCCTTTAGGATATGATACAGCTTTAAAAATACCTTTCTATCCAAGTCTATTATGTCTAGATATTAAAGGATTTAATAATGTATTAGTCCTATTTCTAGCACAAAGTTTATTTGGTATTCT | 479 | South_Sahara |
| *Plasmodium* | CYAOLI09 | FJ404707 | Full | 1 | NEOBS02 | GCAACAGGTGCTTCATTTGTATTTATTTTAACTTATTTACATATTTTAAGAGGATTAAATTATTCATATTCATATTTACCTTTATCATGGATATCAGGATTAATAATATTCTTAATATCTATAGTAACAGCCTTTATGGGTTATGTACTACCATGGGGTCAAATGAGTTTTTGGGGTGCAACTGTAATCACTAATTTATTATACTTTATACCTGGACTCGTTTCATGGATATGTGGAGGATATCTTGTAAGTGACCCAACTTTAAAAAGATTCTTTGTATTACATTTTATATTCCCATTTATAGCTTTATGTATTGTATTTATACATATATTCTTTTTACATCTACAAGGTAGCACAAATCCTTTAGGGTATGATACAGCTTTAAAAATACCCTTCTATCCAAATCTTTTAAGTCTTGATATAAAAGGATTTAATAATATATTAGTATTATTCTTATCACAAAGTTTATTTGGAATATT | 479 | South_Sahara |
| *Plasmodium* | CYAOLI13 | KJ446983 | Partial | 1 | NEOBS02 | TTTATTTTAACTTATTTACATATTTTAAGAGGATTAAATTATTCATATTCATATTTACCTTTATCATGGATATCAGGATTAATAATATTCTTAATATCTATATTAACAGCCTTTATGGGTTATGTACTACCATGGGGTCAAATGAGTTTTTGGGGTGCAACTGTAATCACTAATTTATTATACTTTATACCTGGACTCGTTTCATGGATATGTGGAGGATATCTTGTAAGTGACCCAACTTTAAAAAGATTCTTTGTATTACATTTTATATTCCCATTTATAGCTTTATGTATTGTATTTATACATATATTCTTTTTACATCTACAAGGTAGCACAAATC | 340 | - |
| *Haemoproteus* | CYASTE05 | MF077657 | Full | 1 | NECASP01 | CAACTGGTGCTACATTTGTCTTTATCTTAACTTATTTACATATACTAAGAGGATTAAATTATTCATATTCATATTTACCTTTATCATGGACAACTGGAATATTTATTTTCTTAATTTCTATTGTCACTGCTTTTATGGGTTATGTATTACCTTGGGGTCAAATGAGTTTCTGGGGTGCAACCGTTATAACTAATTTATTATATTTTATACCTGGACTTGTTTCATGGATTTGTGGTGGATATATAATTAGTGATCCAACTTTAAAAAGATTTTTTGTATTACATTTTATATTCCCATTTATAGCTTTATGTATTGTATTTATACATATATTCTTTTTACACTTACAAGGTAGCTCTAATCCTTTAGGATATGATACTGCTTTAAAAATACCTTTCTATCCAAGTCTATTATGTCTAGATATTAAAGGATTTAATAATGTATTAGTCTTATTTCTAGCACAAAGTTTATTTGGAATTTT | 478 | North_America |
| *Haemoproteus* | CYAYNC01 | MN459542 | Full | 1 | NECASP01 | CAACTGGTGCTACATTTGTCTTTATCTTAACTTATTTACATATACTAAGAGGATTAAATTATTCATATTCATATTTACCTTTATCATGGATAACTGGAATATTAATTTTCTTAATTTCTATTGTCACTGCTTTTATGGGTTATGTATTACCTTGGGGTCAAATGAGTTTCTGGGGTGCAACCGTTATAACTAATTTATTATATTTTATACCTGGACTTGTTTCATGGATTTGTGGTGGATATATAATTAGTGATCCAACTTTAAAAAGATTTTTTGTATTACATTTTATATTCCCATTTATAGCTTTATGTATTGTATTTATACATATATTCTTTTTACACTTACAAGGTAGCTCTAATCCTTTAGGATATGATACTGCTTTAAAAATACCTTTCTATCCAAGTCTATTATGTCTAGATATTAAAGGATTTAATAATGTATTAGTCTTATTTCTAGCACAAAGTTTATTTGGAATTTT | 478 | South_America |
| *Plasmodium* | CYNOV1 | KY783726 | Full | 1 | BELL02 | GCAACTGGTGCTTCATTTGTATTTATTTTAACTTATTTACATATTTTAAGAGGATTAAATTATTCATATTCATATTTACCTTTATCATGGATATCTGGATTAATAATATTCTTAATATCTATAGTAACAGCTTTTATGGGTTACGTATTACCTTGGGGTCAAATGAGTTTCTGGGGTGCTACCGTAATAACTAATTTATTATACTTTATACCTGGACTTGTTTCATGGATATGTGGTGGATATCTTGTAAGTGACCCAACTTTAAAAAGATTCTTTGTATTACATTTTACATTTCCTTTTATAGCTTTATGTATTGTATTTATACATATATTCTTTTTACATTTACAAGGTAGCACAAATCCTTTAGGGTATGATACAGCTTTAAAAATACCCTTCTATCCAAATCTTTTAAGTCTTGATATTAAAGGATTTAATAATGTATTAGTTTTATTTTTAGCACAAAGTTTATTTGGAATATT | 479 | Australia_._New_Zeeland |
| *Haemoproteus* | DENADE01 | AY455659 | Full | 1 | SERUT05 | GCTACCGGTGCTACATTTGTTTTTATTCTAACTTACTTACATATCTTAAGAGGATTAAACTATTCATATTCTTATTTACCTCTATCATGGATAACTGGATTAGTTATATTCTTAATTTCCATTGTTACCGCTTTTATGGGTTATGTATTACCTTGGGGTCAAATGAGTTTCTGGGGTGCAACCGTTATTACTAATTTATTATATTTTATACCTGGACTTGTTTCATGGATTTGTGGAGGATATACTATTAGTGATCCAACTTTAAAAAGATTCTTTGTATTACATTTTATATTCCCTTTTATAGCTTTATGTATTGTATTTATTCATATATTCTTCTTACACTTACAAGGTAGCTCTAATCCTTTAGGATATGATACAGCTTTAAAAATACCTTTCTATCCAAGTCTATTATGTCTAGATATCAAAGGATTTAATAATGTATTAGTCCTATTTCTAGCACAAAGTTTATTTGGTATTCT | 479 | Central_America |
| *Haemoproteus* | DENCOR03 | KF314771 | Full | 1 | TABI05 | GCTACTGGAGCTACATTTGTATTTATTCTTACTTACTTACATATTTTAAGAGGATTAAATTATTCATATTCATATTTACCTTTATCATGGATTACTGGATTAGTAATATTTTTAATTTCTATTGTTACTGCTTTTATGGGTTATGTTTTACCTTGGGGTCAAATGAGTTTCTGGGGTGCAACCGTTATTACTAATTTATTATATTTTATACCTGGACTTGTTTCATGGATTTGTGGTGGATATACTATAAGTGATCCAACTCTAAAAAGATTTTTTGTATTACATTTTATATTTCCTTTTATAGCTTTATGCATCGTATTTATACATATATTCTTCTTACATTTACAAGGTAGCTCTAATCCTTTAGGATATGATACAGCTTTAAAAATACCTTTCTATCCAAGTCTATTATGTTTAGATATTAAAGGATTTAATAATGTATTAGTTATATTTTTAGCACAAAGTTTATTTGGTATTCT | 479 | North_America |
| *Plasmodium* | DENCOR09 | OR063124 | Full | 1 | CORCAU05 | AACAGGTGCTTCATTTGTATTTATTCTAACTTATTTACATATTTTAAGAGGATTAAATTATTCATATTCATATTTACCTTTATCATGGATATCTGGATTATTTATATTCTTAATATCTATAGTTACAGCTTTTATGGGTTATGTATTACCTTGGGGTCAAATGAGTTTCTGGGGTGCAACTGTAATTACTAATCTATTATATTTTATACCTGGACTTGTTTCATGGATTTGTGGTGGATATCTTGTAAGTGACCCAACATTAAAAAGATTCTTTGTATTACATTTTACATTTCCATTTATAGCTTTATGTATTGTATTTATACATATATTCTTTCTACATTTACAAGGTAGCACTAATCCTTTAGGGTATGATACAGCTTTAAAAATACCCTTCTATCCAAATCTTTTAAGTCTCGATATTAAAGGATTTAATAATGTATTAGTATTATTTTTAGCACAAAGTTTATTTGGAATATT | 477 | - |
| *Haemoproteus* | DENPEN02 | AF465580 | Full | 1 | DENCOR01 | GCTACCGGTGCTACATTTGTTTTTATTTTAACTTACTTACATATATTAAGAGGATTAAACTACTCATATTCTTACTTACCTTTATCATGGATAACTGGATTAGTAATATTCTTAATCTCTATTGTTACCGCTTTTATGGGTTATGTATTACCTTGGGGTCAAATGAGTTTCTGGGGTGCAACCGTTATTACTAATTTATTATATTTTATACCTGGACTTGTTTCATGGATTTGTGGAGGATATACTATTAGTGATCCAACTTTAAAAAGATTTTTTGTATTACATTTTATATTTCCTTTTATAGCTTTATGTATTGTATTCATACATATATTCTTCTTACACTTACAAGGTAGCTCTAATCCTTTAGGATATGATACAGCTTTAAAAATACCTTTCTATCCAANTCTATTATGTCTAGATATCAAAGGATTTAATAATGTATTAGTCCTATTTCTAGCACAAAGTTTATTTGGAATTCT | 479 | North_America |
| *Plasmodium* | DENVID02 | KU057965 | Full | 1 | EMGOD01 | GCAACAGGTGCTTCATTTGTATTTATTTTAACTTACTTACATATTTTAAGAGGATTAAATTATTCATATTCATATTTACCATTATCATGGATTTCAGGATTAATAATATTCTTAATATCTATAGTAACTGCTTTTATGGGTTATGTATTACCTTGGGGTCAAATGAGTTTCTGGGGTGCTACAGTTATTACTAATTTATTATATTTTATTCCAGGACTTGTATCATGGATTTGTGGTGGATATCTTGTTAGTGACCCAACATTAAAAAGATTCTTTGTATTACACTTTACATTCCCATTTATAGCTTTATGTATTGTATTTATACATATATTCTTTTTACATTTACAAGGTAGCACTAATCCTTTAGGGTATGATACAGCTTTAAAAATACCCTTCTATCCAAATCTATTAAGCCTTGATATTAAAGGATTTAATAATGTATTAGTTTTATTTTTATCTCAAAGTTTATTTGGAATTTT | 479 | South_America, Asia |
| *Haemoproteus* | DICADS01 | MW546950 | Full | 1 | DICLEU01 | TCTTTATTTTAACTTATTTACATATATTAAGAGGATTAAATTATTCATATTCATATTTACCTTTATCATGGACAACTGGAATATTAATTTTCTTAATTTCTATTGTCACTGCTTTTATGGGTTATGTATTACCTTGGGGTCAAATGAGTTTCTGGGGTGCAACCGTTATAACTAATTTATTATATTTTATTCCTGGACTTGTTTCATGGATTTGTGGTGGATATATAATTAGTGATCCAACTTTAAAAAGATTTTTTGTATTACATTTTATATTTCCATTTATAGCTTTATGTATTGTATTTATACATATATTCTTTTTACACTTACAAGGTAGCTCTAATCCTTTAGGATATGATACTGCTTTAAAAATACCTTTCTATCCAAGTCTATTATGTCTAGATATAAAAGGATTTAATAATGTATTAGTCTTATTTCTAGCACAAAGTTTATTTGGAATTTT | 460 | - |
| *Haemoproteus* | DIGCAE01 | KM211348 | Full | 1 | DIGCYA01 | GCTACCGGTGCTACATTTGTTTTTATTCTAACTTACTTACATATCTTAAGAGGATTAAATTATTCATATTCTTATTTACCTTTATCATGGATAACTGGATTAGTTATATTCTTAATTTCAATTGTTACCGCTTTTATGGGTTATGTATTACCTTGGGGTCAAATGAGTTTCTGGGGTGCAACCGTTATAACTAATTTATTATATTTTATTCCTGGACTTGTTTCATGGATTTGTGGAGGATATACTATTAGTGATCCAACTTTAAAAAGATTTTTTGTATTACATTTTATATTTCCTTTTATAGCTTTATGTATTGTATTTATACATATATTCTTCTTACACTTACAAGGTAGCTCTAATCCTTTAGGATATGATACAGCTTTAAAAATACCTTTCTATCCAAGTCTATTATGTCTAGATATCAAAGGATTTAATAATGTATTAGTCCTATTTCTAGCACAAAGTTTATTTGGAATTCT | 479 | South_America |
| *Haemoproteus* | DIGHUM01 | JQ988117 | Full | 1 | MELGEO01 | CTACCGGTGCTACATTTGTTTTTATTCTAACTTACTTACATATATTAAGAGGATTAAACTATTCATATTCATATTTACCTTTATCATGGATAACTGGATTATTTATATTCTTAATTTCAATTGTTACCGCTTTTATGGGTTATGTATTACCTTGGGGTCAAATGAGTTTCTGGGGTGCAACCGTTATTACTAATTTATTATATTTTATTCCTGGACTTGTTTCATGGATTTGTGGAGGATATACTATTAGTGATCCAACTTTAAAAAGATTTTTTGTATTACATTTTATATTTCCTTTTATAGCTTTATGTATTGTATTCATACATATATTCTTCTTACACTTACAAGGTAGCTCTAATCCTTTAGGATATGATACAGCTTTAAAAATACCTTTCTATCCAAGTCTATTATGTCTAGATATCAAAGGATTTAATAATGTATTAGTCCTATTTCTAGCACAAAGTTTATTTGGAATTCT | 478 | South_America |
| *Leucocytozoon* | DIUDIU11 | MK947686 | Full | 1 | APSPI05 | AACTGGTGCATCTTTTGTATTTATCTTAACATATCTACATATTTTAAGAGGATTAAACTATTCATTCTCTTACTTACCTTTATCATGGTATAGTGGTTTAATTATATTCTTAATCTTTATTGTAACTGCTTTTATGGGTTACGTTTTACCATGGGGACAAATGAGTTTCTGGGGAGCAACTGTAATTACTAATTTATTATATTTTATTCCTGGATTAATTAATTGGGTCTGTGGTGGATTCATAATTAATGACCCAACATTAAAAAGATTCTTCGTATTACACTTTATATTCCCATTTATAGCCTTAGCTATTGTATTTATTCATATATTCTTCTTACATATTCATGGTAGCACTAATCCTTTAGGGTATGATACACCTTTAAAAATACCATTCTATCCAAATCTATTAACTTTAGATATTAAAGGATTTAACTATGTATTAGTTATATTTTTATTTCAAAGTTTATTTGGAATTGC | 477 | South_America |
| *Haemoproteus* | DUMCAR08 | MW081131 | Full | 1 | DUMCAR10 | GCTACTGGAGCTACATTTGTATTTATTCTAACTTATTTACATATCTTAAGAGGATTAAATTACTCATATTCATATTTACCACTATCATGGATAACAGGATTGGTAATATTCTTAATTTCTATTGTTACTGCTTTTATGGGTTATGTATTACCTTGGGGTCAAATGAGTTTCTGGGGTGCAACCGTTATTACTAATTTATTATATTTTATACCTGGACTTGTTTCATGGATTTGTGGAGGATATACTATAAGTGATCCAACTTTAAAAAGATTTTTTGTATTACACTTTATATTTCCTTTTATAGCCTTATGTATTGTATTTATACATATATTTTTCTTACACTTACAAGGTAGCTCTAATCCTTTAGGATATGATACAGCTTTAAAAATACCTTTCTATCCAAGTCTATTATGTCTAGATATAAAAGGATTTAATAATGTATTAGTTATATTTTTAGCTCAAAGTTTATTTGGTATTC | 478 | North_America |
| *Haemoproteus* | DUNNO01 | DQ991080 | Full | 1 | MELGEO01 | GCTACCGGTGCTACATTTGTTTTTATTTTAACTTACTTACATATATTAAGAGGATTAAACTACTCATATTCTTATTTACCTTTATCATGGATAACTGGATTAGTAATATTCTTAATCTCTATTGTTACCGCTTTTATGGGTTATGTATTACCTTGGGGTCAAATGAGTTTCTGGGGTGCAACCGTTATTACTAATTTATTATATTTTATACCTGGACTTGTTTCATGGATTTGTGGAGGATATACTATTAGTGATCCAACTTTAAAAAGATTTTTTGTATTACATTTTATATTTCCTTTTATAGCTTTATGTATTGTATTCATACATATATTCTTCTTACACTTACAAGGTAGCTCTAATCCTTTAGGATATGATACAGCTTTAAAAATACCTTTCTATCCAAGTCTATTATGTCTAGATATCAAAGGATTTAATAATGTATTAGTCCTATTTCTAGCACAAAGTTTATTTGGAATTCT | 479 | Europe, North_America |
| *Plasmodium* | ELAALB03 | MK695480 | Full | 1 | NEOBS02 | AACAGGTGCTTCATTTGTATTTATTTTAACTTATTTACATATTTTAAGAGGATTAAATTATTCATATTCATATTTACCTTTATCATGGATATCAGGATTAATAATATTTTTAATATCTATAGTAACAGCCTTTATGGGTTATGTACTACCATGGGGTCAAATGAGTTTTTGGGGTGCAACTGTAATCACTAATTTATTATACTTTATACCTGGACTCGTTTCATGGATATGTGGAGGATATCTTGTAAGTGACCCAACTTTAAAAAGATTCTTTGTATTACATTTTATATTCCCATTTATAGCTTTATGTATTGTATTTATACATATATTCTTTTTACATCTACAAGGTAGCACAAATCCTTTAGGGTATGATACAGCTTTAAAAATACCCTTCTATCCAAATCTTTTAAGTCTTGATATAAAAGGATTTAATAATATATTAGTATTATTCTTATCACAAAGTTTATTTGGAATATT | 477 | South_America |
| *Leucocytozoon* | ELAALB05 | MK947689 | Full | 1 | APSPI04 | AACTGGTGCATCTTTTGTATTTATCTTAACATATCTACATATTTTAAGAGGATTAAACTATTCATTCTCTTACTTACCTTTATCATGGTATAGTGGTTTAGTTATATTCTTAATCTTTATTGTAACTGCTTTTATGGGTTACGTTTTACCATGGGGACAAATGAGTTTCTGGGGAGCAACTGTAATTACTAATTTATTATATTTTATTCCTGGATTAATTAATTGGGTCTGTGGTGGATTCATTATTAATGACCCAACACTAAAAAGATTCTTCGTATTACACTTTATATTCCCATTTGTAGCCTTAGCTATTGTATTTATTCATATATTCTTCTTACATATTCATGGTAGCACTAATCCTTTAGGGTATGATACACCTTTAAAAATACCATTCTATCCAAATCTATTAACTTTAGATATTAAAGGATTTAACTATGTATTAGTTATATTTTTATTTCAAAGTTTATTTGGAATTGC | 477 | South_America |
| *Leucocytozoon* | EMGOD06 |  | Full | 1 | PARUS64 | TCAACAGGTGCATCTTTTGTATTTATATTAACATATCTACATATCTTAAGAGGATTAAATTATTCTTTCTCTTACTTACCTTTATCATGGTATAGTGGTTTAATTATATTCTTAATACTTATTGTAACTGCTTTTATGGGTTACGTTTTACCATGGGGACAAATGAGTTTCTGGGGAGCAACTGTAATTACTAACTTATTATATTTTATTCCTGGATTAATTAATTGGGTCTGTGGTGGATTTATTATTAATGACCCTACACTAAAAAGATTCTTCGTATTACATTTTATATTCCCATTTGTAGCCTTAGCTATTGTATTTATTCATATATTCTTCTTACATATTCATGGTAGCAATAATCCTTTAGGGTATGATACACCTTTAAAAATACCATTCTATCCAAATCTATTAACTTTAGATGTTAAAGGATTTAACTATGTACTAGTTATATTTTTATTTCAAAGTTTATTTGGAATTGC | 479 | Asia |
| *Haemoproteus* | EUPXAN02 | MN459077 | Full | 1 | THAFAN01 | CTACTGGAGCCACATTTGTATTTATTCTAACTTACTTACATATCTTAAGAGGATTAAATTATTCATATTCATATTTACCTATATCATGGATAACAGGATTGGTAATATTCTTAATTTCTATTGTTACTGCTTTTATGGGTTATGTATTACCATGGGGTCAAATGAGTTTCTGGGGTGCAACCGTTATTACTAATTTATTGTACTTTATACCTGGTCTTGTATCATGGATTTGTGGTGGATATACTATAAGCGATCCAACTTTAAAAAGATTTTTTGTACTACATTTTATATTTCCTTTTATAGCCTTATGTATTGTATTTATACATATATTCTTCTTACATTTACAAGGTAGCTCTAATCCTTTAGGATATGATACAGCTTTAAAAATACCTTTCTATCCAAGTCTATTATGTCTAGATATTAAAGGATTTAATAATGTATTAGTTATATTTTTAGCACAAAGTTTATTTGGAATATT | 478 | South_America |
| *Plasmodium* | FALTIN05 | MT281479 | Full | 1 | ZOSBRU01 | GCAACAGGTGCATCATTTGTATTTATTCTTACCTATTTACATATTTTAAGAGGATTAAATTACTCATACTCATATTTACCTTTATCATGGATATCTGGATTAATAATATTTTTAATATCAATAGTAACAGCTTTTATGGGATATGTATTACCTTGGGGTCAAATGAGCTTTTGGGGTGCAACTGTTATAACAAATTTACTATACTTTATTCCTGGTCTTGTTTCATGGATTTGTGGTGGATATCTTGTAAGTGACCCAACATTAAAAAGATTTTTTGTATTACATTTTATATTCCCATTTATAGCCTTATGTATTGTATTTATACATATTTTCTTTTTACATTTACAAGGTAGCACAAATCCTTTAGGGTATGATACAGCTTTAAAAATACCCTTCTATCCAAATCTATTAAGTCTTGATATTAAAGGATTTAATAATGTATTAGTTTTATTTTTATCACAAAGCTTATTTGGAATATT | 479 | Asia |
| *Haemoproteus* | FANTAIL03 | AY714156 | Full | 1 | NECASP01 | GCTACTGGTGCTACATTTGTCTTTATTCTAACTTATTTACATATATTAAGAGGATTAAATTATTCATATTCATATTTACCATTATCATGGACAACTGGAATAATCATTTTCTTAATTTCTATTGTTACTGCTTTTATGGGTTATGTATTACCTTGGGGTCAAATGAGTTTCTGGGGTGCAACCGTTATAACTAATTTATTATATTTTATACCTGGACTTGTTTCATGGATTTGTGGTGGATATATAATTAGTGATCCAACTTTAAAAAGATTTTTTGTATTACATTTTATATTCCCATTTATAGCTTTATGTATTGTATTTATACATATATTCTTTTTACACTTACAAGGTAGCTCTAATCCTTTAGGATATGATACTGCTTTAAAAATACCTTTCTATCCAAGTCTATTATGTCTAGATATTAAAGGATTTAATAATGTATTAGTCTTATTTCTAGCACAAAGTTTATTTGGAATTTT | 479 | Oceania |
| *Leucocytozoon* | FICZAN14 |  | Full | 1 | PARVEN01 | TCAACAGGTGCATCTTTTGTATTTATATTAACATATCTACATATCTTAAGAGGATTAAATTATTCTTTCTCTTATTTACCTTTATCATGGTATAGTGGTTTAATTATATTCTTAATACTTATTGTAACTGCTTTTATGGGTTACGTTTTACCATGGGGACAAATGAGTTTCTGGGGAGCAACTGTAATTACTAACTTATTATATTTTATTCCTGGATTAATTAATTGGGTCTGTGGTGGATTTATTATTAATGACCCTACACTAAAAAGATTCTTCGTATTACATTTTATATTCCCATTTGTAGCCTTAGCTATTGTATTTATTCATATATTCTTCTTACATATTCATGGTAGCAATAATCCTTTAGGGTATGATACACCTTTAAAAATACCATTCTATCCAAATCTATTAATTTTAGATGTTAAAGGATTTAACTATGTACTAGTTATATTTTTATTTCAAAGTTTATTTGGAATTGC | 479 | Asia |
| *Plasmodium* | FICZAN23 |  | Full | 1 | GRW02 | GCAACAGGAGCTTCATTTGTATTTATTTTAACTTATCTACATATTTTAAGAGGATTAAATTATTCTTATTCATATCTACCTTTATCATGGATTTCAGGATTAATTATATTTTTAATATCTATAGTTACTGCTTTTATGGGATATGTATTACCTTGGGGTCAAATGAGTTTTTGGGGAGCAACCGTAATTACTAACTTATTATATTTTATTCCAGGACTTGTTTCATGGATCTGTGGTGGATATTTAGTTAGTGACCCAACATTAAAAAGATTTTTCGTATTACATTTTACATTTCCATTTATAGCTTTATGTATTGTATTTATACATATATTCTTTTTACATCTACAAGGTAGCACAAATCCTTTAGGGTATGATACAGCTTTAAAAATACCCTTCTATCCAAATCTATTAAGTCTCGATATTAAAGGATTTAATAATGTCTTAGTATTATTTTTAGCACAAAGTTTATTTGGAATCTT | 479 | Asia |
| *Haemoproteus* | FIPAR02 | EF380197 | Full | 1 | AEGCAU03 | GCCACAGGTGCTACATTTGTATTTATTTTAACTTACTTACATATTTTAAGAGGATTAAATTATTCATACTCTTACTTACCTTTATCATGGATAACTGGATTATTAATATTCTTAATTTCTATTGTTACTGCTTTTATGGGTTATGTATTACCTTGGGGTCAAATGAGTTTCTGGGGTGCAACTGTTATCACTAATTTATTATATTTTATACCTGGACTAGTTTCATGGATTTGTGGAGGATATATTATAAGTGATCCAACTTTAAAAAGATTTTTTGTATTACATTTTATATTCCCTTTTATAGCCCTATGTATTGTGTTTATACATATATTTTTCTTACATTTACAAGGTAGCTCTAATCCTTTAGGATATGATACAGCTTTAAAAATACCTTTCTATCCAAGTCTATTATGTCTAGATATCAAAGGATTTAATAATGTATTAGTCCTATTTCTAGCACAAAGTTTATTTGGAATACT | 479 | Europe, Asia |
| *Haemoproteus* | FISEM01 | MG976560 | Full | 1 | PSADEC01 | GCTACAGGTGCTACATTTGTGTTTATTTTAACTTATTTACATATCTTAAGAGGATTAAACTATTCATATTCATATCTACCATTATCATGGATAACTGGATTAATAATATTCTTAATTTCCATTGTTACCGCTTTTATGGGTTATGTATTACCTTGGGGTCAAATGAGTTTCTGGGGTGCAACCGTTATTACTAATTTATTATATTTTATACCTGGACTTGTTTCATGGATTTGTGGAGGATATACTATTAGTGATCCAACTTTAAAAAGATTCTTTGTATTACATTTTATATTTCCTTTTATAGCTTTATGTATTGTATTTATACATATATTCTTCTTACACTTACAAGGTAGCTCTAATCCTTTAGGATATGATACAGCTTTAAAAATACCTTTCTATCCAAGTCTATTATGTCTAGATATCAAAGGATTTAATAATGTATTAGTCCTATTTCTAGCACAAAGTTTATTTGGAATTCT | 479 | Asia |
| *Haemoproteus* | FIZAN01 | KJ145100 | Partial | 1 | AECA01 | TGGGGTCAAATGAGTTTCTGGGGTGCAACCGTTATTACTAATTTACTATATTTTATACCTGGACTTGTTTCATGGATTTGTGGAGGATATACTATTAGTGATCCAACTTTAAAAAGATTTTTTGTATTACATTTTATATTTCCATTTATAGCCCTATGTATTGTATTTATACATATATTCTTCTTACACTTACAAGGTAGCTCTAATCCTTTAGGATATGATACAGCTTTAAAAATACCTTTCTATCCAAGTCTATTATGTCTAGATATTAAAGGATTTAATAATGTATTAGTTTTATTTTTAGCACAAAGTTTATTTGGAATACT | 326 | Asia |
| *Plasmodium* | GALLUS47 | OP763609 | Full | 1 | ZOSBRU01 | GCAACAGGCGCATCATTTGTATTTATTCTTACTTATTTACATATTTTAAGAGGATTAAATTACTCATACTCATATTTACCTTTATCATGGATATCTGGATTAATAATATTTTTAATATCAATAGTAACAGCTTTTATGGGATATGTATTACCTTGGGGTCAAATGAGCTTTTGGGGTGCAACTGTTATAACAAATTTACTATACTTTATTCCTGGTCTTGTTTCATGGATTTGTGGTGGATATCTTGTAAGTGACCCAACATTAAAAAGATTTTTTGTATTACATTTTATATTCCCATTTATAGCCTTATGTATTGTATTTATACATATTTTCTTTTTACATTTACAAGGTAGCACAAATCCTTTAGGGTATGATACAGCTTTAAAAATACCCTTCTATCCAAATCTATTAAGTCTTGATATTAAAGGATTTAATAATGTATTAGTTTTATTTTTATCACAAAGCTTATTTGGAATATT | 479 | Asia |
| *Plasmodium* | GBCAM1 | DQ847267 | Full | 1 | RBQ16 | GCAACAGGTGCTTCATTTGTATTTATCTTAACTTATTTACATATTTTAAGAGGATTAAATTATTCATATTCATATTTACCTTTATCATGGATATCTGGACTAGTCATATTTTTAATATCTATTGTAACAGCTTTTATGGGTTATGTATTACCTTGGGGTCAAATGAGTTTCTGGGGTGCTACAGTTATAACTAATTTATTATATTTTATACCTGGACTTGTTTCATGGATATGTGGTGGATATCTTGTAAGTGACCCAACCTTAAAAAGATTCTTTGTATTACATTTTACATTTCCATTTATAGCTTTATGTATTGTATTTATACATATATTCTTTTTACATTTACAAGGTAGCACAAATCCTTTAGGGTATGATACAGCTTTAAAAATACCCTTCTATCCAAATCTTTTAAGTCTTGATATTAAAGGATTTAATAATGTATTAGTATTATTTTTAGCACAAAGTTTATTTGGAATATT | 479 | Europe, South_Sahara |
| *Plasmodium* | GEOPOL01 | OR063213 | Full | 1 | GEOTRI12 | AACTGGTGCATCATTTGTATTTATTCTTACATATCTACATATTTTAAGAGGATTAAATTATTCTTATTCTTATTTACCTTTATCATGGATATCAGGATTAATAATATTTTTAATATCAATAGTTACTGCTTTTATGGGATATGTATTACCTTGGGGTCAAATGAGTTTCTGGGGTGCAACTGTCATTACTAATTTATTATATTTTATACCTGGTCTTGTTTCATGGATCTGTGGTGGATATCTTGTAAGCGACCCAACATTAAAAAGATTTTTTGTATTACATTTTATATTTCCATTCATAGCTTTATGTATTGTATTTATACATATATTCTTTCTACATTTACAAGGTAGCACAAATCCTTTAGGGTATGATACAGCTTTAAAAATACCCTTCTATCCAAATCTATTAAGTCTTGATATTAAAGGATTTAATAATATCTTAGTTTTATTTTTAGCACAAAGTTTATTTGGAATATT | 477 | Central_America |
| *Plasmodium* | GEOTRI01 | EF011170 | Full | 1 | TABI07 | GCAACAGGTGCATCATTTGTATTTATTCTTACATATTTACATATTTTAAGAGGATTAAATTATTCCTATTCTTATTTACCTTTATCATGGATATCAGGATTAATAATATTTTTAATATCAATAGTTACTGCTTTTATGGGATATGTACTACCTTGGGGTCAAATGAGTTTCTGGGGTGCAACCGTCATTACTAATTTATTATATTTTATACCTGGTCTTGTTTCATGGATCTGTGGTGGATATCTTGTAAGCGACCCAACATTAAAAAGATTTTTTGTATTACATTTTATATTTCCATTTATAGCCTTATGTATTGTATTTATACATATATTCTTTCTACATTTACAAGGTAGCACAAATCCTTTAGGGTATGATACAGCTTTAAAAATACCCTTCTATCCAAATCTATTAAGTCTTGATATTAAAGGATTTAATAATATCTTAGTTTTATTTTTAGCACAAAGCTTATTTGGAATATT | 479 | North_America, South_America |
| *Plasmodium* | GEOTRI05 | EU328166 | Partial | 1 | SEIAUR02 | GGTGCAACCGTCATTACTAATTTATTATATTTTATACCTGGTCTTGTTTCATGGATCTGTGGTGGATATCTTGTAAGCGACCCAACATTAAAAAGATTTTTTGTATTACATTTTATATTTCCATTTATAGCCTTATGTATTGTATTTATACATATATTCTTTCTACATTTACAAGGTAGCACAAATCCTTTAGGGTATGACACAGCTTTAAAAATACCCTTCTATCCAAATCTATTAAGTCTTGATATTAAAGGATTTAATAATATCTTAGTTTTATTTTTAGCACAAAGCTTATTTGGAATATT | 305 | - |
| *Plasmodium* | GEOTRI06 | EU328170 | Full | 1 | LARINC02 | GCAACAGGTGCTTCATTTGTTTTCATTTTAACCTATTTACATATTTTAAGAGGATTAAATTACTCATATTCATATTTACCTTTATCATGGATTTCAGGATTATTAATATTTTTAATATCCATAGTTACTGCTTTTATGGGTTATGTATTACCTTGGGGTCAAATGAGTTTCTGGGGTGCTACAGTTATAACTAACTTATTATATTTTATACCTGGACTTGTCTCATGGATTTGTGGTGGATATCTTGTAAGTGACCCAACCTTAAAAAGATTTTTTGTATTACATTTTACATTCCCATTTATAGCTTTATGTATTGTATTCATACATATATTCTTCTTACATTTACAAGGTAGCACAAATCCTTTAGGGTATGATACAGCTTTAAAGATACCCTTCTATCCAAATCTATTAAGTCTTGATATTAAAGGATTTAATAATGTATTAGTTTTATTCTTATCTCAAAGATTATTTGGAATTTT | 479 | - |
| *Plasmodium* | GEOTRI08 | EU328162 | Partial | 1 | VOLJAC01 | GGTGCTACAGTTATAACTAACTTATTATATTTTATACCTGGACTTGTCTCATGGATTTGTGGTGGATATCTTGTAAGTGACCCAACTTTAAAAAGATTTTTCGTATTACATTTTACATTTCCATTTATAGCTTTATGTATTGTATTTATACATATATTCTTCTTACATTTACAAGGTAGCACAAATCCTTTAGGGTATGATACAGCTTTAAAAATACCCTTCTATCCAAATCTATTAAGTCTTGATATTAAAGGATTTAATAATGTATTAGTTTTATTCTTATCTCAAAGTTTATTTGGAATTTT | 305 | - |
| *Plasmodium* | GEOTRI09 | EU328173 | Full | 1 | SEINOV01 | GCAACAGGCGCTTCATTTGTATTTATTTTAACTTACTTACATATTTTAAGAGGATTAAATTACTCATATTCATACTTACCTTTATCATGGATATCTGGTTTAATAATATTTTTAATATCAATAGTAACAGCTTTTATGGGTTATGTATTACCTTGGGGTCAAATGAGTTTCTGGGGTGCTACTGTAATTACTAATTTATTATATTTTATACCTGGACTTGTTTCATGGATTTGTGGTGGATATCTTGTTAGTGACCCAACATTAAAAAGATTCTTCGTATTACATTTTACATTTCCATTTATAGCTTTATGTATTGTATTTATACATATATTCTTCTTACATTTACAAGGTAGCACAAATCCTTTAGGGTATGATACAGCTTTAAAAATACCCTTCTATCCAAATCTTTTAAGTCTCGATATTAAAGGATTTAATAATGTATTAGTATTATTTTTATCACAAAGTTTATTTGGAATATT | 479 | North_America, Central_America, South_America |
| *Plasmodium* | GEOTRI11 | MF817789 | Full | 1 | TABI07 | GCAACAGGTGCATCATTTGTATTTATTCTTACATATTTACATATTTTAAGAGGATTAAATTATTCCTACTCTTATTTACCTTTATCATGGATATCAGGATTAATAATATTTTTAATATCAATAGTTACTGCTTTTATGGGATATGTACTACCTTGGGGTCAAATGAGTTTCTGGGGTGCAACCGTCATTACTAATTTATTATATTTTATACCTGGTCTTGTTTCATGGATCTGTGGTGGATATCTTGTAAGCGACCCAACATTAAAAAGATTTTTTGTATTACATTTTATATTTCCATTTATAGCCTTATGTATTGTATTTATACATATATTCTTTCTACATTTACAAGGTAGCACAAATCCTTTAGGGTATGATACAGCTTTAAAAATACCCTTCTATCCAAATCTATTAAGTCTTGATATTAAAGGATTTAATAATATCTTAGTTTTATTTTTAGCACAAAGCTTATTTGGAATATT | 479 | North_America |
| *Plasmodium* | GEOTRI12 | MW081139 | Full | 1 | GEOPOL01 | GCAACTGGTGCATCATTTGTATTTATTCTTACATATCTACATATTTTAAGAGGATTAAATTATTCTTATTCTTATTTACCTTTATCATGGATATCAGGATTAATAATATTTTTAATATCAATAGTTACTGCTTTTATGGGATATGTATTACCTTGGGGTCAAATGAGTTTCTGGGGTGCAACTGTCATTACTAATTTATTATATTTTATACCTGGTCTTGTTTCATGGATCTGTGGTGGATATCTTGTAAGCGACCCAACATTAAAAAGATTTTTTGTATTACATTTTATATTTCCATTCATAGCTTTATGTATTGTATTTATACATATATTCTTTCTACATTTACAAGGTAGCACAAATCCTTTAGGGTATGATACAGCTTTAAAAATACCCTTCTATCCAAATCTATTAAGTCTTGATATTAAAGGATTTAATAATATCTTAGTTTTATTTTTAGCACAAAGTTTATTTGGAATAT | 478 | North_America |
| *Haemoproteus* | GRMEL01 | MG976544 | Full | 1 | JUHYE01 | GCTACTGGTGCTACATTTGTTTTTATTTTAACTTACTTACATATATTAAGAGGATTAAACTATTCATATTCTTATTTACCTTTATCATGGATAACTGGATTAGTAATATTCTTAATTTCTATTGTTACCGCTTTTATGGGTTATGTATTACCTTGGGGTCAAATGAGTTTCTGGGGTGCAACCGTTATTACTAATTTATTATATTTTATACCTGGACTTGTTTCATGGATTTGTGGAGGATATACTATTAGTGATCCAACTTTAAAAAGATTTTTTGTATTACATTTTATATTTCCTTTTATAGCTTTATGTATTGTATTTATACATATATTCTTCTTACACTTACAAGGTAGCTCTAATCCTTTAGGATATGATACAGCTTTAAAAATACCTTTCTATCCAAGTCTATTATGTCTAGATATCAAAGGATTTAATAATGTATTAGTCCTATTTCTAGCACAAAGTTTATTTGGTATTCT | 479 | Asia |
| *Plasmodium* | GRW02 | AF254962 | Full | 1 | FICZAN23 | CAACAGGAGCTTCATTTGTATTTATTTTAACTTATCTACATATTTTAAGAGGATTAAATTATTCTTATTCATATCTACCTTTATCATGGATTTCAGGATTAATTATATTTTTAATATCTATAGTTACTGCTTTTATGGGATATGTATTACCTTGGGGTCAAATGAGTTTTTGGGGAGCAACCGTAATTACTAACTTATTATATTTTATTCCAGGACTTGTTTCATGGATCTGTGGTGGATATTTAGTTAGTGACCCAACATTAAAAAGATTTTTCGTATTACATTTTACATTTCCATTTATAGCTTTATGTATTGTATTTATACATATATTCTTTTTACATCTACAAGGTAGCACAAATCCTTTAGGGTATGATACAGCTTTAAAAATACCCTTCTATCCAAATCTATTAAGTCTCGATATTAAAGGATTTAATAATGTCTTAGTATTATTTTTAGCACAAAGTTTATTTGGAATCTT | 478 | Europe, South_Sahara, North_Africa_._Middle_East |
| *Haemoproteus* | GYMSAL01 | KU562209 | Full | 1 | ZONALB01 | TACTGGAGCTACATTTGTATTTATTCTAACTTACTTACATATTTTAAGAGGATTAAATTATTCATATTCATATTTACCTTTATCATGGATTACTGGATTGGTAATATTTTTAATTTCTATTGTTACTGCTTTTATGGGTTATGTTTTACCTTGGGGTCAAATGAGTTTCTGGGGTGCAACCGTTATTACTAATTTATTATATTTTATACCTGGACTTGTTTCATGGATATGTGGTGGTTATACTATTAGTGATCCAACTCTAAAAAGATTTTTTGTATTACATTTTATATTTCCTTTTATAGCTTTATGCATCGTATTTATACATATATTCTTCTTACATTTACAAGGTAGCTCTAATCCTTTAGGATATGATACAGCTTTAAAAATACCTTTCTATCCAAGTCTATTATGTTTAGATATTAAAGGATTTAATAATGTATTAGTTATATTTTTAGCACAAAGTTTATTTGGTATTTT | 477 | Central_America |
| *Haemoproteus* | GYMSAL02 | KU562212 | Full | 1 | ZONALB01 | TACTGGAGCTACATTTGTATTTATTCTAACTTACTTACATATTTTAAGAGGATTAAATTATTCATATTCATATTTACCTTTATCATGGATTACTGGATTGGTAATATTTTTAATTTCTATTGTTACTGCTTTTATGGGTTATGTTTTACCTTGGGGTCAAATGAGTTTCTGGGGTGCAACCGTTATTACTAATTTATTATATTTTATACCTGGACTTGTTTCATGGATATGTGGTGGTTATACTATTAGTGATCCAACTCTAAAAAGATTTTTTGTATTACATTTTATATTTCCTTTTATAGCTTTATGCATCGTATTTATACATATATTCTTCTTACATTTACAAGGTAGCTCTAATCCTTTAGGATATGATACAGCTTTAAAAATACCTTTCTATCCAAGTCTATTGTGTTTAGATATTAAAGGATTTAATAATGTATTAGTTATATTTTTAGCACAAAGTTTATTTGGTATTTT | 477 | South_America |
| *Haemoproteus* | HABFUS01 | OR063223 | Full | 1 | SERUT04 | TACCGGTGCTACATTTGTTTTTATTCTAACTTACTTACATATTTTAAGAGGATTAAACTATTCATATTCTTATTTACCTTTATCATGGATATCTGGATTAGTTATATTCTTAATTTCAATTGTTACCGCTTTTATGGGTTATGTATTACCTTGGGGTCAAATGAGTTTCTGGGGTGCAACCGTTATTACTAATTTATTATATTTTATACCTGGACTTGTTTCATGGATTTGTGGAGGATATACTATTAGTGATCCAACTTTAAAAAGATTCTTTGTATTACATTTTATATTTCCTTTTATAGCTTTATGTATTGTATTTATTCATATATTCTTCTTACACTTACAAGGTAGCTCTAATCCTTTAGGATATGATACAGCTTTAAAAATACCTTTCTATCCAAGTCTATTATGTCTAGATATCAAAGGATTTAATAATGTATTAGTCCTATTTCTAGCACAAAGTTTATTTGGTATTCT | 477 | Central_America |
| *Plasmodium* | HEDCOL01 | KJ446995 | Full | 1 | NEOLI01 | GCAACAGGTGCATCATTTGTATTTATTCTTACTTATTTACATATTTTAAGAGGATTAAACTATTCTTATTCATATTTACCTTTATCATGGATGTCAGGATTAATAATATTTTTAATATCAATTGTAACTGCTTTTATGGGATATGTATTACCTTGGGGTCAAATGAGTTTCTGGGGTGCAACTGTTATTACCAACTTACTCTACTTTATACCTGGTCTTGTTTCATGGATTTGTGGTGGATATCTTGTAAGTGATCCAACATTAAAAAGATTTTTTGTATTACATTTTATATTTCCATTTATAGCTTTATGTATTGTGTTTATACATATATTCTTTCTACATTTACAAGGTAGCACAAATCCTTTAGGATATGATACAGCTTTAAAAATACCCTTCTATCCAAATCTATTAAGTCTTGATATTAAAGGATTTAATAATATCTTAGTTTTATTTTTAGCACAAAGTTTATTTGGAAT | 476 | South_Sahara |
| *Haemoproteus* | HEMATR01 | KM211352 | Full | 1 | DIGCYA03 | GCTACCGGTGCTACATTTGTTTTTATTCTAACTTACTTACATATCTTAAGAGGATTAAATTATTCATATTCTTATTTACCTTTATCATGGATAACTGGGTTAGTTATATTCTTAATATCAATTGTTACCGCTTTTATGGGTTATGTATTACCTTGGGGTCAAATGAGTTTCTGGGGTGCAACCGTTATAACTAATTTATTATATTTTATTCCTGGACTTGTTTCATGGATTTGTGGAGGATATACTATTAGTGATCCAACTTTAAAAAGATTTTTTGTATTACATTTTATATTTCCTTTTATAGCTTTATGTATTGTATTTATACATATATTCTTCTTACACTTACAAGGTAGCTCTAATCCTTTAGGATATGATACAGCTTTAAAAATACCTTTCTATCCAAGTCTATTATGTCTAGATATCAAAGGATTTAATAATGTATTAGTCCTATTTCTAGCACAAAGTTTATTTGGAATTCT | 479 | South_America |
| *Leucocytozoon* | HEMATR03 | MN459129 | Partial | 1 | DIGCYA05 | CAACAGGTGCATCTGTTGTCTTTATATTAACATATTTACATATTCTAAGAGGTTTAAATTACTCTTTCTCTTACTTACCTTTATCATGGACAAGTGGTTTAATAATATTCTTAATATTTATTGTTACTGCGTTTATGGGTTATGTCTTACCATGGGGTCAAATGAGTTTCTGGGGAGCTACTGTAATTACAAATTTATTATATTTTATTCCTGGATTAATAAATTGGGTTTGTGGTGGTTTTATAATTAACGACCCAACTCTAAAAAGATTCTTTGTATTACATTTTATATTCCCATTCGTAGCACTAGCAATGGTATTTATTCATATATTCTTCTTACATATTCAAGGTAGCACTAATCCTTTAGGGTATGATACACCTTTAAAAATACCATTCTATCCAAATTTATTAACTCTAGATGTTAAAGGATTTAATTATGTATTAGTA | 446 | South_America |
| *Plasmodium* | HYLLEU02 | OR063230 | Full | 1 | SEIAUR02 | AACAGGTGCATCATTTGTATTTATTCTTACATATTTACATATTTTAAGAGGATTAAATTATTCTTATTCTTATTTACCTTTATCATGGATATCAGGATTAATAATATTTTTAATATCAATAGTTACTGCTTTTATGGGATATGTACTACCTTGGGGTCAAATGAGTTTCTGGGGTGCAACCGTCATTACTAATTTATTATATTTTATACCTGGTCTTGTTTCATGGATCTGTGGTGGATATCTTGTAAGCGACCCAACATTAAAAAGATTTTTTGTATTACATTTTATATTTCCATTTATAGCCTTATGTATTGTATTTATACATATATTCTTTCTACATTTACAAGGTAGCACAAATCCTTTAGGGTATGATACAGCTTTAAAAATACCCTTCTATCCAAACCTATTAAGTCTTGATTTTAAAGGATTTAATTCTATCTTAGTTTTATTTTTAGCACAAAGATTATTTGGAATATT | 477 | Central_America |
| *Leucocytozoon* | HYLMUS02 | MK947576 | Full | 1 | CATMIN07 | CACAGGTGCATCTTTTGTATTTATATTAACATATCTTCATATCTTAAGAGGATTAAACTATTCTTTCTCTTACTTACCTTTATCATGGTATAGTGGTTTAATAATATTCTTAATTTTTATTGTAACTGCTTTCATGGGTTACGTCTTACCATGGGGACAAATGAGTTTCTGGGGAGCAACTGTAATTACTAATTTATTATATTTTATTCCTGGATTAATTAATTGGGTATGTGGTGGATTTATTATTAATGACCCAACACTAAAAAGATTCTTCGTATTACACTTCATATTCCCATTTATAGCATTAGCTATTGTATTTATTCATATATTCTTCTTACATATTCATGGTAGCACTAATCCTTTAGGGTATGATACACCTTTAAAAATACCATTCTATCCAAATCTATTAACTTTAGATATTAAAGGATTCAACTATGTATTAGTTATATTCTTATTTCAAAGTTTATTTGGAATTGC | 477 | North_America, Central_America, South_America |
| *Leucocytozoon* | HYPAM03 | LC230142 | Full | 1 | HYPAM01 | TCAACAGGTGCATCTTTTGTATTTATCTTAACATATCTACATATCTTAAGAGGATTAAATTATTCTTTCTCTTACTTACCTTTATCATGGTATAGCGGTTTAATAATATTCTTAATCTTTATTGTAACTGCTTTTATGGGTTATGTTTTACCATGGGGACAAATGAGTTTCTGGGGAGCAACTGTAATTACTAACTTATTATATTTCATTCCTGGATTAATTAATTGGGTCTGTGGTGGATTTATTATTAATGACCCAACATTAAAAAGATTCTTTGTATTACATTTTATATTCCCATTTATAGCTTTAGCTATTGTATTTATTCATATATTCTTCTTACATATTCATGGTAGCACTAATCCTTTAGGGTATGATACACCTTTAAAAATACCATTCTATCCAAATCTATTAACTTTAGATGTAAAAGGATTTAACTATGTACTAGTTATATTTTTATTCCAAAGTTTATTTGGAATTGC | 479 | Asia |
| *Plasmodium* | ICTCHR03 | OR063249 | Full | 1 | SEIAUR02 | AACAGGTGCATCATTTGTATTTATTCTTACATATTTACATATTTTAAGAGGATTAAATTATTCTTATTCTTATTTACCTTTATCATGGATATCAGGATTAATAATATTTTTAATATCAATAGTTACTGCTTTTATGGGATATGTACTACCTTGGGGTCAAATGAGTTTCTGGGGTGCAACCGTCATTACTAATTTATTATATTTTATACCTGGTCTTGTTTCATGGATCTGTGGTGGATATCTTGTAAGCGACCCAACATTAAAAAGATTTTTTGTATTACATTTTATATTTCCATTTATAGCCTTATGTATTGTATTTATACATATATTCTTTCTACATTTACAAGGTAGCACAAATCCTTTAGGGTATGATACAGCTTTAAAAATACCCTTCTATCCAAATCTATTAAGTCTTGATATTAAAGGATTTAATAATATCTTAGTTTTATTTTTAGCACAAAGCTTATTTGCAATATT | 477 | Central_America |
| *Haemoproteus* | ICTGUL03 |  | Full | 1 | JUHYE01 | GCTACCGGTGCTACATTTGTTTTTATTCTAACTTACTTACATATCTTAAGAGGATTAAACTATTCATATTCTTATTTACCTTTATCATGGATAACTGGATTAGTAATATTCTTAATTTCAATTGTTACCGCTTTTATGGGTTATGTATTACCTTGGGGTCAAATGAGTTTCTGGGGTGCAACCGTTATTACTAATTTATTATATTTTATACCTGGACTTGTTTCATGGATTTGTGGAGGATATACTATTAGTGATCCAACTTTAAAAAGATTTTTTGTATTACATTTTATATTTCCTTTTATAGCTTTATGTATTGTATTTATACATATATTCTTCTTACACTTACAAGGTAGCTCTAATCCTTTAGGATATGATACAGCTTTAAAAATACCTTTCTATCCAAGTCTATTATGTCTAGATATCAAAGGATTTAATAATGTATTAGTCCTATTTCTAGCACAAAGTTTATTTGGTATTCT | 479 | - |
| *Plasmodium* | IOLIND04 | MK493378 | Full | 1 | PYCCAF01 | GCAACAGGTGCATCATTTGTATTCATTCTTACTTATTTACATATTTTAAGAGGATTAAATTATTCATACTCATACTTACCTCTATCCTGGATATCAGGATTATTAATATTTTTAATATCAATAGTAACTGCTTTTATGGGATATGTATTACCTTGGGGTCAAATGAGTTTTTGGGGTGCAACCGTTATTACTAATTTATTATATTTCATACCTGGTCTTGTTTCATGGATTTGCGGTGGATATCTTGTAAGCGATCCAACACTAAAAAGATTCTTCGTATTACATTTTATATTTCCATTTATAGCTTTGTGTATTGTGTTCATACATATATTCTTTCTACATTTACAAGGTAGCACAAATCCTTTAGGGTATGATACAGCTTTAAAAATACCCTTCTATCCAAATCTTTTAAGTCTCGATATCAAAGGATTTAATAATATCCTAGTTTTATTTTTAGCACAAAGTTTATTTGGAATATT | 479 | Asia |
| *Haemoproteus* | JUHYE03 | KF314764 | Full | 1 | JUHYE01 | GCTACTGGTGCTACATTTGTTTTTATTTTAACTTACTTACATATATTAAGAGGATTAAACTATTCATATTCTTATTTACCTTTATCATGGATAACTGGATTAGTAATATTCTTAATTTCTATTGTTACCGCTTTTATGGGTTATGTTTTACCTTGGGGTCAAATGAGTTTCTGGGGTGCAACCGTTATTACTAATTTATTATATTTTATACCTGGACTTGTTTCATGGATTTGTGGAGGATATACTATTAGTGATCCAACTTTAAAAAGATTTTTTGTATTACATTTTATATTTCCTTTTATAGCTTTATGTATTGTATTTATACATATATTCTTCTTACACTTACAAGGTAGCTCTAATCCTTTAGGATATGATACAGCTTTAAAAATACCTTTCTATCCAAGTCTATTATGTCTAGATATCAAAGGATTTAATAATGTATTAGTCCTATTTCTAGCACAAAGTTTATTTGGTATTCT | 479 | North_America |
| *Haemoproteus* | JUHYE13 | MT350653 | Full | 1 | ZONALB01 | GCTACTGGAGCTACATTTGTATTTATTCTAACTTACTTACATATTTTAAGAGGATTAAATTATTCATATTCATATTTACCTTTATCATGGATTACTGGATTGGTAATATTTTTAATTTCTATTGTTACTGCTTTTATGGGTTATGTTTTACCTTGGGGTCAAATGAGTTTCTGGGGTGCAACCGTTATTACTAATTTATTATATTTTATACCTGGACTTGTTTCATGGATATGTGGTGGTTATACTATTAGTGATCCAACTCTAAAAAGATTTTTTGTATTACATTTTATATTTCCTTTTATAGCTTTATGCATCGTATTTATACATATATTCTTCTTACATTTACAAGGTAGCTCTAATCCTTTAGGATATGATACAGCTTTAAAAATACCTTTCTATCCAAGTCTATTATGTTTAGATATTAAAGGATTTAATAATGTATTAGTTATATTTTTAGCGCAAAGTTTATTTGGTATCTT | 479 | - |
| *Leucocytozoon* | JUHYE21 | MT350682 | Full | 1 | CORCAU02 | CAACAGGTGCATCATTTGTATTTATATTAACATACTTACATATATTAAGAGGATTAAATTATTCATTTACTTACTTACCTTTATCATGGATAAGTGGTTTAATAATATTCTTAATATTTATTGTAACTGCTTTTATGGGTTATGTCTTACCATGGGGTCAAATGAGTTTTTGGGGAGCTACTGTTATAACTAATTTATTATATTTTATTCCTGGATTAATTAATTGGGTTTGCGGTGGATTTATTATTAATGACCCAACTCTAAAAAGATTCTTCGTATTACATTTTATATTCCCATTTGTAGCATTAGCTATCGTATTTATACATATATTCTTCTTACATATTCAAGGTAGCACTAATCCTTTAGGGTATGATACACCTTTAAAAATACCATTCTATCCAAATCTATTAACTTTAGATGTTAAAGGATTTAACTATGTATTAGTATTATTCCTATTTCAAAGTTTATTTGGAATTG | 477 | - |
| *Haemoproteus* | JUNPHA14 | MT350663 | Full | 1 | ZONALB01 | GCTACTGGAGCTACATTTGTATTTATTCTAACTTACTTACATATTTTAAGAGGATTAAATTATTCATATTCATATTTACCTTTATCATGGATTACTGGATTGGTAATATTTTTAATTTCTATTGTTACTGCTTTTATGGGTTATGTTTTACCTTGGGGTCAAATGAGTTTCTGGGGTGCAACCGTTATTACTAATTTATTATATTTTATACCTGGACTTGTTTCATGGATATGTGGTGGTTATACTATTAGTGATCCAACTCTAAAAAGATTTTTTGTATTACATTTTATATTTCCTTTTATAGCTTTATGCATCGTATTTATACATATATTCTTCTTACATTTACAAGGTAGCTCTAATCCTTTAGGATATGATACAGCTTTAAAAATACCTTTCTATCCAAGTCTATTATGTTTAGATATTAAAGGATTTAATAATGTATTAGCTATATTTTTAGCACAAAGTTTATTTGGTATCTT | 479 | Central_America |
| *Haemoproteus* | JUNPHA19 | MT350658 | Full | 1 | JUHYE01 | GCTATTGGTGCTACATTTGTTTTTATTTTAACTCACTTACATATATTAAGAGGATTAAACTATTCATATTCTTATTTACCTTTATCATGGATAACTGGATTAGTAATATTCTTAATTTCTATTGTTACCGCTTTTATGGGTTATGTTTTACCTTGGGGTCAAATGAGTTTCTGGGGTGCAACCGTTATTACTAATTTATTATATTTTATACCTGGACTTGTTTCATGGATTTGTGGAGGATATACTATTAGTGATCCAACTTTAAAAAGATTTTTTGTATTACATTTTATATTTCCTTTTATAGCTTTATGTATTGTATTTATACATATATTCTTCTTACACTTACAAGGTAGCTCTAATCCTTTAGGATATGATACAGCTTTAAAAATACCTTTCTATCCAAGTCTATTATGTCTAGATATCAAAGGATTTAATAATGTATTAGTCCTATTTCTAGCACAAAGTTTATTTGGTATTCT | 479 | Central_America |
| *Plasmodium* | KEWA01 | MW081130 | Full | 1 | DENPEN01 | GCAACAGGTGCATCATTTGTATTTATTCTTACTTATCTACATATTTTAAGAGGATTAAATTATTCTTATTCTTATTTACCTTTATCATGGATATCAGGATTAATAATATTCTTAATATCAATAGTAACTGCTTTTATGGGATATGTATTACCTTGGGGTCAAATGAGTTTCTGGGGTGCAACTGTCATTACTAATTTATTATATTTTATACCTGGTCTTGTTTCATGGATTTGTGGTGGATATCTTGTAAGCGACCCAACATTAAAAAGATTTTTTGTATTACATTTTATATTTCCATTTATAGCCTTATGTATTGTATTTATACATATATTCTTTCTACATTTACAAGGTAGCACAAATCCTTTAGGGTATGATACAGCTTTAAAAATACCCTTCTATCCAAATCTATTAAGTCTTGATATTAAAGGATTTAATAATATTTTAGTTTTATTTTTAGCACAAAGTTTATTTGGAATAT | 478 | North_America, Central_America |
| *Leucocytozoon* | LAMUT01 | AB183550 | Partial | 1 | AKGPL06 | TCTACTGGTGCATCATTTGTCTTTATATTAACATATCTACATATATTAAGAGGACTAAACTATTCTTACTCATATTTACCTCTATCATGGATATCAGGTTTAATTATATTCTTTATATCCATAATGACAGCCTTTATGGGTTATGTTTTACCATGGGGACAAATGAGTTATTGGGGAGCAACTGTAATTACCAATTTATTATATTTTATTCCTGGATTAATTTCATGGGTTTGTGGAGGATTCGTTGTTAATGATCCAACTATAAAAAGATTCTTTGTACTTCACTTTATTTTCCCATTTATAGC | 305 | Asia |
| *Haemoproteus* | LARCAC01 | AF465593 | Partial | 1 | CHRRID01 | TACATTTTATATTCCCATTTGTTGCTTTATGTATTGTATTTATACATATATTCTTTTTACACTTACAAGGTAGCTCTAATCCTTTAGGATATGATACAGCTTTAAAAATACCTTTCTATCCAAGTCTATTATGTTTAGATATTAAAGGATTTAGTAATGTATTAGTTTTATACTTAGCTCAAAGTTTATTTGGTATACT | 199 | Europe |
| *Plasmodium* | LBPIP1 | DQ847265 | Full | 1 | RBQ16 | GCAACAGGTGCTTCATTTGTATTTATATTAACTTATTTACATATTTTAAGAGGATTAAATTATTCATATTCATATTTACCTTTATCATGGATATCTGGACTAGTCATATTTTTAATATCTATTGTAACAGCTTTTATGGGTTATGTATTACCTTGGGGTCAAATGAGTTTCTGGGGTGCTACAGTTATAACTAATTTATTATATTTTATACCTGGACTTGTTTCATGGATATGTGGTGGATATCTTGTAAGTGACCCAACCTTAAAAAGATTCTTTGTATTACATTTTACATTTCCATTTATAGCTTTATGTATTGTATTTATACATATATTCTTTTTACATTTACAAGGTAGCACAAATCCTTTAGGGTATGATACAGCTTTAAAAATACCCTTCTATCCAAATCTTTTAAGTCTTGATATTAAAGGATTTAATAATGTATTAGTATTATTTTTAGCACAAAGTTTATTTGGAATATT | 479 | Europe |
| *Haemoproteus* | LEIPER02 | KM211351 | Full | 1 | VIGRI01 | GCTACTGGTGCTACATTTGTTTTTATTTTAACTTATTTACATATCTTAAGAGGATTAAATTATTCATATTCATATTTACCTTTATCATGGATAACTGGATTAATAATATTCTTAATTTCTATTGTTACAGCTTTTATGGGTTATGTATTACCTTGGGGTCAAATGAGTTTCTGGGGTGCAACCGTTATTACTAATTTATTATATTTTATACCTGGACTTGTTTCATGGATTTGTGGAGGATATACTATAAGTGATCCAACTTTAAAAAGATTCTTTGTATTACATTTTATATTCCCTTTTATAGCTTTATGTATTGTATTTATACATATATTCTTCTTACATTTACAAGGTAGCTCTAATCCTTTAGGATATGATACAGCTTTAAAAATACCTTTCTATCCAAGTCTATTATGTCTAGATATTAAAGGATTTAATAATGTATTAGTCCTATTTCTAGCACAAAGTTTATTTGGAATTCT | 479 | South_America |
| *Plasmodium* | LEPCOR05 | KU236434 | Full | 1 | DOLFRI01 | CAACAGGTGCTTCATTTGTATTTATTTTAACTTATTTACATATTTTAAGAGGATTAAATTATTCATATTCATATTTACCTTTATCATGGATATCTGGACTAATTATATTTTTAATATCTATTGTAACAGCTTTTATGGGTTATGTATTACCTTGGGGTCAAATGAGTTTCTGGGGTGCTACAGTTATAACTAATTTATTATATTTTATACCTGGACTTGTTTCATGGATATGTGGTGGATATCTTGTAAGTGACCCAACCTTAAAAAGATTCTTTGTATTACATTTTACATTTCCATTTATAGCCTTATGTATTGTATTTATACATATATTCTTTCTACATTTACAAGGTAGCACAAATCCTTTAGGGTATGATACAGCTTTAAAAATACCCTTCTATCCAAATCTTTTAAGTCTTGATATTAAAGGATTTAATAATGTATTAGTATTATTTTTAGCACAAAGTTTATTTGGAATATT | 478 | South_America |
| *Plasmodium* | LEPCOR10 | OP779145 | Full | 1 | RBQ16 | AACTGGTGCTTCATTTGTATTTATTTTAACTTATTTACATATTTTAAGAGGATTAAACTATTCATATTCATATTTACCTTTATCATGGATGTCTGGATTAATTATATTTTTAATATCTATTGTAACAGCTTTTATGGGTTATGTATTACCTTGGGGTCAAATGAGTTTCTGGGATGCTACAGTTATTACTAATTTATTATATTTTATACCTGGACTTGTTTCATGGATATGTGGTGGATATCTTGTAAGTGACCCAACCTTAAAAAGATTCTTTGTATTACATTTTACATTTCCATTTATAGCTTTATGTATTGTATTTATACATATATTCTTTTTACATTTACAAGGTAGCACAAATCCTTTAGGGTATGATACAGCTTTAAAAATACCCTTCTATCCAAATCTTTTAAGTCTTGATATTAAAGGATTTAATAATGTATTAGTATTATTTTTAGCACAAAGTTTATTTGGAATATT | 477 | South_America |
| *Haemoproteus* | LISP01 | MW081135 | Full | 1 | SERUT05 | GCTACCGGTGCTACATTTGTTTTTATTCTAACTTACTTACATATTTTAAGAGGATTAAACTATTCATATTCTTATTTACCTTTATCATGGATAACTGGATTAGTTATATTCTTAATTTCAATTGTTACCGCTTTTATGGGTTATGTATTACCTTGGGGTCAAATGAGTTTCTGGGGTGCAACCGTTATTACTAATTTATTATATTTTATACCTGGACTTGTTTCATGGATTTGTGGAGGATATACTATTAGTGATCCAACTTTAAAAAGATTCTTTGTATTACATTTTATATTCCCTTTTATAGCTTTATGTATTGTATTTATTCATATATTCTTCTTACACTTACAAGGTAGCTCTAATCCTTTAGGATATGATACAGCTTTAAAAATACCTTTCTATCCAAGTCTATTATGTCTAGATATCAAAGGATTTAATAATGTATTAGTCCTATTTCTAGCACAAAGTTTATTTGGTATTC | 478 | North_America |
| *Plasmodium* | MALCOR02 | MK374282 | Full | 1 | NECASP02 | GCTACAGGTGCTTCATTTGTATTTATATTAACTTATTTACATATATTAAGAGGATTAAATTATTCTTATTCATATTTACCTTTATCATGGATAACTGGATTATTAATATTTTTAATATCTATAGTTACAGCTTTTATGGGTTATGTATTACCATGGGGTCAAATGAGTTTTTGGGGTGCTACAGTCATAACTAATTTATTATATTTTATACCTGGACTTGTTTCATGGATTTGTGGTGGATATCTTGTAAGCGATCCAACTTTAAAAAGATTTTTTGTATTACATTTCACATTTCCTTTTATAGCTTTATGTATTGTATTTATACATATTTTCTTTCTACATTTACAAGGTAGCACAAATCCTTTAGGGTATGATACTGCTTTAAAAATACCCTTCTATCCAAATCTATTAAGTCTTGATATTAAAGGATTTAATAATATTTTAGTATTATTTTTAGCTCAAAGTTTATTTGGAATTTT | 479 | Australia_._New_Zeeland |
| *Leucocytozoon* | MELLIN02 | MK947846 | Full | 1 | SILUT01 | AACCGGTGCATCTTTTGTATTAATATTAACATATCTACATATACTAAGAGGTTTAAATTACTCTTTCTCTTACTTACCTTTATCATGGATAAGTGGTTTAGTAATATTCTTAATATTTATTGTAACTGCTTTTATGGGTTATGTCTTACCATGGGGTCAAATGAGTTTCTGGGGAGCTACTGTAATTACTAACTTATTATATTTTATTCCTGGATTAATTAATTGGGTTTGTGGTGGTTTTATTATTAACGATCCAACTCTAAAAAGATTCTTTGTATTACATTTTATATTCCCATTCGTAGCTTTAGCTATTGTATTTATTCATATATTCTTCTTACATATTCAAGGTAGCACTAATCCATTAGGGTATGATACACCTTTAAAAATACCATTCTATCCAAATCTATTAACTTTAGATGTTAAAGGATTTAATTATGTATTAGTAATATTCTTATTTCAAAGTTTATTTGGTATTGC | 477 | - |
| *Plasmodium* | MELMEL03 | KT193632 | Full | 1 | TABI08 | TTTTACTTATTTACATATTTTAAGAGGATTAAATTATTCATATTCATATTTACCTTTATCATGGATATCTGGATTACTTATATTTTTAATATCTATTGTAACAGCTTTTATGGGTTATGTATTACCTTGGGGTCAAATGAGTTTCTGGGGTGCTACAGTCATTACTAATTTATTATATTTTATACCTGGACTTGTTTCATGGATATGTGGTGGATATCTTGTAAGTGACCCAACCTTAAAAAGATTCTTTGTATTACATTTTACATTTCCATTTATAGCCTTATGTATTGTATTTATACATATATTCTTTTTACATTTACAAGGTAGCACAAATCCTTTAGGGTATGATACAGCTTTAAAAATACCCTTCTATCCAAATCTTTTAAGTCTTGATATTAAAGGATTTAATAATGTATTAGTATTATTCTTAGCACAAAGTTTATTTGGAATATT | 453 | North_America |
| *Plasmodium* | MELMEL04 | KT193633 | Full | 1 | TABI08 | TTTTACTTATTTACATATTTTAAGAGGATTAAATTATTCATATTCATATTTACCTTTATCATGGATATCTGGATTACTTATATTTTTAATATCTATTGTAACAGCTTTTATGGGTTATGTATTACCTTGGGGTCAAATGAGTTTCTGGGGTGCTACAGTTATTACTAATTTATTATATTTTATACCTGGACTTGTTTCATGGATATGTGGTGGATATCTTGTAAGTGACCCAACCTTAAAAAGATTCTTTGTATTACATTTTACATTTCCATTTATAGCCTTATGTATTGTATTTATACATATATTCTTTTTACATTTACAAGGTAGCACAAATCCTTTAGGGTATGATACAGCTTTAAAAATACCCTTCTATCCAAATCTTTTAAGTCTTGATATTAAAGGATTTAATAATGTATTAGTATTATTCTTAGCACAAAGTTTATTTGGAATATT | 453 | North_America |
| *Plasmodium* | MELMEL23 | OR063309 | Full | 1 | TABI08 | AACAGGTGCTTCATTTGTATTTATTTTAACTTATTTACATATTTTAAGAGGATTAAATTATTCATATTCATATTTACCTTTATCATGGATATCTGGATTACTTATATTTTTAATATCTATTGTAACAGCTTTTATGGGTTATGTATTACCTTGGGGTCAAATGAGTTTCTGGGGCGCTACAGTTATTACTAATTTATTATATTTTATACCTGGACTTGTTTCATGGATATGTGGTGGATATCTTGTAAGTGACCCAACCTTAAAAAGATTCTTTGTATTACATTTTACATTTCCATTTATAGCCTTATGTATTGTATTTATACATATATTCTTTTTACATTTACAAGGTAGCACAAATCCTTTAGGGTATGATACAGCTTTAAAAATACCCTTCTATCCAAATCTTTTAAGTCTTGATATTAAAGGATTTAATAATGTATTAGTATTATTCTTAGCACAAAGTTTATTTGGAATATT | 477 | - |
| *Haemoproteus* | MELSTR01 | HM222472 | Full | 1 | PIPUB01 | GCTACTGGTGCTACATTTGTTTTTATATTAACATATTTACATATTTTAAGAGGATTAAATTATTCATATTCATATTTACCTTTATCATGGATAACTGGATTAATGATTTACTTAATTTCTATTGTTACTGCTTTTATGGGTTATGTATTACCTTGGGGTCAAATGAGTTTCTGGGGTGCAACTGTTATTACTAACTTATTATATTTTATACCTGGACTTGTTTCATGGATTTGTGGTGGATATAATATTAGTGATCCTACATTAAAGAGATTCTTTGTATTACATTTTATATTTCCATTTATAGCTTTATGTATTGTATTTATACATATATTCTTCTTACACTTACAAGGTAGCTCTAATCCTTTAGGATATGATACAGCTTTAAAAATACCTTTCTATCCAAGTCTATTATGTTTAGATATTAAAGGATTTAGTAATGTATTAGTATTATATTTAGCTCAAAGTTTATTTGGAATACT | 479 | North_America |
| *Haemoproteus* | MICRO01 | GU296223 | Full | 1 | GEMON01 | GCCACAGGTGCATCATTTGTATTTATTTTAACATACCTACACATTTTAAGAGGATTAAATTACTCATATTCATATTTACCATTATCATGGATTACCGGATTAATAATATTTATTATCTCTATTATGACTGCTTTCTTAGGTTATGTTCTACCTTGGGGTCAAATGAGTTTCTGGGGTGCAACTGTTATTACTAATTTATTATATTTTATTCCAGGATTAGTCTCATGGATTTGTGGTGGATATATTGTTAGTGATCCTACACTAAAAAGATTCTTTGTATTACATTTTATATTTCCATTTATAGCTATATGTATAGTATTTATTCATATATTCTTTTTACATTTACAAGGTAGCTCAAATCCTTTAGGATATGATACAGCATTAAAAATACCATTTTATCCAAATTTACTATGTTTAGATATAAAAGGATTTAATAACGTATTAGTATTATTCTTAGCTCAAAGCTTATTTGGAATATT | 479 | South_America |
| *Plasmodium* | MILANS06 | JN164715 | Full | 1 | TURAF01 | GCAACAGGTGCTTCATTTGTTTTTATTTTAACTTACTTACATATTTTAAGAGGATTAAATTATTCATACTCATACTTACCTTTATCATGGATATCTGGATTAATAATATTCTTAATATCTATAGTTACAGCTTTTATGGGTTATGTATTACCTTGGGGTCAAATGAGTTTCTGGGGTGCTACTGTAATAACCAATTTACTTTATTTTATTCCTGGACTTGTCTCATGGATTTGTGGTGGATATCTTGTAAGTGACCCAACCTTAAAAAGATTCTTTGTATTACATTTTACATTTCCTTTTATAGCTTTATGTATTGTATTTATACATATCTTTTTCTTACATTTACAAGGTAGCACTAATCCTTTAGGGTATGATACAGCTTTAAAAATACCCTTCTATCCAAATCTTTTAAGTCTTGATATTAAAGGATTTAATAATGTATTAGTATTATTCTTAGCTCAAAGTTTATTTGGAATATT | 479 | Europe, South_Sahara, Asia |
| *Haemoproteus* | MIMPOL05 | ON455415 | Full | 1 | MIMPOL03 | GCTACTGGAGCTACATTTGTATTTATTTTAACTTATTTACATATATTAAGAGGATTAAATTATTCATATTCATATTTACCTTTATCATGGATATCTGGATTAATAATATTCTTAATTTCTATAGTTACTGCTTTTATGGGTTATGTATTACCTTGGGGTCAAATGAGTTTCTGGGGTGCAACCGTTATTACTAATTTATTATATTTTATACCTGGACTTGTTTCATGGATTTGTGGTGGATATATTATTAGTGATCCAACTTTAAAAAGATTCTTTGTATTACATTTTATATTCCCATTTATAGCTTTATGTATTGTATTTATACATATATTCTTTTTACACTTACAAGGTAGCTCTAATCCTTTAGGATATGATACTGCTTTAAAAATACCTTTCTATCCAAGTCTATTATGTCTAGATATTAAAGGATTTAATAATGTATTAGTCTTATTTCTAGCACAAAGTTTATTTGGAATAT | 478 | North_America |
| *Haemoproteus* | MIMPOL06 | ON455416 | Full | 1 | MIMPOL03 | GCAACTGGTGCTACATTTGTATTTATTTTAACTTATTTACATATATTAAGAGGATTAAATTATTCATATTCATATTTACCTTTATCATGGATATCTGGATTAATAATATTCTTAATTTCTATAGTTACTGCTTTTATGGGTTATGTATTACCTTGGGGTCAAATGAGTTTCTGGGGTGCAACCGTTATTACTAATTTATTATATTTTATACCTGGACTTGTTTCATGGATTTGTGGTGGATATATTATTAGTGATCCAACTTTAAAAAGATTCTTTGTATTACATTTTATATTCCCATTTATAGCTTTATGTATTGTATTTATACATATATTCTTTTTACACTTACAAGGTAGCTCTAATCCTTTAGGATATGATACTGCTTTAAAAATACCTTTCTATCCAAGTCTATTATGTCTAGATATTAAAGGATTTAATAATGTATTAGTCTTATTTCTAGCACAAAGTTTATTTGGAATAT | 478 | North_America |
| *Haemoproteus* | MONCAC01 | MK493379 | Full | 1 | CATUST02 | GCTACTGGTGCTACATTTGTATTTATTTTAACTTATTTACATATATTAAGAGGATTAAATTATTCATATTCATATTTACCATTATCATGGATATCTGGATTAATAATATTCCTAATTTCTATTGTTACTGCTTTTATGGGTTATGTATTACCTTGGGGTCAAATGAGTTTCTGGGGTGCAACCGTTATAACTAATTTATTATATTTTATACCTGGACTTGTTTCATGGATTTGTGGTGGATATATTATTAGTGATCCAACTTTAAAAAGATTTTTTGTATTACATTTTATATTTCCATTTATAGCTTTATGTATTGTATTTATACATATATTCTTTTTACACTTACAAGGTAGCTCTAATCCTTTAGGATATGATACTGCTTTAAAAATACCTTTCTATCCAAGTCTATTATGTCTAGATATTAAAGGATTTAATAATGTATTAGTCTTATTTCTAGCACAAAGTTTATTTGGAATATT | 479 | Asia |
| *Plasmodium* | MONFAI01 | MK493382 | Full | 1 | PORUF03 | GCAACAGGTGCTTCATTTGTTTTTATTTTAACTTATTTACATATTTTAAGAGGATTAAATTATTCATATTCATATTTACCTTTATCATGGATTTCAGGATTATTAATATTTTTAATATCTATAGTAACAGCTTTTATGGGTTATGTATTACCTTGGGGTCAAATGAGTTTCTGGGGTGCTACTGTTATAACTAATTTATTATATTTTATACCTGGACTTGTCTCATGGATTTGTGGTGGATATCTTGTAAGTGACCCAACCTTAAAAAGATTCTTTGTATTACATTTTACATTCCCATTTATAGCTTTATGTATTGTATTTATACATATATTCTTCTTACATTTACAAGGTAGCACAAATCCTTTAGGGTATGATACAGCTTTAAAAATACCCTTCTATCCAAATCTATTAAGTCTTGATATTAAAGGATTTAATAATGTATTAGTTTTATTCTTATCACAAAGTTTATTTGGAATTCT | 479 | Asia |
| *Haemoproteus* | MW3 | MF118164 | Full | 1 | RW3 | CTACTGGAGCTACATTTGTTTTTATTTTAACTTACTTACATATTTTAAGAGGATTAAATTACTCATATTCATATTTACCTTTATCATGGATATCTGGATTAATAATATTCTTAATCTCTATTGTTACTGCTTTTATGGGTTATGTTTTACCTTGGGGTCAAATGAGTTTCTGGGGTGCAACCGTTATTACTAATTTATTATATTTTATTCCTGGACTTGTATCATGGATTTGTGGTGGATATATTATTAGTGATCCAACACTAAAAAGATTCTTTGTATTACATTTTATATTTCCATTTATAGCTTTATGTATTGTTTTTATACATATATTCTTCTTACATTTACAAGGTAGCTCTAATCCTTTAGGATATGATACAGCTTTAAAAATACCTTTCTATCCAAGTCTATTATGTTTAGATATTAAAGGATTTAATAATGTATTAGTTATATTTTTAGCTCAAAGTTTATTTGGTATTCT | 478 | North_Africa_._Middle_East |
| *Haemoproteus* | MYMAC03 | KY305008 | Partial | 1 | MYIMAC01 | TTAAATTATTCATATTCATATTTACCTCTTTCATGGATATCTGGACTAATTATATTTTTAATTTCTATAGTTACTGCTTTTATGGGTTATGTATTACCTTGGGGTCAAATGAGTTTCTGGGGTGCAACCGTTATAACTAATTTATTATATTTTATACCTGGACTTGTTTCATGGATTTGTGGTGGATATACTATTAGTGATCCAACTTTAAAAAGATTCTTTGTATTACATTTTATATTTCCATTTATAGCTTTATGTATTGTATTTATACACATATTTTTCTTACATTTACAAGGTAGCACTAATCCTTTAGGATATGATACAGCTTTAAAAATACCTTTCTATCCAAGTCTATTATGTCTAGATATTAAAGGATTTAATAACGTATTAGTCCTATTTCTAGCACAAAGTTTATTTGGAATTTT | 425 | South_America |
| *Haemoproteus* | MYIFLA01 | KF482360 | Full | 1 | SITTAKRU1 | GCTACAGGTGCTACATTTGTATTTATTTTAACTTACCTACATATTTTAAGAGGACTAAACTATTCATACTCTTACTTACCTTTATCATGGATAACTGGATTAATAATATTCTTAATTTCTATTGTTACTGCTTTTATGGGTTATGTATTACCTTGGGGTCAAATGAGTTTCTGGGGTGCAACCGTTATTACTAATTTACTATATTTTATACCTGGACTTGTTTCATGGATTTGTGGAGGATATACTATTAGTGATCCAACTTTAAAAAGATTTTTTGTATTACATTTTATATTCCCTTTTATAGCCCTATGTATTGTATTTATACATATATTTTTCTTACACTTACAAGGTAGCTCTAATCCTTTAGGATATGATACAGCTTTAAAAATACCTTTCTATCCAAGTCTATTATGTCTAGATATTAAAGGATTTAATAATGTATTAGTCCTATTTCTAGCACAAAGTTTATTTGGAATACT | 479 | South_America |
| *Haemoproteus* | MYIMAC01 | OM273857 | Full | 1 | MYMAC03 | TACTGGTGCTACATTTGTTTTTATTTTAACATATTTACATATTTTAAGAGGTTTAAATTATTCATATTCATATTTACCTCTTTCATGGATATCTGGACTAATTATATTTTTAATTTCTATAGTTACTGCTTTTATGGGTTATGTATTACCTTGGGGTCAAATGAGTTTCTGGGGTGCAACCGTTATAACTAATTTATTATATTTTATACCTGGACTTGTTTCATGGATTTGTGGTGGATATACTATTAGTGATCCAACTTTAAAAAGATTCTTTGTATTACATTTTATATTTCCATTTATAGCTTTATGTATTGTATTTATACACATATTTTTCTTACATTTACAAGGTAGCACTAATCCTTTAGGATATGATACAGCTTTAAAAATACCTTTCTATCCAAGTCTATTATGTCTAGATATTAAAGGATTTAATAACGTATTAGTCCTATTTCTAGCACAAAGTTTATTTGGAATTTT | 477 | South_America |
| *Haemoproteus* | MYISWA01 | KU562174 | Full | 1 | ELALB01 | TACTGGTGCTACATTTGTTTTTATTTTAACATATTTACATATTTTAAGAGGTTTAAATTATTCATATTCATATTTACCTTTATCATGGATATCTGGATTAATTATATTTTTAATTTCTATAGTTACTGCATTTATGGGTTATGTATTACCTTGGGGTCAAATGAGTTTCTGGGGTGCAACCGTTATAACTAATTTATTATATTTTATACCTGGACTTGTTTCATGGATTTGCGGTGGATATACAATTAGTGATCCAACTTTAAAAAGATTCTTTGTATTACATTTTATATTTCCATTTATAGCTTTATGTATTGTATTTATACATATATTTTTCTTACATTTACAAGGTAGCTCTAATCCTTTAGGATATGATACAGCTTTAAAAATACCTTTCTATCCAAGTCTATTATGTCTAGATATTAAAGGATTTAATAATGTATTAGTCCTATTTCTAGCACAAAGTTTATTTGGAATTTT | 477 | South_America |
| *Plasmodium* | NEOFAS06 | KU562576 | Full | 1 | VOLJAC01 | AACAGGTGCTTCATTTGTTTTCATTCTAACATATTTACATATTTTAAGAGGATTAAATTATTCATATTCATATTTACCTTTATCATGGATTTCAGGATTATTAATATTTCTAATATCTATAGTTACTGCTTTTATGGGTTATGTATTACCTTGGGGTCAAATGAGTTTCTGGGGTGCTACAGTTATAACTAATTTATTATATTTTATACCTGGACTTGTCTCATGGATTTGTGGTGGATATCTTGTAAGTGACCCAACTTTAAAAAGATTTTTCGTATTACATTTTACATTTCCATTTATAGCTTTATGTATTGTATTTATACATATATTCTTCTTACATTTACAAGGTAGCACAAATCCTTTAGGGTATGATACAGCTTTAAAAATACCCTTCTATCCAAATCTATTAAGTCTTGATATTAAAGGATTTAATAATGTATTAGTTTTATTCTTATCTCAAAGTTTATTTGGAATTTT | 477 | South_America |
| *Haemoproteus* | NISALB02 | MZ502247 | Full | 1 | STTRA01 | GCTACTGGTGCTACATTTGTTTTTATATTAACATATTTACATATCTTAAGAGGATTAAATTATTCATATTCATATTTACCTTTATCATGGATAACTGGACTAATGATTTTCTTAATTTCTATTGTTGCTGCTTTTATGGGTTATGTATTACCTTGGGGTCAAATGAGTTTCTGGGGTGCAACCGTTATTACTAACTTATTATATTTTATACCTGGACTTGTTTCATGGATTTGTGGTGGATATAATATTAGTGATCCTACTTTAAAAAGATTCTTTGTATTACATTTTATATTTCCATTTGTAGCTTTATGTATTGTATTTATACATATATTCTTTTTACACTTACAAGGTAGCTCTAATCCTTTAGGATATGATACAGCTTTAAAAATACCTTTCTATCCAAGTCTATTATGTTTAGATATTAAAGGATTTAGTAATGTATTAGTATTATACTTAGCTCAAAGTTTATTTGGTATATT | 479 | Asia |
| *Plasmodium* | NYCNYC01 | KU057967 | Full | 1 | LEVER02 | GCAACAGGTGCTTCATTTGTTTTTATTTTAACTTACCTACATATTTTAAGAGGACTAAATTATTCATACTCATACTTACCATTATCATGGATATCTGGATTAATAATATTCTTAATATCTATAGTTACAGCTTTTATGGGTTATGTATTACCTTGGGGTCAAATGAGTTTCTGGGGTGCTACTGTAATAACTAATTTACTTTATTTTATTCCTGGACTTGTCTCATGGATTTGTGGTGGATATCTTGTAAGTGACCCAACCTTAAAAAGATTCTTTGTATTACATTTTACATTTCCATTTATAGCTTTATGTATTGTATTTATACATATCTTCTTTTTACATTTACAAGGTAGCACTAATCCTTTAGGGTATGATACAGCTTTAAAAATACCCTTCTATCCAAATCTTTTAAGTCTTGATATTAAAGGATTTAATAATGTATTAGTATTATTTTTAGCACAAAGTTTATTTGGAATATT | 479 | North_America, South_America |
| *Leucocytozoon* | OTUSCO03 | MT281494 | Full | 1 | OTSCO02 | TCAACAGGAGCATCTTTTGTATTTATATTAACATATCTACATATTCTAAGAGGATTAAATTATTCATTCTCCTACTTACCTTTATCATGGATAAGTGGTTTAGTTATCTTTTGTTTATTTATTGTAACTGCTTTTATGGGTTATGTCTTACCATGGGGACAAATGAGTTTCTGGGGAGCTACTGTTATTACTAATTTATTATACTTTATTCCTGGATTAATCAATTGGGTTTGCGGTGGATTTATTATTAATGATCCAACACTAAAAAGATTCTTCGTATTACATTTTATATTCCCATTTATAGCACTAGCTATTGTATTTATTCATATATTCTTCTTACATATTCAAGGTAGCACTAATCCTTTAGGGTATGATACCCCTTTAAAAATACCATTCTATCCAAGTCTATTAACTTTAGATGTTAAAGGATTTCATTATGTATTAGTAATATTCTTATTCCAAAGCTTATTTGGTATTGC | 479 | Asia |
| *Haemoproteus* | OTULEM01 | LC230124 | Full | 1 | STTRA01 | GCTACTGGTGCTACATTTGTTTTTATATTAACATATTTACATATCTTAAGAGGATTAAATTATTCATATTCATATTTACCTTTATCATGGATAACTGGACTAATGATTTTTTTAATTTCTATTGTTACTGCTTTTATGGGTTATGTATTACCTTGGGGTCAAATGAGTTTCTGGGGTGCAACCGTTATTACTAACTTATTATATTTTATACCTGGACTTGTTTCATGGATTTGTGGTGGATATAATATTAGTGATCCTACTTTAAAAAGATTCTTTGTATTACATTTTATATTTCCATTTGTAGCTTTATGTATTGTATTTATACATATATTCTTTTTACACTTACAAGGTAGCTCTAATCCTTTAGGATATGATACAGCTTTAAAAATACCTTTCTATCCAAGTCTATTATGTTTAGATATTAAAGGATTTAGTAATGTATTAGTATTATACTTAGCTCAAAGTTTATTTGGTATATT | 479 | Asia |
| *Haemoproteus* | OTUSCO02 | MT281474 | Full | 1 | STTRA01 | GCTACTGGTGCTACATTTGTTTTTATATTAACATATTTACATATCTTAAGAGGATTAAATTATTCATATTCATATTTACCTTTATCATGGATAACTGGACTAATAATTTTCTTAATTTCTATTGTTACTGCTTTTATGGGTTATGTATTACCTTGGGGTCAAATGAGTTTCTGGGGTGCAACCGTTATTACTAACTTATTATATTTTATACCTGGACTTGTTTCATGGATTTGTGGTGGATATAATATTAGTGATCCTACTTTAAAAAGATTCTTTGTATTACATTTTATATTTCCATTTGTAGCTTTATGTATTGTATTTATACATATATTCTTTTTACACTTACAAGGTAGCTCTAATCCTTTAGGATATGATACAGCTTTAAAAATACCTTTCTATCCAAGTCTATTATGTTTAGATATTAAAGGATTTAGTAATGTATTAGTATTATACTTAGCTCAAAGTTTATTTGGTATATT | 479 | Asia |
| *Haemoproteus* | PACPOL01 | KF482354 | Full | 1 | STRZON01 | GCTACTGGTGCTACATTTGTATTTATTTTAACTTATTTACATATATTAAGAGGATTAAATTATTCATATTCCTATTTACCTTTATCATGGATAACTGGATTAATAATATTCTTAATTTCTATTGTTACTGCTTTTATGGGTTATGTATTACCTTGGGGTCAAATGAGTTTCTGGGGTGCAACCGTTATAACAAATTTATTATATTTTATTCCTGGACTTGTTTCATGGATTTGTGGTGGATATATTATTAGTGATCCAACTTTAAAAAGATTTTTTGTATTACATTTTATATTTCCATTTATAGCTTTATGTATTGTATTTATACATATATTCTTTTTACATTTACAAGGTAGCTCTAATCCTTTAGGATATGATACTGCTTTAAAAATACCTTTCTATCCAAGTCTATTATGTCTAGATATTAAAGGATTTAATAATGTATTAGTCTTATTTCTAGCACAAAGTTTATTTGGTATTTT | 479 | South_America |
| *Haemoproteus* | PADOM32 | MG976549 | Full | 1 | PSADEC01 | GCTACCGGTGCTACATTTGTTTTTATTTTAACATATCTACATATCTTAAGAGGGTTAAATTATTCATATTCTTATTTACCTTTATCATGGATAACTGGATTAGTTATATTCTTAATTTCTATTGTTACTGCTTTTATGGGTTATGTATTACCTTGGGGTCAAATGAGTTTCTGGGGTGCAACCGTTATTACTAATTTATTATATTTTATACCTGGACTTGTTTCATGGATTTGTGGAGGATATACTATTAGTGATCCAACTTTAAAAAGATTCTTTGTATTACATTTTATATTTCCTTTTATAGCTTTATGTATTGTATTTATACATATATTCTTCTTACACTTACAAGGTAGCTCTAATCCTTTAGGATATGATACAGCTTTAAAAATACCTTTCTATCCAAGTCTATTATGTCTAGATATCAAAGGATTTAATAATGTATTAGTCCTATTTCTAGCACAAAGTTTATTTGGAATTCT | 479 | Asia |
| *Plasmodium* | PARMIN02 | LC701760 | Full | 1 | PARUS09 | GCAACTGGTGCTTCATTTGTTTTCATTTTAACTTATTTACATATTTTAAGAGGATTAAATTATTCATATTCATATTTACCTTTATCATGGATTTCAGGATTATTAATATTTTTAATATCTATAGTAACAGCTTTTATGGGTTATGTATTACCTTGGGGTCAAATGAGTTTCTGGGGTGCTACTGTTATAACTAATTTATTATATTTTATACCTGGACTTGTCTCATGGATTTGTGGTGGATATCTTGTAAGTGACCCAACCTTAAAAAGATTCTTTGTATTACATTTTACATTCCCATTTATAGCTTTATGCATTGTATTTATACATATATTCTTCTTACATTTACAAGGTAGCACAAATCCTTTAGGGTATGATACAGCTTTAAAAATACCCTTCTATCCAAATCTATTAAGTCTTGATATTAAAGGATTTAATAATGTATTAGTTTTATTCTTATCACAAAGTTTATTTGGAATTTT | 479 | Asia |
| *Plasmodium* | PARUS09 | EF380159 | Full | 1 | PARMIN02 | GGTGCTACTGTTATAACTAATTTATTATATTTTATACCTGGACTTGTCTCATGGATTTGTGGTGGATATCTTGTAAGTGACCCAACCTTAAAAAGATTCTTTGTATTACATTTTACATTCCCATTTATAGCTTTATGCATTGTATTTATACATATATTCTTCTTACATTTACAAGGTAGCACAAATCCTTTAGGGTATGATACAGCTTTAAAAATACCCTTCTATCCAAATCTATTAAGTCTTGATATTAAAGGATTTAATAATGTATTAGTTTTATTCTTATCACAAAGTTTATTTGGAATTTT | 305 | Asia |
| *Leucocytozoon* | PARUS93 | MZ571115 | Full | 1 | PARUS90 | CAACAGGTGCATCATTTGTATTTATATTAACATACTTACATATATTAAGAGGATTAAATTATTCTTTTACTTACTTACCATTATCATGGATAAGTGGTTTAGTAATATTCTTAATATTTATTGTAACTGCTTTTATGGGTTACGTATTACCATGGGGTCAAATGAGTTTTTGGGGAGCTACTGTTATAACAAATTTATTATACTTTATTCCTGGATTAATTAATTGGGTTTGTGGTGGATTCATTATTAATGACCCAACTTTAAAAAGATTCTTTGTATTACATTTTATATTCCCATTTGTAGCATTAGCTATTGTATTTATACATATATTTTTCTTACATATTCAAGGTAGCACTAATCCTTTAGGGTATGATACACCTTTAAAAATACCATTCTATCCAAATCTATTAACTTTAGATGTTAAAGGATTTAACTACGTATTAGTATTATTCCTATTTCAAAGTTTATTTGGAATT | 476 | Europe |
| *Haemoproteus* | PARVEN01 | KT757575 | Partial | 1 | FICZAN14 | TCAACAGGTGCATCTTTTGTATTTATATTAACATATCTACATATCTTAAGAGGATTAAATTATTCTTTCTCTTATTTACCTTTATCATGGTATAGTGGTTTAATTATATTCTTAATACTTATTGTAACTGCTTTTATGGGTTACGTTTTACCATGGGGACAAATGAGTTTCTGGGGAGCAACTGTAATTACTAACTTATTATATTTTATTCCTGGATTAATTAATTGGGTCTGTGGTGGATTTATTATTAATGACCCTACACTAAAAAGATTCTTCGTATTACATTTTATATTCCCATTTGTAGCCTTAGCTATTGTATTTATTCATATATTCTTCTTACATATTCATGGTAGCAATAATCCTTTAGGGTATGATACACCTTTAAAAATACCATTCTATCCAAAT | 405 | Asia |
| *Leucocytozoon* | PASDIF03 | MW546961 | Full | 1 | AFR238 | TCAACAGGTGCATCTTTTGTATTTATATTAACCTATCTACATATATTAAGAGGATTAAACTATTCTTTCTCTTACTTACCTTTATCATGGTATAGTGGTTTAGTTATATTCTTAATCTTTATTGTAACTGCTTTTATGGGTTACGTCTTACCATGGGGACAAATGAGTTTCTGGGGAGCAACTGTAATTACTAATTTATTATATTTTATTCCTGGATTAATTAATTGGGTCTGTGGTGGATTTATTATTAATGACCCAACATTAAAAAGATTCTTTGTATTACACTTTATATTCCCATTTGTAGCATTAGCTATTGTATTTATTCATATATTCTTTTTACATATTCATGGTAGCACTAATCCTTTAGGGTATGATACACCTTTAAAAATACCATTCTATCCAAATCTATTAACCTTAGATATTAAAGGATTTAACTATGTATTAGTTATATTTTTATTTCAAAGTTTATTTGGAATTGC | 479 | South_Sahara |
| *Haemoproteus* | PEUCAS02 | ON455496 | Full | 1 | COLPAS01 | GCAACAGGTGCATGTTTTGTATTTATTTTAACATACTTACATATTCTAAGAGGATTGAATTATTCCTATTCATATTTACCATTATCATGGATTACCGGTTTATTAATATTTCTAATCTCTATTGTAACTGCTTTTATGGGTTACGTATTACCTTGGGGTCAAATGAGTTTCTGGGGTGCAACAGTTATTACTAATTTACTTTATTTTATACCTGGATTAGTCTCATGGATTTGTGGTGGATATATTGTTAGTGACCCTACCCTAAAAAGATTCTTTGTATTACATTTTATATTTCCTTTTATAGCTATATGTATAGTATTTATACATATATTCTTTCTACATTTACAAGGTAGCTCTAATCCTTTAGGATATGATACAGCTTTAAAAATACCCTTCTATCCAAGTCTATTATGCCTAGATATTAAAGGTTTTAATAACGTATTAGTATTATTCTTAGCTCAAAGCTTATTTGGAATAT | 478 | North_America |
| *Haemoproteus* | PHEMEL02 | KJ584595 | Full | 1 | PSADEC01 | CTACCGGTGCTACATTTGTTTTTATTCTAACTTATCTACATATCTTAAGAGGATTAAACTATTCATATTCATATTTACCTTTATCATGGATAACTGGATTAGTAATATTCTTAATTTCAATAGTTACCGCTTTTATGGGTTATGTATTACCTTGGGGTCAAATGAGTTTCTGGGGTGCAACCGTTATTACTAATTTATTATATTTTATACCTGGACTTGTTTCATGGATTTGTGGAGGATATACTATTAGTGATCCAACTTTAAAAAGATTCTTTGTATTACATTTTATATTTCCTTTTATAGCTTTATGTATTGTATTTATACATATATTCTTCTTACACTTACAAGGTAGCTCTAATCCTTTAGGATATGATACAGCTTTAAAAATACCTTTCTATCCAAGTCTATTATGTCTAGATATCAAAGGATTTAATAATGTATTAGTCCTATTTCTAGCACAAAGTTTATTTGGAATTCT | 478 | North_America, South_America |
| *Haemoproteus* | PHFRU01 | EF153654 | Full | 1 | PHRFRU07 | ATTTGTTTTTATTCTAACATACTTACATATTTTAAGAGGGTTAAACTATTCATATTCTTACTTACCTTTATCATGGATAACTGGATTAGTTATATTCTTAATTTCAATTGTTACCGCTTTTATGGGTTATGTATTACCTTGGGGTCAAATGAGTTTCTGGGGTGCAACCGTTATTACTAATTTATTATATTTTATTCCTGGACTTGTTTCATGGATTTGTGGAGGATATACTATTAGTGATCCAACTCTAAAAAGATTTTTTGTATTACATTTTATCTTTCCTTTTATAGCTTTATGTATTGTATTTATACATATATTCTTCTTACACTTACAAGGTAGCTCTAATCCTTTAGGATATGATACAGCTTTAAAAATACCTTTCTATCCAAGTCTATTATGTCTAGATATCAAAGGATTTAATAATGTATTAGTCCTATTTCTAGCACAAAGTTTATTTGGAATTCT | 465 | South_America |
| *Haemoproteus* | PHFRU02 | EF153653 | Full | 1 | PHRFRU14 | ACTTACTTACATATCTTAAGAGGATTAAATTATTCATATTCATATTTACCTTTATCATGGATAACTGGACTAATTATATTCTTAATTTCTATTGTTACCGCTTTTATGGGTTATGTATTACCTTGGGGACAAATGAGTTTCTGGGGTGCAACCGTTATTACTAATTTATTATATTTTATACCTGGACTTGTTTCATGGATATGTGGAGGTTATACTATTAGTGATCCAACTTTAAAAAGATTTTTTGTATTACATTTTATATTTCCTTTTATAGCTTTATGTATTGTATTTATACATATATTCTTCTTACACTTACAAGGTAGCTCTAATCCTTTAGGATATGATACAGCTTTAAAAATACCTTTCTATCCAAGTCTATTATGTCTAGATATCAAAGGATTTAATAATGTATTAGTCCTATTTCTAGCACAAAGTTTATTTGGAATCCT | 449 | South_America |
| *Haemoproteus* | PHRFRU07 | EF153654 | Full | 1 | PHFRU01 | GCTACCGGTGCTACATTTGTTTTTATTCTAACATACTTACATATTTTAAGAGGGTTAAACTATTCATATTCTTACTTACCTTTATCATGGATAACTGGATTAGTTATATTCTTAATTTCAATTGTTACCGCTTTTATGGGTTATGTATTACCTTGGGGTCAAATGAGTTTCTGGGGTGCAACCGTTATTACTAATTTATTATATTTTATTCCTGGACTTGTTTCATGGATTTGTGGAGGATATACTATTAGTGATCCAACTCTAAAAAGATTTTTTGTATTACATTTTATCTTTCCTTTTATAGCTTTATGTATTGTATTTATACATATATTCTTCTTACACTTACAAGGTAGCTCTAATCCTTTAGGATATGATACAGCTTTAAAAATACCTTTCTATCCAAGTCTATTATGTCTAGATATCAAAGGATTTAATAATGTATTAGTCCTATTTCTAGCACAAAGTTTATTTGGAATTCT | 479 | South_America |
| *Haemoproteus* | PHRFRU10 | MN136015 | Full | 1 | PSADEC01 | GCTACCGGTGCTACATTTGTCTTTATTTTAACTTACTTACATATCTTAAGAGGATTAAACTATTCATATTCTTATTTACCTTTATCATGGATAACTGGATTAGTAATATTCTTAATTTCAATTGTTACCGCTTTTATGGGTTATGTATTACCTTGGGGTCAAATGAGTTTCTGGGGTGCAACCGTTATTACTAATTTATTATATTTTATACCTGGACTTGTTTCATGGATTTGTGGAGGATATACTATTAGTGATCCAACTTTAAAAAGATTCTTTGTATTACATTTTATATTTCCTTTTATAGCTTTATGTATTGTATTTATACATATATTCTTCTTACACTTACAAGGTAGCTCTAATCCTTTAGGATATGATACAGCTTTAAAAATACCTTTCTATCCAAGTCTATTATGTCTAGATATCAAAGGATTTAATAATGTATTAGTCCTATTTCTAGCACAAAGTTTATTTGGAATTCT | 479 | South_America |
| *Haemoproteus* | PHRFRU14 | ON399824 | Full | 1 | PHFRU02 | TACTGGTGCTACATTTGTCTTTATTTTAACTTACTTACATATCTTAAGAGGATTAAATTATTCATATTCATATTTACCTTTATCATGGATAACTGGACTAATTATATTCTTAATTTCTATTGTTACCGCTTTTATGGGTTATGTATTACCTTGGGGACAAATGAGTTTCTGGGGTGCAACCGTTATTACTAATTTATTATATTTTATACCTGGACTTGTTTCATGGATATGTGGAGGTTATACTATTAGTGATCCAACTTTAAAAAGATTTTTTGTATTACATTTTATATTTCCTTTTATAGCTTTATGTATTGTATTTATACATATATTCTTCTTACACTTACAAGGTAGCTCTAATCCTTTAGGATATGATACAGCTTTAAAAATACCTTTCTATCCAAGTCTATTATGTCTAGATATCAAAGGATTTAATAATGTATTAGTCCTATTTCTAGCACAAAGTTTATTTGGAATCCT | 477 | South_America |
| *Haemoproteus* | PHRPLE01 | MN135996 | Full | 1 | MELGEO01 | GCTACCGGTGCTACATTTGTTTTTATTCTAACTTACTTACATATTTTAAGAGGGTTAAACTATTCATATTCATATTTACCTTTATCATGGATAACTGGATTATTTATATTCTTAATTTCAATTGTTACCGCTTTTATGGGTTATGTATTACCTTGGGGTCAAATGAGTTTCTGGGGTGCAACCGTTATTACTAATTTATTATATTTTATTCCTGGACTTGTTTCATGGATTTGTGGAGGATATACTATTAGTGATCCAACTTTAAAAAGATTTTTTGTATTACATTTTATATTTCCTTTTATAGCTTTATGTATTGTATTCATACATATATTCTTCTTACACTTACAAGGTAGCTCTAATCCTTTAGGATATGATACAGCTTTAAAAATACCTTTCTATCCAAGTCTATTATGTCTAGATATCAAAGGATTTAATAATGTATTAGTCCTATTTCTAGCACAAAGTTTATTTGGAATTCT | 479 | South_America |
| *Haemoproteus* | PHSIB2 | MH513601 | Full | 1 | GW5 | CTACTGGTGCTACATTTGTCTTTATTTTAACTTATTTACATATATTAAGAGGACTAAATTATTCATATTCATATTTACCTTTATCATGGATATCTGGATTATTAATATTCTTAATTTCTATTGTTACTGCTTTTATGGGTTATGTATTACCTTGGGGTCAAATGAGTTTCTGGGGTGCAACCGTTATAACTAATTTATTATATTTTATACCTGGACTTGTTTCATGGATTTGTGGTGGATATATTATTAGTGATCCAACTTTAAAAAGATTTTTTGTATTACATTTTATATTCCCATTTATAGCTTTATGTATTGTATTTATACATATATTCTTTTTACACTTACAAGGTAGCTCTAATCCTTTAGGATATGATACTGCTTTAAAAATACCTTTCTATCCAAGTCTATTATGTCTAGATATTAAAGGATTTAATAATGTATTAGTCTTATTTCTAGCACAAAGTTTATTTGGAATATT | 478 | Europe |
| *Leucocytozoon* | PHYBOR02 | MG726120 | Full | 1 | CATUST11 | TCTACAGGTGCATCTTTTGTCTTTATATTAACATATCTACATATCTTAAGAGGTTTAAATTATTCATTCTCTTATTTACCTTTATCATGGTATACAGGTTTAATAATATTCTTAATATTCATTGTAACTGCTTTTATGGGTTACGTATTACCATGGGGACAAATGAGTTTCTGGGGAGCAACTGTTATTACTAATTTATTATATTTTATTCCTGGATTAATCAATTGGGTATGTGGTGGATTTATTATTAATGATCCAACCCTAAAAAGATTCTTCGTATTACATTTCATATTCCCATTTGTAGCTTTAGCTATTGTATTTATTCATATATTCTTCTTACATATTCATGGTAGCACTAATCCTTTAGGGTATGATACACCTCTAAAAATACCATTCTATCCAAATCTATTAACTTTAGATATTAAAGGATTTAACTATGTATTAGTTATATTCTTATTCCAAAGTTTATTTGGAATTGC | 479 | North_America |
| *Leucocytozoon* | PICVIR01 | KJ488902 | Full | 1 | PARUS37 | TCAACAGGTGCATCATTTGTATTTATATTAACATACCTACATATATTAAGAGGATTAAATTATTCTTTTACTTACTTACCTTTATCATGGATAAGTGGTTTAGTAATATTCTTAATATTTATTGTAACTGCTTTTATGGGTTATGTCTTACCATGGGGTCAAATGAGTTTTTGGGGAGCTACTGTCATTACTAATTTATTATATTTTATTCCTGGACTAATTAATTGGGTTTGTGGTGGATTTATTATTAACGATCCAACTCTAAAAAGATTCTTCGTATTACATTTTATATTCCCATTTGTAGCACTAGCTATTGTATTTATACATATATTCTTCTTACATATTCAAGGTAGCACTAATCCTTTAGGGTATGATACACCTTTAAAAATACCATTCTATCCAAATCTATTAACTCTAGATGTTAAAGGATTAAACTATGTATTAGTATTATTCCTATTTCAAAGTTTATTTGGAATTGC | 479 | Europe |
| *Haemoproteus* | PIRFLA08 | MK216095 | Full | 1 | PSADEC01 | GCTACCGGTGCTACATTTGTTTTTATTTTAACTTACTTACATATATTAAGAGGATTAAAYTATTCATATTCTTATTTACCTTTATCATGGATAACTGGATTAGTAATATTCTTAATTTCAATTGTTACCGCTTTTATGGGTTATGTATTACCTTGGGGTCAAATGAGTTTCTGGGGTGCAACCGTTATTACTAATTTATTATATTTTATACCTGGACTTGTTTCATGGATTTGTGGAGGATATACTATTAGTGATCCAACTTTAAAAAGATTCTTTGTATTACATTTTATATTTCCTTTTATAGCTTTATGTATTGTATTTATACATATATTCTTCTTACACTTACAAGGTAGCTCTAATCCTTTAGGATATGATACAGCTTTAAAAATACCTTTCTATCCAAGTCTATTATGTCTAGATATCAAAGGATTTAATAATGTATTAGTCCTATTTCTAGCACAAAGTTTATTTGGAATTCT | 479 | - |
| *Haemoproteus* | PIRFLA09 | MK216034 | Full | 1 | PSADEC01 | CTACCGGTGCTACATTTGTTTTTATTCTAACTTACTTACATATCCTAAGAGGATTAAACTATTCATATTCTTATTTACCTTTATCATGGATAACTGGATTAGTAATATTCTTAATTTCAATTGTTACCGCTTTTATGGGTTATGTATTACCTTGGGGTCAAATGAGTTTCTGGGGTGCAACCGTTATTACTAATTTATTATATTTTATACCTGGACTTGTTTCATGGATTTGTGGAGGATATACTATTAGTGATCCAACTTTAAAAAGATTCTTTGTATTACATTTTATATTTCCTTTTATAGCTTTATGTATTGTATTTATACATATATTCTTCTTACACTTACAAGGTAGCTCTAATCCTTTAGGATATGATACAGCTTTAAAAATACCTTTCTATCCAAGTCTATTATGTCTAGATATCAAAGGATTTAATAATGTATTAGTCCTATTTCTAGCACAAAGTTTATTTGGAATTCT | 478 | North_America |
| *Haemoproteus* | PIRLUD08 | MK783148 | Full | 1 | PSADEC01 | GCTACCGGTGCTACATTTGTGTTTATTCTAACTTACTTACATATCTTAAGAGGATTAAACTATTCATATTCATATTTACCTTTATCATGGATAACTGGATTAGTAATATTCTTAATTTCAATAGTTACCGCTTTTATGGGTTATGTATTACCTTGGGGTCAAATGAGTTTCTGGGGTGCAACCGTTATTACTAATTTATTATATTTTATACCTGGACTTGTTTCATGGATTTGTGGAGGATATACTATTAGTGATCCAACTTTAAAAAGATTCTTTGTATTACATTTTATATTTCCTTTTATAGCTTTATGTATTGTATTTATACATATATTCTTCTTACACTTACAAGGTAGCTCTAATCCTTTAGGATATGATACAGCTTTAAAAATACCTTTCTATCCAAGTCTATTATGTCTAGATATCAAAGGATTTAATAATGTATTAGTCCTATTTCTAGCACAAAGTTTATTTGGAATTCT | 479 | North_America |
| *Plasmodium* | POEATR02 | MW876833 | Full | 1 | BAEBIC01 | AACAGGTGCTTCATTTGTATTTATTTTAACTTATTTACATATTTTAAGAGGATTAAATTATTCATATTCATATTTACCTTTATCATGGATATCTGGACTAATTATATTTTTAATATCTATTGTAACAGCTTTTATGGGTTATGTATTACCTTGGGGTCAAATGAGTTTCTGGGGTGCTACAGTTATTACTAATTTATTATATTTTATACCTGGACTTGTTTCATGGATATGTGGTGGATATCTTGTAAGTGACCCAACCTTAAAAAGATTCTTTGTATTACATTTTACATTTCCATTTATAGCCTTATGTATTGTATTTATACATATATTCTTTTTACATTTACAAGGTAGCACAAATCCTTTAGGGTATGATACAGCTTTAAAAATACCCTTCTATCCAAATCTTTTAAGTCTTGACATTAAAGGATTTAATAATGTATTAGTATTATTTTTAGCACAAAGTTTATTTGGAATACT | 477 | North_America |
| *Haemoproteus* | POEATR07 | MW876836 | Full | 1 | DENPET01 | TACTGGAGCTACATTTGTATTTATTCTTACTTACTTACATATTTTAAGAGGATTAAATTATTCATATTCATATTTACCTTTATCATGGATTACTGGACTGGTAATATTTTTAATTTCTATTGTTACTGCTTTTATGGGTTATGTTTTACCTTGGGGTCAAATGAGTTTCTGGGGTGCAACCGTTATTACTAATTTATTATATTTTATACCTGGACTTGTTTCATGGATTTGTGGTGGATATACTATAAGTGATCCAACTCTAAAAAGATTTTTTGTATTACATTTTATATTTCCTTTTATAGCTTTATGCATCGTATTTATACATATATTCTTCTTACATTTACAAGGTAGCTCTAATCCTTTAGGATATGATACAGCTTTAAAAATACCTTTCTATCCAAGTCTATTATGTTTAGATATTAAAGGATTTAATAATGTATTAGTTATATTTTTAGCACAAAGTTTATTTGGTATTTT | 477 | North_America |
| *Haemoproteus* | PSABIF02 | KU562233 | Full | 1 | PSAVIR01 | TACCGGTGCCACATTTGTCTTTATTCTAACTTACTTACATATATTAAGAGGATTAAACTATTCATACTCTTACTTACCTTTATCATGGATAACTGGATTAGTAATATTCTTAATTTCAATTGTTACTGCATTTATGGGTTATGTATTACCTTGGGGTCAAATGAGTTTCTGGGGTGCAACCGTTATAACTAATTTATTATATTTTATACCTGGACTTGTTTCATGGATTTGTGGAGGATATACTATTAGTGATCCAACTTTAAAAAGATTTTTTGTATTACATTTTATATTCCCTTTTATAGCTTTATGTATTGTATTTATACATATTTTCTTCTTACACTTACAAGGTAGCTCTAATCCTTTAGGATATGATACAGCTTTAAAAATACCTTTCTATCCAAGTCTATTATGTCTAGATATCAAAGGATTTAATAACATATTAGTCCTATTTCTAGCACAAAGTTTATTTGGAATTCT | 477 | South_America |
| *Haemoproteus* | PTIPER04 | MK061646 | Full | 1 | PTIPER02 | GCTACTGGTGCTACATTTGTTTTTATTTTAACTTATTTACATATTTTAAGAGGATTAAATTATTCATATTCATATTTACCTTTATCATGGATAACTGGATTAATAATATTTTTAATTTCTATAGTTACTGCTTTTATGGGTTATGTATTACCTTGGGGTCAAATGAGTTTCTGGGGTGCAACCGTTATTACTAATTTATTATATTTTATACCTGGACTTGTTTCATGGATTTGTGGAGGATATACAATAAGTGATCCAACTTTAAAAAGATTTTTTGTATTACATTTTATATTCCCTTTTATAGCTTTATGTATTGTATTTATACATATATTCTTCTTACATTTACAAGGTAGCTCTAATCCTTTAGGATATGATACAGCTTTAAAAATACCTTTCTATCCAAGTCTATTATGTCTAGATATTAAAGGATTTAATAATGTATTAGTCCTATTTCTAGCACAAAGTTTATTTGGTATATT | 479 | Oceania |
| *Haemoproteus* | PTIVIC02 | JX021542 | Full | 1 | MACFLA01 | GCTACTGGTGCTACATTTGTTTTTATTTTAACTTATTTACATATATTAAGAGGATTAAATTATTCATATTCATATTTACCTTTATCATGGATAACTGGATTATGTATATTCTTAATTTCTATTGTTACTGCTTTTATGGGTTATGTATTACCTTGGGGTCAAATGAGTTTCTGGGGTGCAACCGTTATAACTAATTTATTATATTTTATACCTGGACTAGTTTCATGGATTTGTGGTGGATATATTATTAGTGATCCAACTTTAAAAAGATTTTTTGTATTACATTTTATATTTCCATTTATAGCTTTATGTATTGTGTTTATACATATATTCTTTTTACACTTACAAGGTAGCACTAATCCTTTAGGATATGATACAGCTTTAAAAATACCTTTCTATCCAAGTCTATTATGTCTAGATATAAAAGGATTTAATAATGTATTAGTCTTATTTCTAGCACAAAGTTTATTTGGAATATT | 479 | Australia_._New_Zeeland |
| *Haemoproteus* | QUIQUI01 | MF817765 | Full | 1 | MELGEO01 | GCTACCGGTGCTACATTTGTTTTTATTTTAACTTACTTACATATCTTAAGAGGATTAAACTATTCATATTCTTATTTACCTTTATCATGGATAACTGGATTAGTAATATTCTTAATTTCTATTGTTACCGCTTTTATGGGTTATGTATTACCTTGGGGTCAAATGAGTTTCTGGGGTGCAACCGTTATTACTAATTTATTATATTTTATACCTGGACTTGTTTCATGGATTTGTGGAGGATATACTATTAGTGATCCAACTTTAAAAAGATTTTTTGTATTACATTTTATATTTCCTTTTATAGCTTTATGTATTGTATTCATACATATATTCTTCTTACACTTACAAGGTAGCTCTAATCCTTTAGGATATGATACAGCTTTAAAAATACCTTTCTATCCAAGTCTATTATGTCTAGATATCAAAGGATTTAATAATGTATTAGTCCTATTTCTAGCACAAAGTTTATTTGGAATTCT | 479 | North_America |
| *Plasmodium* | RBQ15 | EF117215 | Partial | 1 | HYPAM01 | ATTCTTTGTATTACACTNCATATTTCCGTNNNNNNNNNNNNGTATTGTATTTATACNTATANTCTTTTTACATTTACAAGGNAGCACAAATCCTTTAGGGTATGATACAGCTTTAAAAATACCCTTCTATCCAAATCTNTTAANTCTTGATATTAAAGGATTTAATAATNTATTAGTNTTATTTTTAGCACAAAGTTTATTTGGAATATT | 210 | South_Sahara |
| *Plasmodium* | RBQ18 | EF117211 | Partial | 1 | CXPIP01 | TCNTNCATTNNNAAGGNAGCACNAATCCTTTAGGGTATGATACAGNTTTTAAAANACCCTTCTATCCANNTCTTTTAAGTCTTGATATTAAAGGATTTANTAACGTGTTAGTATTATTTTTAGCACAAAGTTTATTTGGAATATT | 145 | South_Sahara |
| *Leucocytozoon* | RECOB3 | DQ847221 | Full | 1 | RS4 | TCAACAGGTGCATCTTTTGTATTTATATTAACATATCTACATATATTAAGAGGATTAAACTATTCTTTCTCTTACTTACCTTTATCATGGTATAGTGGTTTAATTATATTCTTAATCTTTATTGTAACTGCTTTTATGGGTTACGTCTTACCATGGGGACAAATGAGTTTCTGGGGAGCAACTGTAATTACTAATTTATTATATTTTATTCCTGGATTAATTAATTGGGTCTGTGGTGGATTTATTATTAATGACCCAACATTAAAAAGATTCTTTGTATTACACTTTATATTCCCATTTGTAGCATTAGCTATTGTATTTATTCATATATTCTTTTTACATATTCATGGTAGCACTAATCCTTTAGGGTATGATACACCTTTAAAAATACCATTCTATCCAAATCTATTAACCTTAGATATTAAAGGATTTAACTATGTATTAGTTATATTTTTATTTCAAAGTTTATTTGGAATTG | 478 | Europe, South_Sahara, North_Africa_._Middle_East |
| *Plasmodium* | RECOB4 | DQ847260 | Full | 1 | NEOLI01 | GCAACAGGTGCATCATTTGTATTTATTCTTACTTATTTACATATTTTAAGAGGATTAAACTATTCTTATTCATATTTACCTTTATCATGGATATCAGGATTAATAATATTTTTAATATCAATAGTAACTGCTTTTATGGGATATGTATTACCTTGGGGTCAAATGAGTTTCTGGGGTGCAACTGTTATTACCAACTTACTCTACTTTATACCTGGTCTTGTTTCATGGATTTGTGGTGGATATCTTGTAAGTGATCCAACATTAAAAAGATTTTTTGTATTACATTTTATATTTCCATTTATAGCTTTATGTATTGTGTTTATACATATATTCTTTCTACATTTACAAGGTAGCACAAATCCTTTAGGATATGATACAGCTTTAAAAATACCCTTCTATCCAAATCTATTAAGTCTTGATATTAAAGGATTTAATAATATCTTAGTTTTATTTTTAGCACAAAGTTTATTTGGAATATT | 479 | South_Sahara, South_America |
| *Plasmodium* | RHYSIM01 | KU562769 | Full | 1 | SALCOE01 | AACTGGAGCTTCATTTGTATTTATTTTAACTTATTTACATATTTTAAGAGGATTAAATTATTCTTATTCATATTTACCTTTATCATGGATTTCAGGACTAATTATATTTTTAATATCTATAGTAACTGCTTTTATGGGTTATGTATTACCTTGGGGTCAAATGAGTTTTTGGGGAGCAACTGTAATTACCAATTTATTATATTTTATTCCAGGACTTGTATCATGGATTTGTGGTGGATATCTTGTTAGTGACCCAACACTAAAAAGATTTTTTGTATTACATTTTACATTTCCATTTATAGCTTTATGTATTGTATTTATACATATATTCTTTTTACATTTACAAGGTAGCACTAATCCTTTAGGGTATGATACAGCTTTAAAAATACCCTTCTATCCAAATCTATTAAGTCTCGATATTAAAGGATTTAATAATATATTAGTACTATTTTTAGCACAAAGTTTATTTGGAATCTT | 477 | North_America, Central_America, South_America |
| *Haemoproteus* | ROFI3 | JX556907 | Full | 1 | MELGEO01 | GCTACCGGTGCTACATTCGTTTTTATTTTAACTTACTTACATATATTAAGAGGATTAAACTACTCATATTCTTATTTACCTTTATCATGGATAACTGGATTAGTAATATTCTTAATCTCTATTGTTACCGCTTTTATGGGTTATGTATTACCTTGGGGTCAAATGAGTTTCTGGGGTGCAACCGTTATTACTAATTTATTATATTTCATACCTGGACTTGTTTCATGGATTTGTGGAGGATATACTATTAGTGATCCAACTTTAAAAAGATTTTTTGTATTACATTTTATATTTCCTTTTATAGCTTTATGTATTGTATTCATACATATATTCTTCTTACACTTACAAGGTAGCTCTAATCCTTTAGGATATGATACAGCTTTAAAAATACCTTTCTATCCAAGTCTATTATGTCTAGATATCAAAGGATTTAATAATGTATTAGTCCTATTTCTAGCACAAAGTTTATTTGGAATTCT | 479 | Europe |
| *Plasmodium* | ROFI5 | JX556909 | Full | 1 | RBQ16 | GCAACAGGTGCTTCATTTGTATTTATCTTAACTTATTTACATATTTTAAGAGGATTAAATTATTCATATTCATATTTACCTTTATCATGGATATCTGGACTAGTCATATTTTTAATATCTATTGTAACAGCTTTTATGGGTTATGTATTACCTTGGGGTCAAATGAGTTTCTGGGGTGCTACAGTTATAACTAATTTATTATATTTTATACCTGGACTTGTTTCATGGATATGTGGTGGATATCTTGTAAGTGACCCAACTTTAAAAAGATTCTTTGTATTACATTTTACATTTCCATTTATAGCTTTATGTATTGTATTTATACATATATTCTTTTTACATTTACAAGGTAGCACAAATCCTTTAGGGTATGATACAGCTTTAAAAATACCCTTCTATCCAAATCTTTTAAGTCTTGATATTAAAGGATTTAATAATGTATTAGTATTATTTTTAGCACAAAGTTTATTTGGAATATT | 479 | Europe |
| *Plasmodium* | SALMAX02 | KU562838 | Full | 1 | RBQ16 | AACAGGTGCTTCATTTGTATTTATTTTAACTTATTTACATATTTTAAGAGGATTAAATTATTCATATTCATATTTACCTTTATCATGGATATCTGGATTAATTATATTTTTAATATCTATTGTAACAGCTTTTATGGGTTATGTATTACCTTGGGGTCAAATGAGTTTCTGGGGTGCTACAGTTATTACTAATTTATTATATTTTATACCTGGACTTGTTTCATGGATATGTGGTGGATATCTTGTAAGTGACCCAACCTTAAAAAGATTCTTTGTATTACATTTTACATTTCCATTTATAGCTTTATGTATTGTATTTATACATATATTCTTTTTACATTTACAAGGTAGCACAAATCCTTTAGGGTATGATACAGCTTTAAAAATACCCTTCTATCCAAATCTTTTAAGTCTTGATATTAAAGGATTTAATAATGTATTAGTATTATTTTTAGCACAAAGTTTATTTGGAATATT | 477 | South_America |
| *Haemoproteus* | SALMAX03 | MN459369 | Full | 1 | SERUT04 | CTACCGGTGCTACATTTGTTTTTATTCTAACTTACTTACATATTTTAAGAGGATTAAACTATTCATTTTCTTATTTACCTTTATCATGGATAACTGGATTAGTTATATTCTTAATTTCAATTGTTACCGCTTTTATGGGTTATGTATTACCTTGGGGTCAAATGAGTTTCTGGGGTGCAACCGTTATTACTAATTTATTATATTTTATACCTGGACTTGTTTCATGGATTTGTGGAGGATATACTATTAGTGATCCAACTTTAAAAAGATTCTTTGTATTACATTTTATATTTCCTTTTATAGCTTTATGTATTGTATTTATTCATATATTCTTCTTACACTTACAAGGTAGCTCTAATCCTTTAGGATATGATACAGCTTTAAAAATACCTTTCTATCCAAGTCTATTATGTCTAGATATCAAAGGATTTAATAATGTATTAGTCCTATTTCTAGCACAAAGTTTATTTGGTATTCT | 478 | South_America |
| *Plasmodium* | SATOR02 | MF442564 | Full | 1 | SATOR01 | GCAACAGGTGCATCATTTGTATTTATTCTTACTTATTTACACATTTTAAGAGGATTAAATTACTCTTACTCTTATTTACCTTTATCATGGATATCAGGATTATTAATATTTTTAATATCAATAGTAACTGCCTTTATGGGATATGTATTACCTTGGGGTCAAATGAGTTTCTGGGGTGCTACTGTTATTACCAATTTATTATACTTTATACCTGGTCTTGTTTCATGGATCTGTGGTGGATATCTTGTAAGTGACCCAACATTAAAAAGATTCTTTGTTTTACATTTTATATTTCCATTTATAGCTTTATGTATCGTATTTATACATATATTCTTTTTACATTTACAAGGTAGCACAAATCCTTTAGGGTATGATACAGCTTTAAAAATACCCTTCTATCCAAATCTATTAAGTCTTGATATTAAAGGATTTAATAATATATTTGTTTTATTTTTAGCACAAAGTCTATTTGGAATATT | 479 | South_Sahara |
| *Haemoproteus* | SCLCAU03 | MN458625 | Full | 1 | SERUT05 | CTACCGGTGCTACATTTGTTTTTATTCTAACTTACTTACATATCTTAAGAGGATTAAACTATTCATATTCTTATTTACCTTTATCATGGATAACTGGATTAGTTATATTCTTAATTTCAATTGTTACCGCTTTTATGGGTTATGTATTACCTTGGGGTCAAATGAGTTTCTGGGGTGCAACCGTTATTACTAATTTATTATATTTTATACCTGGACTTGTTTCATGGATTTGTGGAGGATATACTATTAGTGATCCAACTTTAAAAAGATTCTTTGTATTACATTTTATATTCCCTTTTATAGCTTTATGTATTGTATTTATTCATATATTCTTCTTACACTTACAAGGTAGCTCTAATCCTTTAGGATATGATACAGCTTTAAAAATACCTTTCTATCCAAGTCTATTATGTCTAGATATCAAAGGATTTAATAATGTATTAGTCCTATTTCTAGCACAAAGTTTATTTGGTATTCT | 478 | South_America |
| *Plasmodium* | SEIAUR01 | DQ838988 | Full | 1 | TABI08 | GCAACAGGTGCTTCATTTGTATTTATTTTAACTTATTTACATATTTTAAGAGGATTAAATTATTCATATTCATATTTACCTTTATCATGGATATCTGGATTACTTATATTTTTAATATCTATTGTAACAGCTTTTATGGGTTATGTATTACCTTGGGGTCAAATGAGTTTCTGGGGTGCTACAGTTATTACTAATTTATTATATTTTATACCTGGACTTGTTTCATGGATATGTGGTGGATATCTTGTAAGTGACCCAACCTTAAAAAGATTCTTTGTATTACATTTTACATTTCCATTTATAGCCTTATGTATTGTATTTATACATATATTCTTTTTACATTTACAAGGTAGCACAAATCCTTTAGGGTATGATACAGCTTTAAAAATACCCTTCTATCCAAATCTTTTAAGTCTTGATATTAAAGGATTTAATAATGTATTAGTATTATTCTTAGCACAAAGTTTATTTGGAATATT | 479 | North_America, Central_America, South_America |
| *Plasmodium* | SEIAUR04 | MW081126 | Full | 1 | SETPEN01 | TTTGTATTTATTCTTACTTATCTACATATTTTAAGAGGATTAAATTATTCTTATTCTTATTTACCTTTATCATGGATATCAGGATTAATAATATTCTTAATATCAATAGTAACTGCTTTTATGGGATATGTATTACCTTGGGGTCAAATGAGTTTCTGGGGTGCAACTGTCATTACTAATTTATTATATTTTATACCTGGTCTTGTTTCATGGATTTGTGGTGGATATCTTGTAAGCGACCCAACATTAAAAAGATTTTTTGTATTACATTTTATATTCCCATTTATAGCCTTATGTATTGTATTTATACATATATTCTTTCTACATTTACAAGGTAGCACAAATCCTTTAGGGTATGATACAGCTTTAAAAATACCCTTCTATCCAAATCTATTAAGTCTTGATATTAAAGGATTTAATAATATTTTAGTTTTATTTTTAGCACAAAGTTTATTTGGAATAT | 463 | North_America |
| *Haemoproteus* | SETAUD02 | MF752572 | Partial | 1 | MELGEO01 | ACTTACTTACATATATTAAGAGGATTAAACTATTCATATTCTTATTTACCTTTATCATGGATAACTGGATTAGTAATATTCTTAATCTCTATTGTTACCGCTTTTATGGGTTATGTATTACCTTGGGGTCAAATGAGTTTCTGGGGTGCAACCGTTATTACTAATTTATTATATTTTATACCTGGACTTGTTTCATGGATTTGTGGAGGATATACTATTAGTGATCCAACTTTAAAAAGATTTTTTGTATTACATTTTATATTTCCTTTTATAGCTTTATGTATTGTATTCATACATATATTCTTCTTACACTTACAAGGTAGCTCTAATCCTTTAGGATATGATACAGCTTTAAAAATACCTTTCTATCCAAGTCTATTATGTCTAGATATCAAAGGATTTAATAATGTATTAGTCCTA | 420 | North_America |
| *Haemoproteus* | SETAUD05 | MF752575 | Full | 1 | SERUT04 | CTACCGGTGCTACATTTGTTTTTATTCTAACTTACTTACATATTTTAAGAGGATTAAACTATTCATATTCTTATTTACCTTTATCATGGATAACTGGATTAGTTATATTCTTAATTTCAATTGTTACCGCTTTTATGGGTTATGTATTACCTTGGGGTCAAATGAGTTTCTGGGGTGCAACCGTTATTACTAATTTATTATATTTTATACCTGGACTTGTTTCATGGATTTGTGGAGGATATACTATTAGTGATCCAACTTTAAAAAGATTCTTTGTATTACATTTTATATTTCCTTTTATAGCTTTATGTATTGTATTTATTCATATATTCTTCTTACACTTACAAGGTAGCTCTAATCCTTTAGGATATGATACAGCTTTAAAAATACCTTTCTATCCAAGTCTATTATGTCTAGATATCAAAGGATTTAATAATGTATTAGTCCTATTTCTAGCACAAAGTTTATTTGGTATTCT | 478 | North_America, Central_America |
| *Haemoproteus* | SETAUD07 | MF752588 | Full | 1 | TABI05 | CTACCGGAGCTACATTTGTATTTATTCTTACTTACTTACATATTTTAAGAGGATTAAATTATTCATATTCATATTTACCTTTATCATGGATTACTGGATTGGTAATATTTTTAATTTCTATTGTTACTGCTTTTATGGGTTATGTTTTACCTTGGGGTCAAATGAGTTTCTGGGGTGCAACCGTTATTACTAATTTATTATATTTTATACCTGGACTTGTTTCATGGATTTGTGGTGGATATACTATAAGTGATCCAACTCTAAAAAGATTTTTTGTATTACATTTTATATTTCCTTTTATAGCTTTATGCATCGTATTTATACATATATTCTTCTTACATTTACAAGGTAGCTCTAATCCTTTAGGATATGATACAGCTTTAAAAATACCTTTCTATCCAAGTCTATTATGTTTAGATATTAAAGGATTTAATAATGTATTAGTTATATTTTTAGCACAAAGTTTATTTGGTATTCT | 478 | North_America |
| *Haemoproteus* | SETAUD09 | MF752617 | Full | 1 | MELGEO01 | ACTTACTTACATATATTAAGAGGATTAAACTATTCATATTCTTATTTACCTTTATCATGGATAACTGGATTAGTAATATTCTTAATTTCTATTGTTACCGCTTTTATGGGTTATGTATTACCTTGGGGTCAAATGAGTTTCTGGGGTGCAACCGTTATTACTAATTTATTATATTTTATACCTGGACTTGTTTCATGGATTTGTGGAGGATATACTATTAGTGATCCAACTTTAAAAAGATTTTTTGTATTACATTTTATATTTCCTTTTATAGCTTTATGTATTGTATTCATACATATATTCTTCTTACACTTACAAGGTAGCTCTAATCCTTTAGGATATGATACAGCTTTAAAAATACCTTTCTATCCAAGTCTATTATGTCTAGATATCAAAGGATTTAATAATGTATTAGTCCTA | 420 | North_America |
| *Haemoproteus* | SETAUD22 | MF752637 | Partial | 1 | VIRFLA02 | ATATTTTAAGAGGATTAAATTATTCATATTCATATTTACCTTTATCATGGATAACTGGACTAATAATATTTTTAATTTCTATTGTTACAGCTTTTATGGGTTATGTATTACCTTGGGGTCAAATGAGTTTCTGGGGTGCAACCGTTATTACTAATTTATTATATTTTATACCTGGACTTGTTTCATGGATTTGTGGAGGATATACTATTAGTGATCCCACTTTAAAAAGATTTTTTGTATTACATTTTATATTTCCTTTTATAGCTTTATGTATTGTATTTATACATATATTTTTCTTACACTTACAAGGTAGCTCTAATCCTTTAGGATATGATACGGCTTTAAAAATACCTTTCTATCCAAGTCTATTATG | 373 | North_America |
| *Leucocytozoon* | SETAUD25 | MF752690 | Full | 1 | DENCOR06 | CAACCGGTGCATCTTTTGTATTTATATTAACATATCTACATATACTAAGAGGTTTAAATTACTCTTTCTCTTACTTACCTTTATCATGGATAAGTGGTTTAGTAATATTCTTAATATTTATTGTAACTGCTTTTATGGGTTATGTCTTACCATGGGGTCAAATGAGTTTCTGGGGAGCTACTGTAATTACTAACTTATTATATTTTATTCCTGGATTAATTAATTGGGTTTGTGGTGGTTTTATTATTAACGATCCAACTCTAAAAAGATTCTTTGTATTACATTTTATATTCCCATTCGTAGCTTTAGCTATTGTATTTATTCATATATTCTTCTTACATATTCAAGGTAGCACTAATCCATTAGGGTATGATACACCTTTAAAAATACCATTCTATCCAAATCTATTAACTTTAGATGTTAAAGGATTTAATTATGTATTAGTAATATTTTTATTTCAAAGTTTATTTGGTATT | 476 | North_America |
| *Leucocytozoon* | SETCOR06 | MH753108 | Full | 1 | SETAUD27 | TCAACAGGTGCATCATTTGTCTTTATATTAACATACTTACATATATTAAGAGGATTAAATTATTCATTTACTTACTTACCTTTATCATGGATAAGTGGTTTAATAATATTCTTAATATTTATTGTAACTGCTTTTATGGGTTATGTCTTACCATGGGGTCAAATGAGTTTTTGGGGAGCTACTGTTATAACTAATTTATTATATTTTATTCCTGGATTAATTAATTGGGTTTGCGGTGGATTTATTATTAACGACCCAACTCTAAAAAGATTCTTCGTATTACATTTTATATTCCCATTTGTAGCATTAGCTATCGTATTTATACATATATTCTTCTTACATATTCAAGGTAGCACTAATCCTTTAGGGTATGATACACCTTTAAAAATACCATTCTATCCAAATCTATTAACTTTAGATGTTAAAGGATTTAACTATGTATTAGTATTATTCCTATTTCAAAGTTTATTTGGAATTGC | 479 | North_America, Central_America |
| *Plasmodium* | SETPEN01 | ON455432 | Full | 1 | SEIAUR04 | GCAACAGGTGCATCATTTGTATTTATTCTTACTTATCTACATATTTTAAGAGGATTAAATTATTCTTATTCTTATTTACCTTTATCATGGATATCAGGATTAATAATATTCTTAATATCAATAGTAACTGCTTTTATGGGATATGTATTACCTTGGGGTCAAATGAGTTTCTGGGGTGCAACTGTCATTACTAATTTATTATATTTTATACCTGGTCTTGTTTCATGGATTTGTGGTGGATATCTTGTAAGCGACCCAACATTAAAAAGATTTTTTGTATTACATTTTATATTCCCATTTATAGCCTTATGTATTGTATTTATACATATATTCTTTCTACATTTACAAGGTAGCACAAATCCTTTAGGGTATGATACAGCTTTAAAAATACCCTTCTATCCAAATCTATTAAGTCTTGATATTAAAGGATTTAATAATATTTTAGTTTTATTTTTAGCACAAAGTTTATTTGGAATAT | 478 | North_America |
| *Haemoproteus* | SPIARB01 | KF314762 | Full | 1 | MELGEO01 | GCTACCGGTGCTACATTTGTTTTTATTTTAACTTACTTACATATTTTAAGAGGATTAAACTACTCATATTCTTATTTACCTTTATCATGGATAACTGGATTAGTAATATTCTTAATCTCTATTGTTACCGCTTTTATGGGTTATGTATTACCTTGGGGTCAAATGAGTTTCTGGGGTGCAACCGTTATTACTAATTTATTATATTTTATACCTGGACTTGTTTCATGGATTTGTGGAGGATATACTATTAGTGATCCAACTTTAAAAAGATTTTTTGTATTACATTTTATATTTCCTTTTATAGCTTTATGTATTGTATTCATACATATATTCTTCTTACACTTACAAGGTAGCTCTAATCCTTTAGGATATGATACAGCTTTAAAAATACCTTTCTATCCAAGTCTATTATGTCTAGATATCAAAGGATTTAATAATGTATTAGTCCTATTTCTAGCACAAAGTTTATTTGGAATTCT | 479 | North_America |
| *Haemoproteus* | SPISEN01 | MF374494 | Full | 1 | STSEN1 | GCTACTGGTGCTACATTTGTTTTTATATTAACATATTTACATATCTTAAGAGGATTAAATTATTCATATTCATACTTACCTTTATCATGGATAACTGGACTATTAATCTTCTTAATTTCTATTGTTACTGCTTTTATGGGTTATGTATTACCTTGGGGTCAAATGAGTTTCTGGGGTGCAACCGTTATTACTAACTTATTATATTTCATACCTGGACTTGTTTCATGGATTTGTGGTGGATATAATATTAGTGATCCTACTTTAAAAAGATTTTTTGTATTACATTTTATATTCCCATTTATAGCTTTATGTATTGTATTTATACATATATTCTTCTTACACTTACAAGGTAGCACTAATCCTTTAGGATATGATACAGCTTTAAAAATACCTTTCTATCCAAGTCTATTATGTTTAGATATTAAAGGATTTAGTAATGTATTAGTATTATACTTAGCTCAAAGTTTATTTGGTATACT | 479 | North_Africa_._Middle_East |
| *Plasmodium* | SPMEN03 | JF833046 | Full | 1 | SPMEN01 | GCAACAGGAGCTTCATTTGTATTTATTTTAACTTATCTACATATTTTAAGAGGATTAAACTATTCATACTCATATTTACCTTTATCATGGATATCAGGATTAATAATATTCTTAATATCAATAGTTACAGCTTTTATGGGTTATGTATTACCTTGGGGTCAAATGAGTTTCTGGGGTGCAACTGTTATAACTAATTTATTATATTTTATTCCTGGACTTGTCTCATGGATTTGTGGTGGATATCTTGTAAGTGACCCAACTTTAAAAAGATTTTTTGTATTACATTTTACATTTCCATTTATAGCTTTATGTATTGTATTTATACATATATTCTTTTTACATTTACAAGGTAGCACTAATCCTTTAGGGTATGATACAGCTTTAAAAATACCCTTCTATCCAAATCTATTAAGTCTCGATATAAAAGGATTTAATAATGTATTAGTTTTATTTTTAGCACAAAGTTTATTTGGAATTTT | 479 | Central_America, South_America |
| *Plasmodium* | STEGRA01 | MN104957 | Full | 1 | PV5 | GCAACAGGTGCATCATTTGTATTTATTCTTACTTACTTACATATTTTAAGAGGATTAAATTATTCATATTCATATTTACCTTTATCATGGATATCAGGATTATTAATATTTCTAATATCAATAGTAACTGCTTTTATGGGATATGTATTACCTTGGGGTCAAATGAGTTTTTGGGGTGCAACTGTCATTACTAACTTATTATACTTTATACCTGGTCTTGTTTCATGGATTTGTGGTGGATATCTCGTAAGTGACCCAACATTAAAAAGATTCTTTGTATTACACTTCATATTTCCATTCATAGCTTTATGTATTGTATTTATACATATATTCTTTCTACATTTACAAGGTAGCACAAATCCTTTAGGGTATGATACAGCTTTAAAAATACCCTTCTATCCAAATCTATTAAGTCTTGATATTAAAGGATTTAATAATATCCTAGTTTTATTTTTAGCACAAAGTTTATTTGGAATATT | 479 | - |
| *Plasmodium* | STIERY01 | MN202216 | Full | 1 | PV8 | GCAACAGGTGCATCATTTGTATTTATTCTCACTTATCTACATATTTTAAGAGGATTAAATTATTCATATTCATATTTACCTTTATCATGGATATCAGGATTAATAATATTTCTAATATCAATAGTAACTGCTTTTATGGGATATGTATTACCTTGGGGTCAAATGAGTTTTTGGGGTGCAACTGTTATTACTAATTTATTATATTTTATACCTGGTCTTGTTTCATGGATTTGCGGTGGATATCTTGTAAGCGACCCAACATTAAAAAGATTCTTTGTATTACATTTTATATTTCCGTTTATAGCTTTATGTATTGTGTTTATACATATATTCTTTTTACATTTACAAGGTAGCACAAATCCTTTAGGGTATGATACAGCTTTAAAAATACCCTTCTATCCAAATCTATTAAGTCTTGATATTAAAGGATTTAATAATATTCTAGTTTTATTTTTAGCACAAAGTTTATTTGGAATATT | 479 | - |
| *Haemoproteus* | STRURA03 | MK330140 | Full | 1 | ASOT07 | CTACTGGTGCTACATTTGTTTTTATATTAACATATTTACATATCTTAAGAGGATTAAATTATTCATATTCATATTTACCTTTATCATGGATAACTGGATTAACTATTTTCTTAATTTCTATTGTAACTGCTTTTATGGGTTATGTATTACCTTGGGGTCAAATGAGTTTCTGGGGTGCAACCGTTATTACTAACTTATTATATTTTATTCCTGGACTTGTTTCATGGATTTGTGGTGGATATAATATTAGTGATCCTACTTTAAAAAGATTCTTTATATTACATTTTATATTTCCATTTATAGCTTTATGTATTGTATTTATACATATATTCTTTTTACATTTACAAGGTAGTTCTAATCCTTTAGGATATGATACAGCTTTAAAAATACCTTTCTATCCAAGTCTATTATGTTTAGATATTAAAGGATTTAGTAATATATTAGTATTATATTTAGCTCAAAGTTTATTTGGTATATT | 478 | Europe |
| *Leucocytozoon* | SYCON05 | KP688304 | Full | 1 | SYAT42 | TCAACAGGTGCATCTTTTGTATTTATATTAACATATCTACATATATTAAGAGGATTAAATTATTCTTTCTCTTACTTACCTTTATCATGGTATAGTGGTTTAATTATATTCTTAATCTTTATTGTAACTGCTTTTATGGGTTACGTCTTACCATGGGGACAAATGAGTTTCTGGGGAGCAACTGTAATTACTAATTTATTATATTTTATTCCTGGATTAATTAATTGGGTCTGTGGTGGATTTATTATTAATGACCCAACATTAAAAAGATTCTTTGTATTACATTTTATATTCCCATTTGTAGCATTAGCTATTGTATTTATTCATATATTCTTTTTACATATTCATGGTAGCACTAATCCTTTAGGGTATGATACACCTTTAAAAATACCATTCTATCCAAATCTATTAACCTTAGATATTAAAGGATTTAACTATGTATTAGTTATATTTTTATTTCAAAGTTTATTTGGAATTGC | 479 | Europe |
| *Leucocytozoon* | SYCON06 | KP688305 | Full | 1 | SYBOR25 | TCAACAGGTGCATCTTTTGTATTTATATTAACATATCTACATATATTAAGAGGATTAAACTATTCTTTCTCTTACTTACCTTTATCATGGTATAGTGGTTTAATTATATTCTTAATCTTTATTGTAACTGCTTTTATGGGTTACGTCTTACCATGGGGACAAATGAGTTTCTGGGGAGCAACTGTAATTACTAATTTATTATATTTTATTCCTGGATTAATTAATTGGGTCTGTGGTGGATTTATTATTAATGACCCAACATTAAAAAGATTCTTTGTATTACACTTTATATTCCCATTCGTAGCATTAGCTATTGTATTTATTCATATATTCTTTTTACATATTCATGGTAGCACTAATCCTTTAGGGTATGATACACCTTTAAAAATACCATTCTATCCAAATCTATTAACCTTAGATATTAAAGGATTTAACTATGTATTAGTTATATTTTTATTTCAAAGTTTATTTGGAATTGC | 479 | Europe |
| *Haemoproteus* | SYCUR03 | MZ571109 | Full | 1 | LWT3 | CTACTGGAGCTACATTTGTTTTTATTCTAACTTACTTACATATTTTAAGAGGATTAAATTACTCATACTCATACTTACCTTCATCATGGATAACTGGACTAGTCATATTCTTAATTTCTATCGTTACTGCTTTTATGGGTTATGTTTTACCTTGGGGTCAAATGAGTTTCTGGGGTGCAACTGTTATTACTAATTTATTATATTTTATACCTGGATTAGTTTCATGGATTTGTGGGGGATATACTATAAGTGATCCAACTTTAAAAAGATTTTTTGTATTACATTTTATATTTCCATTTATAGCTTTATGTATTGTCTTTATTCATATATTCTTCTTACATCTACAAGGTAGCTCTAACCCTTTAGGATATGATACAGCTTTAAAAATACCTTTCTATCCAAGTCTATTATGTCTAGATATTAAAGGATTTAATAATGTATTAGTTATATTTTTAGCACAAAGTTTATTTGGAATTCT | 478 | Europe |
| *Haemoproteus* | TACCRI01 | KU562138 | Full | 1 | LEPRUF01 | TACCGGTGCTACATTTGTTTTTATTCTAACTTACTTACATATCTTAAGAGGATTAAACTATTCATATTCTTACTTACCTTTATCATGGATAACTGGATTAGTAATATTCTTAATTTCAATTGTTACTGCATTTATGGGTTATGTATTACCTTGGGGTCAAATGAGTTTCTGGGGTGCAACCGTTATAACTAATTTATTATATTTTATACCTGGACTTGTTTCATGGATTTGTGGAGGATATACTATTAGTGATCCAACTTTAAAAAGATTTTTTGTATTACATTTTATATTCCCTTTTATAGCTTTATGTATTGTATTTATACATATATTCTTCTTACACTTACAAGGTAGCTCTAATCCTTTAGGATATGATACAGCTTTAAAAATACCTTTCTATCCAAGTCTATTATGTCTAGATATCAAAGGATTTAATAATGTATTAGTCCTATTTCTAGCACAAAGTTTATTTGGAATTCT | 477 | South_America |
| *Plasmodium* | TARUF01 | JX021475 | Full | 1 | VOLJAC01 | CAACAGGTGCTTCATTTGTTTTCATTCTAACCTATTTACATATTTTAAGAGGATTAAATTATTCATATTCATATTTACCTTTATCATGGATTTCAGGATTATTAATATTTCTAATATCTATAGTTACTGCTTTTATGGGTTATGTATTACCTTGGGGTCAAATGAGTTTCTGGGGTGCTACAGTTATAACTAATTTATTATATTTTATACCTGGACTTGTCTCATGGATTTGTGGTGGATATCTTGTAAGTGACCCAACTTTAAAAAGATTTTTCGTATTACATTTTACATTTCCATTTATAGCTTTATGTATTGTATTTATACATATATTCTTCTTACATTTACAAGGTAGCACAAATCCTTTAGGGTATGATACAGCTTTAAAAATACCCTTCTATCCAAATCTATTAAGTCTTGATATTAAAGGATTTAATAATGTATTAGTTTTATTCTTATCTCAAAGTTTATTTGGAATTTT | 478 | South_America |
| *Haemoproteus* | THAMEL01 | OQ579011 | Full | 1 | ASCLA01 | TTTGTTTTTATATTAACATATTTACATATCTTAAGAGGATTAAATTATTCATATTCATACTTACCTTTATCATGGATTACTGGATTAATGATTTTCTTAATTTCTATTGTCACTGCTTTTATGGGTTATGTATTACCTTGGGGTCAAATGAGTTTCTGGGGTGCAACCGTTATTACTAACTTATTATATTTCATACCTGGACTTGTTTCATGGATTTGTGGTGGATATAATATTAGTGATCCTACTTTAAAAAGATTCTTTGTATTACATTTTATTTTTCCATTTATAGCTTTATGTATTGTATTTATACATATATTCTTTTTACACTTACAAGGTAGCTCTAATCCTTTAGGATATGATACAGCTTTAAAAATACCTTTCTATCCAAGTCTATTATGTTTAGATATTAAAGGATTTAGTAATGTATTAGTATTATACTTAGCTCAAAGTTTATTTGGTATACT | 464 | - |
| *Plasmodium* | THRPAL01 | MN458578 | Full | 1 | SALCOE01 | CAACTGGAGCTTCATTTGTATTTATTTTAACTTATTTACATATTTTAAGAGGATTAAATTATTCTTATTCATATTTACCTTTATCATGGATTTCAGGACTAATCATATTTTTAATATCTATAGTAACTGCTTTTATGGGTTATGTATTACCTTGGGGTCAAATGAGTTTTTGGGGAGCAACTGTAATTACCAATTTATTATATTTTATTCCAGGACTTGTATCATGGATTTGTGGTGGATATCTTGTTAGTGACCCAACACTAAAAAGATTTTTTGTATTACATTTTACATTTCCATTTATAGCTTTATGTATTGTATTTATACATATATTCTTTTTACATTTACAAGGTAGCACTAATCCTTTAGGGTATGATACAGCTTTAAAAATACCCTTCTATCCAAATCTATTAAGTCTCGATATTAAAGGATTTAATAATATATTAGTACTATTTTTAGCACAAAGTTTATTTGGAATCTT | 478 | South_America |
| *Haemoproteus* | THRSAY01 | MT724390 | Full | 1 | PSADEC01 | TACCGGTGCTACATTTGTTTTTATTCTAACTTACTTACATATTCTAAGAGGATTAAACTATTCATATTCATATTTACCTTTATCATGGATAACTGGATTAGTAATATTCTTAATTTCAATTGTTACCGCTTTTATGGGTTATGTATTACCTTGGGGTCAAATGAGTTTCTGGGGTGCAACCGTTATTACTAATTTATTATATTTTATACCTGGACTTGTTTCATGGATTTGTGGAGGATATACTATTAGTGATCCAACTTTAAAAAGATTCTTTGTATTACATTTTATATTTCCTTTTATAGCTTTATGTATTGTATTTATACATATATTCTTCTTACACTTACAAGGTAGCTCTAATCCTTTAGGATATGATACAGCTTTAAAAATACCTTTCTATCCAAGTCTATTATGTCTAGATATCAAAGGATTTAATAATGTATTAGTCCTATTTCTAGCACAAAGTTTATTTGGAATTCT | 477 | South_America |
| *Haemoproteus* | TOXCUR01 | MZ604568 | Full | 1 | MIMPOL03 | CTACTGGTGCTACATTTGTATTTATTTTAACTTATTTACATATATTAAGAGGATTAAATTATTCATATTCATATTTACCTTTATCATGGATATCTGGATTAATAATATTCTTAATTTCTATAGTTACTGCTTTTATGGGTTATGTATTACCTTGGGGTCAAATGAGTTTCTGGGGTGCAACCGTTATTACTAATTTATTATATTTTATACCTGGACTTGTTTCATGGATTTGTGGTGGATATATTATTAGTGATCCAACTTTAAAAAGATTCTTTGTATTACATTTTATATTCCCATTTATAGCTTTATGTATTGTATTTATACATATATTCTTTTTACACTTACAAGGTAGCTCTAATCCTTTAGGATATGATACTGCTTTAAAAATACCTTTCTATCCAAGTCTATTATGTCTAGATATTAAAGGATTTAATAATGTATTAGTCTTATTTCTAGCACAAAGTTTATTTGGAATATT | 478 | North_America |
| *Leucocytozoon* | TROAED02 | KF767431 | Full | 1 | ZOCAP06 | CAACTGGTGCATCTTTTGTATTTATCTTAACATATCTACATATTTTAAGAGGATTAAACTATTCATTCTCTTACTTACCTTTATCATGGTATAGTGGTTTAGTTATATTCTTAATCTTTATTGTAACTGCTTTTATGGGTTACGTTTTACCATGGGGACAAATGAGTTTCTGGGGAGCAACTGTAATTACTAATTTATTATATTTTATTCCTGGATTAATTAATTGGGTCTGTGGTGGATTCATAATTAATGACCCAACATTAAAAAGATTCTTCGTATTACACTTTATATTCCCATTTGTAGCCTTAGCTATTGTATTTATTCATATATTCTTCTTACATATTCATGGTAGCACTAATCCTTTAGGGTATGATACACCTTTAAAAATACCATTCTATCCAAATCTATTAACTTTAGATATTAAAGGATTTAACTATGTATTAGTTATATTTTTATTTCAAAGTTTATTTGGAATTGC | 478 | South_America |
| *Plasmodium* | TROAED21 | JQ988551 | Full | 1 | TABI07 | CTTACATATTTACATATTTTAAGAGGATTAAATTATTCTTATTCTTATTTACCTTTATCATGGATATCAGGATTAATAATATTTTTAATATCAATAGTTACTGCTTTTATGGGATATGTACTACCTTGGGGTCAAATGAGTTTCTGGGGTGCAACCGCCATTACTAATTTATTATATTTTATACCTGGTCTTGTTTCATGGATCTGTGGTGGATATCTTGTAAGCGACCCAACATTAAAAAGATTTTTTGTATTACATTTTATATTTCCATTTATAGCCTTATGTATTGTATTTATACATATATTCTTTCTACATTTACAAGGTAGCACAAATCCTTTAGGGTATGATACAGCTTTAAAAATACCCTTCTATCCAAATCTATTAAGTCTTGATATTAAAGGATTTAATAATATCTTAGTTTTATTTTTAGCACAAAGCTTATTTGGAATATT | 452 | South_America |
| *Plasmodium* | TROAED24 | KJ620788 | Full | 1 | SIAMEX02 | CAACTGGTGCTTCATTTGTATTTATTTTAACTTATTTACATATTTTAAGAGGACTAAATTATTCATATTCATATTTACCTTTATCATGGATATCTGGATTAATAATATTTTTAATATCTATAGTAACAGCTTTTATGGGTTATGTATTACCTTGGGGTCAAATGAGTTTCTGGGGTGCTACTGTAATTACTAATTTATTATATTTTATACCTGGACTTGTTTCATGGATATGTGGTGGATATCTTGTAAGCGACCCAACTTTAAAAAGATTCTTTGTATTACATTTTACATTTCCATTTATAGCTTTATGTATTGTATTTATACATATATTCTTTTTACATTTACAAGGTAGCACAAATCCTTTAGGGTATGATACAGCTTTAAAAATACCCTTCTATCCAAATCTTTTAAGTCTTGATATTAAAGGATTTAATAATGTACTAGTATTATTTTTAGCACAAAGTTTATTTGGAATATT | 478 | North_America, Central_America |
| *Plasmodium* | TSUB01 | MG598394 | Full | 1 | ZOSBRU01 | CAACAGGTGCATCATTTGTATTTATTCTTACTTATTTACATATTTTAAGAGGATTAAATTACTCATACTCATATTTACCTTTATCATGGATATCTGGATTAATAATATTTTTAATATCAATAGTAACAGCTTTTATGGGATATGTATTACCTTGGGGTCAAATGAGCTTTTGGGGTGCAACTGTTATAACAAATTTACTATACTTTATTCCTGGTCTTGTTTCATGGATTTGTGGTGGATATCTTGTAAGTGACCCAACATTAAAAAGATTTTTTGTATTACATTTTATATTCCCATTTATAGCCTTATGTATTGTATTTATACATATTTTCTTTTTACATTTACAAGGTAGCACAAATCCTTTAGGGTATGATACAGCTTTAAAAATACCCTTCTATCCAAATCTATTAAGTCTTGATATTAAAGGATTTAATAATGTATTAGTTTTATTTTTATCACAAAGCTTATTTGGAATATT | 478 | South_America, Asia |
| *Leucocytozoon* | TUMER02 | GU391354 | Full | 1 | TUREUN01 | ACAGGTGCATCTTTTGTATTTATATTAACATATCTACATATCTTAAGAGGATTAAATTATTCTTTCTCTTATTTACCATTATCATGGTATAGTGGTTTAATTATATTTTTAATCTTTATTGTAACTGCTTTTATGGGTTACGTCTTACCATGGGGACAAATGAGTTTCTGGGGAGCTACTGTAATTACTAATTTATTATACTTTATTCCTGGATTAATCAATTGGGTATGTGGTGGATTCATTATTAATGACCCAACATTAAAAAGATTCTTCGTATTACACTTCATATTCCCATTTGTTGCATTAGCTATTGTATTTATTCATATATTCTTCCTACATATTCATGGTAGCACAAATCCTTTAGGGTATGATACACCTTTAAAAATACCATTCTATCCAAATCTATTAACTTTAGATATTAAAGGATTTAACTATGTATTAGTTATATTTTTATTTCAAAGTTTATTTGGAATTGC | 476 | Europe, North_Africa_._Middle_East |
| *Plasmodium* | TUMIG22 | KM598210 | Full | 1 | TUMIG05 | GCAACAGGTGCTTCATTTGTTTTCATTTTAACCTATTTACATATTTTAAGAGGATTAAATTATTCATATTCATATTTACCTTTATCATGGATTTCAGGATTATTAATATTTTTAATATCTATAGTAACAGCTTTTATGGGTTATGTATTACCTTGGGGTCAAATGAGTTTCTGGGGTGCTACTGTTATAACTAATTTATTATATTTTATACCTGGACTTGTCTCATGGATTTGTGGTGGATATCTTGTAAGTGACCCAACCTTAAAAAGATTTTTTGTATTACATTTTACATTCCCATTTATAGCTTTATGTATTGTATTTATACATATATTCTTCTTACATTTACAAGGTAGCACAAATCCTTTAGGGTATGATACAGCTTTAAAAATACCCTTCTATCCAAATCTATTAAGTCTTGATATTAAAGGATTTAATAATGTATTAGTTTTATTTTTATCTCAAAGTTTATTTGGAATTTT | 479 | Central_America |
| *Leucocytozoon* | TUREUN01 | LC701766 | Full | 1 | TUMER02 | TCAACAGGTGCATCTTTTGTATTTATATTAACATATCTACATATCTTAAGAGGATTAAATTATTCTTTCTCTTATTTACCATTATCATGGTATAGTGGTTTAATTATATTTTTAATCTTTATTGTAACTGCTTTTATGGGTTACGTCTTACCATGGGGACAAATGAGTTTCTGGGGAGCTACTGTAATTACTAATTTATTATACTTTATTCCTGGATTAATCAATTGGGTATGTGGTGGATTCATTATTAATGACCCAACATTAAAAAGATTCTTCGTATTACACTTCATATTCCCATTTGTTGCATTAGCTATTGTATTTATTCATATATTCTTCCTACATATTCATGGTAGCACAAATCCTTTAGGGTATGATACACCTTTAAAAATACCATTCTATCCAAATCTATTAACTTTAGATATTAAAGGATTTAACTATGTATTAGTTATATTTTTATTTCAAAGTTTATTTGGAATTGC | 479 | Asia |
| *Leucocytozoon* | TURFAL05 | MK947548 | Full | 1 | TUFAL04 | AACAGGTGCATCTTTTGTATTTATATTAACATATTTACACATATTAAGAGGATTAAATTATTCTTTCTCTTACTTACCTTTATCATGGATAAGTGGTTTAATTATATTTTTAATATTTATTGTAACTGCTTTTATGGGTTATGTCTTACCATGGGGTCAAATGAGTTTCTGGGGAGCTACAGTAATTACTAATCTATTATATTTTATTCCTGGACTAATTAATTGGGTTTGTGGAGGATTTATTATTAATGACCCAACTCTAAAAAGATTCTTTGTATTACATTTTATATTCCCATTTGTAGCATTAGCAATCGTATTTATACATATATTCTTCTTACATATTCAAGGTAGCACTAATCCTTTAGGGTATGATACACCTTTAAAAATACCATTCTATCCAAATCTATTAACTTTAGATGTTAAAGGATTTAATTATGTAATAGTATTATTCTTATTCCAAAGTTTATTTGGAATTGC | 477 | South_America |
| *Plasmodium* | TURMER06 | MK493395 | Full | 1 | TUBOU01 | GCAACAGGTGCATCATTTGTATTTATTCTTACTTATTTACATATTTTAAGAGGATTAAATTATTCATATTCATACTTACCTTTATCATGGATATCAGGATTAATAATATTTCTAATATCAATAGTAACAGCTTTTATGGGATATGTATTACCTTGGGGTCAAATGAGTTTTTGGGGTGCAACTGTTATAACTAATTTATTATATTTTATACCTGGTCTTGTTTCATGGATCTGTGGTGGATATCTTGTAAGTGACCCAACATTAAAAAGATTCTTTGTTTTACATTTTATATTTCCATTTATAGCTTTATGTATTGTATTTATACATATATTCTTTTTACATTTACAAGGTAGCACAAATCCTTTAGGGTATGATACAGCTTTAAAAATACCCTTCTATCCAAATCTATTAAGTCTTGATATTAAAGGATTTAATAATATCTTAGTCTTATTTTTAGCACAAAGCTTATTTGGAATATT | 479 | Asia |
| *Leucocytozoon* | TURSER01 | MN459538 | Full | 1 | TROAED08 | TCAACAGGTGCATCTTTTGTATTTATATTAACATATTTACATATCTTAAGAGGATTAAATTATTCTTTCTCTTATTTACCTTTATCATGGTATAGTGGTTTAATTATATTTTTAATCTTTATTGTAACTGCTTTTATGGGTTACGTCTTACCATGGGGACAAATGAGTTTCTGGGGAGCTACTGTAATTACTAATTTATTATACTTTATTCCTGGACTAATCAATTGGGTATGTGGTGGATTTATTATTAATGACCCAACATTAAAAAGATTCTTTGTATTACACTTCATATTCCCATTTGTTGCATTAGCTATTGTATTTATTCATATATTCTTCCTACATATTCATGGTAGCACAAATCCTTTAGGGTATGATACACCTTTAAAAATACCATTCTATCCAAATCTATTAACTTTAGATATTAAAGGATTTAACTATGTATTAGTTATATTTTTATTTCAAAGTTTATTTGGAATTGC | 479 | South_America |
| *Haemoproteus* | TURSTR02 | MF565817 | Full | 1 | TURGUL01 | GCTACCGGTGCTACATTTGTATTTATATTAACCTACTTACATATTTTAAGAGGATTAAATTATTCATATTCATATTTACCTTTATCATGGATAACCGGATTAATAATATTCTTAATTTCCATTGTTACCGCTTTTATGGGTTATGTATTACCTTGGGGTCAAATGAGTTTCTGGGGTGCAACCGTTATTACTAATTTATTATATTTTATACCTGGACTTGTATCATGGATTTGTGGAGGATATACTATTAGTGATCCAACTTTAAAAAGATTTTTTGTATTACATTTTATATTTCCTTTTATAGCTTTATGTATTGTATTTATTCATATATTCTTTTTACACTTACAAGGTAGCTCTAATCCTTTAGGATATGATACAGCTTTAAAAATACCTTTCTATCCAAGTCTATTATGTCTAGATATCAAAGGATTTAATAATGTATTAGTTTTATTCCTAGCACAAAGTCTATTTGGAATTCT | 479 | Asia |
| *Haemoproteus* | TURTYM02 |  | Full | 1 | TURTYM01 | GCAACTGGTGCATCATTTGTATTTATTTTAACATACTTACATATTCTAAGAGGATTAAATTATTCTTATTCTTATTTACCATTATCATGGATTACCGGATTAATAATATTTTTAATCTCTATTGTAACTGCTTTTATGGGTTACGTTTTACCTTGGGGTCAAATGAGTTTCTGGGGTGCAACTGTTATAACTAATTTACTATATTTTATTCCTGGATTAGTATCATGGATTTGTGGTGGATATATAGTTAGTGACCCAACCCTAAAAAGATTCTTTGTATTACATTTTATATTTCCATTTATAGCAATATGTATAGTATTTATACATATATTCTTTCTACATTTACAAGGTAGCTCTAATCCTTTAGGATATGATACAGCTTTAAAAATACCCTTCTATCCAAGTCTATTATGTCTAGATATTAAAGGTTTTAATAACGTATTAGTTCTATTCTTAGCTCAAAGTTTATTTGGAATATT | 479 | - |
| *Plasmodium* | TURUF03 | JX021473 | Full | 1 | TURUF01 | CAACAGGTGCATCATTTGTATTTATTCTTACTTACCTACATATTTTAAGAGGATTAAATTATTCTTATTCTTATTTACCTTTATCATGGATATCAGGATTAATAATATTTTTAATATCAATAGTTACAGCTTTTATGGGATATGTATTACCTTGGGGTCAAATGAGTTTCTGGGGTGCTACTGTTATTACTAATTTGTTATATTTTATACCTGGTCTTGTTTCATGGATTTGTGGTGGATATCTTGTTAGTGATCCAACATTAAAAAGATTTTTTGTTTTACATTTTATATTTCCATTTATAGCTTTATGTATTGTATTTATACATATATTCTTTTTACATTTACAAGGTAGCACAAATCCTTTAGGGTATGATACAGCTTTAAAAATACCCTTCTATCCAAATCTATTAAGTCTTGATATTAAAGGATTTAATAATATTCTAGTTTTATTTTTAGCACAAAGTTTATTTGGAATATT | 478 | South_America |
| *Haemoproteus* | TYTAL2 | JN863576 | Full | 1 | TYTAL8 | AAGAGGATTAAATTATTCATATTCATATTTACCATTATCATGGATAACTGGATTATTTATTTTCTTAATTTCTATTGTTACTGCTTTTATGGGTTATGTATTACCTTGGGGTCAAATGAGTTTCTGGGGTGCAACTGTTATTACTAACTTATTATATTTTATACCTGGACTTGTTTCATGGATTTGTGGTGGATATAATATTAGTGATCCTACTTTAAAAAGATTCTTTGTATTACATTTTATATTTCCATTTATAGCATTATGTATTGTATTTATACATATATTCTTTTTACATTTACAAGGTAGCTCTAATCCTTTAGGATATGATACAGCTTTAAAAATACCTTTCTATCCAAGTCTATTATGTTTAGATATTAAAGGATTTAGTAATATATTAGTTTTATATTTAGCTCAAAGTTTATTTGGTATATT | 432 | - |
| *Haemoproteus* | TYTAL8 | OQ447191 | Full | 1 | TYTAL2 | GCTACTGGTGCTACATTTGTTTTTATATTAACATATTTACACATCTTAAGAGGATTAAATTATTCATATTCATATTTACCATTATCATGGATAACTGGATTATTTATTTTCTTAATTTCTATTGTTACTGCTTTTATGGGTTATGTATTACCTTGGGGTCAAATGAGTTTCTGGGGTGCAACTGTTATTACTAACTTATTATATTTTATACCTGGACTTGTTTCATGGATTTGTGGTGGATATAATATTAGTGATCCTACTTTAAAAAGATTCTTTGTATTACATTTTATATTTCCATTTATAGCATTATGTATTGTATTTATACATATATTCTTTTTACATTTACAAGGTAGCTCTAATCCTTTAGGATATGATACAGCTTTAAAAATACCTTTCTATCCAAGTCTATTATGTTTAGATATTAAAGGATTTAGTAATATATTAGTTTTATATTTAGCTCAAAGTTTATTTGGTATATT | 479 | - |
| *Plasmodium* | VERCEL03 | OR063554 | Full | 1 | TABI08 | AACAGGTGCTTCATTTGTATTTATTTTAACTTATTTACATATTTTAAGAGGACTAAATTATTCATATTCATATTTACCTTTATCATGGATATCTGGATTACTTATATTTTTAATATCTATTGTAACAGCTTTTATGGGTTATGTATTACCTTGGGGTCAAATGAGTTTCTGGGGTGCTACAGTTATTACTAATTTATTATATTTTATACCTGGACTTGTTTCATGGATATGTGGTGGATATCTTGTAAGTGACCCAACCTTAAAAAGATTCTTTGTATTACATTTTACATTTCCATTTATAGCCTTATGTATTGTATTTATACATATATTCTTTTTACATTTACAAGGTAGCACAAATCCTTTAGGGTATGATACAGCTTTAAAAATACCCTTCTATCCAAATCTTTTAAGTCTTGATATTAAAGGATTTAATAATGTATTAGTATTATTCTTAGCACAAAGTTTATTTGGAATATT | 477 | - |
| *Haemoproteus* | VIGIL06 | KJ584604 | Partial | 1 | VIGRI01 | TATCTTAAGAGGATTAAATTATTCATATTCATATTTACCTTTATCATGGATAACTGGATTATTAATATTCTTAATTTCTATTGTTACAGCTTTTATGGGTTATGTATTACCTTGGGGTCAAATGAGTTTCTGGGGTGCTACCGTTATTACTAATTTATTATATTTTATACCTGGACTTGTTTCATGGATTTGTGGAGGATATACTATAAGTGATCCAACTTTAAAAAGATTCTTTGTATTACATTTTATATTCCCTTTTATAGCTTTATGTATTGTATTTATACATATATTCTTCTTACATTTACAAGGTAGCTCTAATCCTTTAGGATATGATACAGCTTTAAAAATACCTTTCTATCCAAGTCTATTATGTCTAGATATTAAAGGATTTAATAATGTATTAGTCCTATTTCTAGCACAAAGTTTATTTGGAATTCT | 438 | North_America |
| *Haemoproteus* | VIGIL07 | KJ584605 | Full | 1 | VIGRI01 | CTACTGGTGCTACATTTGTTTTTATTTTAACTTATTTACATATCTTAAGAGGATTAAATTATTCATATTCATATTTACCTTTATCATGGATAACTGGATTATTAATATTCTTAATTTCTATTGTTACAGCTTTTATGGGTTATGTATTACCTTGGGGTCAAATGAGTTTCTGGGGTGCAACCGTTATTACTAATTTATTATATTTTATACCTGGACTTGTTTCATGGATTTGTGGAGGATATACTATAAGTGATCCAACTTTAAAAAGATTCTTTGTATTACATTTTATATTCCCTTTTATAGCTTTATGTATTGTATTTATACATATATTCTTCTTACATTTACAAGGTAGCTCTAATCCTTTAGGATATGATACAGCTTTAAAAATACCTTTCTATCCAAGTCTATTATGTCTAGATATTAAAGGATTTAATAATGTATTAGTCCTATTTCTAGCACAAAGTTTATTTGGAATTCT | 478 | North_America, Central_America |
| *Haemoproteus* | VIRBEL02 | OR063560 | Full | 1 | VIGRI01 | TACCGGTGCTACATTTGTCTTTATTTTAACTTACTTACATATCTTAAGAGGATTAAATTATTCATATTCTTATTTACCTTTATCATGGATAACTGGATTAATAATATTCTTAATTTCTATTGTTACAGCTTTTATGGGTTATGTATTACCTTGGGGTCAAATGAGTTTCTGGGGTGCAACCGTTATTACTAATTTATTATATTTTATACCTGGACTTGTTTCATGGATTTGTGGAGGATATACTATAAGTGATCCAACTTTAAAAAGATTCTTTGTATTACATTTTATATTCCCTTTTATAGCTTTATGTATTGTATTTATACATATATTCTTCTTACATTTACAAGGTAGCTCTAATCCTTTAGGATATGATACAGCTTTAAAAATACCTTTCTATCCAAGTCTATTATGTCTAGATATTAAAGGATTTAATAATGTATTAGTCCTATTTCTAGCACAAAGTTTATTTGGAATTCT | 477 | - |
| *Haemoproteus* | VIRHUT01 | MH091780 | Full | 1 | SERUT02 | TACCGGTGCTACATTTGTTTTTATTCTAACTTACTTACATATCTTAAGAGGATTAAACTATTCATATTCTTATTTACCTTTATCATGGATAACTGGATTAGTAATATTCTTAATTTCAATTGTTACCGCTTTTATGGGTTATGTATTACCTTGGGGTCAAATGAGTTTCTGGGGTGCAACCGTTATTACTAATTTATTATATTTTATACCTGGACTTGTTTCATGGATTTGTGGAGGATATACTATTAGTGATCCAACTTTAAAAAGATTCTTTGTATTACATTTTATATTTCCTTTTATAGCTTTATGTATTGTATTTATTCATATATTCTTCTTACACTTACAAGGTAGCTCTAATCCTTTAGGATATGATACAGCTTTAAAAATACCTTTCTATCCAAGTCTATTATGTCTAGATATCAAAGGATTTAATAATGTATTAGTCCTATTTCTAGCACAAAGTTTATTTGGAATTCT | 477 | Central_America |
| *Plasmodium* | WW3 | AF495577 | Full | 1 | LARINC02 | GCAACAGGTGCTTCATTTGTTTTCATTTTAACCTATTTACATATTTTAAGAGGATTAAATTACTCATATTCATATTTACCTTTATCATGGATTTCAGGATTATTAATATTTTTAATATCCATAGTTACTGCTTTTATGGGTTATGTATTACCTTGGGGTCAAATGAGTTTCTGGGGTGCTACAGTTATAACTAACTTATTATATTTTATACCTGGACTTGTCTCATGGATTTGTGGTGGATATCTTGTAAGTGACCCAACCTTAAAAAGATTTTTTGTATTACATTTTACATTCCCATTTATAGCTTTATGTATTGTATTCATACATATATTCTTCTTACATTTACAAGGTAGCACAAATCCTTTAGGGTATGATACAGCTTTAAAGATACCCTTCTATCCAAATCTATTAAGTCTTGATATTAAAGGATTTAATAATGTATTAGTTTTATTCTTATCTCAAAGTTTATTTGGAATTTT | 479 | Europe, South_Sahara, North_America, Central_America, South_America |
| *Haemoproteus* | ZEGAL05 | GU296215 | Full | 1 | ZEGAL04 | GCCACAGGTGCATCATTTGTATTTATTTTAACATACCTACATATTTTAAGAGGATTAAATTACTCATATTCATATTTACCATTATCATGGATTACCGGATTAATAATATTTATAATCTCTATAATGACTGCTTTCTTGGGTTATGTTCTACCTTGGGGTCAAATGAGTTTCTGGGGTGCAACTGTTATTACTAATTTACTATATTTTATTCCGGGATTAGTATCATGGATTTGTGGTGGTTATATAGTTAGTGATCCTACACTAAAAAGATTCTTTGTATTACATTTTATATTTCCATTTATAGCTATATGTATAGTATTTATACATATATTCTTTTTACATTTACAAGGTAGCTCTAATCCTCTAGGATATGATACAGCATTAAAAATACCATTTTACCCAAATTTACTATGTTTAGATATAAAAGGATTTAATAACGTATTAGTATTATTCTTAGCTCAAAGCTTATTTGGAATATT | 479 | South_America |
| *Haemoproteus* | ZEGAL07 | GU296220 | Full | 1 | ZEGAL06 | GCAACAGGTGCATCATTTGTATTTATTTTAACATACCTACACATTTTAAGAGGATTAAATTACTCATATTCATATTTACCATTATCATGGATTACCGGATTAATCATATTTATAATCTCTATTATGACTGCTTTCTTAGGTTATGTTCTACCTTGGGGTCAAATGAGTTTCTGGGGTGCAACTGTTATTACTAATTTATTATATTTTATTCCAGGATTAGTCTCATGGATTTGTGGTGGATATATTGTTAGTGATCCTACACTAAAAAGATTCTTTGTATTACATTTTATATTTCCATTTATAGCTATATGTATTGTATTTATTCATATATTCTTTTTACATCTACAAGGTAGCTCTAATCCTTTAGGATATGATACAGCATTAAAAATACCATTTTATCCAAATTTACTATGTTTAGATATAAAAGGATTTAATAACGTATTAGTATTATTCTTAT | 457 | South_America |
| *Haemoproteus* | ZOCAP08 | KC480265 | Full | 1 | APSPI01 | GCTACTGGTGCTACATTTGTCTTTATTTTAACTTACTTACATATATTAAGAGGATTAAATTATTCATATTCTTATTTACCTTTATCATGGATAACTGGACTAATAATATTCTTAATTTCTATTGTTACCGCTTTTATGGGTTATGTATTACCTTGGGGACAAATGAGTTTCTGGGGTGCAACCGTTATTACTAATTTATTATATTTTATACCTGGACTTGTTTCATGGATTTGTGGAGGATATACTATTAGTGATCCAACTTTAAAAAGATTTTTTGTACTACATTTTATATTTCCTTTTATAGCTTTATGTATTGTATTTATACATATATTCTTCTTACACTTACAAGGTAGCTCTAATCCTTTAGGATATGATACAGCTTTAAAAATACCTTTCTATCCAAGTCTATTATGTCTAGATATCAAAGGATTTAATAATGTATTAGTCCTATTTCTAGCACAAAGTTTATTTGGAATTCT | 479 | North_America, Central_America, South_America |
| *Plasmodium* | ZOCAP09 | KC480267 | Full | 1 | RBQ16 | GCAACAGGTGCTTCATTTGTATTTATTTTAACTTATTTACATATTTTAAGAGGATTAAATTATTCATATTCATATTTACCATTATCATGGATATCAGGATTAGTCATATTTTTAATATCTATTGTAACTGCTTTTATGGGTTATGTATTACCTTGGGGTCAAATGAGTTTCTGGGGTGCTACAGTTATAACTAATTTATTATATTTTATACCTGGACTTGTTTCATGGATATGTGGTGGATATCTTGTAAGTGACCCAACCTTAAAAAGATTCTTTGTATTACATTTTACATTTCCATTTATAGCTTTATGTATTGTATTTATACATATATTCTTTTTACATTTACAAGGTAGCACAAATCCTTTAGGGTATGATACAGCTTTAAAAATACCCTTCTATCCAAATCTTTTAAGTCTTGATATTAAAGGATTTAATAATGTATTAGTATTATTTTTAGCACAAAGTTTATTTGGAATATT | 479 | South_America |
| *Plasmodium* | ZOCAP11 | KF537281 | Partial | 1 | TABI07 | TTAAGAGGATTAAATTATTCTTATACTTATTTACCTTTATCATGGATATCAGGATTAATAATATTTTTAATATCAATAGTTACTGCTTTTATGGGATATGTACTACCTTGGGGTCAAATGAGTTTCTGGGGTGCAACCGTCATTACTAATTTATTATATTTTATACCTGGTCTTGTTTCATGGATCTGTGGTGGATATCTTGTAAGCGACCCAACATTAAAAAGATTTTTTGTATTACATTTTATATTTCCATTTATAGCCTTATGTATTGTATTTATACATATATTCTTTCTACATTTACAAGGTAGCACAAATCCTTTAGGGTATGATACAGCTTTAAAAATACCCTTCTATCCAAATCTATTAAGTCTTGATATTAAAGGATTTAATAATATCTTAGTTTTATTTTTAGCACAAAGCTTATTTGGAATATT | 434 | South_America |
| *Plasmodium* | ZOCAP12 | KF537291 | Full | 1 | SEIAUR02 | GCAACAGGTGCATCATTTGTATTTATTCTTACATATTTACATATTTTAAGAGGATTAAATTATTCTTATTCTTATTTACCTTTATCATGGATATCAGGATTAATAATATTTTTAATATCAATAGTTACTGCTTTTATGGGATATGTACTACCTTGGGGTCAAATGAGTTTCTGGGGTGCAACCGTCATTACTAATTTATTATATTTTATACCTGGTCTTGTTTCATGGATCTGTGGTGGATATCTTGTAAGCGACCCAACATTAAAAAGATTTTTTGTATTACATTTTATATTTCCATTTATAGCCTTATGTATTGTATTTATACATATATTCTTTCTACATTTACAAGGTAGCACAAATCCTTTAGGGTATGATACAGCTTTAAAAATACCCTTCTATCCAAATCTATTAAGTCTTGATATTAAAGGATTTAATAATATCTTAGTTTTATTTTTAACACAAAGCTTATTTGGAATATT | 479 | South_America |
| *Haemoproteus* | ZOCAP14 | KF537329 | Full | 1 | CHLOP01 | GCTACCGGTGCTACATTTGTTTTTATTCTAACTTACTTACATATCTTAAGAGGATTAAATTATTCATATTCTTATTTACCTTTATCATGGATAACTGGATTAGTTATATTCTTAATTTCAATTGTTACCGCTTTTATGGGTTATGTATTACCTTGGGGTCAAATGAGTTTCTGGGGTGCAACCGTTATAACTAATTTATTATATTTTATTCCTGGACTTGTTTCATGGATTTGTGGAGGATATACTATAAGTGATCCAACTTTAAAAAGATTTTTTGTATTACATTTTATATTTCCTTTTATAGCTTTATGTATTGTATTTATACATATATTCTTCTTACACTTACAAGGTAGCTCTAATCCTTTAGGATATGATACAGCTTTAAAAATACCTTTCTATCCAAGTCTATTATGTCTAGATATCAAAGGATTTAATAATGTATTAGTCCTATTTCTAGCACAAAGTTTATTTGGAATTCT | 479 | South_America |
| *Haemoproteus* | ZOCAP15 |  | Full | 1 | PSADEC01 | CTACCGGTGCTACATTTGTTTTTATTCTAACCTATTTACATATCCTAAGAGGATTAAACTATTCATATTCTTATTTACCTTTATCATGGATAACTGGATTAGTAATATTCTTAATTTCAATTGTTACCGCTTTTATGGGTTATGTATTACCTTGGGGTCAAATGAGTTTCTGGGGTGCAACCGTTATTACTAATTTATTATATTTTATACCTGGACTTGTTTCATGGATTTGTGGAGGATATACTATTAGTGATCCAACTTTAAAAAGATTCTTTGTATTACATTTTATATTTCCTTTTATAGCTTTATGTATTGTATTTATACATATATTCTTCTTACACTTACAAGGTAGCTCTAATCCTTTAGGATATGATACAGCTTTAAAAATACCTTTCTATCCAAGTCTATTATGTCTAGATATCAAAGGATTTAATAATGTATTAGTCCTATTTCTAGCACAAAGTTTATTTGGAATTCT | 478 | South_America |
| *Haemoproteus* | ZOCAP17 | MH444681 | Full | 1 | PSADEC01 | GCTACCGGTGCTACATTTGTTTTTATTTTAACTTACTTACATATCTTAAGAGGATTAAACTATTCATATTCTTATTTACCTTTATCATGGATAACTGGATTAGTAATATTCTTAATTTCAATTGTTACCGCTTTTATGGGTTATGTATTACCTTGGGGTCAAATGAGTTTCTGGGGTGCAACCGTTATTACTAATTTATTATATTTTATACCTGGACTTGTTTCATGGATTTGTGGAGGATATACTATTAGTGATCCAACTTTAAAAAGATTCTTTGTATTACATTTTATATTTCCTTTTATAGCTTTATGTATTGTATTTATACATATATTCTTCTTACACTTACAAGGTAGCTCTAATCCTTTAGGATATGATACAGCTTTAAAAATACCTTTCTATCCAAGTCTATTATGTCTAGATATCAAAGGATTTAATAATGTATTAGTCCTATTTCTAGCACAAAGTTTATTTGGAATTCT | 479 | North_America, South_America |
| *Haemoproteus* | ZONALB15 | OR063597 | Full | 1 | ZONALB01 | TACTGGAGCTACATTTGTATTTATTCTAACTTACTTACATATTTTAAGAGGATTAAATTATTCATATTCATATTTACCTTTATCATGGATTACTGGATTGGTAATATTTTTAATTTCTATTGTTACTGCTTTTATGGGTTATGTTTTACCTTGGGGTCAAATGAGTTTCTGGGGTGCAACCGTTATTACTAATTTATTATATTTTATACCTGGACTTGTTTCATGGATATGTGGTGGTTATACTATTAGTGATCCAACTCTAAAAAGATTTTTTGTATTACATTTTATATTTCCTTTTATAGCTTTATGCATCGTATTTATACATATATTCTTCTTACATTTACAAGGTAGCTCTAATCCTTTAGGATATGATACAGCTTTAAAAATACCTTTCTATCCAAGTCTATTATGTTTAGATATTAAAGGATTTAATAATATATTAGTTATATTTTTAGCACAAAGTTTATTTGGTATTTT | 477 | - |
| *Haemoproteus* | ZONCAP01 | MK695390 | Full | 1 | APSPI01 | TACCGGTGCTACATTTGTCTTTATTTTAACTTACTTACATATATTAAGAGGATTAAATTATTCATATTCTTATTTACCTTTATCATGGATAACTGGACTAATAATATTCTTAATTTCTATTGTTACCGCTTTTATGGGTTATGTATTACCTTGGGGACAAATGAGTTTCTGGGGTGCAACCGTTATTACTAATTTATTATATTTTATACCTGGACTTGTTTCATGGATTTGTGGAGGATATACTATTAGTGATCCAACTTTAAAAAGATTTTTTGTACTACATTTTATATTTCCTTTTATAGCTTTATGTATTGTATTTATACATATATTCTTCTTACACTTACAAGGTAGCTCTAATCCTTTAGGATATGATACAGCTTTAAAAATACCTTTCTATCCAAGTCTATTATGTCTAGATATCAAAGGATTTAATAATGTATTAGTCCTATTTCTAGCACAAAGTTTATTTGGAATTCT | 477 | South_America |
| *Haemoproteus* | ZOSLAT04 | JX021550 | Full | 1 | ZOSFLA01 | GCTACTGGTGCTACATTTGTCTTTATTTTAACTTATTTACATATATTAAGAGGATTAAATTATTCATATTCATATTTACCATTATCATGGATAACAGGATTAATAATATTTTTAATTTCTATTGTTACTGCTTTTATGGGTTATGTACTACCTTGGGGTCAAATGAGTTTCTGGGGTGCAACCGTTATTACTAATTTATTATATTTTATACCTGGATTAGTTTCATGGATTTGTGGTGGATATATTATTAGTGATCCAACTTTAAAAAGATTTTTTGTATTACATTTTATATTTCCATTTATAGCTTTATGTATTGTATTTATACATATATTCTTTTTACACTTACAAGGTAGCTCTAATCCTTTAGGATATGATACTGCTTTAAAAATACCTTTCTATCCAAGTCTATTATGTCTAGATATTAAAGGATTTAATAATGTATTAGTCTTATTTCTAGCACAAAGTTTATTCGGAATTTT | 479 | Australia_._New_Zeeland |
| *Haemoproteus* | ZOSLUG02 | KT595668 | Full | 1 | ZOSLUG01 | TACATATTTTAAGAGGATTAAATTATTCATATTCATATTTACCTGCATCATGGATAACTGGATTAATTATATTCTTAATTTCTATTGTTACTGCTTTTATGGGTTATGTTTTACCTTGGGGTCAAATGAGTTTCTGGGGTGCAACCGTTATTACTAATTTATTATACTTTATACCTGGACTTGTTTCATGGATTTGTGGTGGATATACTATAAGTGATCCAACCTTAAAAAGATTTTTTGTATTACATTTCATATTTCCATTTATAGCCTTATGTATTGTCTTTATTCATATATTTTTCCTACACTTACAAGGTAGCTCTAATCCTTTAGGATATGATACAGCTTTAAAAATACCTTTCTATCCAAGTCTATTATGTCTAGATATTAAAGGATTTAATAATGTATTAGTTATATTTTTAGCACAAAGTTTATTTGGAATTCT | 442 | South_Sahara |
| *Haemoproteus* | ZOSLUG01 | KT376917 | Full | 1 | ZOSLUG02 | GCTACCGGAGCTACATTTGTTTTTATTCTAACTTACTTACATATTTTAAGAGGATTAAATTATTCATATTCATATTTACCTGCATCATGGATAACTGGATTAATTATATTCTTAATTTCTATTGTTACTGCTTTTATGGGTTATGTTTTACCTTGGGGTCAAATGAGTTTCTGGGGTGCAACCGTTATTACTAATTTATTATACTTTATACCTGGACTTGTTTCATGGATTTGTGGTGGATATACTATAAGTGATCCAACCTTAAAAAGATTTTTTGTATTACATTTCATATTTCCATTTATAGCCTTATGTATTGTCTTTATTCATATATTTTTCCTACACTTACAAGGTAGCTCTAATCCTTTAGGATATGATACAGCTTTAAAAATACCTTTCTATCCAAGTCTATTATGTCTAGATATTAAAGGATTTAATAATGTATTAGTTATATTTTTAGCACAAAGTTTATTTGGAATTCT | 479 | South_Sahara |
| *Haemoproteus* | ZOSPAL06 | MF565820 | Full | 1 | ZOSPAL01 | ACTGGTGCTACATTTGTCTTTATTTTAACTTATTTACATATATTAAGAGGATTAAATTATTCATATTCATATTTACCATTATCATGGATAACAGGATTATTAATATTTTTAATTTCTATTGTTACTGCTTTTATGGGTTATGTACTACCTTGGGGTCAAATGAGTTTCTGGGGTGCAACCGTTATTACTAATTTATTATATTTTATTCCTGGATTAGTTTCATGGATTTGTGGTGGATATATTATTAGTGATCCAACTTTAAAAAGATTTTTTGTATTACATTTTATATTCCCATTTATAGCTTTATGTATTGTATTTATACATATATTCTTTTTACACTTACAAGGTAGCTCTAATCCTTTAGGATATGATACTGCTTTAAAAATACCTTTCTATCCAAGTCTATTATGTCTAGATATTAAAGGATTTAATAATGTATTAGTCTTATTTCTAGCACAAAGTTTATTCGGAATTTT | 476 | Asia |
| *Haemoproteus* | ZOSXAN02 | KX604237 | Full | 1 | ZOSLAT11 | GCTACTGGTGCTACATTTGTCTTTATTTTAACTTATTTACATATATTAAGAGGATTAAATTATTCATATTCATATTTACCATTATCATGGATAACAGGATTAATAATATTTTTAATTTCTATTGTTACTGCTTTTATGGGTTATGTACTACCTTGGGGTCAAATGAGTTTCTGGGGTGCAACCGTTATCACTAATTTATTATATTTTATACCTGGATTAGTTTCATGGATTTGTGGTGGATATATTATTAGTGATCCAACTTTAAAAAGATTTTTTGTATTACATTTTATATTTCCATTTATAGCTTTATGTATTGTATTTATACATATATTCTTTTTACACTTACAAGGTAGCTCTAATCCTTTAGGATATGATACTGCTTTAAAAATACCTTTCTATCCAAGTCTATTATGTCTAGATATTAAAGGATTTAATAATGTATTAGTCTTATTTCTAGCACAAAGT | 465 | Oceania |
